# Amine-to-Halogen Exchange Enables an Amine–Acid Etherification

**DOI:** 10.1021/jacsau.5c01528

**Published:** 2026-02-13

**Authors:** Andrew McGrath, Sandip Kumar Das, Eunjae Shim, Andrew Outlaw, Serik Zhumagazy, Hamid Rashidi Nodeh, Jean-Francois Brazeau, Zhicai Shi, Jennifer D. Venable, Christine Gelin, Paul M. Zimmerman, Tim Cernak

**Affiliations:** † Department of Medicinal Chemistry, College of Pharmacy, 600688 University of Michigan, Ann Arbor, Michigan 48109, United States; ‡ Department of Chemistry, 1259 University of Michigan, Ann Arbor, Michigan 48109, United States; § Therapeutics Discovery, Johnson & Johnson Innovative Medicines, La Jolla, San Diego, California 92121, United States; ∥ KAUST Catalysis Center (KCC), King Abdullah University of Science and Technology KAUST, Thuwal 23955-6900, Saudi Arabia

**Keywords:** etherification, halogenation, amine-acid, C−O cross-coupling, high-throughput experimentation

## Abstract

Reactions that form ethers are used broadly for pharmaceutical, fragrance, materials, and agrichemical applications. We report here an amine–acid etherification reaction that proceeds via a facile amine-halogen exchange and an ester-selective reduction. The method employs free aliphatic amines and carboxylic acids to form C­(sp^3^)–O ether bonds directly. This method allows a diverse range of readily available alkyl amines and acids to be transformed into synthetically valuable alkyl ethers, which can be challenging to access by conventional methods. Our etherification reaction is suitable for late-stage diversification and building block repurposing to expand chemical space access. Additionally, this methodology provides straightforward access to medicinally relevant α-deuterated ethers. Reaction development was facilitated by high-throughput experimentation and computational and experimental mechanistic studies. Furthermore, the deamination strategy can be extended to other nucleophiles, enabling the synthesis of phenolic ethers and a range of halide products from amines. Critically, the distinct recipe of etherification reagents we identified enables selective reduction of esters in the presence of secondary amides and distinctly promotes the one-pot amine–acid etherification, whereas related conditions for ester reduction cannot. Overall, this work establishes a versatile amine-halogen exchange as a platform for constructing structurally diverse ethers from abundant feedstocks.

## Graphical abstract

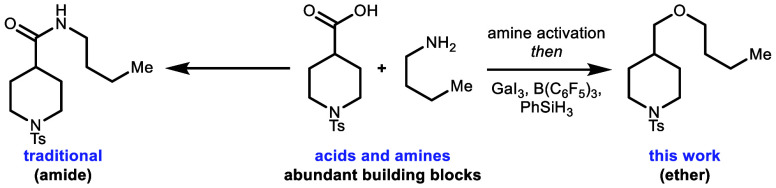

Ethers are one of the most prevalent structural motifs in bioactive molecules.^1–3^ Traditional reactions, such as the Williamson ether synthesis,^4^ are commonly employed to access simple aliphatic ethers from alcohols and alkyl halides, but harsh reaction conditions typically prevent applications with complex substrates. To bridge this gap, complementary methods for ether synthesis have been developed, including metal-catalyzed couplings of alcohols with aryl halides,^5–9^ hydroalkoxylation of alkenes,^10–12^ reductive etherification of alcohols with ketones or aldehydes,^13,14^ and the reduction of esters to ethers.^15,16^ As a further advance to the ether synthesis toolbox, we envisioned a mild method based on two of the most broadly available building blocks: amines and carboxylic acids. While a carboxylic acid is easily envisaged as an oxidized ether precursor, leveraging abundant amine building blocks would require a robust amine-activation strategy. We disclose here an approach based on in situ amine-to-halogen exchange, a transformation that converts amines into alkyl halides under mild conditions, enabling direct coupling of amines with carboxylic acids to deliver ethers following reduction in a one-pot process (**1** + **2** → **3**, Figure 1A)

**Figure 1:**
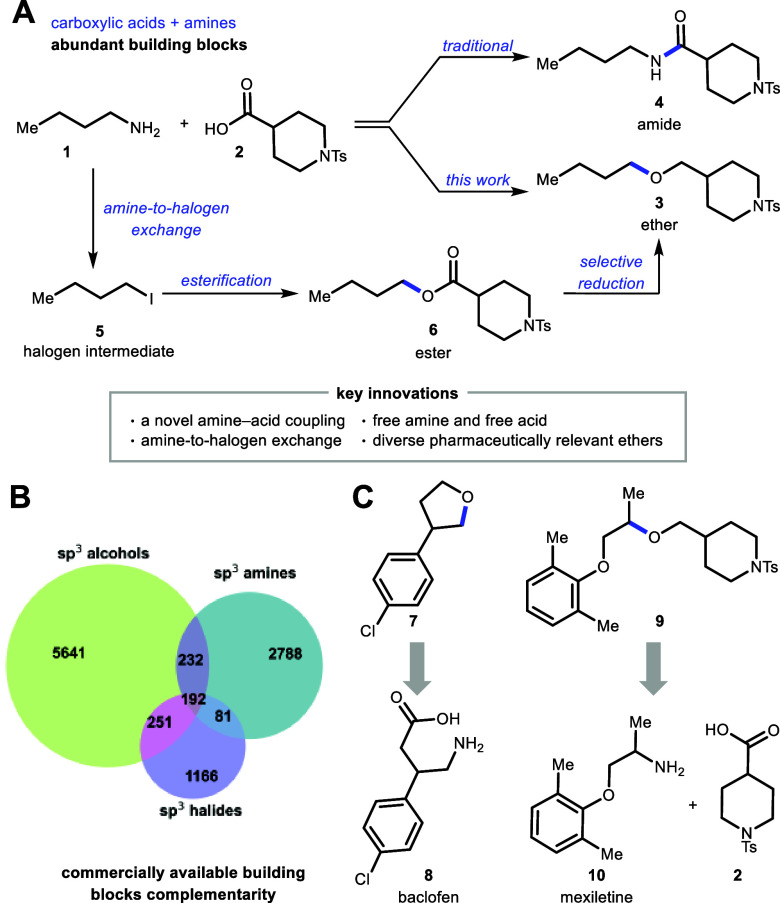
(A) Our approach utilizes amines and carboxylic acids in ether synthesis as a complement to amide coupling. (B) Analysis of available amines (blue) and alcohols (green) as well as available primary and secondary halides (purple) from MilliporeSigma shows each class of building blocks covers a unique chemical space. Of the ∼10k commercially available building blocks analyzed, only 192 can be purchased with an amine, alcohol, or halide functional handle. (C) Diverse alkyl ethers from pharmaceutical amines and acids.

Amines and carboxylic acids are abundant and structurally diverse feedstocks, and we have been exploring their use in a range of transformations to complement conventional amide formation.^17–26^ While substantial advances have been made in forging C–C bonds from these precursors,^18,20,27–32^ direct methods for C–O bond formation from amines and acids remain comparatively rare.^21,33–38^ A seminal contribution by Katritzky demonstrated that amines could be transformed into esters by activation as pyridinium salts; however, harsh conditions, such as heating above 170 °C under vacuum, limited the scope, and the isolation of the intermediate salt introduced an extra step.^33^ To address these limitations, we recently reported a mild synthesis of esters from carboxylic acids and amine-derived pyridinium salts.^21^ This esterification is a powerful addition to the amine-acid coupling toolkit—and has yielded esters of superior biological activity relative to amide coupled analogs (**1 + 2 → 4**) when used in the synthesis of heterobifunctional protein degraders.^24^ We were drawn to an amine–acid etherification as a coupling of especially high relevance to medicinal chemistry.^39^ To develop one we explored the mechanistic role of potassium iodide (KI), a required additive in our esterification reaction, whose purpose was previously unclear. Here we show that the conversion of pyridinium salts into primary alkyl halides (cf. **5**), with KI and other halide salts, is a remarkably facile process. This amine-to-halogen exchange is leveraged to achieve the first amine–acid etherification (Figure 1A). Our one-pot strategy, which employs free amines with carboxylic acids, proceeds through three sequential steps: (i) amine-to-halogen exchange followed by (ii) esterification and (iii) ester-selective reduction to the ether. In this way, amines, which are available in a wider structurally diversity than alkyl halides, can serve as unconventional ether precursors. We conducted a survey of commercially available building blocks from MilliporeSigma (Figure 1B), which revealed that more than twice as many amines can be purchased relative to primary and secondary alkyl halides, with minimal overlap in substituent patterns. When expanded to include alcohols, it becomes clear that each functionality provides access to a distinct region of chemical space. Thus, an amine–acid etherification platform complements existing ether synthesis strategies by opening opportunities for chemical space exploration. Bioactive amine and acid building blocks are broadly available, making an operationally simple etherification a viable strategy for late-stage diversification such as the formation of **7** from baclofen (**8**) or of ether **9** from mexiletine (**10**) and **2** (Figure 1C).

To achieve this one-pot etherification, we selected *N*-tosyl isonipecotic acid (**2**) and *n*-butyl amine (**1**) as model substrates, 2,4,6-triphenylpyrylium tetrafluoroborate (**11**) as an activator, and KI as a promoter. While the amine-to-halogen transformation followed by the esterification proceeded smoothly to produce ester **6** under our previously reported conditions,^21^ one-pot reduction of ester **6** to the ether (**3**) was initially unsuccessful (see Supporting Information Figure S1). To identify suitable conditions for the etherification step, we next embarked on a high-throughput experimentation (HTE)^40^ reaction optimization campaign. Experimentally, ester **6** was prepared in bulk and the crude reaction mixture served as a stock solution to dose into a mixture of other reagents when building the HTE array, facilitating a three-step, one-pot protocol (Figure 2A). We began our investigation with a reaction array using four oxophilic metals, specifically, gallium,^15^ iron,^41^ indium,^16^ or aluminum,^42^ either alone or in combination with boron-based cocatalysts such as trispentafluorophenylborane [B­(C_6_F_5_)_3_], or triphenyl borane (Ph_3_B), and using either diphenylsilane (Ph_2_SiH_2_) or 1,1,3,3-tetramethyldisiloxane (TMDS) as a terminal reductant (Figure 2A, entry 1). An initial reaction array using 3.0 equiv of each oxophilic metal and 0.3 equiv of additive, if present, yielded the desired ether **3** in 7 of 24 wells.In contrast, using a catalytic amount of oxophilic metal (0.3 equiv.) failed to give ether 3 in any well regardless of the presence of borane (see the Supporting Information for details). Gallium tribromide (GaBr_3_) consistently delivered the desired product, regardless of whether a cocatalyst was used. Additionally, aluminum trichloride (AlCl_3_) was able to deliver desired ether **3** in 29% assay yield but only when using B­(C_6_F_5_)_3_ as a cocatalyst (Figure 2A, entry 1). These results informed the next array of 96 reactions. Halide variants of gallium and aluminum salts were surveyed with three boron-derived Lewis acids, a blank, and four silane reductants (Figure 2A, entry 2). From this HTE array, the combination of gallium triiodide (GaI_3_), Ph_3_B, and Ph_2_SiH_2_ emerged as the highest yielding conditions, furnishing ether **3** in 36% assay yield. Further exploration of other boranes, gallium sources, and solvents provided only a marginal increase in yield (see the Supporting Information for details). We then turned our attention toward additives that have been shown to have a beneficial effect in ester reductions.^43,44^ Specifically, Lewis and Brønsted acidic additives as well as borate salts were evaluated to improve yield. An array of six additives, two boranes, and two equivalents of gallium salts (0.5 and 3.0 equiv) revealed trimethylsilyl chloride (TMSCl) as a key additive to increase the assay yield to 58%, though the required stoichiometry of gallium additive could not be reduced (Figure 2A, entry 3). With this new data, one final six-by-four array was performed, examining various silyl halides and their interplay with borane Lewis acid additives (Figure 2A, entry 4). This array revealed trimethoxysilyl chloride [(MeO)_3_SiCl] to be the highest yielding silyl halide when used with GaI_3_, boron-based Lewis acids B­(C_6_F_5_)_3_ or B­(Mes)_3_, and reductant Ph_2_SiH_2_, providing ether 3 in 71% assay yield (63% isolated yield). Further studies revealed that purification was generally more facile when phenylsilane was used in place of Ph_2_SiH_2_ (see Supporting Information), resulting in GaI_3_, B­(C_6_F_5_)_3_, (MeO)_3_SiCl, and phenylsilane as the final optimized conditions.

**Figure 2:**
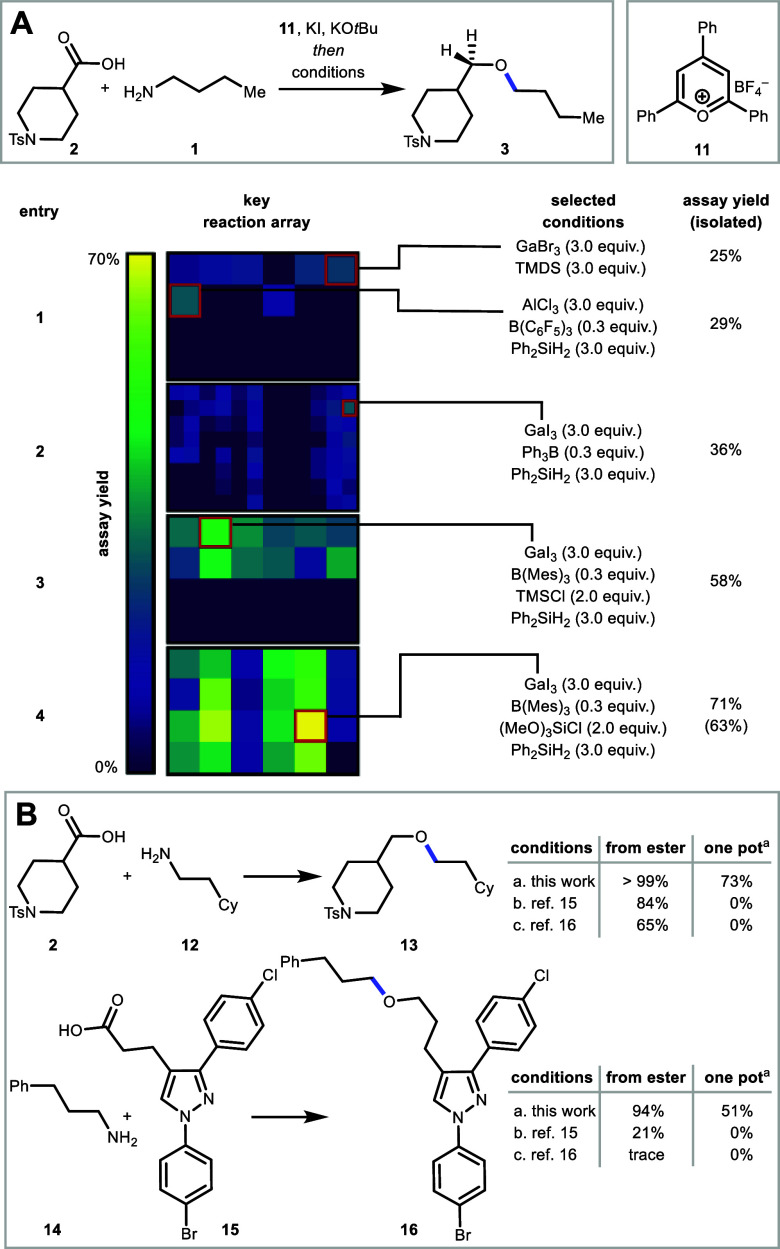
(A) HTE optimization on 10 μmol scale in 24 and 96-reaction arrays. Assay yields were determined by UPLC-MS. Isolated yields were determined by repeating the reaction on 0.20 mmol scale. Etherification reactions were done at 65 °C. Ts = *p*-toluenesulfonyl. TMDS = 1,1,3,3-tetramethyldisiloxane. Mes = 2,4,6-trimethylphenyl. TMSCl = trimethylsilyl chloride. (B) Comparison of our reductive etherification to reported methods. From isolated ester and free amine/acid. ^a^For one-pot reaction, 3.0 equiv. GaI_3_ is used. Conditions: (a) 50 mol % GaI_3_, 25 mol % B­(C_6_F_5_)_3_, 2.0 equiv. (MeO)_3_SiCl, 2.0 equiv. PhSiH_3_, dioxane, 65 °C, 1.5 h. (b) 1 mol % GaBr_3_, 1.1 equiv. TMDS, 60 °C, 1 h (ref 15). (c) 5 mol % InBr_3_, 4.0 equiv. Et_3_SiH, CHCl_3_, 60 °C, 1 h (ref 16). NMR yields determined by ^1^H NMR using 1,3,5-trimethoxybenzene as internal standard (see Supporting Information for details and full array recipes).

To demonstrate the efficacy of our developed ester reduction conditions, we conducted a head-to-head comparison with previously reported methodologies (Figure 2B). For this comparison, we examined the reduction of both purified ester intermediates and one-pot transformations directly from free amines and acids. When the purified ester was subjected to our optimized conditions (conditions a), quantitative conversion to **13** was observed by NMR analysis. In contrast, the previously reported methods (conditions b and conditions c) by Biermann^15^ and Sakai^16^ afforded ether **13** in slightly lower crude NMR yields of 84% and 65%, respectively. In sharp contrast, etherification starting from free amine (**12**) and acid (**2**) delivered a 73% NMR yield of ether **13** by our method, while no product formation was observed using either of the existing methods. We further evaluated a more complex heterocyclic acid (**15**) and amine (**14**) under all three reduction protocols. Using the purified ester intermediate, our method achieved a 94% NMR yield of the ether product **16**, while the Biermann and Sakai conditions afforded only 21% and trace amounts, respectively. Starting directly from the free amine and acid, our protocol provided ether (**16**) in 51% yield, whereas no product was observed with either of the reported methods. These findings demonstrate the superior efficiency of our method for ester reduction, and the only one capable of achieving etherification coupling from a free amine and a free acid.

With the optimal conditions in hand, the scope of the reaction was explored next (Figure 3). Both alpha-primary amine (for example **13**–**23**, 26–**42**) and alpha-secondary amine (**24**, **25**, **9**, **43**) substrates were employed. A variety of functional groups were tolerated, including alkenes (**34**, **39**), ethers and thio ethers (**18**, **20**), heterocycles (**19**, **23**, **28**, **29**, **35**, **36**, **16**, **38**), aryl halides (**21**, **7**, **16**, **38**), and basic amines (**27**, **35**, **38**). Intramolecular etherification was also successful (**7**). When purified ester was used as the starting material, the GaI_3_ loading could be reduced to 0.5 equiv, suggesting byproducts of the esterification reaction, likely 2,4,6-triphenylpyridine, poison the gallium additive. Moreover, this method is convenient to set up, retaining comparable performance when conducted inside or outside an inert atmosphere glovebox (49% vs 55% for product **13**). Amines and acids are prevalent in drug molecules, and we explored late-stage diversification using our method. Etherification was successful using baclofen (**7**), hippuric acid (**31**), isocaproic acid (**32**), isoleucine (**33**), oleyl amine (**34**), urapidil (**35**), oxaprozin (**36**), gemfibrozil (**37**), an intermediate from an antituberculosis drug (**16**), cetirizine (**38**), and citronellic acid (**39**) as substrates. Given the prevalence of deuterium in pharmaceuticals.^45^ we pondered whether we could incorporate deuterium into metabolically labile α-hetero C–H bonds by changing the reductant to Ph_2_SiD_2_. This protocol successfully produced deuterated ethers (**40**–**43**) in 55–65% yield with >95% deuterium incorporation, as determined by NMR.

**Figure 3:**
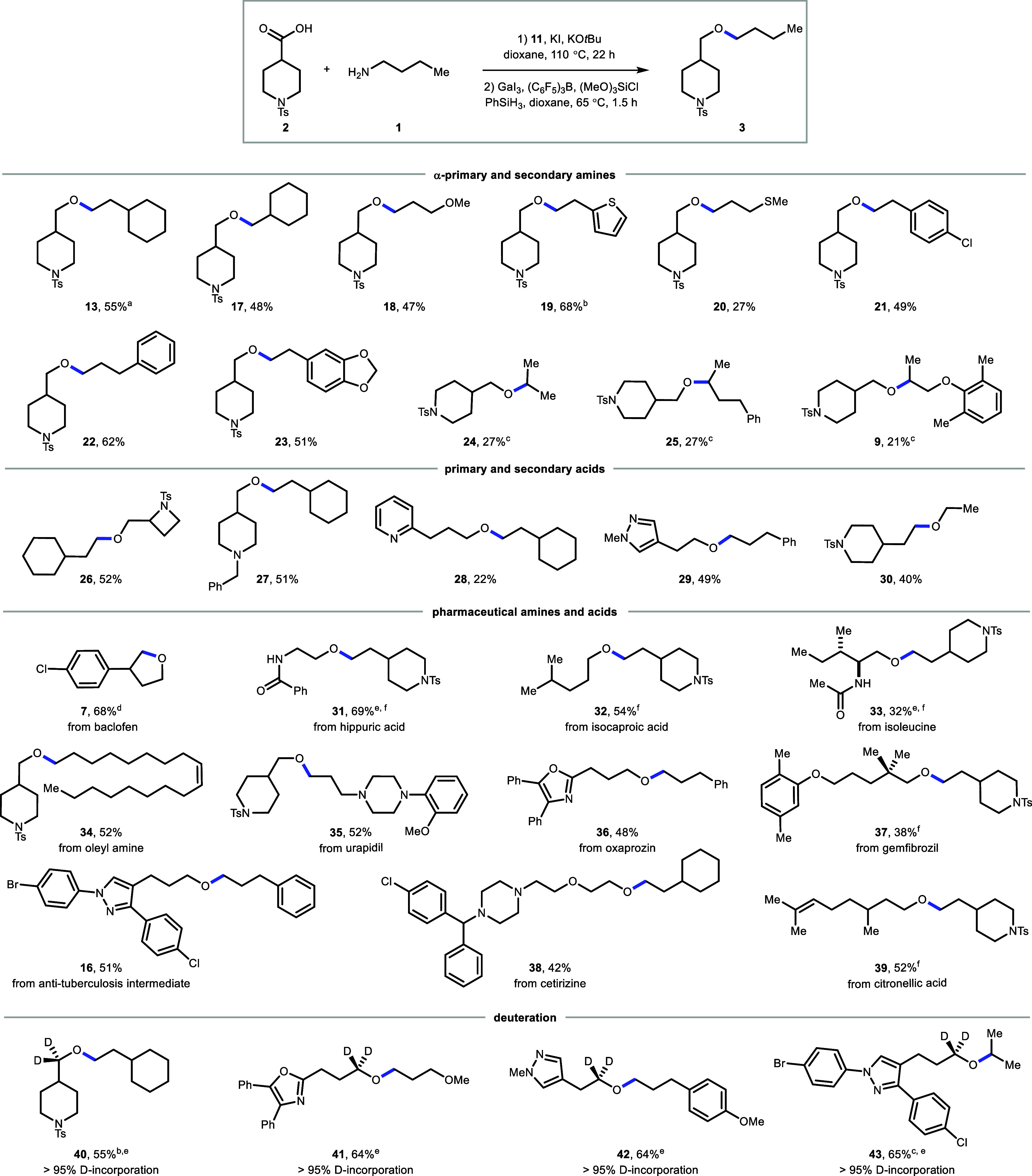
Substrate scope of amine–acid reductive etherification on 0.10–0.25 mmol scale. Esterification reactions were performed with KI (1.5 equiv), *t*BuOK (1.0–2.0 equiv), **11** (1.0 equiv). Etherification reactions were conducted with GaI_3_ (3.0 equiv), B­(C_6_F_5_)_3_ (0.3 equiv), (MeO)_3_SiCl (2.0 equiv), PhSiH_3_ (3.0 equiv). Isolated yields are shown. ^a^Reaction performed outside the glovebox delivered the desired ether in 49% yield. ^b^Performed with B­(Mes)_3_ (0.4 equiv). ^c^Esterification step was run at 80 °C. ^d^With DIPEA (1.0 equiv) as a base. ^e^Using purified ester and Ph_2_SiH_2_ or Ph_2_SiD_2_ as a reductant. ^f^2.0 equiv. *t*BuOK. See the Supporting Information for detailed reaction conditions.

To expand the scope of our reaction, we were interested in developing a C­(sp^2^)–C­(sp^3^) etherification using phenols instead of carboxylic acids (Figure 4A). We investigated using phenol **44** in combination with atorvastatin intermediate **45** and found that, through modification of the esterification protocol (see Supporting Information for reaction condition studies), we isolated ether **46** in 64% yield in a two-step one-pot protocol. This protocol was used to alkylate ivacaftor to give **47** in 40% yield. We also used it to forge a bond between lysine and tyrosine (**48**). Finally, our method was applied to an amine targeting the E3 ligase von Hippel-Lindau (VHL)^46^ and estradiol yielding **49** in 25% yield and highlighting the utility of this strategy in synthesizing proteolysis targeting chimeras (PROTACs). Next, omitting carboxylic acid from the reaction with **50** allowed for the isolation of alkyl iodide (**51**) (Figure 4B). By switching the halide salt, we were able to isolate the corresponding bromide (**52**) and chloride (**53**) analogs of **50**. This halogenation method was further applied to four other amines, resulting in the isolation of various halogenated derivatives (**54**–**64**), providing facile access to halogens from amines (see Supporting Information).^47^

**Figure 4:**
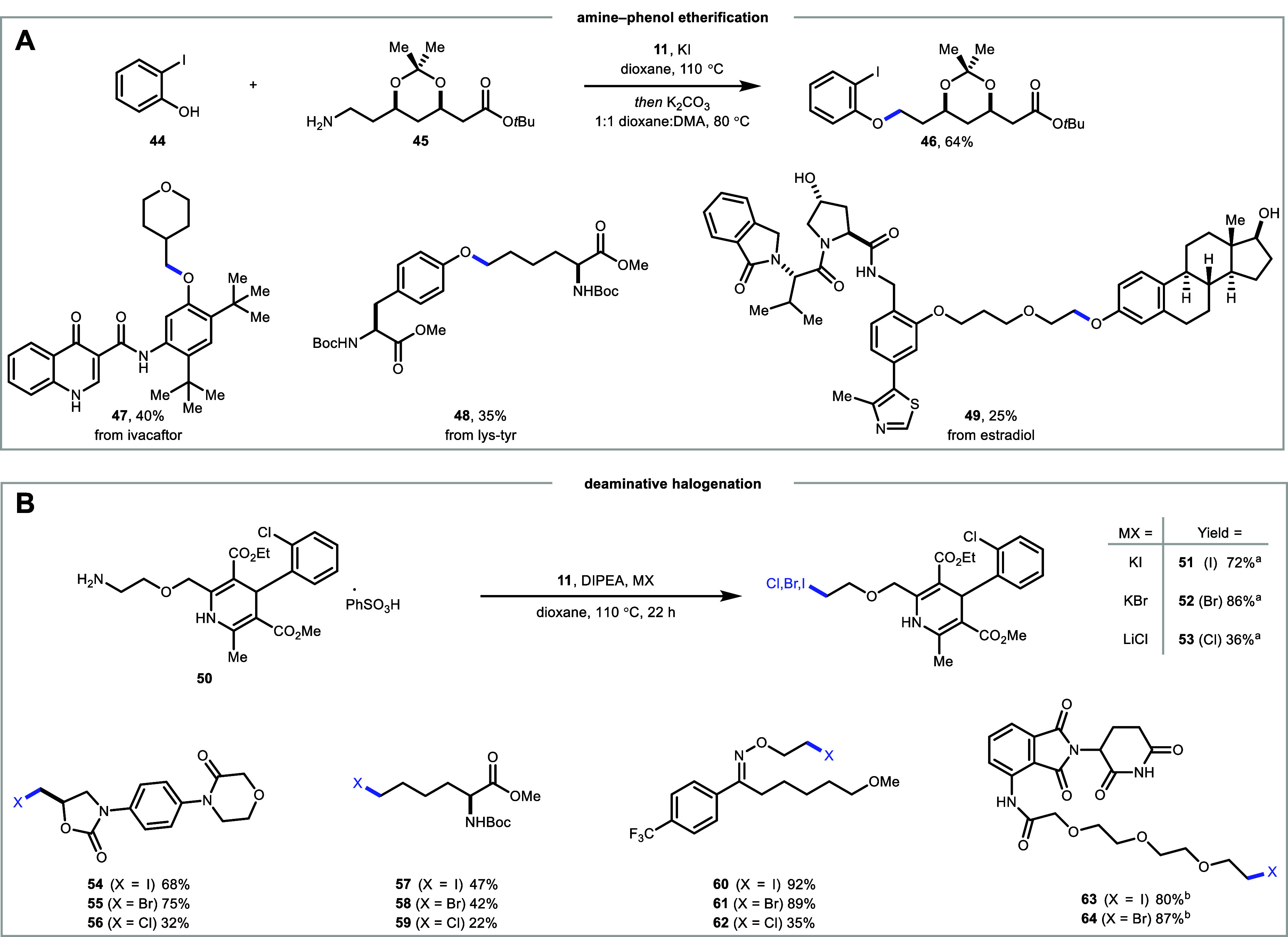
(A) Scope of etherification using various phenols. (B) Preparation of various alkyl halogenated compounds using complex molecules as an amine source. Isolated yields are shown. ^a^DMA as a solvent. ^b^2,4,6-tris­(4-(trifluoromethyl)­phenyl) pyrylium tetrafluoroborate as an activator.

Based on literature precedent,^34^ we proposed a mechanism for ether formation (Figure 5A). In this mechanism, amine (**14**), in the presence of 2,4,6-triphenylpyrylium tetrafluoroborate (**11**), is first converted into the corresponding pyridinium salt (**65**). This intermediate undergoes an S_N_2 reaction in the presence of KI, producing an alkyl iodide intermediate (**66**) and 2,4,6-triphenylpyridine as a byproduct, which was confirmed by LC-MS. Subsequently, the acid (**2**), in the presence of a base, undergoes nucleophilic displacement (S_N_2) with alkyl iodide intermediate (**66**) to form the ester (**67**). The ester is then reduced to yield the desired ether (**22**). We next conducted kinetic analysis of the three key transformations in our sequence: halogenation, esterification, and etherification (Figure 5A). In the halogenation step, rapid formation of the pyridinium salt intermediate (**65**) was observed within the first hour, followed by its gradual consumption to give the corresponding halide (**66**), with complete conversion achieved after ∼22 h. This behavior was also evident from visual inspection, as the immediate coloration of the reaction mixture indicated pyridinium salt formation, while subsequent loss of color progressed with halide formation. In contrast, esterification of halide (**66**) with acid (**2**) in the presence of potassium *tert*-butoxide proceeded much more rapidly, reaching full conversion within 3 h. The final etherification step, involving reduction of ester (**67**) to ether (**22**), displayed an even faster profile: 50% product conversion was achieved within 30 min, and complete consumption of ester (**67**) was observed within 1.5 h, affording ether (**22**) in 69% NMR yield. These studies collectively demonstrate distinct kinetic profiles for each transformation, with halogenation proceeding more slowly relative to the rapid esterification and etherification steps.

**Figure 5:**
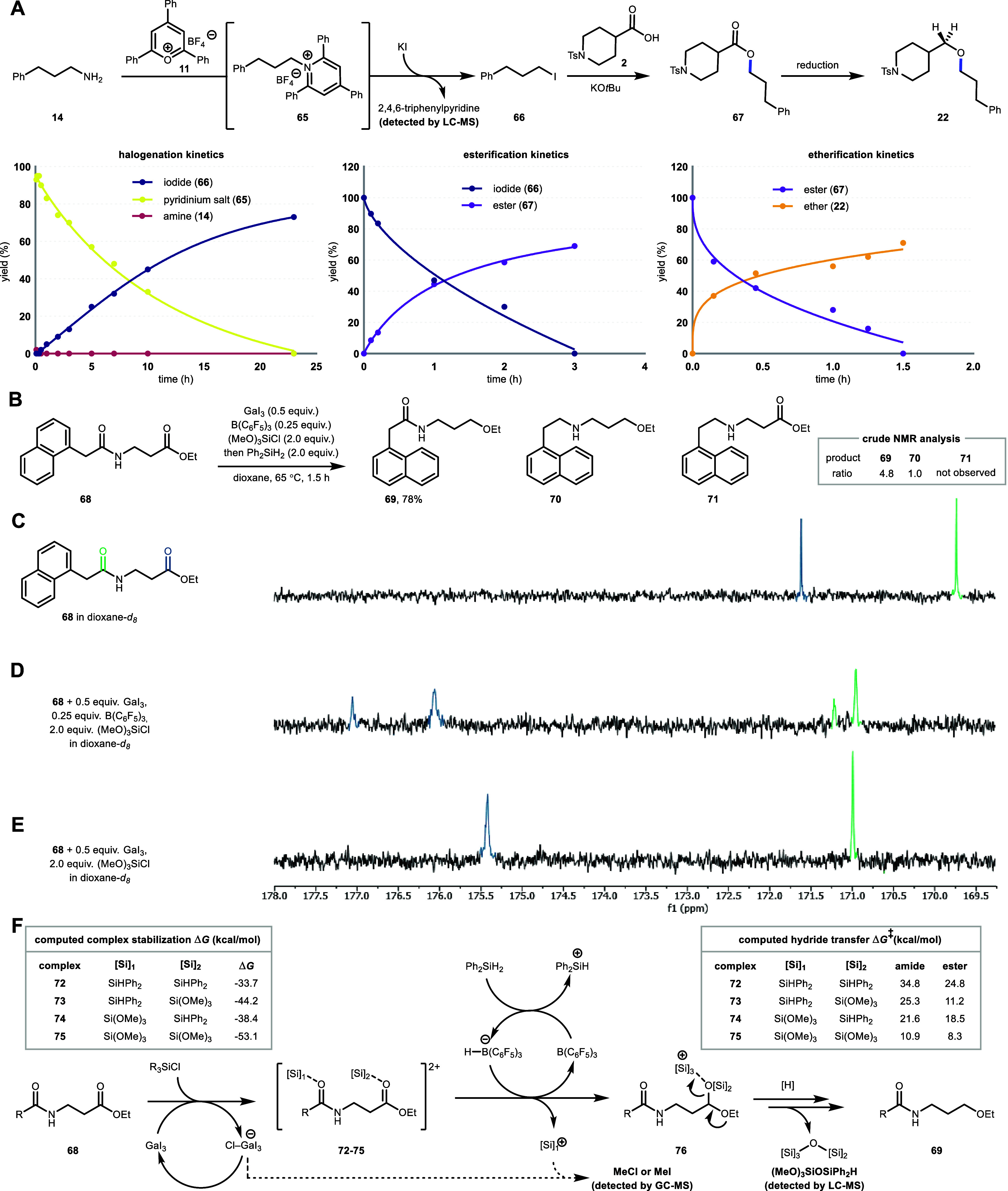
(A) Proposed mechanism of ether formation and kinetics experiment on halogenation, esterification, and etherification steps. Each step was studied independently to assess reaction progress and establish mechanistic insights. (B) Selective reduction of **68**. (C)^13^C NMR of the carbonyl region of **68**. (D) **68**, 0.5 equiv of GaI_3_, 0.25 equiv of B­(C_6_F_5_)_3_, and 2.0 equiv of (MeO)_3_SiCl. (E) **68** and 0.5 equiv of GaI_3_, and 2.0 equiv of (MeO)_3_SiCl in dioxane-d_8_. The more downfield peak corresponds to the ester carbonyl carbon. See the Supporting Information for full spectra. (F) Proposed mechanism of selective ester reduction.

Remarkably, selective reduction of esters to ethers in the presence of amides was achieved (**31**, **33**), although it was necessary to isolate the intermediate esters to lower the gallium loading required. This intriguing selectivity was studied further by reducing **68**, obtaining 78% yield of ether **69**, which formed along with doubly reduced product **70** in a 4.8:1 ratio, while no mono-reduction of the amide (**71**) was observed (Figure 5B). Hypothesizing that this selectivity stems from the activation of two carbonyls, the role of each reagent and the order of addition were evaluated. The necessity of all reagents was first confirmed with control experiments (see Supporting Information). Then, the impact of different reagent addition orders on conversion was assessed, revealing the importance of adding (MeO)_3_SiCl after the two primary Lewis acids but before the reductant (see Supporting Information). Based on this result, how B­(C_6_F_5_)_3_, GaI_3_, and (MeO)_3_SiCl interact with **68** was studied by NMR. When **68** was treated with the two Lewis acids followed by (MeO)_3_SiCl, the ester and amide carbonyl carbon signals split in the ^13^C NMR spectrum and shifted downfield to 177.0, 176.0 ppm and 171.1, 170.8 ppm pairs, respectively (Figure 5D). ^19^F-NMR of this mixture showed no change in the fluorine peaks of B­(C_6_F_5_)_3_ alone, suggesting nonparticipation of B­(C_6_F_5_)_3_ in the activation of the two carbonyls (see Supporting Information). When **68** was treated with GaI_3_ and (MeO)_3_SiCl, ^13^C NMR analysis also showed shifted carbonyl peaks at 175.3 and 170.9 ppm (Figure 5E), further supporting the role of these additives in activating **68** (see Supporting Information Spectrum S1–S25 for NMR spectra of **68** when treated with different combinations of reagents). Collectively, these studies lead to the hypothesis that GaI_3_, (MeO)_3_SiCl and the substrate interact to activate a carbonyl group.^44^ B­(C_6_F_5_)_3_, which is a spectator at this point, is thought to interact with the silane reductant and act as a hydride shuttle, as earlier proposed by Piers et al.^48^

The activation of carbonyls via silicon-based Lewis acids was next studied in detail with density functional theory simulations (see Supporting Information for further details). First, the energies of silyl ion binding to carbonyls (**72**–**75**) were compared under the assumption that both carbonyls are activated before hydride transfer. Out of the four possibilities, trimethoxy silylium binding to both carbonyls (**75**) showed the largest stabilization (Figure 5F, left table). These four complexes were then computationally subjected to hydride transfer from the H–B­(C_6_F_5_)_3_ borate anion to either carbonyl to gain insight into the origin of regioselectivity. In all cases, the barrier leading to the ether is lower than that leading to the amine, regardless of the activation modes considered (Figure 5F, right table). The lowest barrier among them was from **75**, which has trimethoxy silylium binding to both carbonyls of **68**. These results are in line with the order of addition studies, supporting the initial importance of activating the carbonyl groups of the substrate with (MeO)_3_SiCl and GaI_3_. To further support the mechanistic hypotheses, side products were identified using mass spectrometry methods. GC-MS analysis of the reaction headspace detected chloromethane and iodomethane in significant quantities (see the Supporting Information), which may form from the reaction between gallium tetrahalide anion and a trimethoxy silyl cation as the methyl source (Figure 5F, dashed arrow). On the other hand, LC-MS showed a significant amount of trimethoxy silyl diphenyl silyl ether (see Supporting Information). The presence of this intermediate suggests that after hydride transfer, the resultant diphenyl silyl species assists in the activation of silyl acetal 76. Taken together, a plausible reaction pathway is proposed where the activation of carbonyl groups proceeds through silyliums,^44^ presumably trimethoxy silylium—which is subsequently reduced (Figure 5F). Among Lewis acids, gallium halides seem privileged in their selective activation of esters. This is demonstrated by the examination of various Lewis acids, which identified gallium triiodide as the selective activator: while platinum, and other group 16 metals are able to effect reduction of esters in the absence of amides, none proved to be selective in the presence of amides (see Supporting Information).

Finally, to examine the selective reduction of an ester over an amide, we conducted an experiment using substrates **68** and **77** for comparison of our protocol to reported conditions (Figure 6).^15,16^ With our method applied to substrate **68**, a mixture of ether (**69**) and the doubly reduced product (**70**) was obtained in crude NMR yields of 70% and 17%, respectively. In contrast, Biermann’s conditions delivered only 6% of desired product **69**, and 17% of over-reduced product **70**. For substrate **77**, our protocol delivered ether **31** and **78** in 70% and 18% NMR yield, respectively, whereas no product formation was observed under the Biermann conditions. The Sakai methodology was ineffective for generating either product in both examples tested.

**Figure 6:**
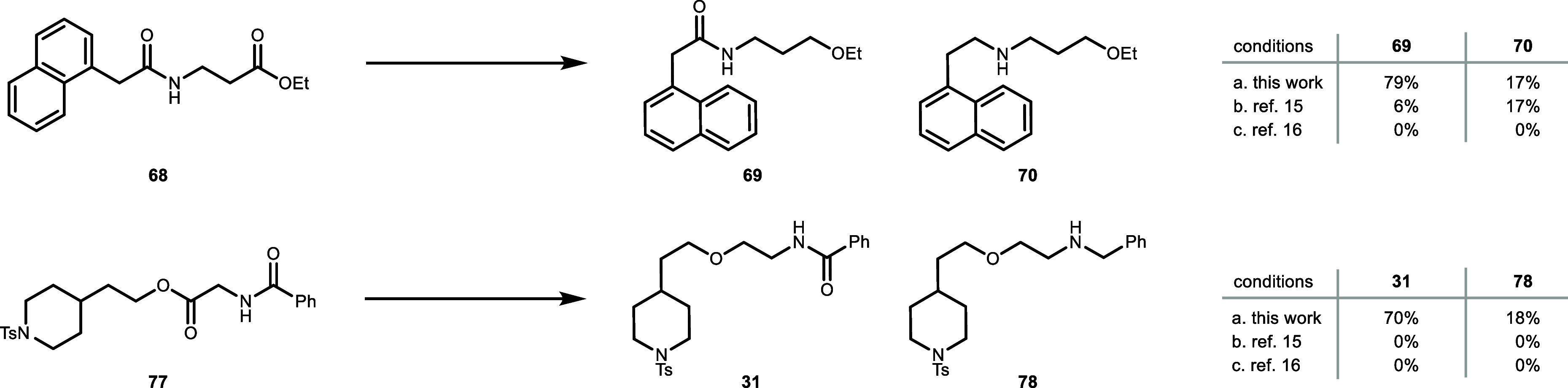
Comparison of our developed selective ester reduction in the presence of amide with reported methods. Ph_2_SiH_2_ was used as a reductant. Conditions: (a) 50 mol % GaI_3_, 25 mol % B­(C_6_F_5_)_3_, 2.0 equiv. (MeO)_3_SiCl, 2.0 equiv. Ph_2_SiH_2_, dioxane, 65 °C, 1.5 h. (b) 1 mol % GaBr_3_, 1.1 equiv. TMDS, 60 °C, 1 h (ref 15). (c) 5 mol % InBr_3_, 4.0 equiv. Et_3_SiH, CHCl_3_, 60 °C, 1 h (ref 16). Yields determined by NMR using 1,3,5-trimethoxybenzene as internal standard (see Supporting Information for details).

In summary, we have developed a deaminative etherification of aliphatic amines with carboxylic acids, enabled by amine–halogen exchange and ester-selective reduction. This method has proven successful in generating structurally diverse alkyl–alkyl ether bonds. We systematically explored the versatility of the approach, demonstrating its compatibility with complex drug molecules. A two-step one-pot protocol initiating directly from the free amine, showcases the versatility of the method. Furthermore, the deamination strategy can be extended to other nucleophiles, enabling the synthesis of phenolic ethers and various halide products. The reaction is selective for the reduction of esters over amides, proceeding through gallium-catalyzed activation of a silyl chloride and boron–mediated hydride reduction. Given the abundance of primary amines and acids from common feedstock chemicals, we anticipate that this operationally simple deaminative etherification will find widespread application in the preparation of both synthetically and biologically important ethers.

## Supplementary Material



## Notes

Full characterization, copies of all spectral data and experimental procedures (PDF)
