# Supplementary material for: Amine-to-Halogen Exchange Enables an Amine–Acid Etherification
Source: JACS Au. 2026 Feb 13;6(3):1667–75. doi: 10.1021/jacsau.5c01528 (PMC13014198; doi:10.1021/jacsau.5c01528)
Supplement: Supplementary file 1 [file au5c01528_si_001.pdf]

Supporting information for

Amine to Halogen Exchange Enables an Amine-Acid Etherification

Andrew McGrath[1], Sandip Kumar Das[1], Eunjae Shim[2], Andrew Outlaw[1], Hamid Rashidi Nodeh[1], Jean-Francois Brazeau[3], Zhicai Shi[3], Jennifer D. Venable[3], Christine Gelin[3], Paul M. Zimmerman[2], Timothy Cernak[1,2 *]

[1]Department of Medicinal Chemistry, College of Pharmacy, University of Michigan, Ann Arbor, Michigan 48109, United States.

[2]Department of Chemistry, University of Michigan, Ann Arbor, Michigan 48109, United States.

[3]Therapeutics Discovery, Janssen Research & Development, LLC, Welsh & McKean Roads, Spring House, Pennsylvania 19477, United States.

*Corresponding author: tcernak@umich.edu

Contents

General Information…………S2

General Procedures…………S3

General Procedure A: Deaminative Esterification…………S3

General Procedure B: Amine–Acid Reductive Etherification…………S3

General Procedure B: Amine–Acid Reductive Etherification (no glovebox) …………S4

General Procedure C: Reduction of Purified Esters to Ethers…………S4

General Procedure D: Preparation of Ethers from Phenols and Amines…………S5

General Procedure E: Preparation of Alkyl Halides from Amines…………S5

General Procedure F: Platinum Catalyzed Ether Synthesis from Pyridinium Salts and Carboxylic Acids…………S5

General Procedure G: Preparation of Pyridinium Salts from Corresponding Amines…………S6

General Procedure H: Procedure for High-Throughput Experimentation in 24- and 96-Well Plates…………S6

Troubleshooting Tips…………S7

Preparation of Starting Materials…………S9

HTE Optimization of Deaminative Reductive Etherification…………S14

Extended Etherification Optimization Data…………S27

NMRs of Mechanistic studies…………S29

Reagent Control and Order of Addition Experiments…………S54

Mechanistic Experiments…………S54

Kinetic Experiments…………S55

Discovery of a Platinum-catalyzed Reductive Etherification…………S57

Extended Platinum Catalyzed Etherification Optimization ……………………………S63
Platinum vs Gallium Comparison……………………………………………………...S65
Comparison of Our Developed Reductive Etherification Method with Previously Reported Etherification Methods……………………………………………………………………S66
Deaminative Amine–Phenol Etherification Optimization………………………………S67
Products Characterization Data…………………………………………………………S68
Computational Details…………………………………………………………………....S91
References ……………………………………………………………………………...S96
NMR Spectra……………………………………………………………………………S98

General Information

All reactions were conducted in oven- or flame-dried glassware under an atmosphere of nitrogen unless stated otherwise. Reactions were set up in an MBraun LABmaster Pro Glove Box (H_2O level <0.1 ppm, O_2 level <0.1 ppm), or using standard Schlenk technique with a glass vacuum manifold connected to an inlet of dry nitrogen gas. Acetonitrile (MeCN) was purified using an MBraun SPS solvent purification system by purging with nitrogen and then passing the solvent through a column of activated alumina. 1,4-dioxane, *N,N*-dimethylacetamide (DMA), and dimethyl sulfoxide (DMSO) were purchased as anhydrous solvents and used as received. Reagents were purchased from Sigma Aldrich, Ambeed, Alfa Aesar, Oakwood Chemical, or TCI Chemical. Liquid primary amines were passed through a plug of basic alumina before use. Potassium iodide was ground with a mortar and pestle before use. Molecular sieves were dried at 200 °C under vacuum overnight and stored in an oven. All other chemicals were used as received. Glass 1 dram (Fisher Scientific #03-339-21B) or 2-dram vials (Fisher Scientific #03-339-21D) were used as reaction vessels, fitted with a screw cap and Teflon-coated silicone septa (ChemGlass #CG-4910-02), and magnetic stir bars (Fisher Scientific #14-513-93 or #14-513-65).

Proton nuclear magnetic resonance (^{1}H NMR) spectra were recorded on a Varian MR-500 MHz, or Varian MR-400 MHz spectrometer and chemical shifts are reported in parts per million (ppm) using the solvent residual peak as an internal standard ($CDCl_3$ at 7.26 ppm, DMSO-d_6 at 2.50 ppm). Data are reported using the abbreviations: app = apparent, s = singlet, d = doublet, t = triplet, q = quartet, m = multiplet, comp = complex, br = broad. Coupling constant(s) are reported in Hz. Proton-decoupled carbon nuclear magnetic resonance spectra (^{13}C NMR) spectra were recorded on a Varian MR-500 MHz or Varian MR-400 MHz spectrometer and chemical shifts are reported in ppm using the solvent as an internal standard ($CDCl_3$ at 77.16 ppm, DMSO-d_6 at 39.52 ppm). 19F NMR spectra were recorded on the same spectrometers as above. 13C and 19F signals are singlets unless otherwise stated. 1H-COSY, HSQC and HMBC were used where appropriate to facilitate structural determination.

High resolution mass spectrometry data (HRMS) were obtained on an Agilent 6230 TOF LC/MS equipped with ESI detector in positive mode and on a Micromass AutoSpec Ultima Magnetic Sector instrument with EI detector in positive mode. Reaction analysis was typically performed by thin-layer chromatography on silica gel or using a Waters I-class ACQUITY UPLC-MS (Waters Corporation, Milford, MA, USA) equipped with in-line photodiode array detector (PDA), evaporative light scattering detector (ELSD) and QDa mass detector (Both ESI positive and

negative ionization mode). Typically, 0.1 µL sample injections were taken from acetonitrile solutions of reaction mixtures or products (~1 mg/mL). A partial loop injection mode was used with the needle placement at 2.0 mm from bottom of the wells and a 0.2 µL air gap at pre aspiration and post-aspiration. Column used: Waters Cortecs UPLC C18+ column, 2.1mm × 50 mm with (Waters S5 #186007114) with Waters Cortecs UPLC C18+ VanGuard Pre-column 2.1mm × 5 mm (Waters #186007125), Mobile Phase A: 0.1 % formic acid in Optima LC/MS-grade water, Mobile Phase B: 0.1% formic acid in Optima LC/MS-grade MeCN. Flow rate: 0.8 mL/min. Column temperature: 45 °C. The PDA sampling rate was 20 points/sec. The QDa detector monitored m/z 150-750 with a scan time of 0.06 seconds and a cone voltage of 30 V. The ELSD had a gain of 750, data rate of 10 pps, time constant "normal" 0.2000 sec, a gas pressure of 40.0 psi, with the nebulizer in cooling mode at 75% power level and the drift tube temperature set to 50 °C. The PDA detector range was between 210 nm – 400 nm with a resolution of 1.2 nm. 2 S–3 minute and 8-minute methods were used. The method gradients are below: 2 min method, 0 min: 0.8 mL/min, 95% 0.1% formic acid in water/5% 0.1% formic acid in acetonitrile; 1.5 min: 0.8 mL/min, 0.1% 0.1% formic acid in water/99.9% 0.1% formic acid in acetonitrile; 1.91 min: 0.8 mL/min, 95% 0.1% formic acid in water/5% 0.1% formic acid in acetonitrile. 8 min method, 0 min: 0.8 mL/min, 95% 0.1% formic acid in water/5% 0.1% formic acid in acetonitrile; 7.5 min: 0.8 mL/min, 0.1% 0.1% formic acid in water/99.9% 0.1% formic acid in acetonitrile; 7.91 min: 0.8 mL/min, 95% 0.1% formic acid in water/5% 0.1% formic acid in acetonitrile.

Flash chromatography was performed on silica gel (230 – 400 Mesh, Grade 60) under a positive pressure of Nitrogen. Thin Layer Chromatography was performed on 25 µm TLC Silica gel 60 F254 glass plates purchased from Fisher Scientific (part number: S07876). Visualization was performed using ultraviolet light (254 and 365 nm) and/or potassium permanganate (KMnO4) stain. Reverse-phase prep-HPLC was performed on a Teledyne ISCO CombiFlash® EZ Prep (RediSep Prep C18, 100 Å, 5 µm, 150 mm × 20 mm (part no. 692203810) using 0.1% formic acid in water and 0.1% formic acid in acetonitrile eluent.

General Procedures

General Procedure A: Deaminative Esterification

In a nitrogen filled glovebox, a dry two-dram vial equipped with a stir bar was charged with potassium iodide (KI) (1.5 equiv.), triphenylpyrylium tetrafluoro borate (TPP^+) (1.0 equiv.), powdered 4 Å mol sieves (500 mg/ mmol amine), potassium *tert*-butoxide (1.0-2.0 equiv.), the carboxylic acid (1.0 equiv.) and the amine (1.0 equiv.). 1,4-dioxane (3.33 mL/ mmol amine) was added. If either the amine or the acid was a liquid, they were added to the reaction after the addition of solvent. After all reagents were added, the vial was capped, removed from the glovebox, and stirred at 500 rpm at 80-110 °C for 22 hours. Upon completion, the reaction was diluted with ethyl acetate (EtOAc) (25 mL/mmol) and filtered through a pad of celite. The solvent was removed *in vacuo*. Purification was achieved as described.

General Procedure B: Amine–Acid Reductive Etherification

In a nitrogen filled glovebox, a dry two-dram vial (vial **1**) equipped with a stir bar was charged with potassium iodide (1.5 equiv), triphenylpyrylium tetrafluoro borate (1.0 equiv), powdered 4 Å mol sieves (500 mg/ mmol), potassium *tert*-butoxide (1.0-2.0 equiv.), the carboxylic acid (1.0 equiv.) and the amine (1.0 equiv.). 1,4-dioxane (3.33 mL/ mmol amine) was added. If either the amine or the acid was a liquid, they were added to the reaction after the addition of solvent. After all

reagents were added, the vial was capped, removed from the glovebox, and stirred at 500 rpm at 80-110 °C for 22 hours. Upon completion, the reaction vial was cooled to room temperature and returned to the glovebox along with two additional flame dried vials. Dioxane was added (1.67 mL/mmol amine) to vial **1**. Gallium (III) iodide (GaI_3) (3.0 equiv.) was weighed into a second vial (vial **2**) and dioxane (2.50 mL/mmol amine) added. Tris-(pentafluorophenyl)borane (BCF) (0.40 equiv.) was weighed into a third vial (vial **3**) and dioxane (2.50 mL/mmol amine) added. Vials **2** and **3** were capped and vortexed until each reagent had fully dissolved. Vial **2** was added to vial **1** without stirring followed by vial **3**. Trimethoxychlorosilane (2.0 equiv) was added to vial **1** followed by phenylsilane (3.0 equiv.) (**Caution: this will generate a lot of heat and effervescence of hydrogen and methylchloride which are flammable. We encountered no issues up to a 0.35 mmol scale, but there may be hazards at larger scales**). Upon addition of the silane, the vial was capped, and removed from the glovebox. The reaction was stirred at 500 rpm at 65 °C for 90 minutes. Upon cooling to room temperature, the vial was uncapped (**Caution: pressure may have built up in the vial which can cause solvent to overflow**), diluted with (EtOAc) (25 mL/mmol) partitioned between additional EtOAc (100 mL/mmol) and saturated aqueous sodium bicarbonate (100 mL/mmol). The aqueous layer was extracted twice with EtOAc (100 mL/mmol). The organic layers were combined, dried over sodium sulfate and the solvent removed *in vacuo*. Purification was achieved as described.

General Procedure B: Amine–Acid Reductive Etherification (no glovebox):

In air, an oven dried 2-dram vial (**vial 1**) with a stir bar was added *N*-tosyl isonipecotic acid (56 mg, 0.20 mmol, 1.0 equiv.), triphenylpyrylium tetrafluoroborate (79 mg, 0.20 mmol, 1.0 equiv.), potassium iodide (48 mg, 0.30 mmol, 1.5 equiv.), and potassium tert-butoxide (22 mg, 0.20 mmol, 1.0 equiv). The vial was capped and evaluated and backfilled with nitrogen three times. 0.60 mL of dioxane was added by microliter syringe followed by 2-cyclohexylethylamine (28.0 μL, 0.20 mmol, 1.0 equiv.). This was heated at 110 °C for 22 hours. Upon cooling to room temperature, dioxane was added (1.67 mL/mmol amine) to vial **1**. Gallium (III) iodide (GaI_3) (270.2 mg, 3.0 equiv.) was weighed into a second vial (vial **2**), sealed with PTFE line cap, and evacuated and backfilled three times with N_2. Dioxane (2.50 mL/mmol amine) added. Tris-(pentafluorophenyl)borane (BCF) (41.0 mg, 0.40 equiv.) was weighed into a third vial (vial **3**), sealed with PTFE line cap, and evacuated and backfilled three times with N_2. Dioxane (2.50 mL/mmol amine) added. Vials **2** and **3** were vortexed until each reagent had fully dissolved. Vial **2** was added to vial **1** without stirring followed by vial **3**. Trimethoxychlorosilane (57.6 μL,2.0 equiv.) was added to vial **1** followed by phenylsilane (59.1μL, 3.0 equiv.) (Caution: this will generate a lot of heat and effervescence of hydrogen and methylchloride which are flammable. We encountered no issues up to a 0.35 mmol scale, but there may be hazards at larger scales). The reaction was stirred at 500 rpm at 65 °C for 90 minutes. Upon cooling to room temperature, the vial was uncapped (Caution: pressure may have built up in the vial which can cause solvent to overflow), diluted with (EtOAc) (25 mL/mmol) partitioned between additional EtOAc (100 mL/mmol) and saturated aqueous sodium bicarbonate (100 mL/mmol). The aqueous layer was extracted twice with EtOAc (100 mL/mmol). The organic layers were combined, dried over sodium sulfate and the solvent removed in vacuo. Isolated in 49% yield (37.2 mg) as brown solid after purification by column chromatography (10-30% ethyl acetate in hexane).

General Procedure C: Reduction of Purified Esters to Ethers

Three dried two-dram vials were brought into the glovebox. In Vial **1** equipped with a stir bar, corresponding ester (1.0 equiv.) was added. Dioxane was added (3.33 mL/mmol ester) to vial **1**.

GaI_3 (0.5 equiv.) was weighed into a second vial (vial **2**) and dioxane (3.33 mL/mmol ester) added. Tris-(pentafluorophenyl)borane or trimesityl borane (0.25 equiv.) was weighed into a third vial (vial **3**) and dioxane (3.33 mL/mmol ester) added. Vials **2** and **3** were capped and vortexed until each reagent had fully dissolved. Vial **2** was added to vial **1** without stirring followed by vial **3**. Trimethoxychlorosilane (2.0 equiv.) was added to vial **1** followed by diphenylsilane (2.0 equiv.) (**Caution: this will generate heat and effervescence of hydrogen and methyl chloride which are flammable.**). Upon addition of the silane, the vial was capped, and removed from the glovebox. The reaction was stirred at 500 rpm at 65 °C for 90 minutes. Upon cooling to room temperature, the vial was uncapped (**Caution: pressure may have built up in the vial which can cause solvent to overflow**), diluted with (EtOAc) (25 mL/mmol) partitioned between additional EtOAc (100 mL/mmol) and saturated aqueous sodium bicarbonate (100 mL/mmol). The aqueous layer was extracted twice with EtOAc (100 mL/mmol). The organic layers were combined, dried over sodium sulfate and the solvent removed *in vacuo*. Purification was achieved as described.

General Procedure D: Preparation of Ethers from Phenols and Amines

An oven dried two-dram vial equipped with a stir bar was added triphenylpyrylium tetrafluoro borate TPP^+ (1.0 equiv.). The appropriate metal halide salt (KI, KBr, or LiCl) (3.0 equiv.) was then added followed by the amine (1.0 equiv.) if it was solid and potassium *tert*-butoxide (1.0 equiv.) if the amine was a salt. The vial was then capped with a septa cap and evacuated/backfilled with nitrogen 3 times. Dioxane (3.33 mL/ mmol amine) was added via syringe. If the amine was a liquid, it was also added via syringe at this point. The vial was transferred to a hotplate, stirred at 500 rpm at 110 °C for 22 hours. The reaction was cooled to room temperature and transferred via syringe to a vial containing the desired phenol (1.0 equiv.), potassium carbonate (3.0 equiv.), and DMA (3.33 mL/mmol amine). This was stirred at 500 rpm at 80 °C for 4 hours. Upon completion, the reaction was diluted with ethyl acetate (EtOAc) (25 mL/mmol) partitioned between additional EtOAc (100 mL/mmol) and saturated aqueous sulfate (100 mL/mmol). The aqueous layer was extracted twice with EtOAc (100 mL/mmol). The organic layers were combined, dried over sodium sulfate and the solvent removed *in vacuo.* Purification was achieved as described.

General Procedure E: Preparation of Alkyl Halides from Amines

An oven dried two-dram vial equipped with a stir bar was added triphenylpyrylium tetrafluoro borate TPP^+ (1.0 equiv.). The appropriate metal halide salt (KI, KBr, or LiCl) (3.0 equiv.) was then added followed by the amine (1.0 equiv.) if it was solid and potassium *tert*-butoxide (1.0 equiv.) if the amine was a salt. The vial was then capped with a septa cap and evacuated/backfilled with nitrogen 3 times. Dioxane (3.33 mL/ mmol amine) or DMA (10.0 mL/mmol amine) was added via syringe. If the amine was a liquid, it was also added via syringe at this point. The vial was transferred to a hotplate, stirred at 500 rpm at 110 °C for 22 hours. For reactions in dioxane upon completion, the reaction was diluted with ethyl acetate (EtOAc) (25 mL/mmol) and filtered through a pad of celite. The solvent was removed *in vacuo*. For reactions in DMA, upon completion, the reaction was diluted with ethyl acetate (EtOAc) (25 mL/mmol) partitioned between additional EtOAc (100 mL/mmol) and saturated aqueous sulfate (100 mL/mmol). The aqueous layer was extracted twice with EtOAc (100 mL/mmol). The organic layers were combined, dried over sodium sulfate and the solvent removed *in vacuo.* After column purification, we isolated desired alkyl halides.

General Procedure F: Platinum Catalyzed Ether Synthesis from Pyridinium Salts and Carboxylic Acids

An oven dried two-dram vial equipped with a stir bar was added pyridinium salt of corresponding amine (1.0 equiv.). KI (1.0 equiv.) was then added followed by the carboxylic acid (1.0 equiv.), and proton sponge (1.0 equiv.) The vial was then capped with a septa cap and evacuated/backfilled with nitrogen 3 times. Chlorobenzene (3.33 mL/ mmol acid) was added via syringe followed by phenylsilane (3.0 equiv.). The vial was transferred to a hotplate, stirred at 500 rpm at 80 °C for benzylic pyridiniums or 110 °C for primary pyridiniums for 22 hours. Upon cooling to room temperature, the vial was brought into the glovebox and platinum (II) chloride (2.5 mol%) was added followed by phenylsilane (3.0 equiv). The vial was removed from the glovebox and stirred at 500 rpm at 80 °C for two hours. Upon completion the reaction was diluted with (EtOAc) (25 mL/mmol) partitioned between additional EtOAc (100 mL/mmol) and saturated aqueous sodium bicarbonate (100 mL/mmol). The aqueous layer was extracted twice more with EtOAc (100 mL/mmol). The organic layers were combined, dried over sodium sulfate and the solvent removed *in vacuo*. Purification was achieved as described.

General Procedure G: Preparation of Pyridinium Salts from Corresponding Amines

Following a reported procedure[1] A dry round-bottom flask equipped with a stir bar was charged with triphenylpyrylium tetrafluoroborate (1.0 equiv.) and ethanol (1.0 mL/ mmol). Amine (1.2 equiv.) was then added with stirring. This was stirred at 500 rpm at 80 °C for 4 hours. Upon completion the reaction was allowed to cool to room temperature and poured into diethyl ether (Et_2O) (5 mL/mmol) and stirred at 1200 rpm for 1 hour. The desired product was collected by filtration and washed with Et_2O (10 mL/ mmol).

General Procedure H: Procedure for High-Throughput Experimentation in 24- and 96-Well Plates

Stock solutions, or suspensions, were prepared as shown in the heatmap preparation table. In an inert atmosphere glovebox, reagents were weighed and dissolved or suspended in anhydrous solvent to achieve their listed concentrations in the table. Stock solutions of reagents were stirred until either a clear solution or a uniform slurry was achieved. A 24- or 96-well aluminum microvial plate (Analytical Sales & Services cat. no. 25243) was equipped with oven-dried shell vials (Analytical Sales & Services cat. no. 884001) and then moved into the glovebox. Stock solutions were dosed to the appropriate shell vials according to the plate map shown in table using single channel micropipettes. A parylene-coated stir dowel (Analytical Sales & Services cat. no. 13258) was then added to each vial. The micro vial plate was sealed, removed from the glove box, and stirred on a tumble stirrer with heating to indicated temperature for planned reaction time in a heating block.

After that, from each reaction, a 30 µL aliquot of the quenched reaction mixture was added into a 96-well polypropylene collection plate (Analytical Sales & Services cat. no. 17P687). A solution of caffeine in MeCN [0.1 (M)] 30 µL as internal standard was added, followed by pure MeCN (300 µL) and mixed by pipetting up and down. The reactions were then analyzed by UPLCMS. The assay yields were produced by measuring the UV absorbance of desired product relative to the caffeine internal standard.

Troubleshooting Tips

In several instances of running these reactions when a pyridinium salt is dissolved in dioxane, after stirring with a halide salt, a precipitate forms. It is often a fine yellow or white powder that is presumed to be the pyridinium halide salt that is no longer soluble. When this precipitate forms, the reaction will no longer proceed in dioxane and only starting pyridinium salt is observed. This issue occurred most often with phenethylamines but happens in other cases with no apparent trend. There are two ways to remedy this. If not performing the reductive etherification, adding 10 volume percent dimethylacetamide (DMA) will make the reaction proceed, but will shut down the reductive etherification, requiring purification of the intermediate ester. The other option is to start a new reaction but instead of using 2,4,6-triphenylpyrylium tetrafluoroborate, use 2,4,6-tris(4-(trifluoromethyl) phenyl)pyrylium tetrafluoroborate which is prepared in a single step from the requisite starting materials.[60]

The other issue we encountered was around peroxide content in dioxane bottles. We found that when using diphenylsilane as a reductant dioxane that tested positive for 0.5 mg/mL of peroxide using MQuant® peroxide testing strips (Sigma-Aldrich 1100110002), the desired reductive etherification would not take place. When using phenylsilane as the reductant we found up to 5.0 mg/mL was tolerated.

The following flow chart describes what conditions to use when reducing a purified ester. This is helpful in preparing deuterated derivatives if deuterated phenylsilane is not available as deuterated diphenylsilane is commercially available, or if for any other reason the intermediate ester must be purified. For selective reduction of amides, it is ***highly recommended*** to purify the intermediate ester as any excess silane will begin to reduce the amide carbonyl. We have found if a reaction does not go to completion, adding another equivalent of Gallium and silane will generally accomplish this.

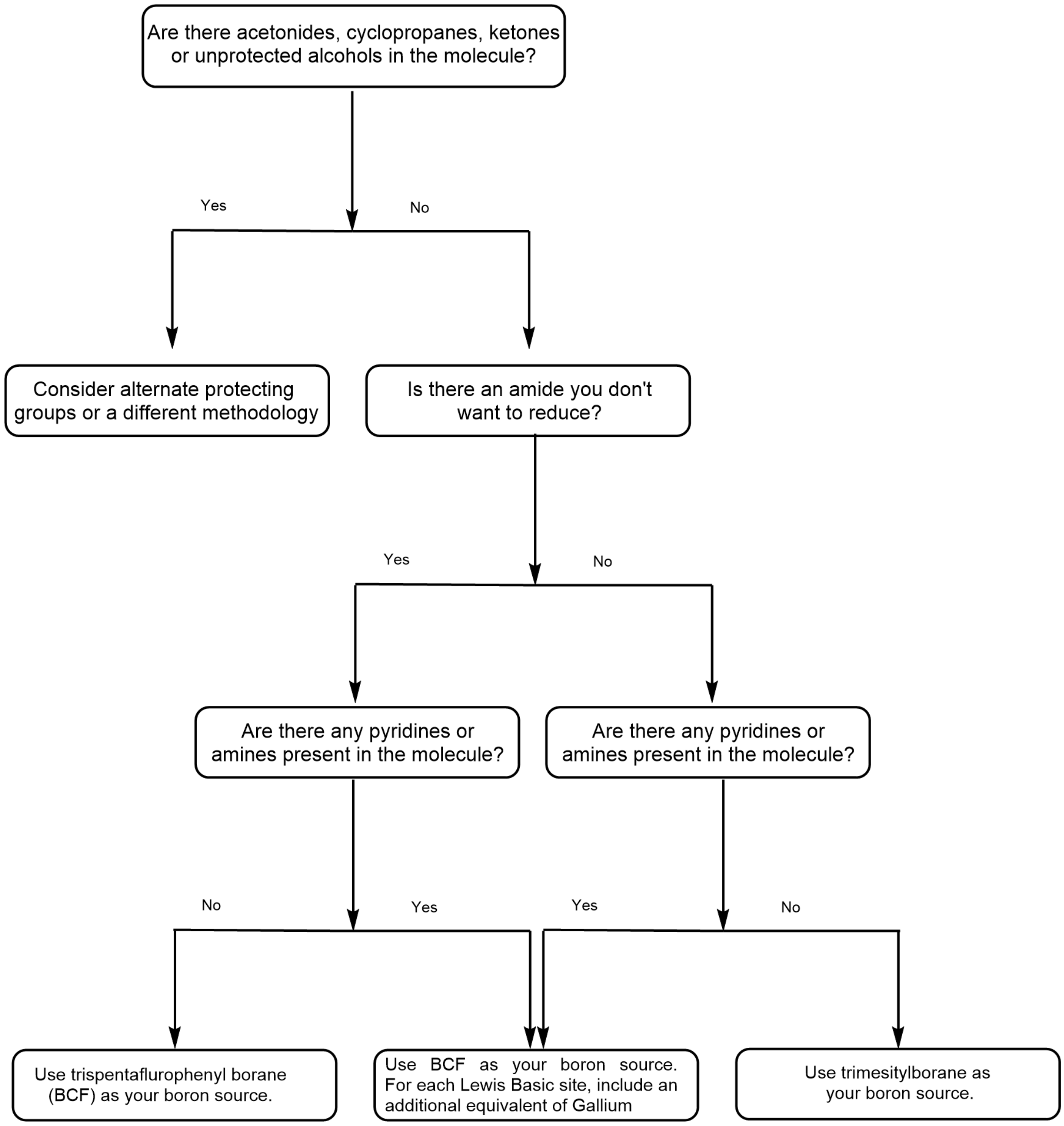

Scheme S1. Flowchart depicting conditions to use based on the substrate being reduced.

Preparation of Starting Materials

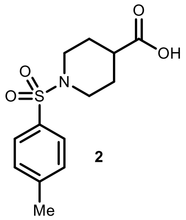

1-tosylpiperidine-4-carboxylic acid (2)

Following a previously reported procedure,[2] a dry 250 mL round bottom flask equipped with a stir bar was charged with piperidine-4-carboxylic acid (6.45 g, 50.0 mmol, 1.0 equiv.) and NaOH (4.00 g, 100 mmol, 2.0 equiv.). 100 mL of a 1:1 mixture of Et_2O and H_2O were added followed by tosyl chloride (9.55 g, 50.0 mmol, 1.0 equiv.). The clear mixture was stirred at 500 RPM at rt for 18 h. A white cloudy precipitate was formed, and in a separatory funnel, the mixture was diluted with 100 mL of Et_2O, and H_2O added until the precipitate was completely dissolved. The two layers were separated, and the pH of the aqueous layer was adjusted to 3 by addition of 3(M) HCl. The precipitate was filtered and reconstituted in EtOAc. solvent removed *in vacuo* to give **2** (8.6 g, 60%) as a white solid.

The NMR spectra match those reported in the literature.

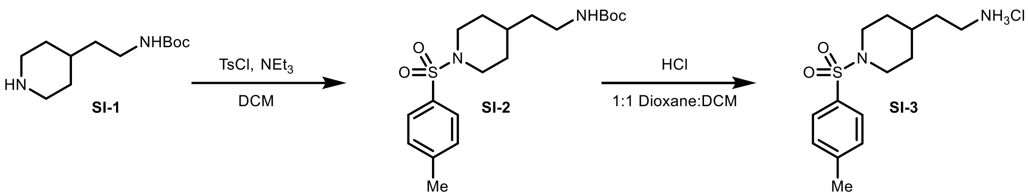

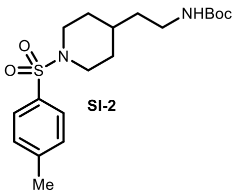

tert-butyl (2-(1-tosylpiperidin-4-yl)ethyl)carbamate (SI-2)

Following a literature procedure[3], a dry two-dram vial equipped with a stir bar was charged with tert-butyl (2-(piperidin-4-yl)ethyl)carbamate (1.00 g, 4.38 mmol, 1.0 equiv.). The vial was evacuated and refilled with nitrogen 3x. 2.75 mL of dichloromethane (DCM) was added followed by triethylamine (1.22 mL,8.76 mmol, 2.0 equiv.). This was stirred until all solids dissolved followed by a dropwise addition of tosyl chloride (835 mg, 4.38 mmol, 1.0 equiv.) in 1.75 mL of DCM containing 10 µL of TEA. This mixture was stirred for 14 hours at room temperature. Upon completion, the reaction was diluted with (DCM) (5.0 mL) partitioned between additional DCM (75 mL) and 1M aqueous hydrochloric acid (HCl) (30 mL). The layers were separated, and the organic layer washed with aqueous sodium bicarbonate (30 mL). The organic layer was separated, dried

over sodium sulfate and the solvent removed *in vacuo* to give **SI-2** (1.59 g, 95%) as a white solid. This product was used without further purification.

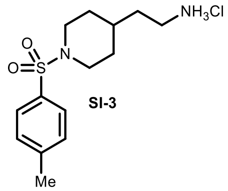

SI-3

2-(1-tosylpiperidin-4-yl)ethan-1-amine (SI-3)

A dry 50 mL round bottom flask equipped with a stir bar was charged with **SI-2** (1.59 g, 4.16 mmol, 1.0 equiv). Under air, 10 mL of DCM was added, and the solution stirred until it completely dissolved. 4M HCl in dioxane (10.4 mL, 1.52 g, 41.6 mmol, 10.0 equiv.) was added dropwise to the solution. This was stirred for 2 hours at room temperature and 300 rpm. The cloudy white solution was diluted with 30 mL of Et_2O and filtered. The filtrate was washed with another 25 mL of ether and dried under high-vacuum overnight to give **SI-3** (1273 mg, 96%) as a white solid.

^{1}H NMR (499 MHz, DMSO-d_6) δ 7.90 (s, 2H), 7.61 (d, *J* = 8.2 Hz, 2H), 7.45 (d, *J* = 8.0 Hz, 2H), 3.59 (dt, *J* = 11.6, 3.5 Hz, 2H), 2.73 (h, *J* = 5.8 Hz, 2H), 2.41 (s, 3H), 2.13 (td, *J* = 11.9, 2.5 Hz, 2H), 1.69 (d, *J* = 10.2 Hz, 2H), 1.44 (q, *J* = 7.3 Hz, 2H), 1.29 (dtq, *J* = 14.3, 7.1, 3.5 Hz, 1H), 1.14 (qd, *J* = 12.1, 4.0 Hz, 2H).

^{13}C NMR (126 MHz, DMSO-d_6) δ 143.47, 132.37, 129.78, 127.49, 45.93, 36.30, 32.92, 31.40, 30.49, 20.99.

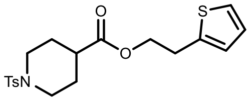

SI-4

2-(thiophen-2-yl)ethyl 1-tosylpiperidine-4-carboxylate (SI-4)

Compound **SI-4** was prepared on a 0.2 mmol scale from **1** and 2-(2-thiophenyl)-ethylamine according to a modified general procedure **A** where **11** was replaced with 2,4,6-tris(4-(trifluoromethyl) phenyl) pyrylium tetrafluoroborate to give 20.0 mg (26%) of a yellow oil after purification with ethyl acetate & hexanes. R_f = 0.24 in 20% EA in hexanes

^{1}H NMR (400 MHz, $CDCl_3$) δ 7.63 (d, *J* = 8.2 Hz, 2H), 7.32 (d, *J* = 8.0 Hz, 2H), 7.14 (dd, *J* = 5.1, 1.2 Hz, 1H), 6.92 (dd, *J* = 5.1, 3.4 Hz, 1H), 6.81 (dd, *J* = 3.4, 1.2 Hz, 1H), 4.27 (t, *J* = 6.5 Hz, 2H), 3.59 (dt, *J* = 12.2, 4.1 Hz, 2H), 3.11 (t, *J* = 6.5 Hz, 2H), 2.49 – 2.38 (m, 5H), 2.25 (tt, *J* = 10.6, 4.0 Hz, 1H), 1.94 (dt, *J* = 12.0, 3.9 Hz, 2H), 1.79 (dtd, *J* = 14.3, 10.7, 4.0 Hz, 2H).

^{13}C NMR (101 MHz, $CDCl_3$) δ 173.8, 143.7, 139.9, 133.1, 129.8, 127.8, 127.0, 125.7, 124.2, 64.9, 45.5, 40.1, 29.3, 27.5, 21.7, 21.6.

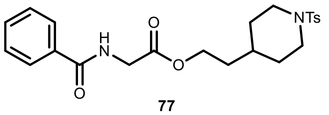

77

2-(1-tosylpiperidin-4-yl)ethyl benzoylglycinate (77)

Compound **77** was prepared from on a 0.2 mmol scale hippuric acid and **SI-3** via a modified general procedure **A** where 2 equivalents of base were used to give 64.8 mg (73%) of a white solid after purification with ethyl acetate.

R_f = 100% EA: 0.25

^{1}H NMR (400 MHz, $CDCl_3$) δ 7.82 – 7.75 (m, 2H), 7.67 – 7.59 (m, 2H), 7.56 – 7.47 (m, 1H), 7.48 – 7.39 (m, 2H), 7.32 (d, *J* = 7.7 Hz, 2H), 6.61 (s, 1H), 4.20 (dd, *J* = 7.5, 5.8 Hz, 4H), 3.76 (d, *J* = 11.4 Hz, 2H), 2.43 (s, 3H), 2.25 – 2.15 (m, 2H), 1.74 (d, *J* = 9.8 Hz, 2H), 1.60 (q, *J* = 6.2 Hz, 2H), 1.33 (s, 2H).

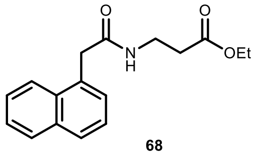

68

To an oven dried 50 mL round bottom flask equipped with a stir bar was added potassium 2-(naphthalen-1-yl)acetate (1.00 g, 4.50 mmol, 1.0 equiv.) followed by 15 mL of DCM and 1.00 mL of dimethylformamide. Thionyl chloride (363 µL ,595 mg, 5.00 mmol, 1.1 equiv. was added) followed by stirring at 35 °C for one hour. During this time a 2-dram vial was charged with beta alanine ethyl ester hydrochloride salt (830 mg, 5.4 mmol, 1.2 equiv.). This was suspended in 5.0 mL of DCM and DIPEA (1.34 g, 1.80 mL, 10.8 mmol, 2.4 equiv.). After one hour, the beta alanine solution was transferred to the acid chloride solution via syringe. This was allowed to stir for 20 additional minutes at 35 °C. Upon completion, the reaction was cooled to room temperature, diluted with an additional 30 mL of DCM, and washed with 50 mL of aqueous 1M HCl followed by 50 mL of saturated sodium carbonated. The organic layer was dried over sodium sulfate and concentrated *in vacuo*. The resulting residue was purified via column chromatography with ethyl acetate and hexanes to give 1.0 g (83%) of **68** as a white solid.

^{1}H NMR (499 MHz, dioxane-d_8) δ 8.03 (d, *J* = 8.3 Hz, 1H), 7.88 (dd, *J* = 7.6, 1.8 Hz, 1H), 7.80 (d, *J* = 8.0 Hz, 1H), 7.50 (pd, *J* = 6.8, 1.5 Hz, 2H), 7.42 (dt, *J* = 15.2, 7.1 Hz, 2H), 6.55 (t, *J* = 6.2 Hz, 1H), 3.96 (q, *J* = 7.1 Hz, 2H), 3.90 (s, 2H), 3.33 (q, *J* = 6.3 Hz, 2H), 2.40 (t, *J* = 6.3 Hz, 2H), 1.13 (t, *J* = 7.1 Hz, 3H).

^{13}C NMR (126 MHz, dioxane-d_8) δ 171.62, 169.76, 133.94, 132.48, 132.37, 128.42, 127.63, 127.51, 126.08, 125.63, 125.39, 124.11, 59.76, 40.75, 35.01, 33.64, 13.57.

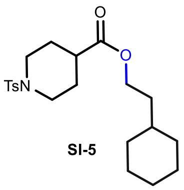

SI-5

SI-5 was prepared on a 0.20 mmol scale according to general procedure **A** from **2** (56.7 mg, 0.20 mmol, 1.0 equiv.) and 2-cyclohexylethan-1-amine (25.4 mg, 1.0 equiv.). Isolated in 67% yield (53 mg) as brown solid after purification by column chromatography (10-30% ethyl acetate in hexane).

^{1}H NMR (600 MHz, $CDCl_3$) δ 7.64 (d, *J* = 7.8 Hz, 2H), 7.32 (d, *J* = 7.8 Hz, 2H), 4.08 (t, *J* = 7.2 Hz, 2H), 3.62-3.59 (m, 2H), 2.47 (td, *J* = 12.0, 3.0 Hz, 2H), 2.43 (s, 3H), 2.26-2.21 (m, 1H), 1.98 -1.94 (m, 2H), 1.84 – 1.78 (m, 2H), 1.70 -1.63 (m, 5H), 1.47 (q, *J* = 6.6 Hz, 2H), 1.33 – 1.27 (m, 1H), 1.24 -1.09 (m, 4H), 0.93 -0.87 (m, 2H).

^{13}C NMR (150 MHz, $CDCl_3$) δ 173.9, 143.5, 133.1, 129.6, 127.7, 63.0, 45.4, 40.1, 35.9, 34.6, 33.1, 27.4, 26.2, 21.5.

HRMS (ESI) Calculated $C_{21}H_{32}NO_4S[M+H]^+$: 394.2052, Found 394.2045.

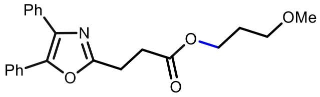

SI-6

SI-6 was prepared on a 0.20 mmol scale according to general procedure **A** from Oxaprozin (58.7 mg, 0.20 mmol, 1.0 equiv.) and 3-Methoxypropylamine (20.3 µL, 1.0 equiv.). Isolated in 59% yield (47.5 mg) as brown solid after purification by column chromatography (10-35% ethyl acetate in hexane).

^{1}H NMR (600 MHz, $CDCl_3$) δ 7.64-7.62 (m, 2H), 7.58-7.56 (m, 2H), 7.38 – 7.30 (m, 6H), 4.22 (t, *J* = 6.6 Hz, 2H), 3.42 (t, *J* = 6.0 Hz, 2H), 3.29 (s, 3H), 3.18 (t, *J* = 7.2 Hz, 2H), 2.92 (t, *J* = 7.2 Hz, 2H), 1.90 (p, *J* = 6.0 Hz, 2H).

^{13}C NMR (150 MHz, $CDCl_3$) δ 171.9, 161.7, 145.4, 135.1, 132.5, 129.0, 128.6, 128.5, 128.4, 128.0, 127.9, 126.5, 69.0, 62.0, 58.7, 31.2, 29.0, 23.6.

HRMS (ESI) Calculated $C_{22}H_{24}NO_4$ $[M+H]^+$: 366.1705, Found 366.1695.

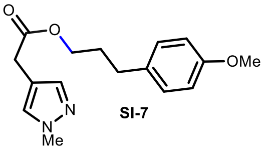

SI-7

SI-7 was prepared on a 0.20 mmol scale according to general procedure **A** from the 2-(1-methyl-1H-pyrazol-4-yl)acetic acid (28.0 mg, 0.20 mmol, 1.0 equiv.) and 3-(4-methoxyphenyl)propan-1-amine (33.0 mg, 1.0 equiv.). Isolated in 55% yield (31.7 mg) as brown solid after purification by column chromatography (10-35% ethyl acetate in hexane).

^{1}H NMR (600 MHz, $CDCl_3$) δ 7.41 (s, 1H), 7.34 (s, 1H), 7.26 (s, 1H), 7.07 (d, J = 9.0 Hz, 2H), 6.84-6.81 (m, 2H), 4.11 (t, J = 6.6 Hz, 2H), 3.87 (s, 3H), 3.79 (s, 3H), 3.48 (s, 2H), 2.61 (t, J = 7.8 Hz 2H), 1.95–1.90 (m, 2H).

^{13}C NMR (150 MHz, $CDCl_3$) δ 171.6, 157.9, 139.3, 133.1, 129.4, 129.3, 113.8, 113.3, 64.2, 55.3, 38.9, 31.2, 30.4, 30.3.

HRMS (ESI) Calculated $C_{16}H_{21}N_2O_3$ $[M+H]^+$: 289.1552, Found 289.1448.

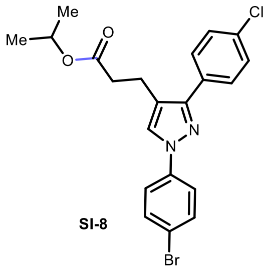

SI-8

SI-8 was prepared on a 0.20 mmol scale according to general procedure **A** from 3-(1-(4-bromophenyl)-3-(4-chlorophenyl)-1H-pyrazol-4-yl)propanoic acid (81.1 mg, 0.20 mmol, 1.0 equiv.) and isopropyl amine (17.1 µL, 1.0 equiv.). Isolated in 57% yield (46.2 mg) as white solid after purification by column chromatography (10-35% ethyl acetate in hexane).

^{1}H NMR (600 MHz, $CDCl_3$) δ 7.79 (s, 1H), 7.67 – 7.65 (m, 2H), 7.61 – 7.59 (m, 2H), 7.57 – 7.55 (m, 2H), 7.44 – 7.41 (m, 2H), 5.04 – 4.98 (m, 1H), 3.00 (t, J = 7.2 Hz, 2H), 2.59 (t, J = 7.8 Hz, 2H), 1.21 (d, J = 6.0 Hz, 6H).

^{13}C NMR (150 MHz, $CDCl_3$) δ 172.1, 150.6, 138.9, 134.0, 132.4, 131.8, 129.1, 128.8, 126.4, 120.3, 120.1, 119.4, 68.0, 34.9, 21.8, 20.1.

HRMS (ESI) Calculated $C_{21}H_{21}BrClN_2O_2$ $[M+H]^+$: 447.0475, Found 447.0470.

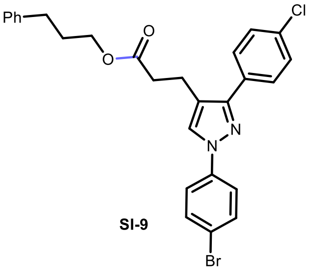

SI-9

SI-9 was prepared on a 0.20 mmol scale according to general procedure **A** from 3-(1-(4-bromophenyl)-3-(4-chlorophenyl)-1H-pyrazol-4-yl)propanoic acid (81.1 mg, 0.20 mmol, 1.0 equiv.) and 3-Phenyl-1-propylamine (28.0 µL, 1.0 equiv.). Isolated in 61% yield (64.0 mg) as brown solid after purification by column chromatography (10-35% ethyl acetate in hexane).

^{1}H NMR (600 MHz, $CDCl_3$) δ 7.80 (s, 1H), 7.66 (d, J = 8.4 Hz, 2H), 7.59 (d, J = 8.6 Hz, 2H), 7.55 (d, J = 9.0 Hz, 2H), 7.43 (d, J = 8.6 Hz, 2H), 7.27 – 7.25 (m, 3H), 7.19-7.17 (m, 1H), 7.12 (d, J = 7.2 Hz, 2H), 4.10 (t, J = 6.6 Hz, 2H), 3.02 (t, J = 7.2Hz, 2H), 2.64-2.61 (m, 4H), 1.95-1.90 (m, 2H).

^{13}C NMR (150 MHz, $CDCl_3$) δ 171.6, 149.6, 140.0, 137.9, 133.0, 131.4, 130.8, 128.0, 127.8, 127.4,127.3, 125.4, 125.0, 119.2,119.1 118.4, 63.0, 33.6, 32.6, 31.1, 29.1, 19.1.

HRMS (ESI) Calculated $C_{27}H_{25}BrClN_2O_2$ $[M+H]^+$: 523.0788, Found 523.0780.

HTE Optimization of Deaminative Reductive Etherification

Screen examining 3 Lewis acids (0.3 equiv.), 2 boranes, and 2 silanes

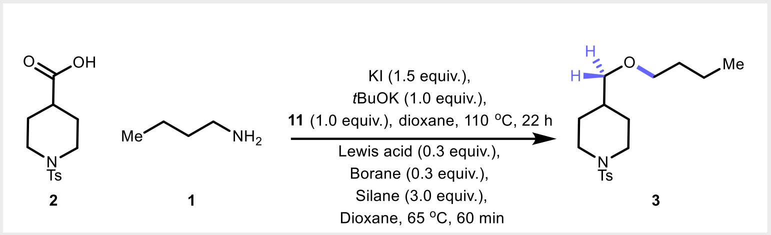

name	molarity	color
Gallium Bromide	0.1	#48039f
Aluminum Chloride	0.1	#fdb130
Indium (III) Bromide	0.1	#44039e
DODECACARBONYLTRIIRON	0.03	#280592
TRIS(PENTAFLUOROPHENYL)BORANE	0.03	#b02991
triphenylborane	0.03	#e87059
Catalyst 2 blank	0.03	#ce4b75
diphenylsilane	0.3	#7801a8
tetramethyldisiloxane	0.3	#e26660
Ester SM	0.1	#fada24

Reagents	Solvent	C_{stock} (M)	V_{dose} (μL)	Wells	Order Added
Gallium (III) Bromide	Dioxane	0.9	25	A, 1-6	1
Aluminium Chloride	Dioxane	0.9	25	B, 1-6	1
Indium (III) Bromide	Dioxane	0.9	25	C, 1-6	1
Dodecacarbonyltriiron	Dioxane	0.9	25	D, 1-6	1
trispentafluorophenyl borane	Dioxane	0.12	25	A,B,C,D, 1,4	2
triphenylborane	Dioxane	0.12	25	A,B,C,D, 2,5	2
Borane blank	Dioxane	0.12	25	A,B,C,D, 3,6	2
**5 ester solution**	Dioxane	0.3	25	All	3
diphenylsilane	Dioxane	0.9	25	A,B,C,D, 1-3	4
tetramethyldisiloxane	Dioxane	0.9	25	A,B,C,D, 4-6	4

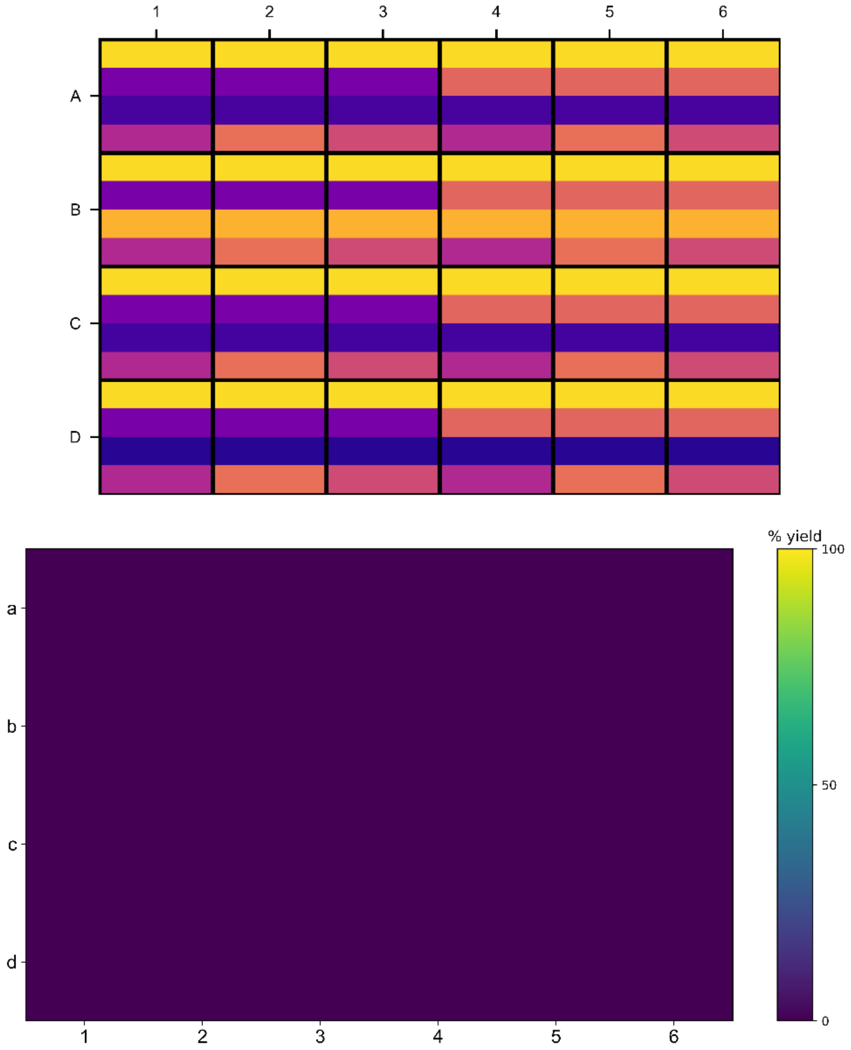

Figure S1. Results of screen examining 3 Lewis acids (0.3 equiv.), 2 boranes, and 2 silanes.

Screen examining 3 Lewis acids (3.0 equiv.), 2 boranes, and 2 silanes

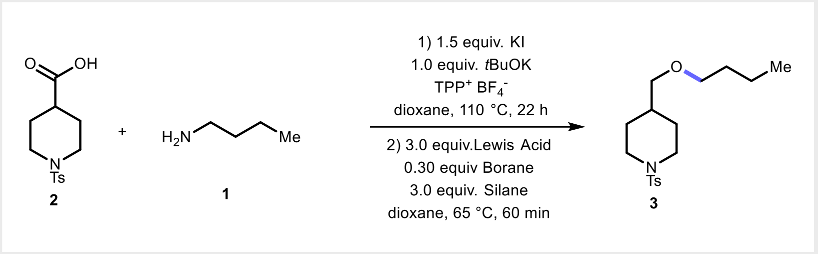

name	molarity	color
Gallium Bromide	0.3	#48039f
Aluminum Chloride	0.3	#fdb130
Indium (III) Bromide	0.3	#44039e
DODECACARBONYLTRIIRON	0.1	#280592
TRIS(PENTAFLUOROPHENYL)BORANE	0.03	#b02991
triphenylborane	0.03	#e87059
Catalyst 2 blank	0.03	#ce4b75
diphenylsilane	0.3	#7801a8
tetramethyldisiloxane	0.3	#e26660
Ester SM	0.1	#fada24

Reagents	Solvent	C_{stock} (M)	V_{dose} (μL)	Wells	Order Added
Gallium (III) Bromide	Dioxane	0.9	25	A, 1-6	1
Aluminium Chloride	Dioxane	0.9	25	B, 1-6	1
Indium (III) Bromide	Dioxane	0.9	25	C, 1-6	1
Dodecacarbonyltriiron	Dioxane	0.9	25	D, 1-6	1
trispentafluorophenyl borane	Dioxane	0.12	25	A,B,C,D, 1,4	2
triphenylborane	Dioxane	0.12	25	A,B,C,D, 2,5	2
Borane blank	Dioxane	0.12	25	A,B,C,D, 3,6	2
**5 ester solution**	Dioxane	0.3	25	All	3
diphenylsilane	Dioxane	0.9	25	A,B,C,D, 1-3	4
tetramethyldisiloxane	Dioxane	0.9	25	A,B,C,D, 4-6	4

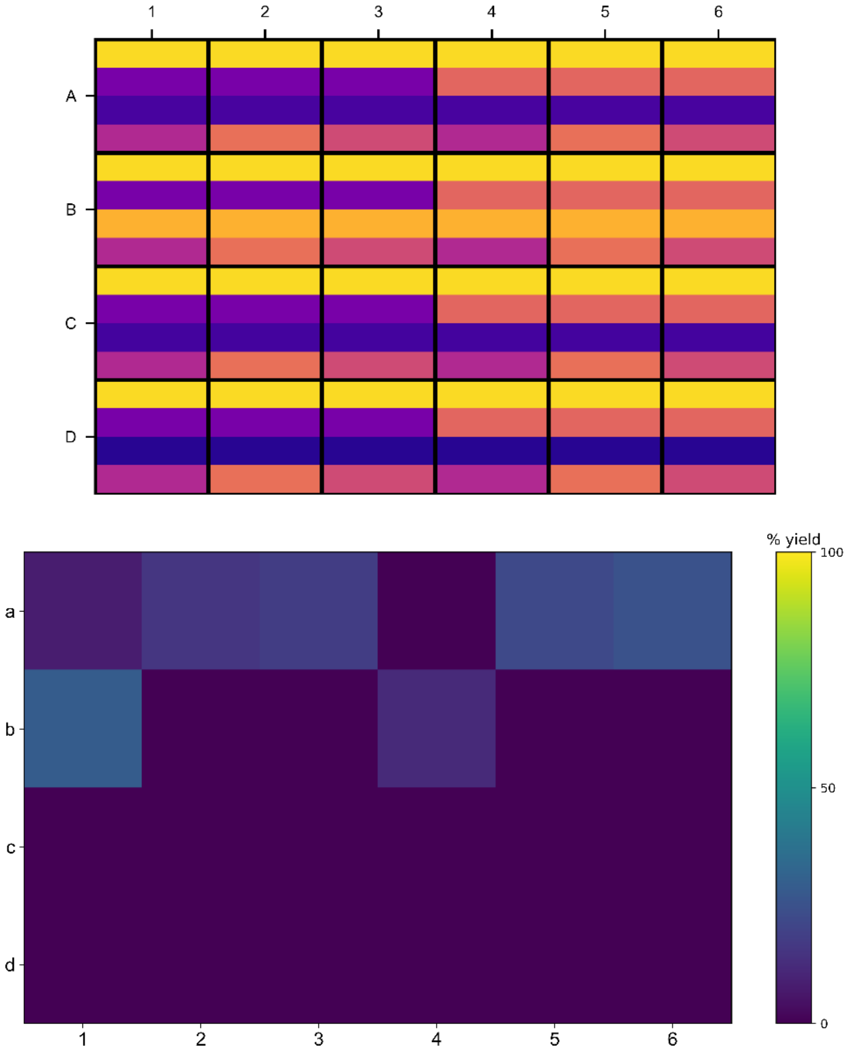

Figure S2. Results of screen examining 3 Lewis acids (3.0 equiv), 2 boranes, and 2 silanes.

96 well screen examining 6 Lewis acids, 4 boranes, and 4 silanes

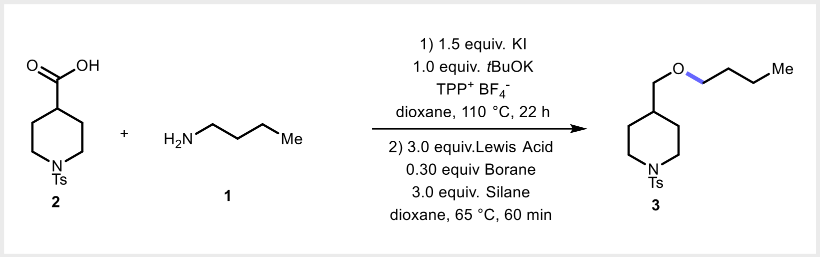

name	molarity	color
Aluminum Chloride	0.3	#febe2a
Aluminum bromide	0.3	#f79342
Aluminum iodide	0.3	#cc4977
Gallium(III) chloride	0.3	#46039f
Gallium(III) bromide	0.3	#48039f
Gallium(III) Iodide	0.3	#4903a0
Diphenylsilane	0.3	#e26660
Phenylsilane	0.3	#f99a3e
1,1,3,3-Tetramethyldisiloxane	0.3	#f3f027
Triethylsilane	0.3	#da5b69
TRIS(PENTAFLUOROPHENYL)BORANE	0.03	#fbd724
Triphenylborane	0.03	#9e199d
Catalyst blank	0.1	#cb4679
Boron trifluoride etherate, ca. 48% BF3	0.06	#ee7b51
Ester SM	0.1	#a72197

Reagents	Solvent	C_{stock} (M)	V_{dose} (μL)	Wells	Order Added
Aluminum Chloride	Dioxane	0.9	25	A-H, 1,7	1
Aluminum Bromide	Dioxane	0.9	25	A-H, 2,8	1
Aluminum Iodide	Dioxane	0.9	25	A-H, 3,9	1
Gallium (III) Chloride	Dioxane	0.9	25	A-H, 4,10	1
Gallium (III) Bromide	Dioxane	0.9	25	A-H, 5,11	1
Gallium (III) Iodide	Dioxane	0.9	25	A-H, 6,12	1
trispentafluorophenyl borane	Dioxane	0.12	25	A-D, 1-6	2
triphenylborane	Dioxane	0.12	25	A-D, 7-12	2
Borane blank	Dioxane	0.12	25	E-H, 1-6	2
BF3 etherate	Dioxane	0.12	25	E-H, 7-12	2
**5 ester solution**	Dioxane	0.3	25	All	3
phenylsilane	Dioxane	0.9	25	A,E 1-12	4
diphenylsilane	Dioxane	0.9	25	B, F 1-12	4
tetramethyldisiloxane	Dioxane	0.9	25	C,G 1-12	4
triethylsilane	Dioxane	0.9	25	D,H 1-12	4

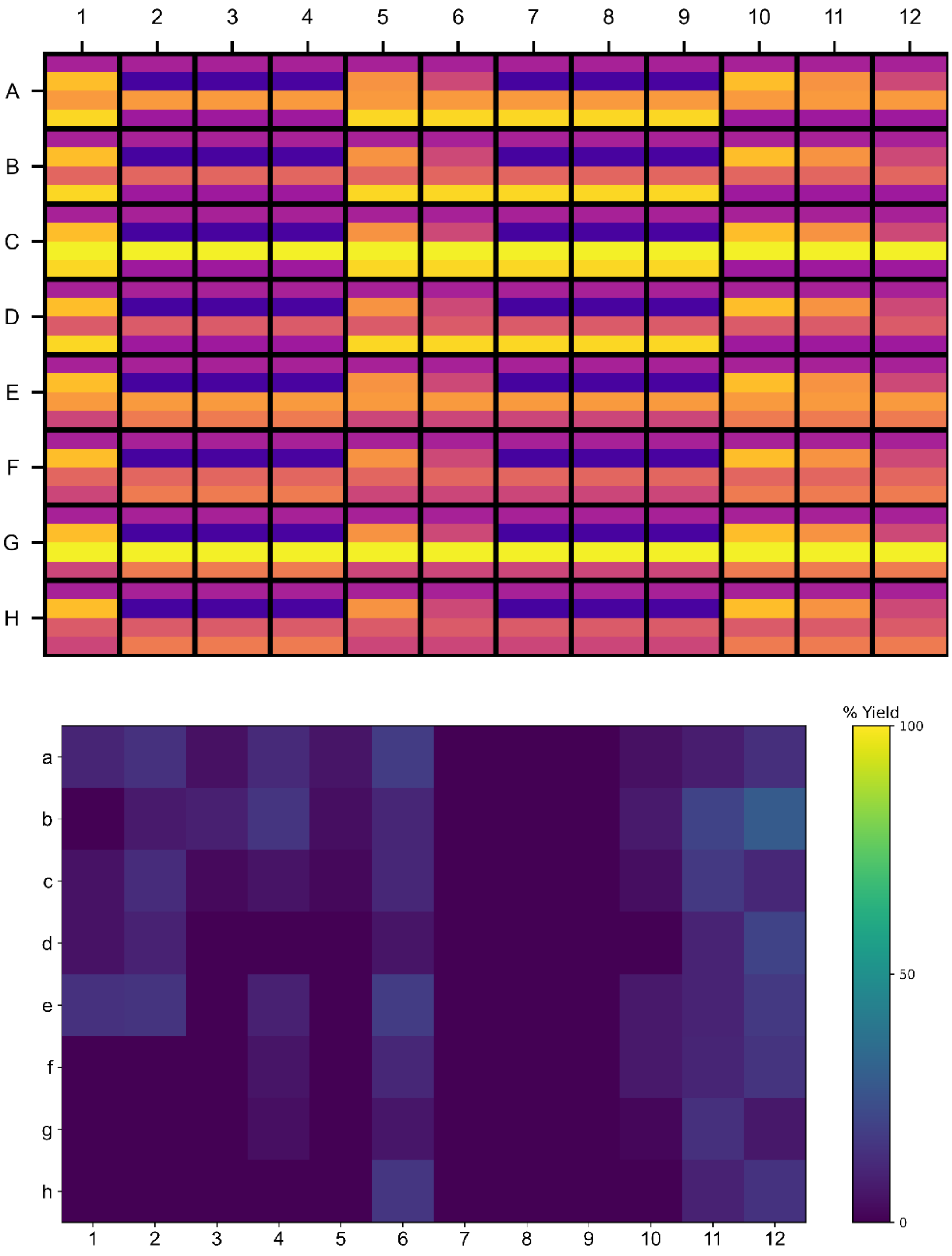

Figure S3. Results of 96 well screen examining 6 Lewis acids, 4 boranes, and 4 silanes.

Screen examining 6 additives, 2 boranes, and 2 gallium loadings

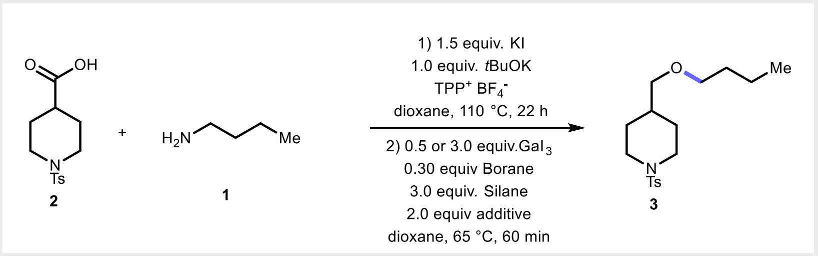

name	molarity	color
Ester	0.1	#f9dd25
Gallium Iodide	0.3	#fbd324
Gallium iodide 0.5 equiv	0.05	#6300a7
Triphenylborane	0.03	#5b01a5
Trimesitylborane	0.03	#fa9e3b
Additive blank	0.1	#2a0593
CHLOROTRIMETHYLSILANE	0.2	#7a02a8
TRIMETHYL BORATE	0.2	#2c0594
HCl in dioxane 4 molar	0.1	#da5a6a
Sodium tetraphenylborate	0.01	#fdcb26
Sodium tetrakis[3,5-bis(trifluoromethyl)phenyl]borate	0.005	#e56b5d

Reagents	Solvent	Cstock (M)	Vdose (µL)	Wells	Order Added
Gallium (III) Iodide	Dioxane	0.9	25	A,B 1-6	1
Gallium (III) Iodide	Dioxane	0.9	25	C,D 1-6	1
Triphenylborane	Dioxane	0.12	25	A,C 1-6	2
Chlorotrimethylsilane	Dioxane	0.12	25	B,D 1-6	2
Blank	Dioxane	0.12	25	A,B,C,D, 1	3
TMSCl	Dioxane	0.6	25	A,B,C,D, 2	3
B(OMe)3	Dioxane	0.6	25	A,B,C,D, 3	3
HCl in dioxane	Dioxane	0.6	25	A,B,C,D, 4	3
Sodium tetraphenylborate	Dioxane	0.6	25	A,B,C,D, 5	3
sodium tetrakis (3,5-CF3 phenyl)borate	Dioxane	0.6	25	A,B,C,D, 6	3
**5 ester solution**	Dioxane	0.3	25	All	4
diphenylsilane	Dioxane	0.9	25	All	5

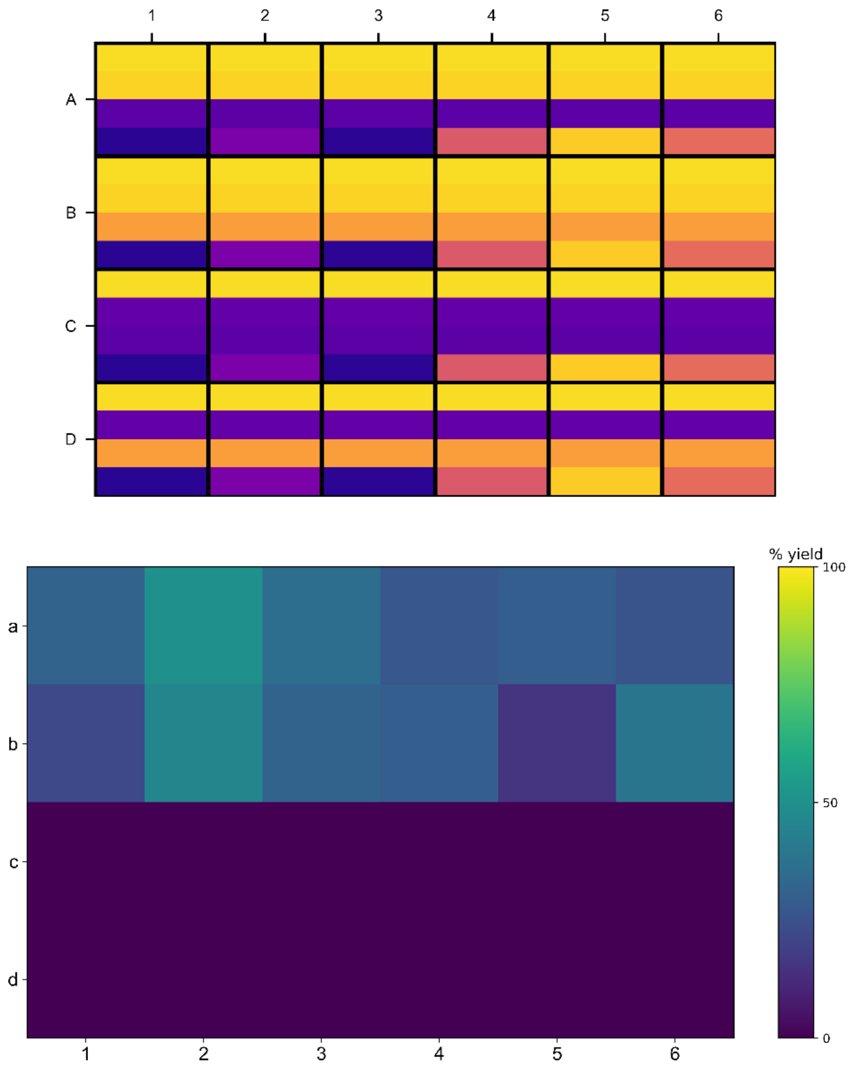

Figure S4. Results of screen examining 6 additives, 2 boranes, and 2 gallium loadings.

Screen examining 6 silyl halides and 4 boranes

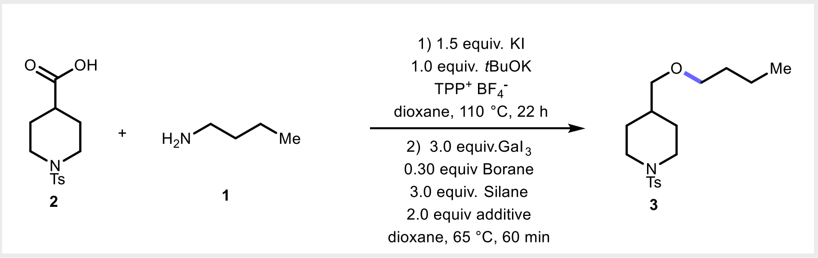

name	molarity	color
Gallium Iodide	0.25	#e06363
Triphenylborane	0.025	#6c00a8
TRIS(PENTAFLUOROPHENYL)BORANE	0.025	#c5407e
tri-mesityl borane	0.025	#f99a3e
Ester solution	0.1	#f7e225
Bromotrimethylsilane	0.2	#e56b5d
CHLOROTRIMETHYLSILANE	0.2	#ca457a
Chlorotriethylsilane	0.2	#c23c81
dichlorodimethylsilane	0.2	#41049d
Trimethoxychlorosilane	0.2	#7e03a8
Additive blank	0.1	#e26561
borane blank	0.1	#8305a7
Diphenylsilane	0.3	#fdae32

Reagents	Solvent	Cstock (M)	Vdose (µL)	Wells	Order Added
Gallium (III) Iodide	Dioxane	1.2	20	All	1
triphenylborane	Dioxane	1.2	20	A 1-6	2
trispentafluorophenyl borane	Dioxane	0.15	20	B 1-6	2
Chlorotrimethylsilane	Dioxane	0.15	20	C 1-6	2
Blank	Dioxane	0.15	20	D 1-6	2
TMSBr	Dioxane	0.8	20	A,B,C,D, 1	3
TMSCl	Dioxane	0.8	20	A,B,C,D, 2	3
TESCl	Dioxane	0.8	20	A,B,C,D, 3	3
Si(Me)2Cl2	Dioxane	0.8	20	A,B,C,D, 4	3
Trimethoxysilyl chloride	Dioxane	0.8	20	A,B,C,D, 5	3
Additive Blank	Dioxane	0.8	20	A,B,C,D, 6	3
**5 ester solution**	Dioxane	0.4	20	All	4
diphenylsilane	Dioxane	1.2	20	All	5

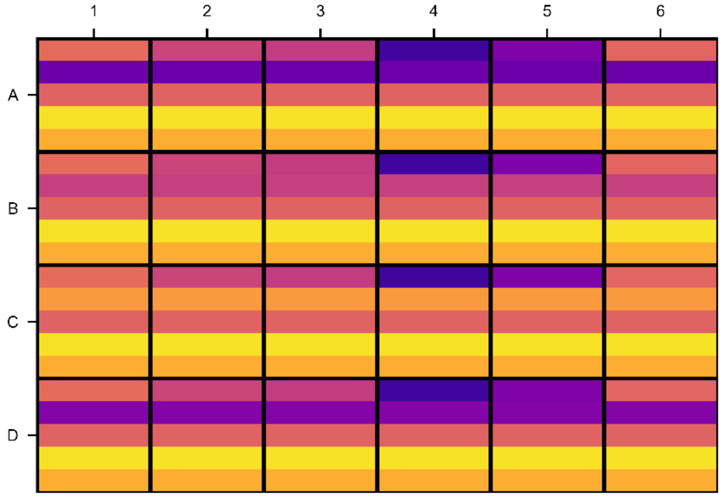

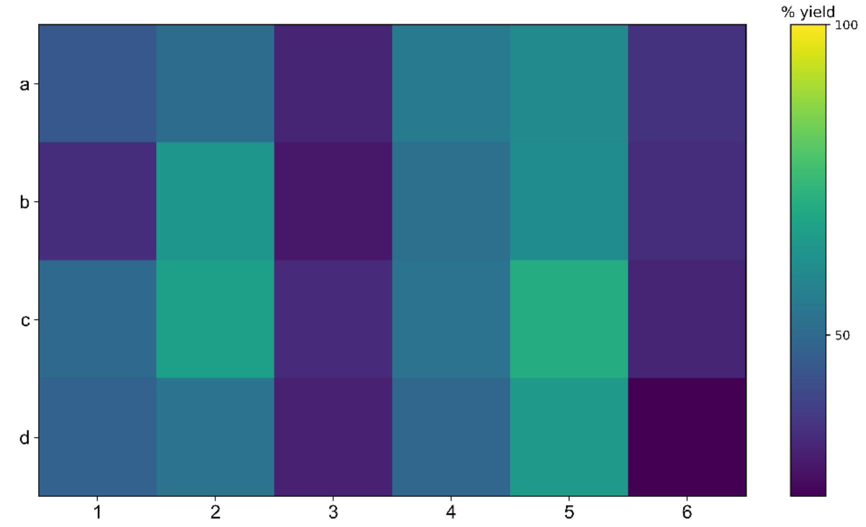

Figure S5. Results of screen examining 6 silyl halides and 4 boranes.

Screen examining 4 solvents and 6 silanes.

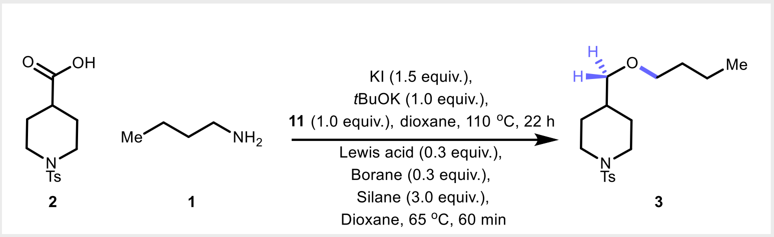

name	molarity	color
Phenylsilane	0.2	#be3885
Diphenylsilane	0.3	#f8e125
triethylsilane	0.4	#e06363
1,1,3,3-tetramethyldisiloxane	0.3	#5601a4
diethylsilane	0.3	#f2f227
dimethylphenylsilane	0.4	#fa9c3c
Ester Solution	0.1	#ed7a52
Gallium Iodide	0.3	#6e00a8
Triphenylborane	0.03	#910ea3
Phenylsilane	0.2	#be3885
Diphenylsilane	0.3	#f8e125
triethylsilane	0.4	#e06363
1,1,3,3-tetramethyldisiloxane	0.3	#5601a4
diethylsilane	0.3	#f2f227
dimethylphenylsilane	0.4	#fa9c3c
Ester Solution	0.1	#ed7a52
Gallium Iodide	0.3	#6e00a8
Triphenylborane	0.03	#910ea3
Phenylsilane	0.2	#be3885
Diphenylsilane	0.3	#f8e125
triethylsilane	0.4	#e06363
1,1,3,3-tetramethyldisiloxane	0.3	#5601a4
diethylsilane	0.3	#f2f227
dimethylphenylsilane	0.4	#fa9c3c
Ester Solution	0.1	#ed7a52
Gallium Iodide	0.3	#6e00a8
Triphenylborane	0.03	#910ea3
Phenylsilane	0.2	#be3885
Diphenylsilane	0.3	#f8e125
triethylsilane	0.4	#e06363
1,1,3,3-tetramethyldisiloxane	0.3	#5601a4
diethylsilane	0.3	#f2f227
dimethylphenylsilane	0.4	#fa9c3c
Ester Solution	0.1	#ed7a52
Gallium Iodide	0.3	#6e00a8
Triphenylborane	0.03	#910ea3

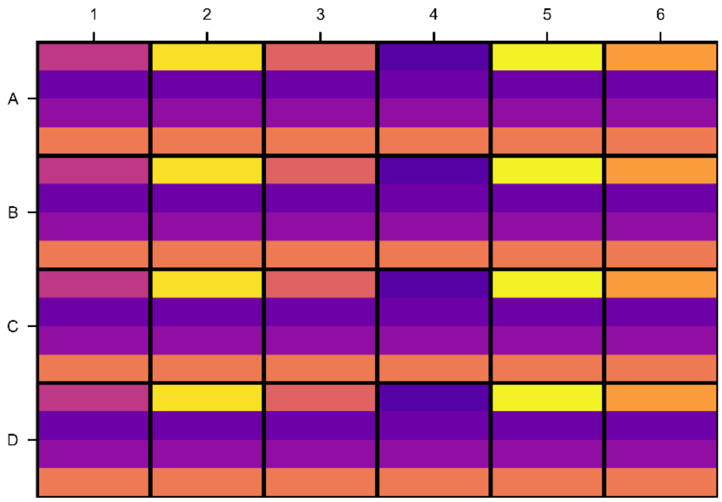

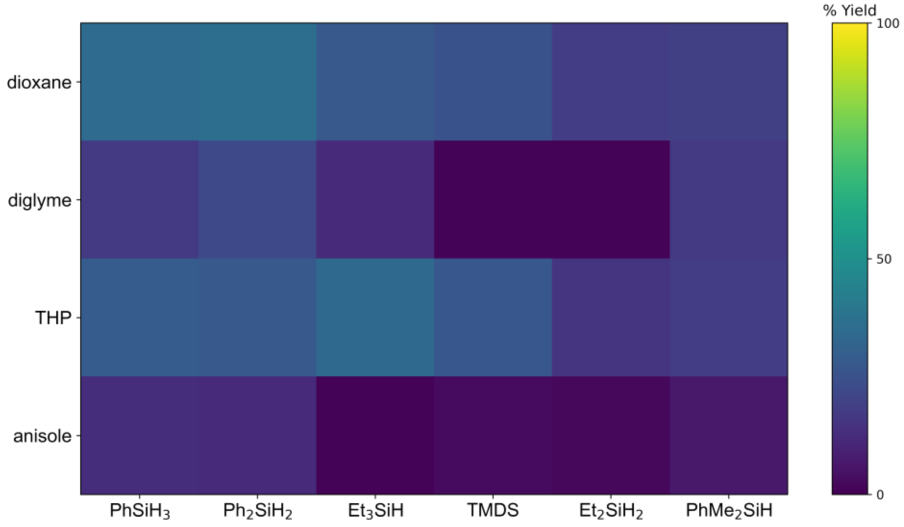

Figure S6. Results of screen examining 4 solvents and 6 silanes.

Screen examining 16 Lewis Acids at 3 loadings and 2 cocatalysts

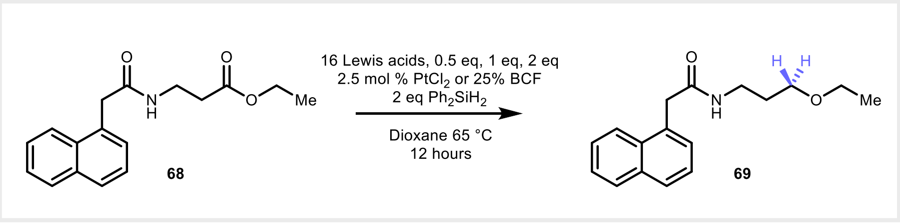

Reagents	Solvent	Cstock (M)	Vdose (µL)	Wells	Order Added
GaCl3 (3 equiv)	Dioxane	1.2	25	A 1,3	1
PdCl2 (2 equiv)	Dioxane	0.8	25	B 1,3	1
AgNO3(2 equiv)	Dioxane	0.8	25	C 1,3	1
AuCl (2 equiv)	Dioxane	0.8	25	D 1,3	1
BiCl3 (2 equiv)	Dioxane	0.8	25	E 1,3	1
RuCl3 (2 equiv)	Dioxane	0.8	25	F 1,3	1
InCl3 (2 equiv)	Dioxane	0.8	25	G 1,3	1
FeCl2 (2 equiv)	Dioxane	0.8	25	H 1,3	1
Co(acac)3 (2 equiv)	Dioxane	0.8	25	A 2,4	1
AlCl3 (2 equiv)	Dioxane	0.8	25	B 2,4	1
NiCl2 (2 equiv)	Dioxane	0.8	25	C 2,4	1
FeCl3 (2 equiv)	Dioxane	0.8	25	D 2,4	1
ZrCl4 (2 equiv)	Dioxane	0.8	25	E 2,4	1
ZnCl2 (2 equiv)	Dioxane	0.8	25	F 2,4	1
CuCl (2 equiv)	Dioxane	0.8	25	G 2,4	1
MnCl2 (2 equiv)	Dioxane	0.8	25	H 2,4	1
GaCl3 (1.5 equiv)	Dioxane	0.6	25	A 5,7	1
PdCl2 (1 equiv)	Dioxane	0.4	25	B 5,7	1
AgNO3(1 equiv)	Dioxane	0.4	25	C 5,7	1
AuCl (1 equiv)	Dioxane	0.4	25	D 5,7	1
BiCl3 (1 equiv)	Dioxane	0.4	25	E 5,7	1
RuCl3 (1 equiv)	Dioxane	0.4	25	F 5,7	1
InCl3 (1 equiv)	Dioxane	0.4	25	G 5,7	1
FeCl2 (1 equiv)	Dioxane	0.4	25	H 5,7	1
Co(acac)3 (1 equiv)	Dioxane	0.4	25	A 6,8	1
AlCl3 (1 equiv)	Dioxane	0.4	25	B 6,8	1
NiCl2 (1 equiv)	Dioxane	0.4	25	C 6,8	1
FeCl3 (1 equiv)	Dioxane	0.4	25	D 6,8	1
ZrCl4 (1 equiv)	Dioxane	0.4	25	E 6,8	1
ZnCl2 (1 equiv)	Dioxane	0.4	25	F 6,8	1
CuCl (1 equiv)	Dioxane	0.4	25	G 6,8	1
MnCl2 (1 equiv)	Dioxane	0.4	25	H 6,8	1
GaCl3 (0.75 equiv)	Dioxane	0.3	25	A 9,11	1
PdCl2 (0.5 equiv)	Dioxane	0.2	25	B 9,11	1
AgNO3(0.5 equiv)	Dioxane	0.2	25	C 9,11	1
AuCl (0.5 equiv)	Dioxane	0.2	25	D 9,11	1
BiCl3 (0.5 equiv)	Dioxane	0.2	25	E 9,11	1
RuCl3 (0.5 equiv)	Dioxane	0.2	25	F 9,11	1
InCl3 (0.5 equiv)	Dioxane	0.2	25	G 9,11	1
FeCl2 (0.5 equiv)	Dioxane	0.2	25	H 9,11	1
Co(acac)3 (0.5 equiv)	Dioxane	0.2	25	A 10,12	1
AlCl3 (0.5 equiv)	Dioxane	0.2	25	B 10,12	1
NiCl2 (0.5 equiv)	Dioxane	0.2	25	C 10,12	1
FeCl3 (0.5 equiv)	Dioxane	0.2	25	D 10,12	1
ZrCl4 (0.5 equiv)	Dioxane	0.2	25	E 10,12	1
ZnCl2 (0.5 equiv)	Dioxane	0.2	25	F 10,12	1
CuCl (0.5 equiv)	Dioxane	0.2	25	G 10,12	1
MnCl2 (0.5 equiv)	Dioxane	0.2	25	H 10,12	1
trispentafluorophenylborane	Dioxane	0.1	25	A-H 1,2,5,6,9,10	2
PtCl2	Dioxane	0.04	25	A-H 3,4,7,8,11,12	2
**68**	Dioxane	0.4	25	All	3
diphenylsilane	Dioxane	0.8	25	All	4

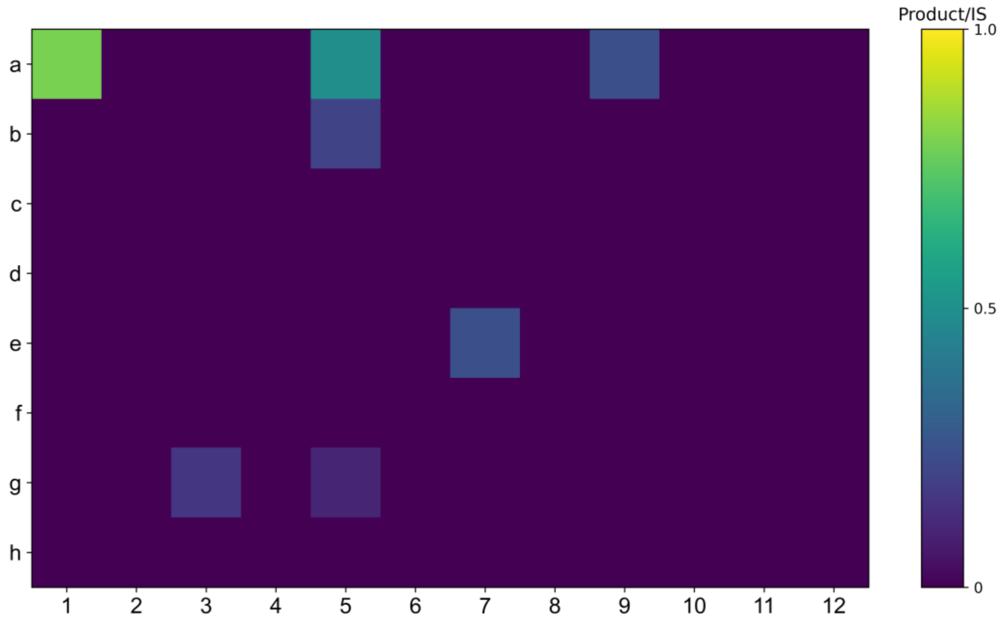

Figure S7. Results of amide selectivity screen.

Extended Etherification Optimization Data

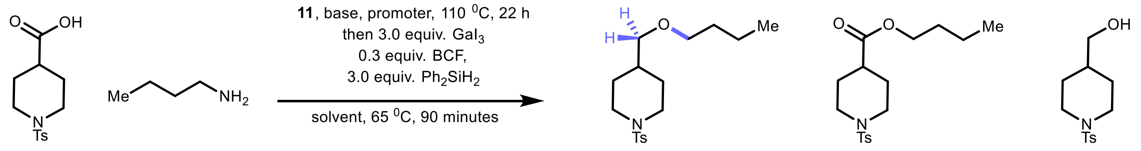

entry	base	Promoter	Concentration (Step 1)	Concentration (Step 2)	Solvent	LC yield ether	Pre-reduction LC yield ester	Post-reduction LC yield ester
1	KO*t*Bu	KI	0.3 M	0.1 M	dioxane	40%	70%	ND
2	KO*t*Bu	KBr	0.3 M	0.1 M	dioxane	35%	65%	ND
3	DIPEA	KI	0.3 M	0.1 M	dioxane	35%	63%	ND
4	KO*t*Bu	KI	0.3 M	0.1 M	MeCN	ND	55%	55%
5	KO*t*Bu	KI	0.3 M	0.1 M	$CF_3C_6H_5$	20%	45%	20%
6	KO*t*Bu	KI	0.3 M	0.1 M	Pyridine	ND	ND	ND
7	KO*t*Bu	KI	0.3 M	0.1 M	DCE	25%	48%	22%
8	KO*t*Bu	KI	0.3 M	0.3 M	dioxane	40%	70%	48%
9	KO*t*Bu	KI	0.1 M	0.1 M	dioxane	28%	40%	ND
10	KO*t*Bu	KI	0.3 M	0.05 M	dioxane	36%	72%	5%

Table S1. Extended optimization table for gallium promoted etherification examining solvent, promoter, and concentration. Alcohol SI-10 is the major byproduct of the reaction and accounts for any difference between the sum of ether and post-reduction ester yields vs pre-reduction ester yields.

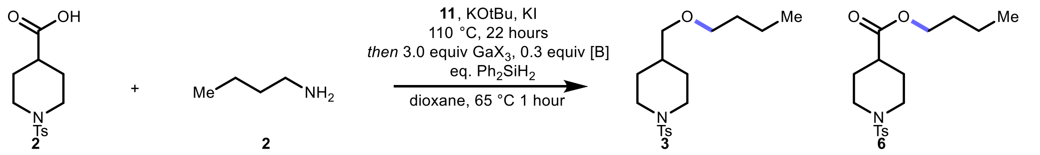

entry	Ga source	Boron Source	Ligand	LC yield ether (isolated)	LC yield ester
1	GaI_3	Mes_3B	None	65% (63%)	ND
2	GaOTf	Mes_3B	None	Trace	77 %
3	GaI_3	BCF	None	60%	ND
4	GaI_3	BF_3	None	20%	ND
5	GaI_3	$B(OMe)_3$	None	25%	ND
6	GaI_3	$PhB(OH)_2$	None	35%	25%
7	GaI_3	Mes_3B	None	40%	54%
8	GaI_3	Mes_3B	PCy_3	ND	79%
9	GaI_3	Mes_3B	**L1**	ND	76%
10	GaI_3	Mes_3B	**L2**	ND	77%

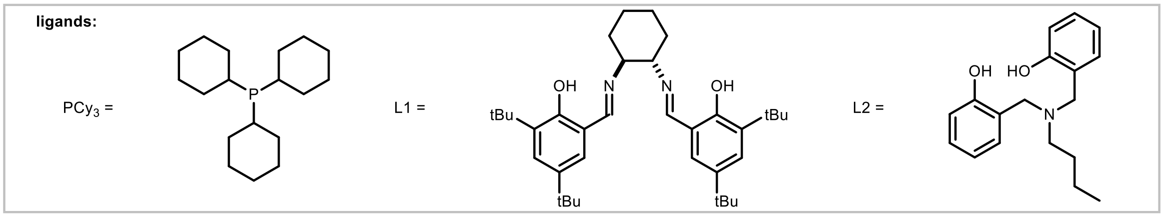

Table S2. Extended optimization table of gallium promoted etherification examining gallium source, boron source, and the effect of ligands.

NMRs of Mechanistic studies

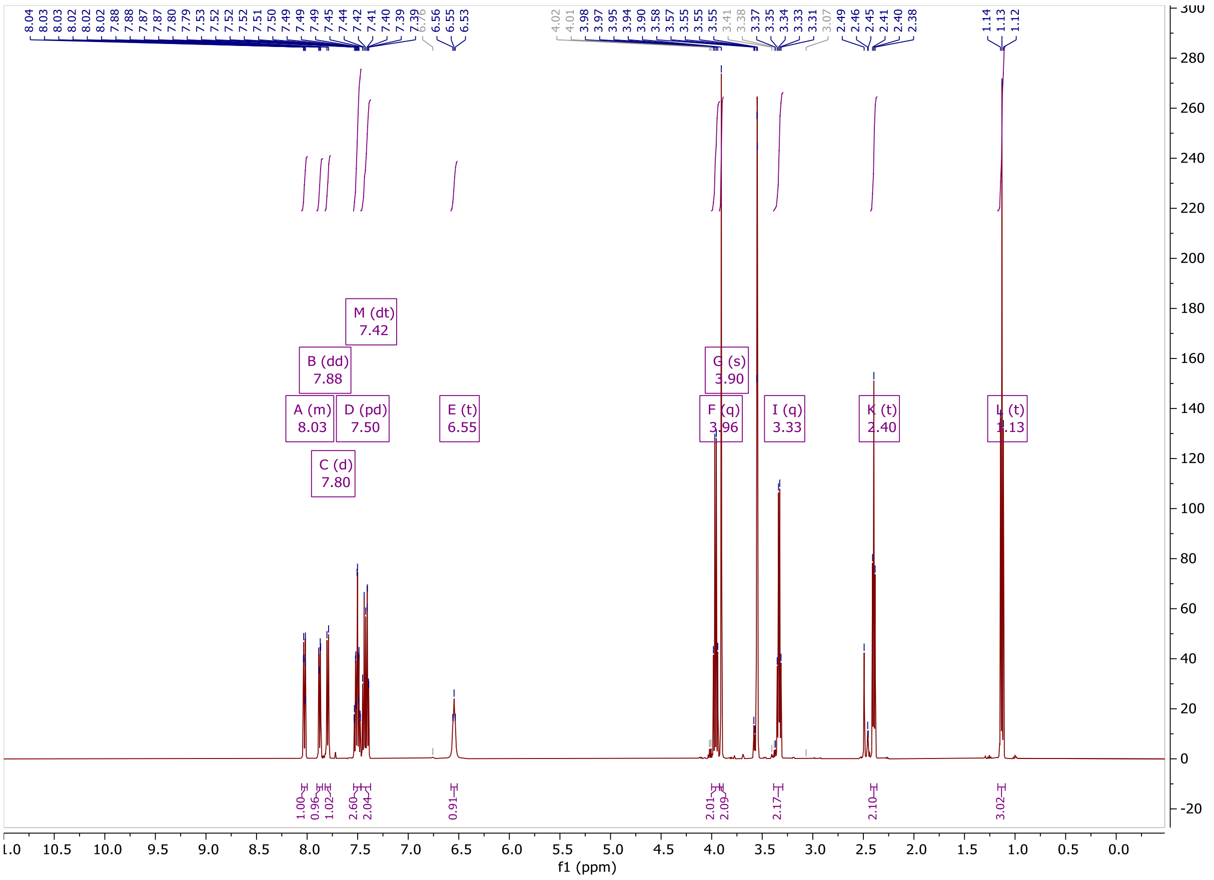

Spectrum S1. ^{1}H NMR of **68** in dioxane d_8.

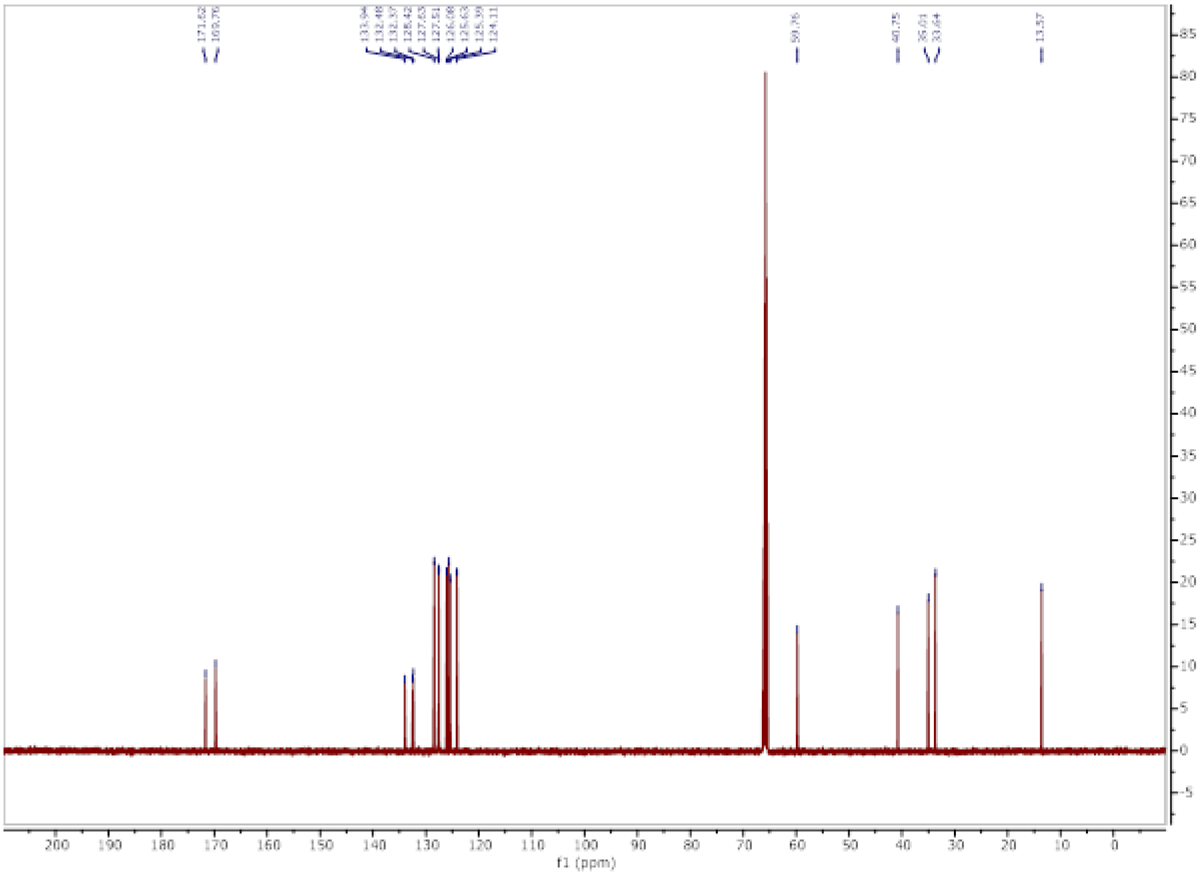

Spectrum S2. ^{13}C NMR of compound **68** in Dioxane d_8.

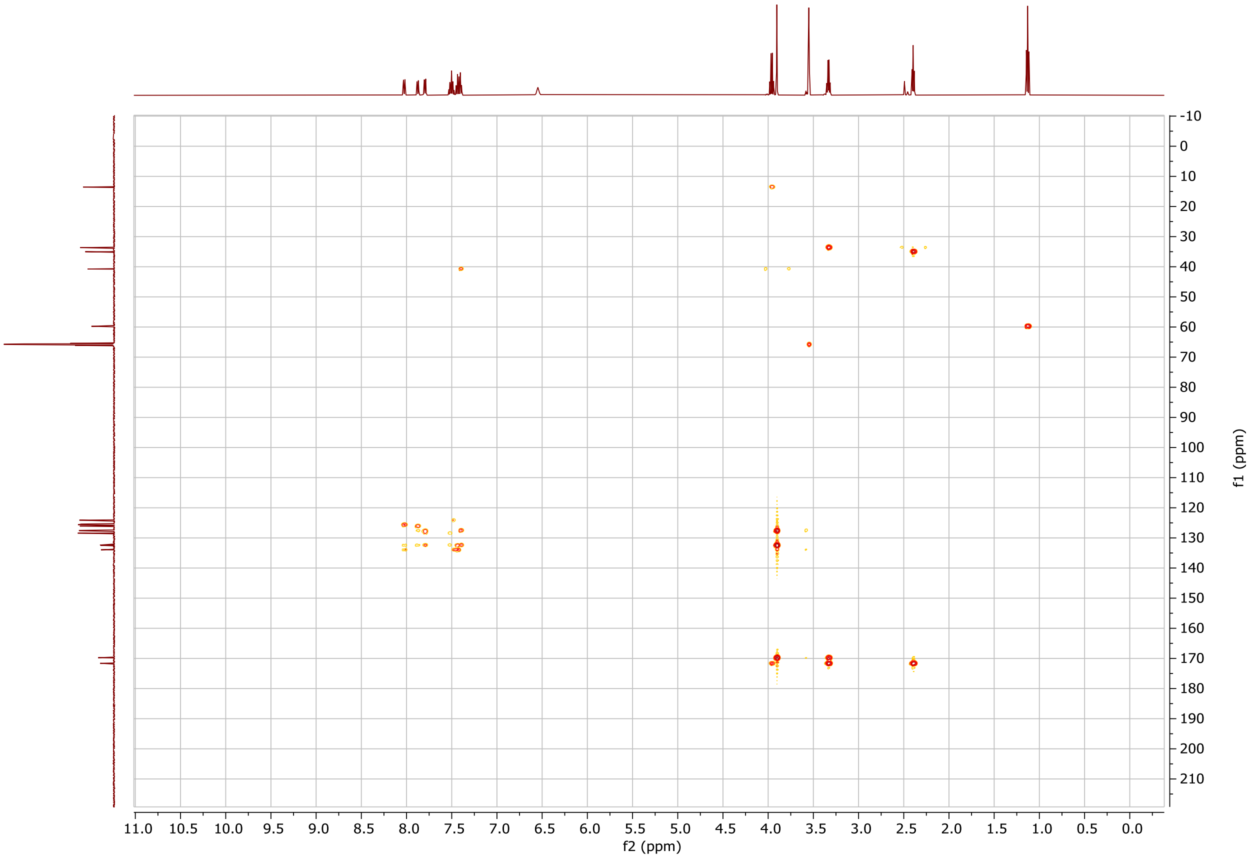

Spectrum S3. Full HMBC of **68** in dioxane d_8.

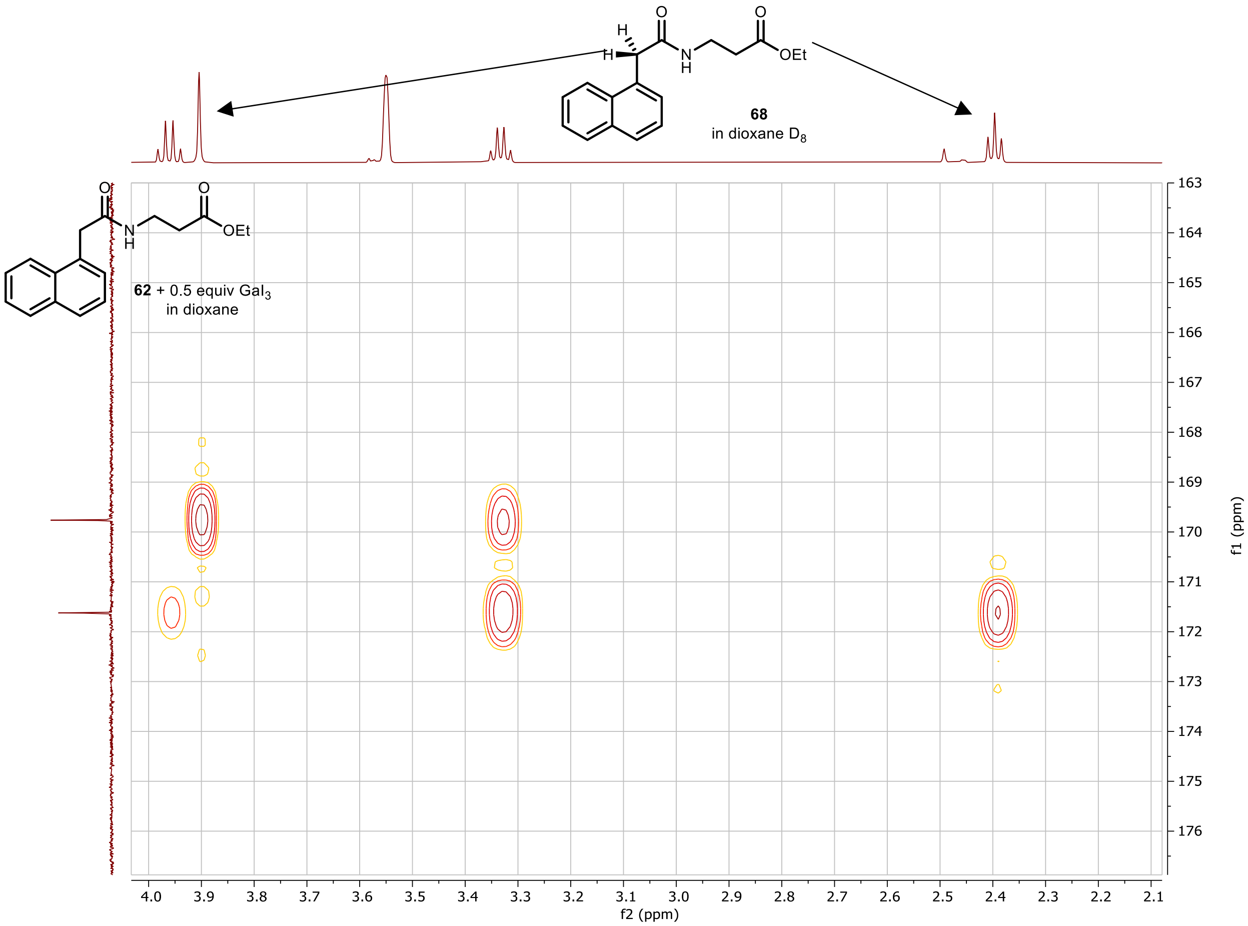

Spectrum S4. Zoomed HMBC of **68.**

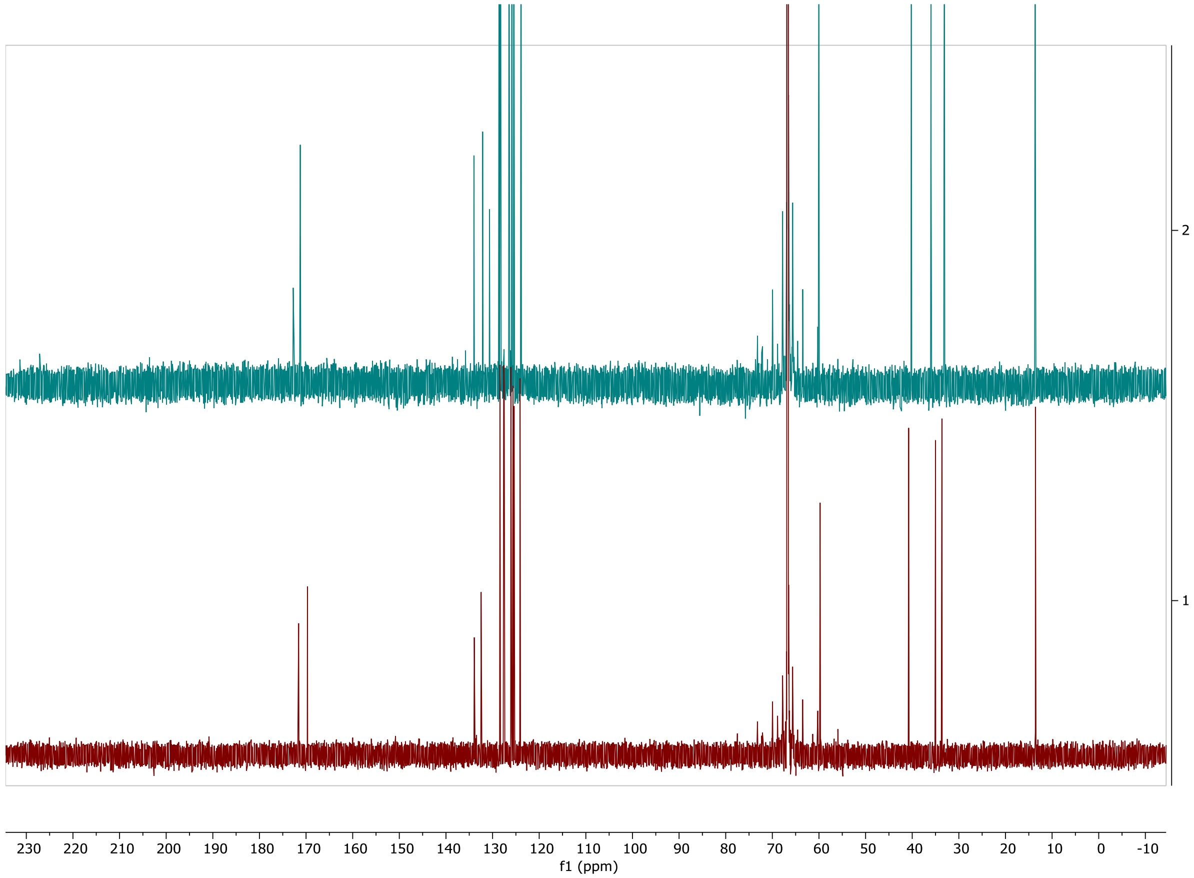

Spectrum S5. ^{13}C NMR of **68** in dioxane (red, bottom) vs **68** + 0.5 equiv. of GaI_3 (teal, top) full spectrum.

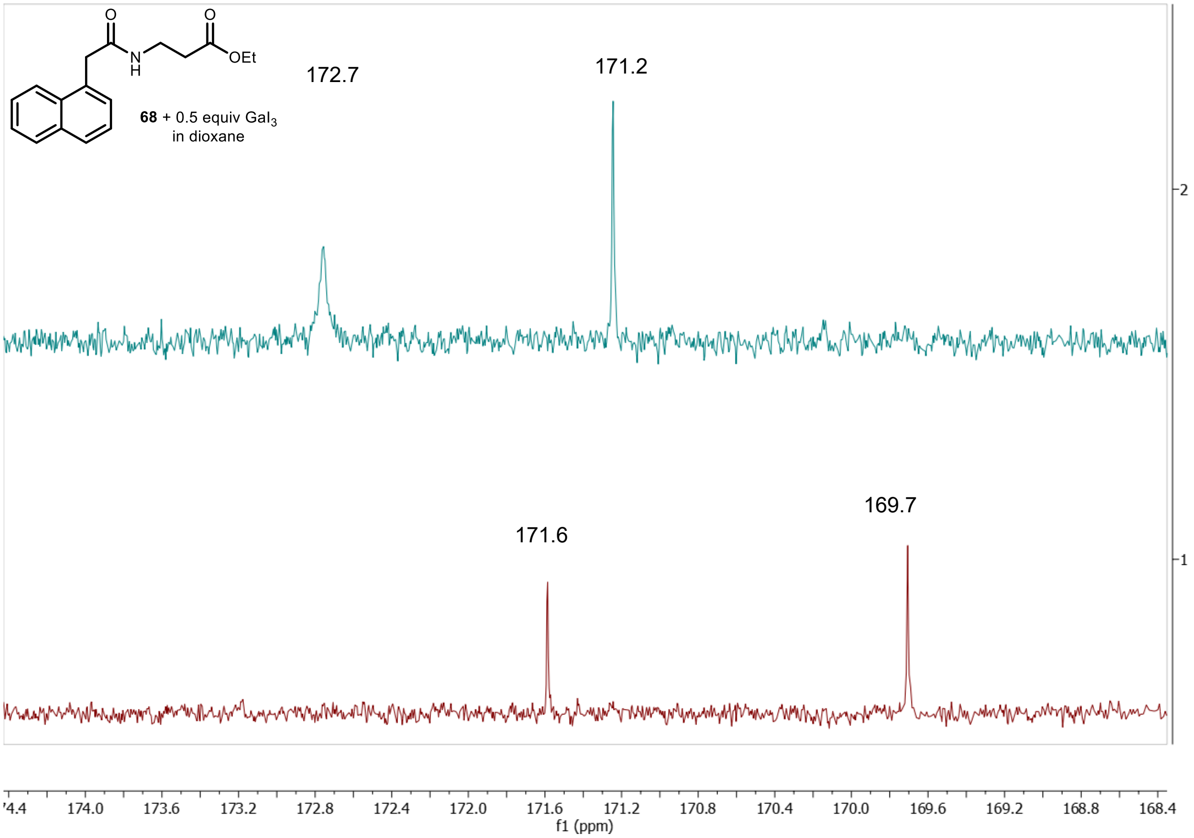

Spectrum S6. ^{13}C NMR of **68** in dioxane (red, bottom) vs **68** + 0.5 equiv. of GaI_3 (teal, top) carbonyl region.

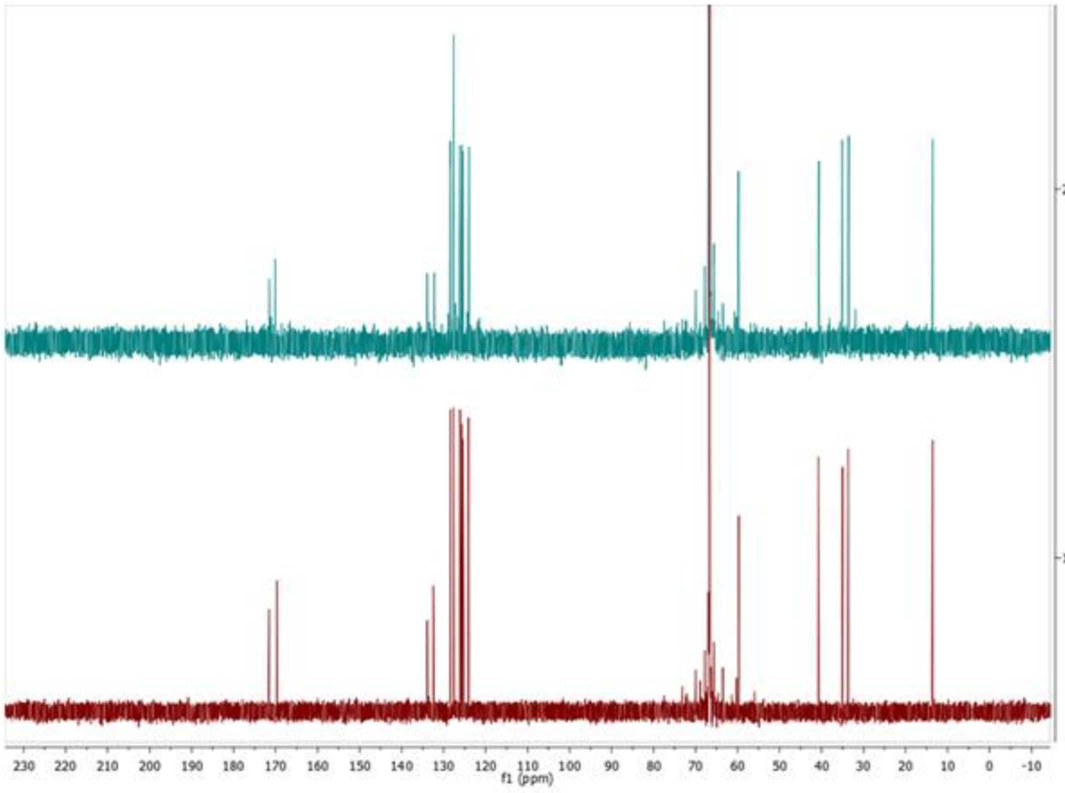

Spectrum S7. ^{13}CNMR of **68** in dioxane (red, bottom) vs **68** + 0.25 equiv. of trispentafluorophenyl (teal, top) full spectrum.

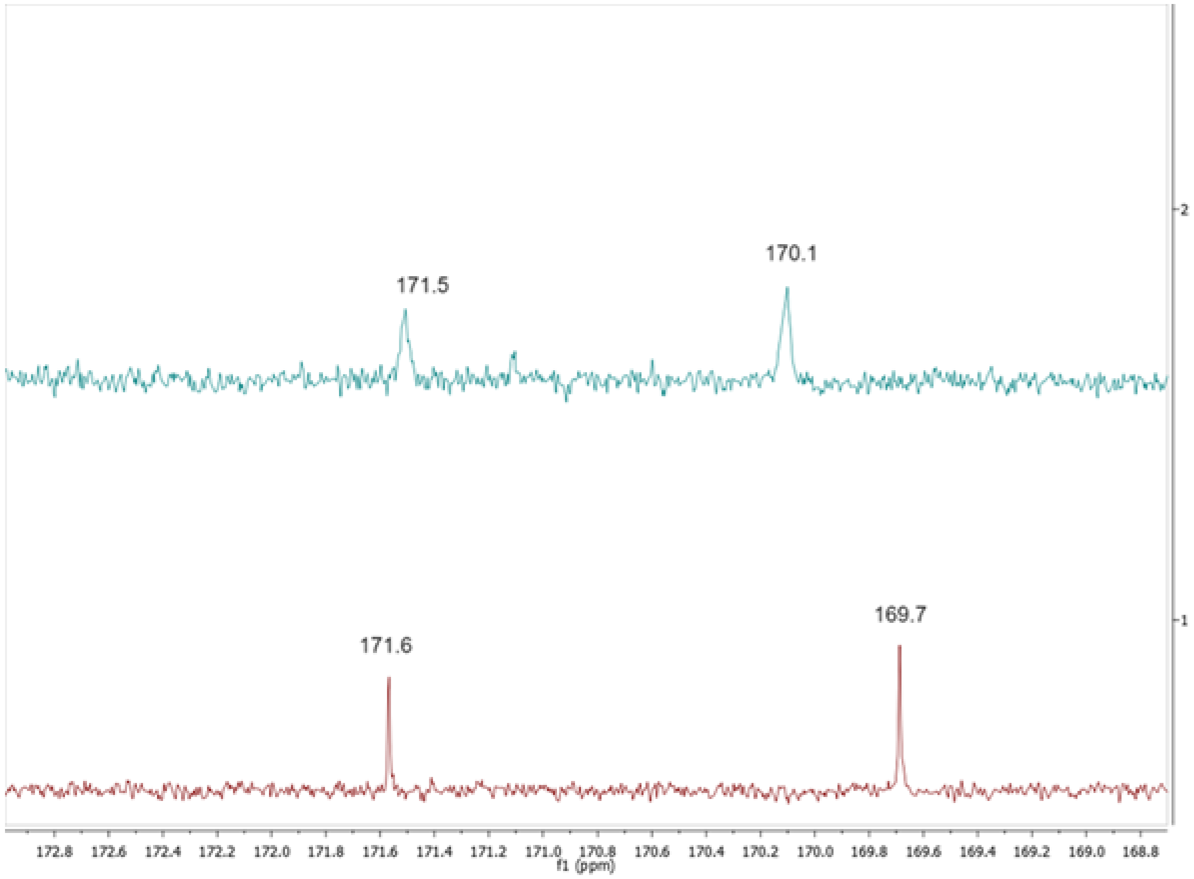

Spectrum S8. ^{13}C NMR of **68** in dioxane (red, bottom) vs **68** + 0.25 equiv. of trispentafluorophenyl (teal, top) carbonyl region.

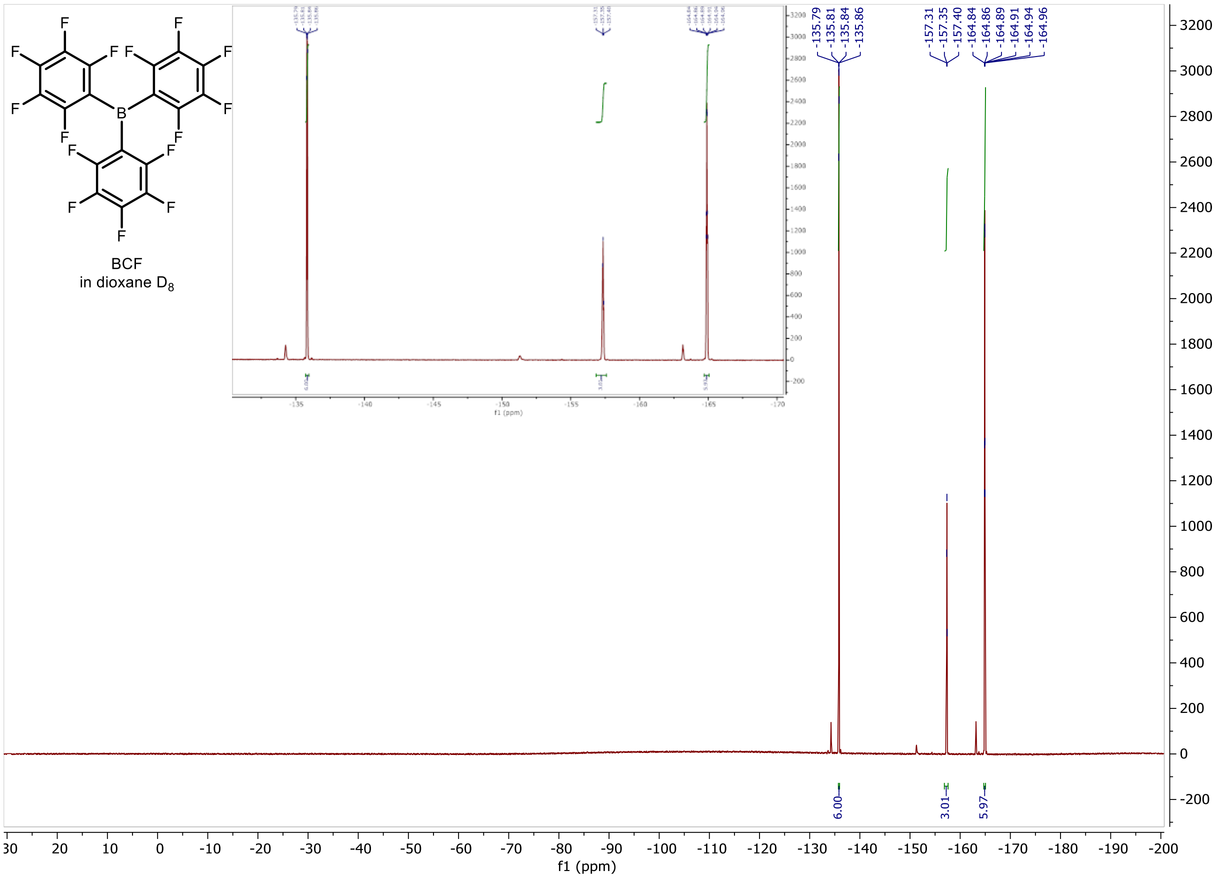

Spectrum S9. ^{19}F NMR of trispentafluorophenyl borane in dioxane d_8.

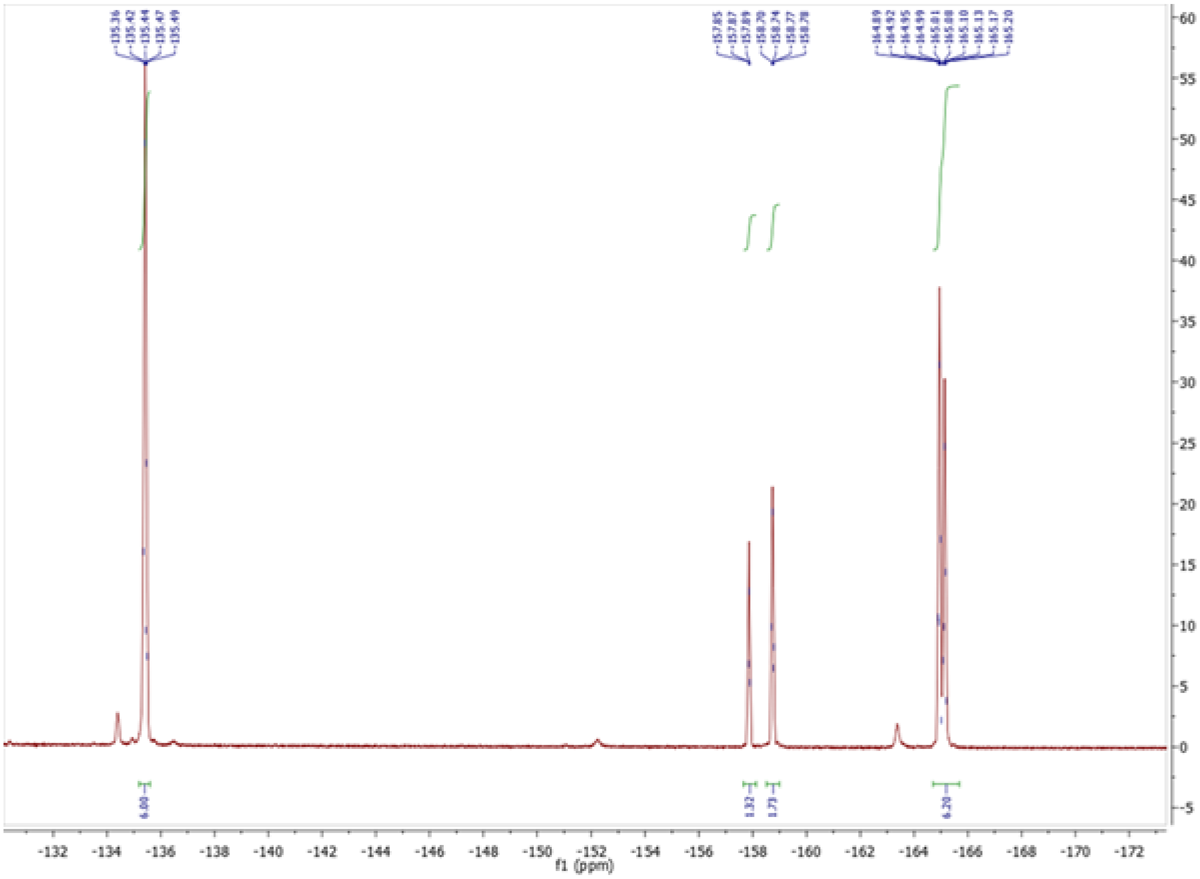

Spectrum S10. ^{19}F NMR of **68** + 0.25 equiv. of trispentalfuorophenylborane zoomed to region containing peaks.

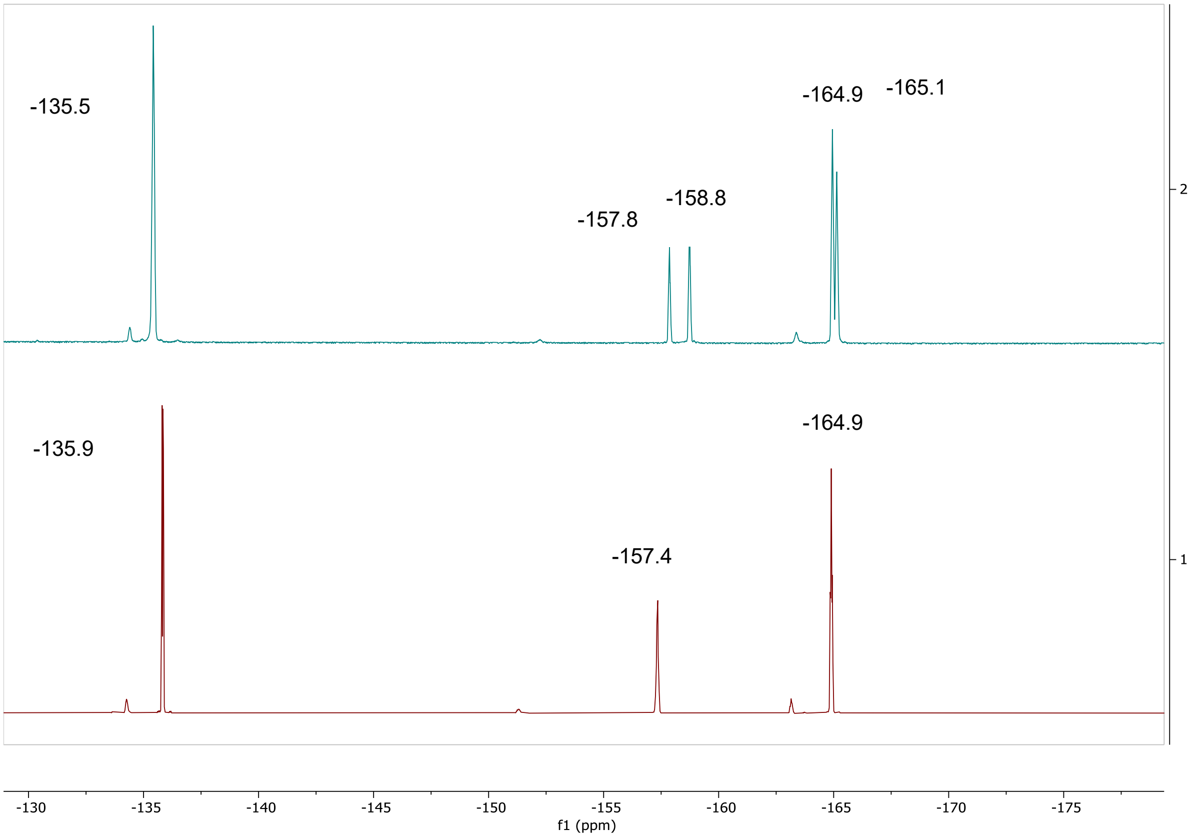

Spectrum S11. ^{19}F NMR of trispentalfuorophenylborane in dioxane (red, bottom) vs **68** + 0.25 equiv. of trispentafluorophenyl (teal, top) peaks region.

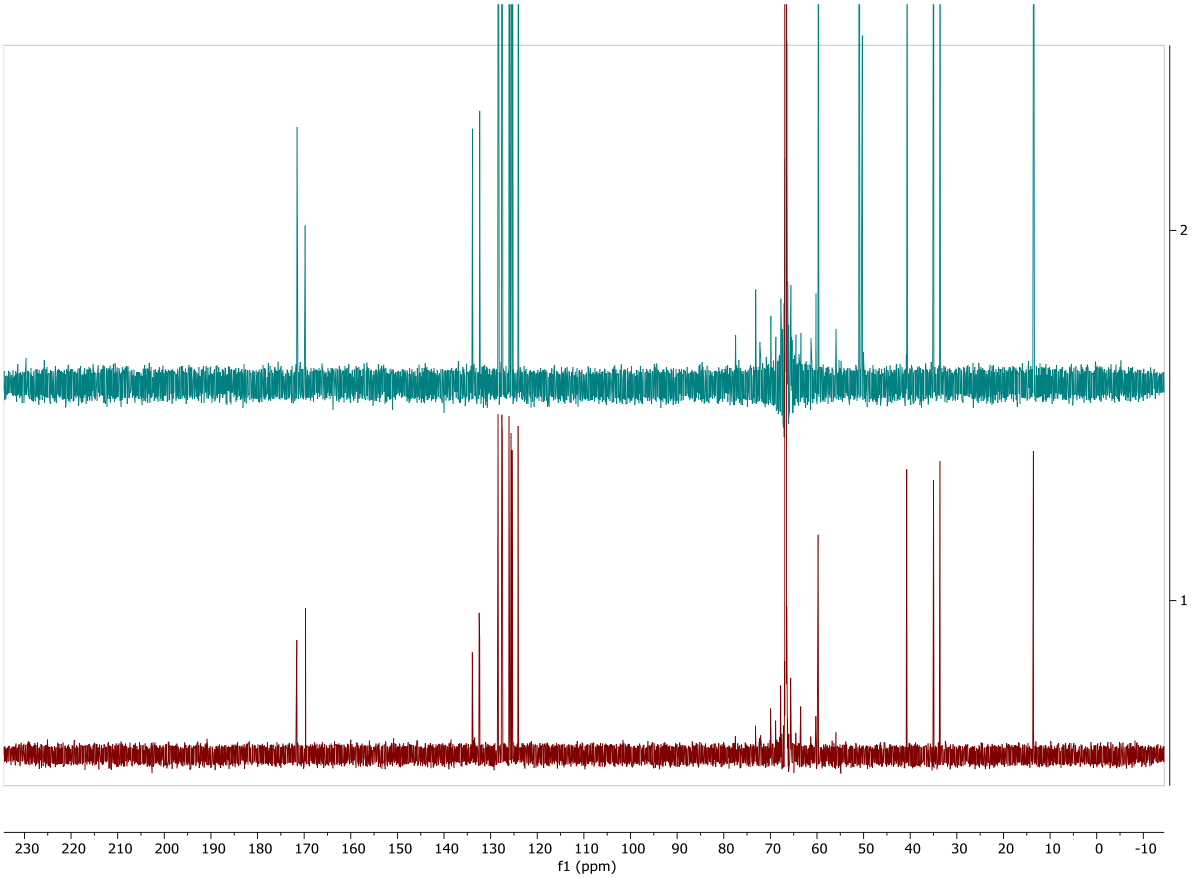

Spectrum S12. ^{13}C NMR of **68** in dioxane (red, bottom) vs **68** + 2.0 equiv. of trimethoxysilylchloride (teal, top) full spectrum.

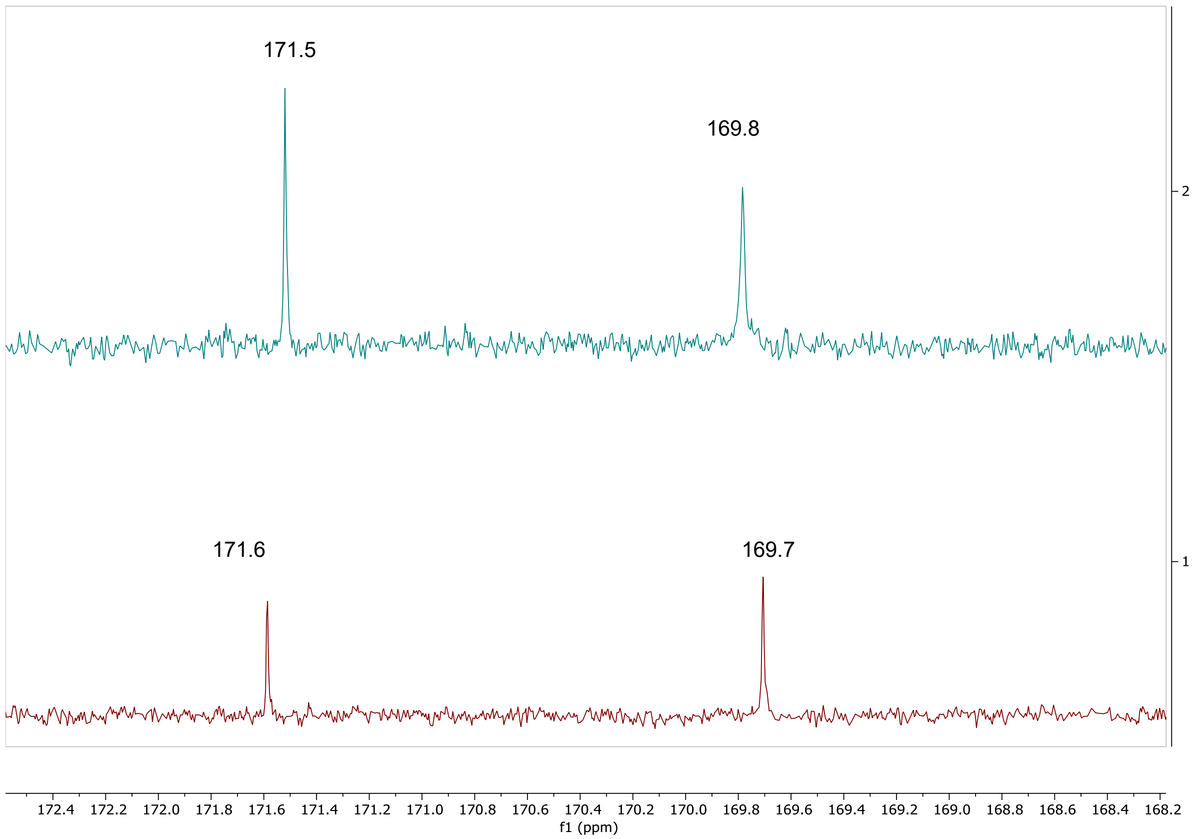

Spectrum S13. ^{13}C-NMR of **68** in dioxane (red, bottom) vs **68** + 2.0 equiv. of trimethoxysilyl chloride (teal, top) carbonyl region.

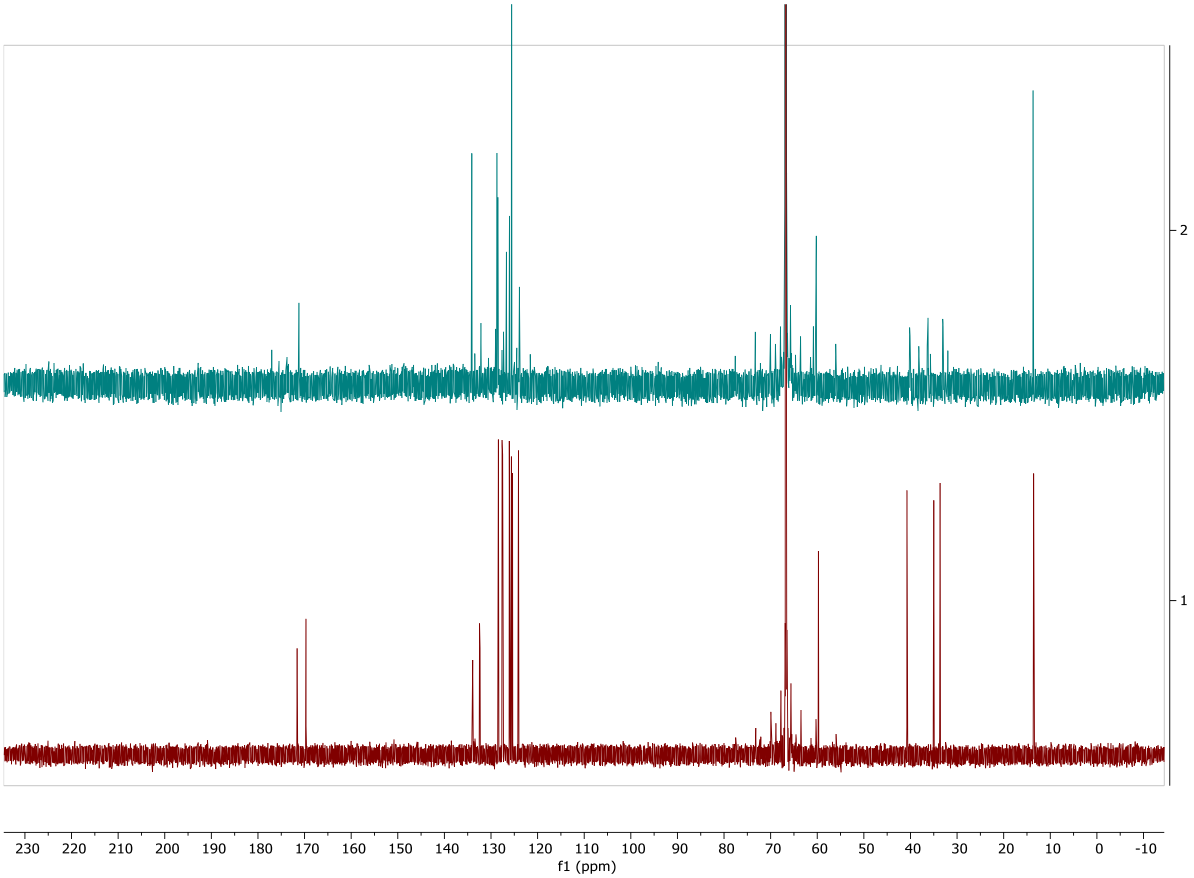

Spectrum S14. ^{13}C NMR of **68** in dioxane (red, bottom) vs **68** + 0.5 equiv. of GaI_3 and 0.25 equiv. of trispentafluorophenyl borane (teal, top) full spectrum.

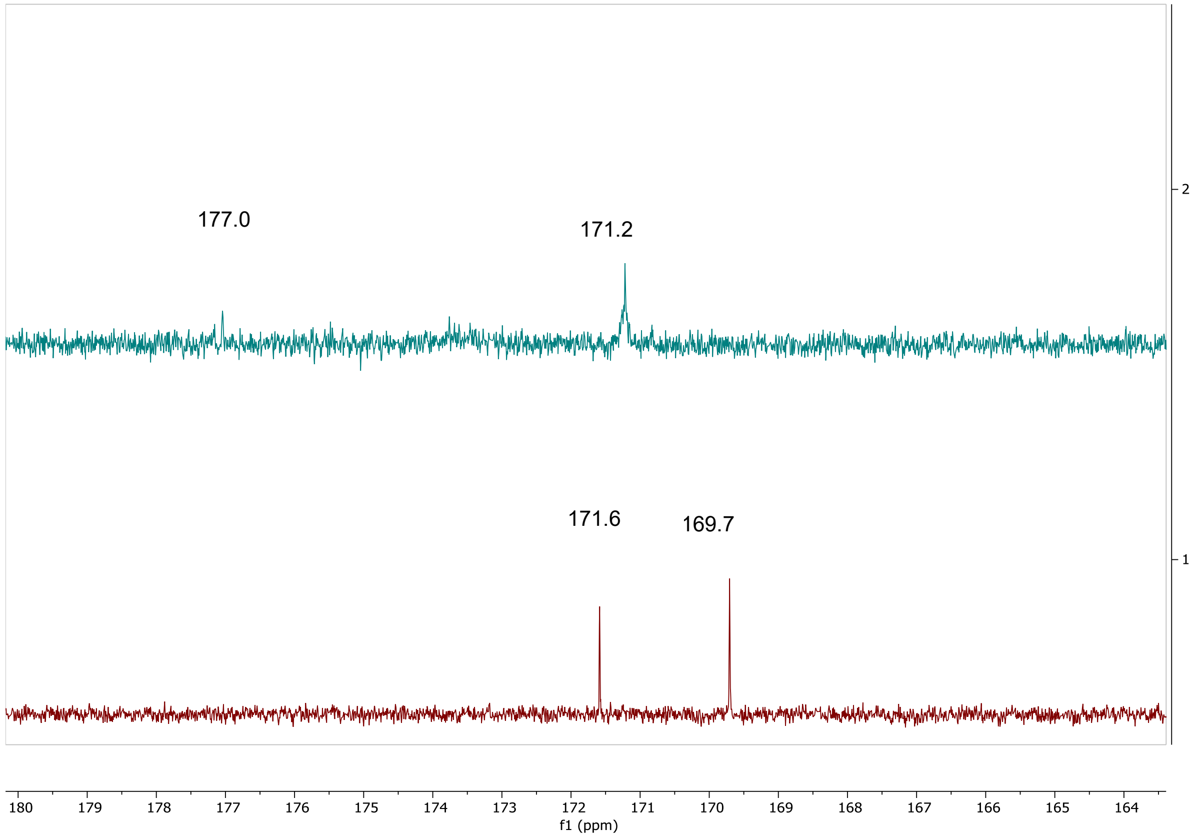

Spectrum S15. ^{13}C NMR of **68** in dioxane (red, bottom) vs **68** + 0.5 equiv. of GaI_3 and 0.25 equiv. of trispentafluorophenyl borane (teal, top) carbonyl region.

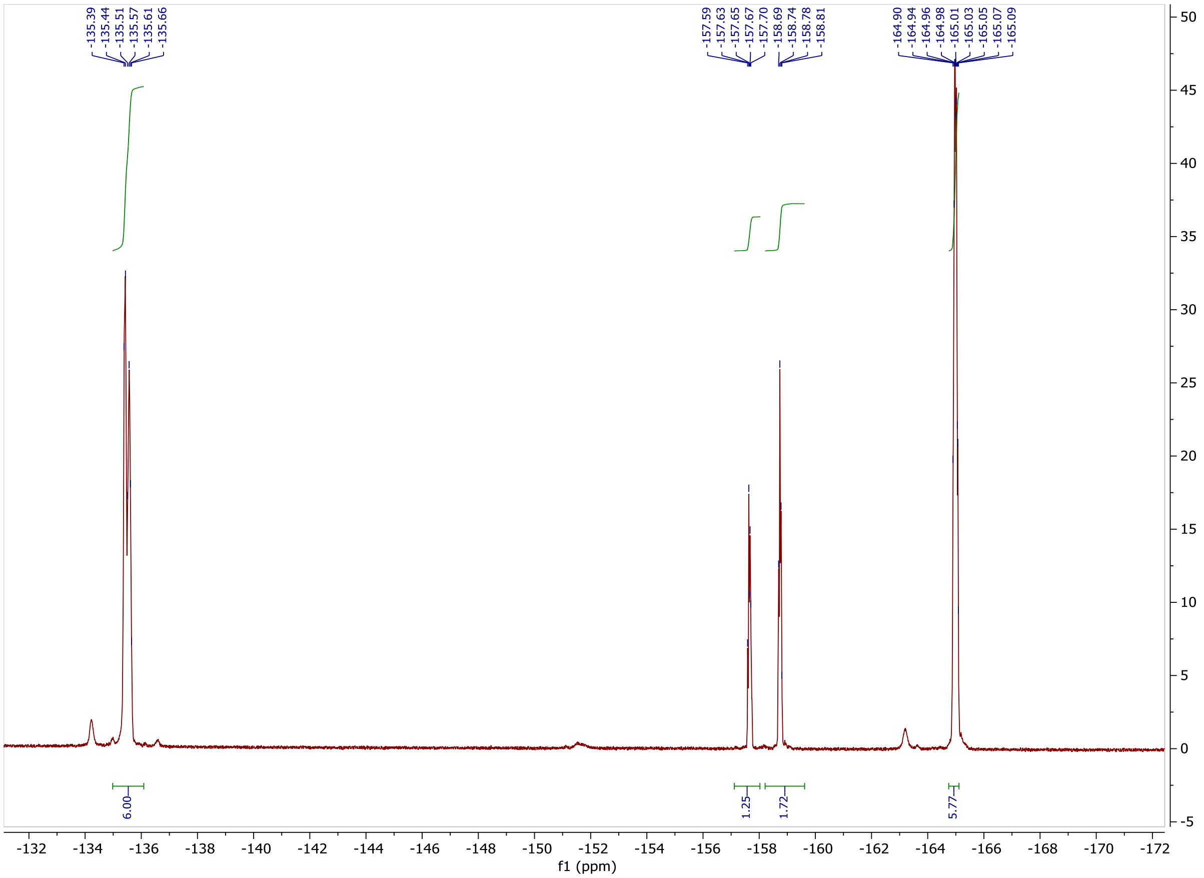

Spectrum S16. ^{19}F NMR of **68** + 0.5 equiv. of GaI_3 and 0.25 equiv. of trispentafluorophenyl borane (teal, top) peaks region.

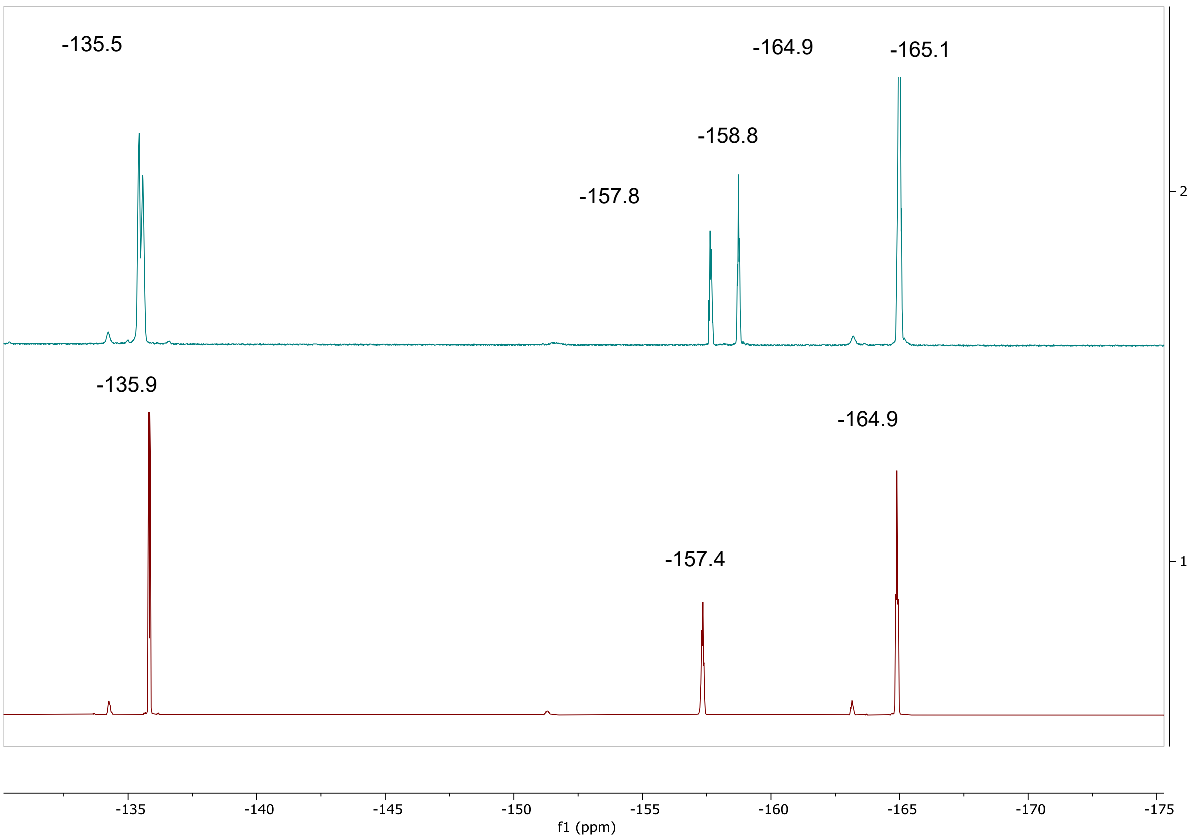

Spectrum S17. ^{19}F NMR of **68** in dioxane (red, bottom) vs **68** + 0.5 equiv. of GaI_3 and 0.25 equiv. of trispentafluorophenyl borane (teal, top) peaks region.

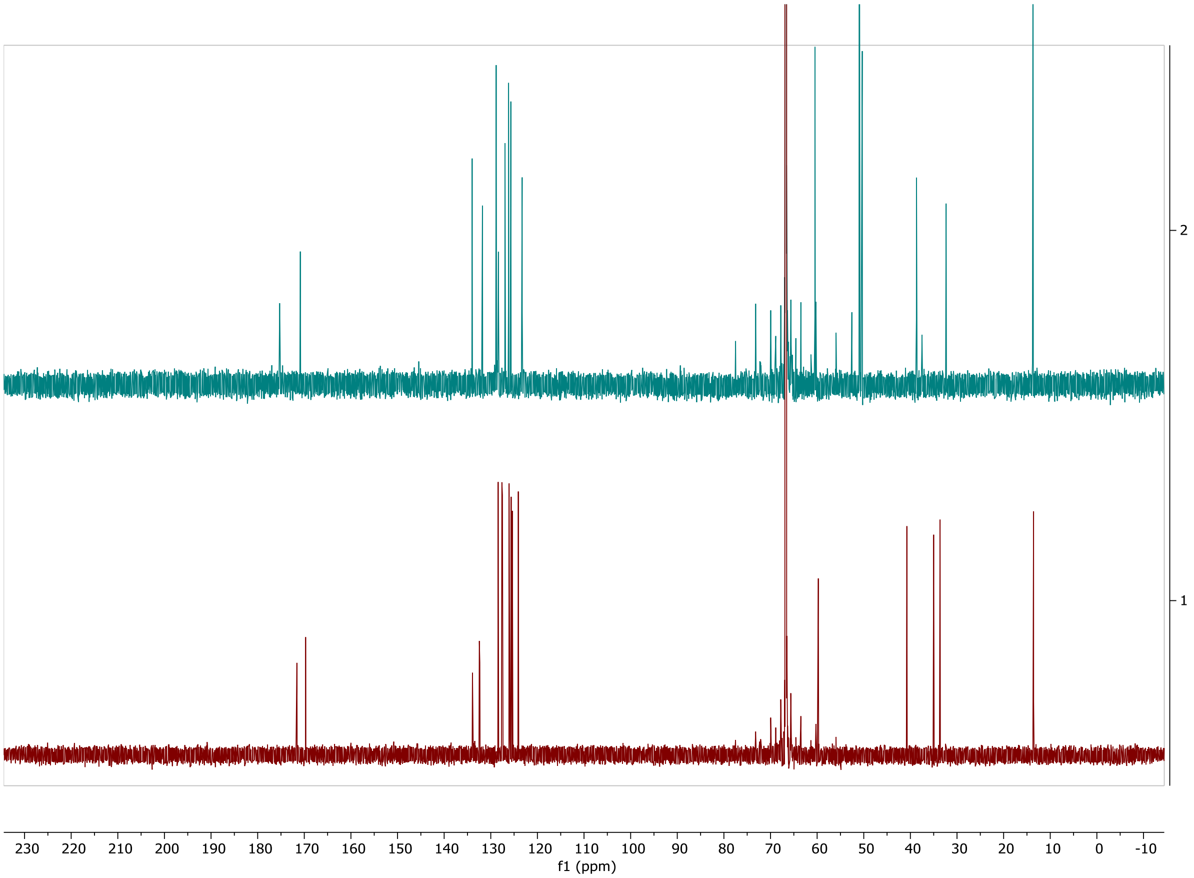

Spectrum S18. ^{13}C NMR of **68** in dioxane (red, bottom) vs **68** + 0.5 equiv. of GaI_3 and 2.0 equiv. of trimethoxysilyl chloride (teal, top) full spectrum.

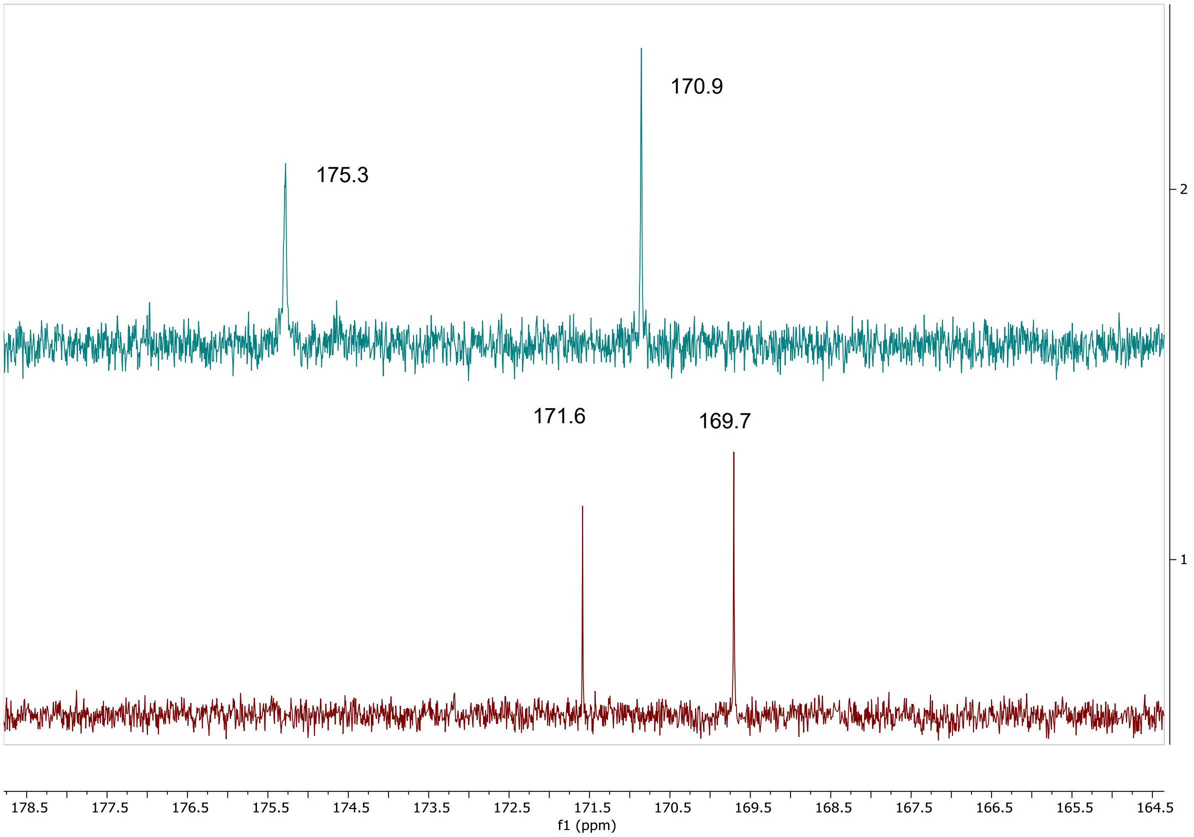

Spectrum S19. ^{13}C NMR of **68** in dioxane (red, bottom) vs **68** + 0.5 equiv. of GaI_3 and 2.0 equiv. of trimethoxysilyl chloride (teal, top) carbonyl region.

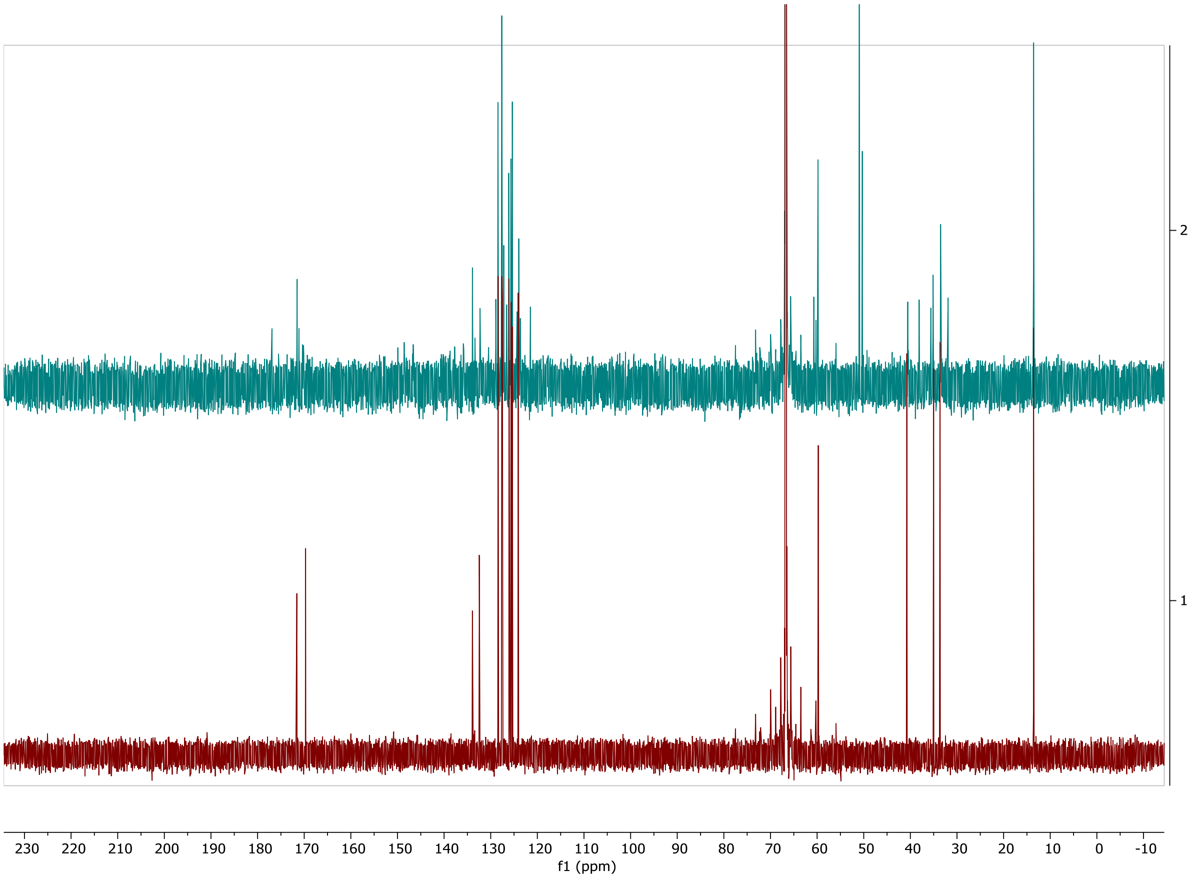

Spectrum S20. ^{13}C NMR of **68** in dioxane (red, bottom) vs **68** + 0.25 equiv. of trispentafluorophenyl borane and 2.0 equiv. of trimethoxysilyl chloride (teal, top) full spectrum.

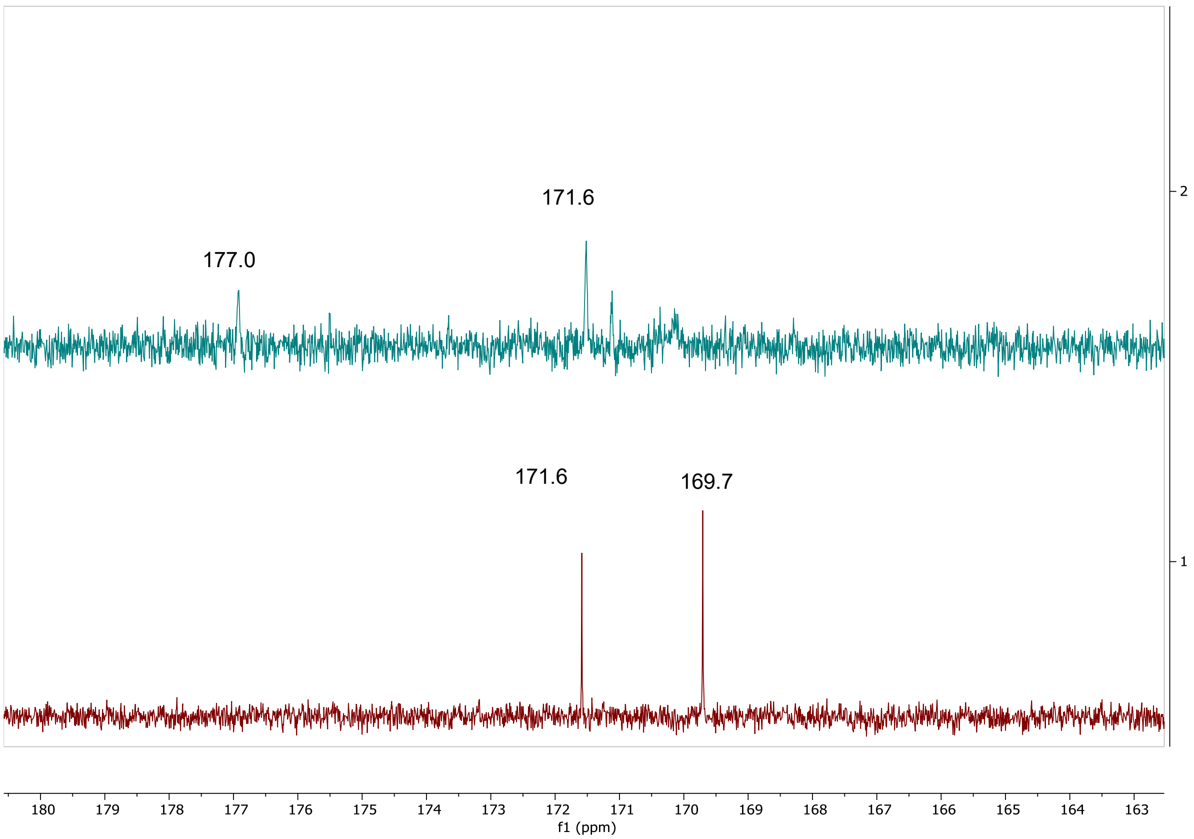

Spectrum S21. ^{13}C NMR of **68** in dioxane (red, bottom) vs **68** + 0.25 equiv. of trispentafluorophenyl borane and 2.0 equiv. of trimethoxysilyl chloride (teal, top) carbonyl region.

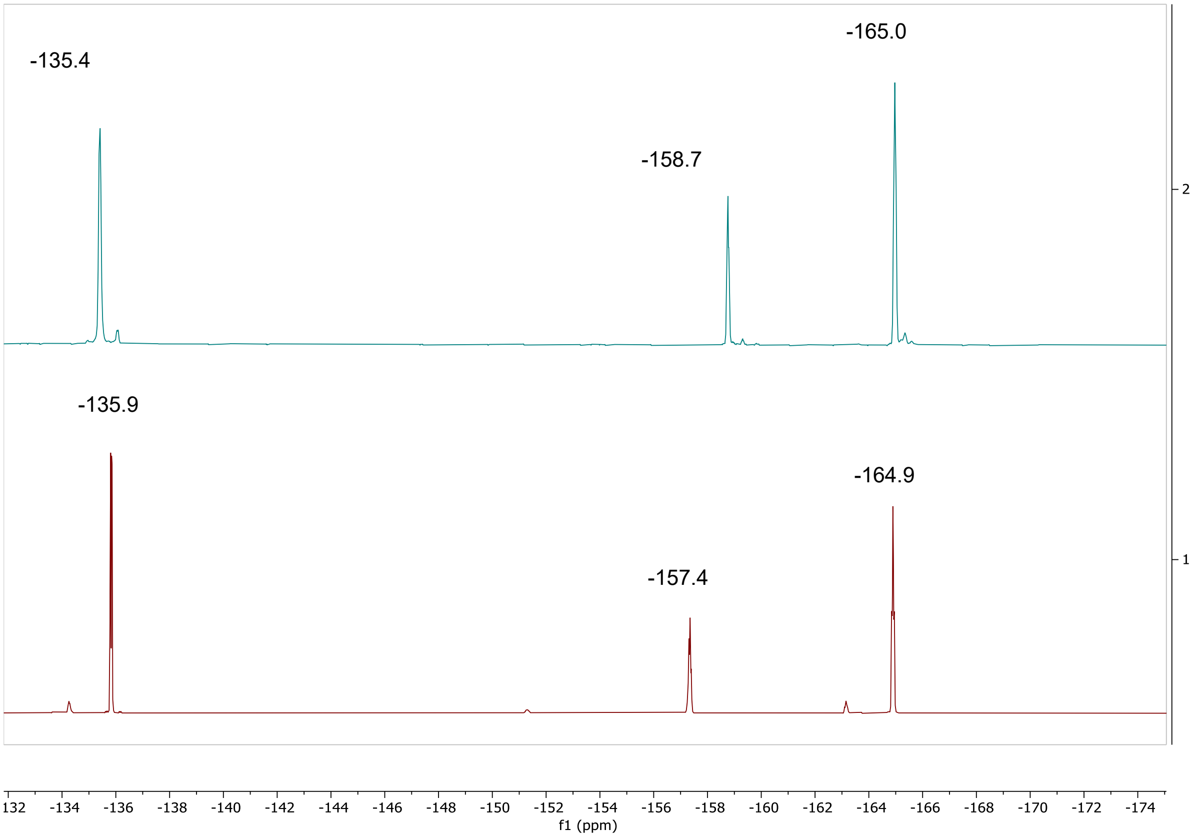

Spectrum S22. ^{19}F NMR of trispentafluorophenyl borane in dioxane (red, bottom) vs **68** + 0.25 equiv. of trispentafluorophenyl borane and 2.0 equiv. of trimethoxysilyl chloride (teal, top) carbonyl region.

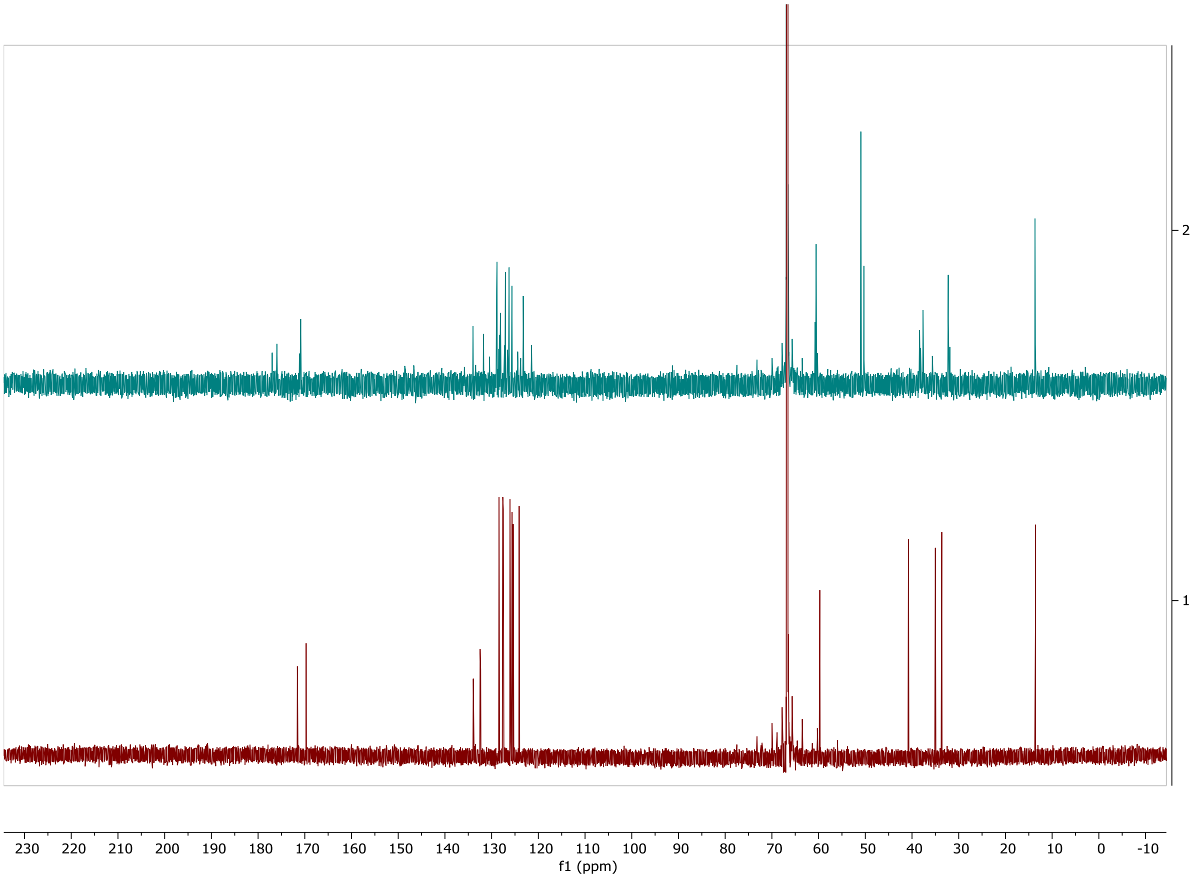

Spectrum S23. ^{13}C NMR of **68** in dioxane (red, bottom) vs **68** + 0.5 equiv. GaI_3 + 0.25 equiv. of trispentafluorophenyl borane and 2.0 equiv. of trimethoxysilyl chloride (teal, top) full spectrum.

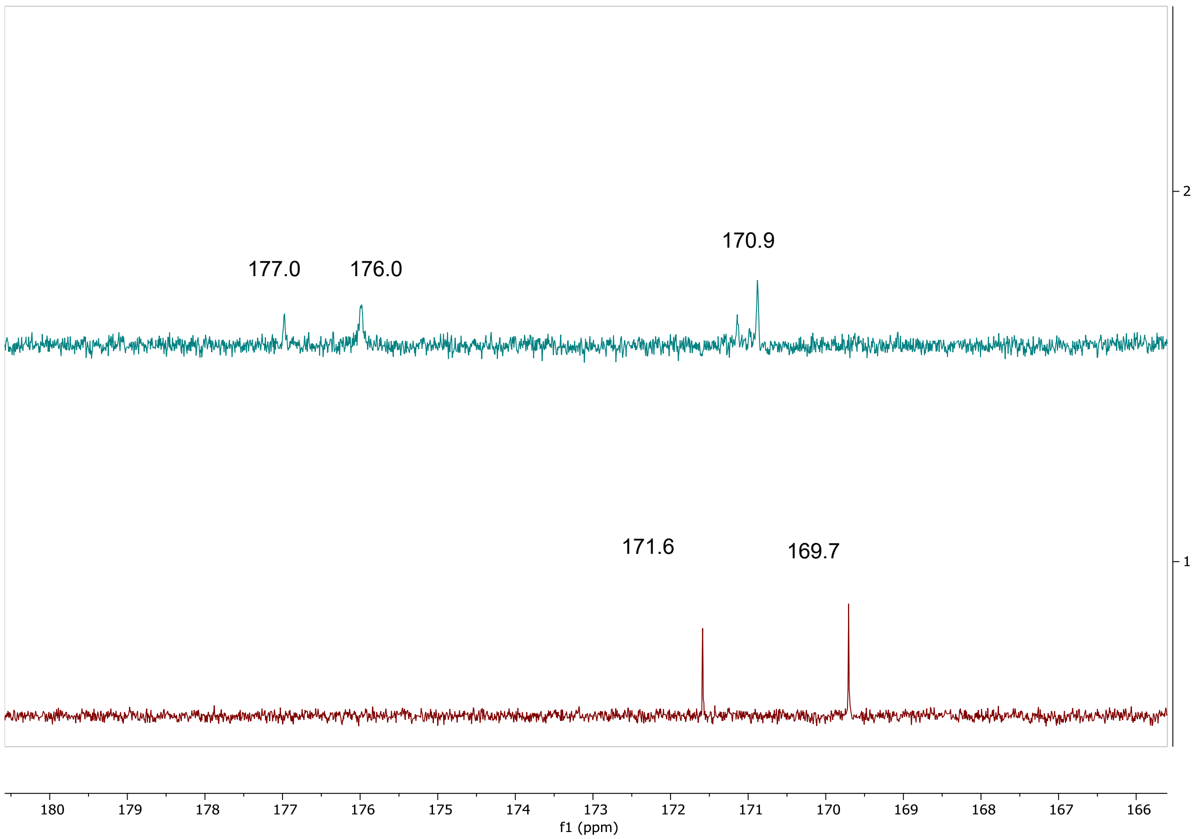

Spectrum S24. ^{13}C NMR of **68** in dioxane (red, bottom) vs **68** + 0.5 equiv. GaI_3 + 0.25 equiv. of trispentafluorophenyl borane and 2.0 equiv. of trimethoxysilyl chloride (teal, top) carbonyl region.

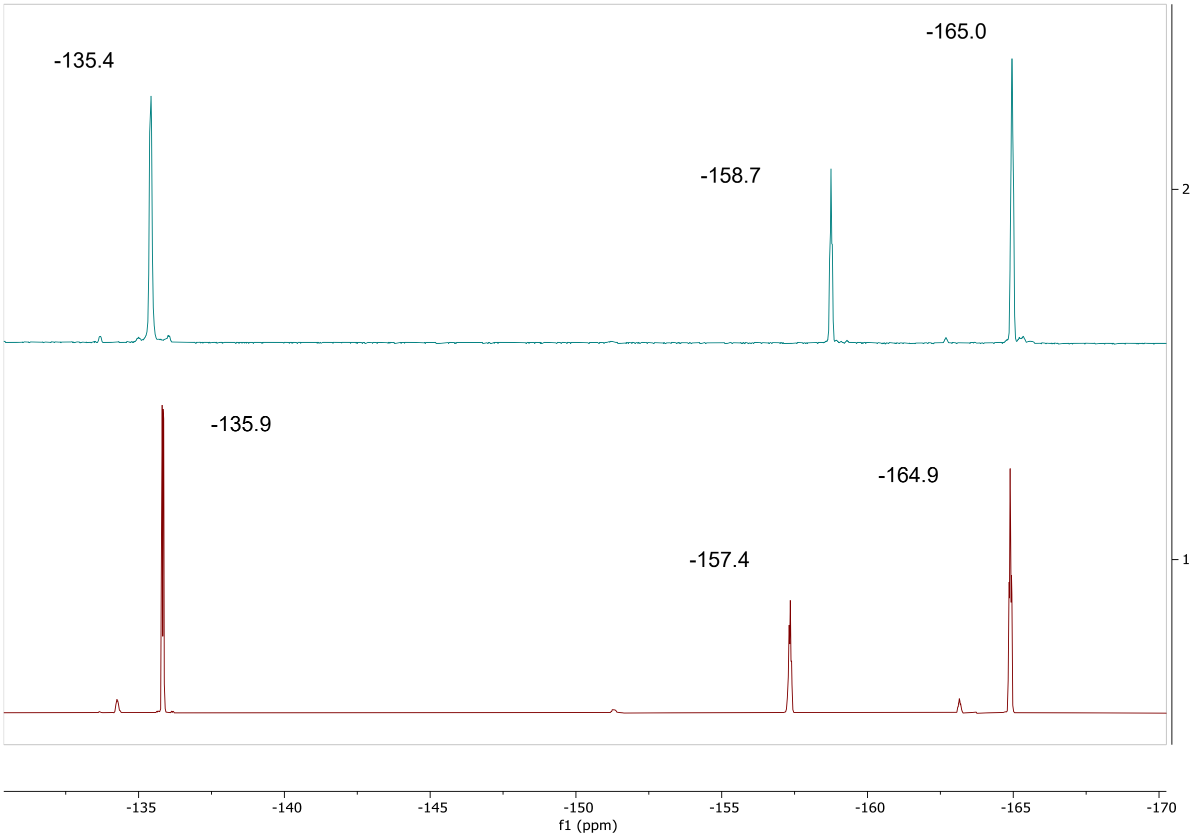

Spectrum S25. ^{19}F NMR of trispentafluorophenyl borane in dioxane (red, bottom) vs **68** + 0.5 equiv. GaI_3 + 0.25 equiv. of trispentafluorophenyl borane and 2.0 equiv. of trimethoxysilyl chloride (teal, top) carbonyl region.

Reagent Control and Order of Addition Experiments

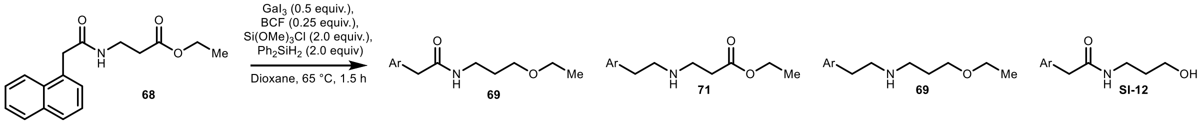

Entry	Order of addition	Reagent Excluded	Additional Additive	P/IS **38**	P/IS **40**	P/IS **39**	P/IS **SI-12**	RSM
1	BCF, Si, then Ga+SiH	None		0.51	0.26	0.034	0.55	1.1
2	Ga, Si then BCF+SiH	None		0.11	0	0	0.6	2.35
3	Ga,BCF the Si+SiH	None		0.44	0.20	0.25	0.93	0.24
4	Ga,BCF,Si then SiH	None		1.89	0	0.38	0.18	0
5	Ga,BCF then SiH	$Si(OMe)_3Cl$		1.21	0.91	0.29	1.18	0.10
6	BCF,Si then SiH	GaI_3		0.48	0.0	0.0	0.34	1.31
7	Ga, Si then SiH	BCF		0	0.78	0.0	0.0	2.99
8	Ga,then SiH	BCF, $Si(OMe)_3Cl$		trace	trace	0	0	2.95
9	BCF then SiH	GaI_3, $Si(OMe)_3Cl$		trace	0.0	0.0	0	2.93
10	Si then SiH	GaI_3, BCF		0	0.00	0.0	0.0	2.99
11	Ga, BCF then SiH	$Si(OMe)_3Cl$	$GaCl_3$ (2 equiv)	1.82	0	0	0.0 4	0

Table S3. Mechanistic study to know the role of each reagent in the reaction.

Mechanistic Experiments

GC-MS Headspace Experiment

Two dried two-dram vials and an 8 mL crimp top microwave vial (part number from Hamid) were brought inside the glovebox was added. In the microwave vial, equipped with a stir bar, **68**(0.20 mmol, 1.0 equiv. was added). This was dissolved in 665 µL of dioxane. In vial **1**, GaI_3 (45 mg, 0.10 mmol 0.5 equiv.) was dissolved in 665 µL of dioxane. In Vial **2**, trispentafluorophenyl borane (25 mg, 0.05 mmol, 0.25 equiv.) was dissolved in 665 µL of dioxane. Vial **1** was added to the microwave vial followed immediately by vial **2**. Trimethoxychlorosilane (56 µL, 0.40 mmol, 2.0 equiv., and diphenylsilane (74 µL, 0.40 mmol, 2.0 equiv.). The vial was crimped shut, removed from the glovebox and stirred at 500 rpm at 65 °C for 90 minutes. At this point the vial was removed from the heat source and placed on a heated (40 °C) GC-MS rack and a sample of the headspace above the reaction taken. After analysis, we observed mass of MeCl and MeI.

Detection of the Side Product

In the glovebox, equipped with a stir bar, **68** (0.20 mmol, 1.0 equiv. was added). This was dissolved in 665 µL of dioxane. In vial **1**, GaI_3 (45 mg, 0.10 mmol 0.5 equiv.) was dissolved in 665 µL of dioxane. In Vial **2**, trispentafluorophenyl borane (25 mg, 0.05 mmol, 0.25 equiv.) was dissolved in 665 µL of dioxane. Vial **1** was added to the microwave vial followed immediately by vial **2**. Trimethoxychlorosilane (56 µL, 0.40 mmol, 2.0 equiv., and diphenylsilane (74 µL, 0.40 mmol, 2.0 equiv.). The vial was crimped shut, removed from the glovebox and stirred at 500 rpm at 65 °C for 90 minutes. Analyzing the reaction mass in UPLC-MS, we observed mass of trimethoxy silyl diphenyl silyl ether [$(OMe)_3SiOSiPh_2H$] which supports the formation of intermediate **76**.

Kinetic Experiments

Halogenation

In the glovebox, an oven dried one dram vial equipped with a stir bar, was charged with amine (**58**) (0.10 mmol,1.0 equiv.), 2,4,6-triphenylpyrylium tetrafluoroborate (**59**) (0.11 mmol, 1.1 equiv.) and KI (0.15 mmol, 1.5 equiv.) and dioxane (0.10 M). The vial was capped, removed from the glovebox, and stirred at 500 rpm at 110 °C. At different time intervals, 10 μL of the reaction mixture was withdrawn, and the conversion was monitored by ¹H NMR spectroscopy using 1,3,5-trimethoxybenzene as an internal standard.

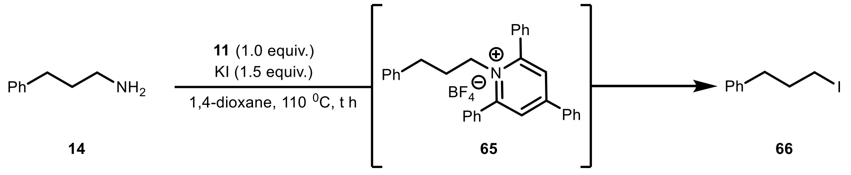

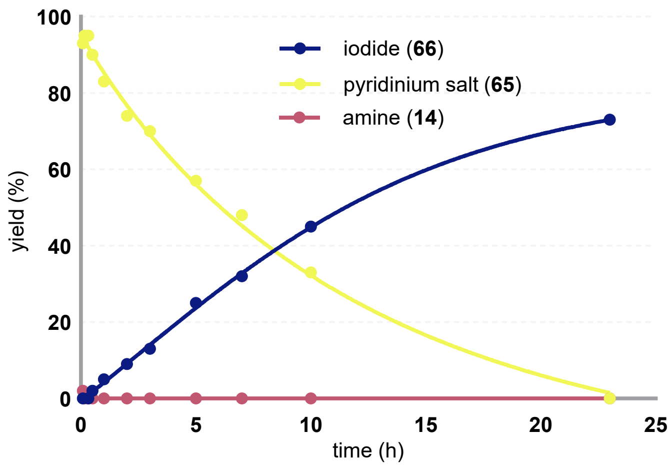

Esterification

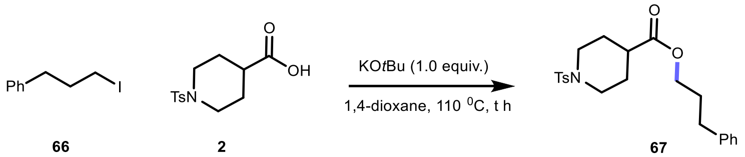

In the glovebox, an oven dried one dram vial equipped with a stir bar, was charged with iodide (**66**) (0.10 mmol,1.0 equiv.), acid (0.10 mmol, 1.0 equiv.) and KO*t*Bu (0.15 mmol, 1.5 equiv.) and dioxane (0.10 M). The vial was capped, removed from the glovebox, and stirred at 500 rpm at 110 °C. At different time intervals, 10 μL of the reaction mixture was withdrawn, and the conversion was monitored by ¹H NMR spectroscopy using 1,3,5-trimethoxybenzene as an internal standard.

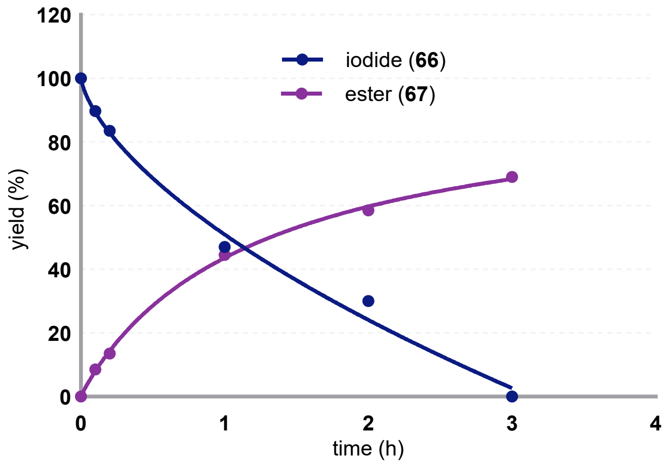

Etherification

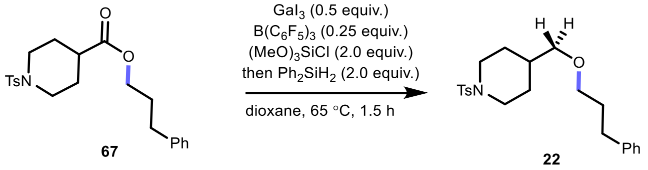

Three dried two-dram vials were brought into the glovebox. In Vial **1** equipped with a stir bar, corresponding ester (0.10 mmol,1.0 equiv.) was added. Dioxane was added (3.33 mL/mmol ester) to vial **1**. GaI_3 (0.5 equiv.) was weighed into a second vial (vial **2**) and dioxane (3.33 mL/mmol ester) added. Tris-(pentafluorophenyl)borane or trimesityl borane (0.25 equiv.) was weighed into a third vial (vial **3**) and dioxane (3.33 mL/mmol ester) added. Vials **2** and **3** were capped and vortexed until each reagent had fully dissolved. Vial **2** was added to vial **1** without stirring followed by vial **3**. Trimethoxychlorosilane (2.0 equiv.) was added to vial **1** followed by diphenylsilane (2.0 equiv.) Upon addition of the silane, the vial was capped and removed from the glovebox. The reaction was stirred at 500 rpm at 65 °C. At different time intervals, 10 µL of the reaction mixture was withdrawn, and the conversion was monitored by ^1H NMR spectroscopy using 1,3,5-trimethoxybenzene as an internal standard.

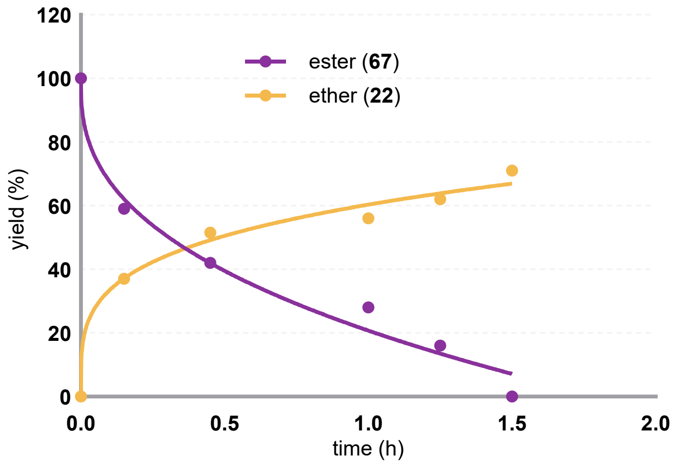

Discovery of a Platinum catalyzed Reductive Etherification

The initial discovery of this reaction was via platinum-based catalysis from the pyridinium salt and acid. These conditions were discovered to work for benzylic pyridinium salts, and somewhat for primary, however the structure reactivity relationship was discovered to be quite flat and optimization attempts were abandoned in favor of the gallium-based conditions.

Screen examining 6 Lewis acids, 2 silanes, +/- trifluoracetic acid

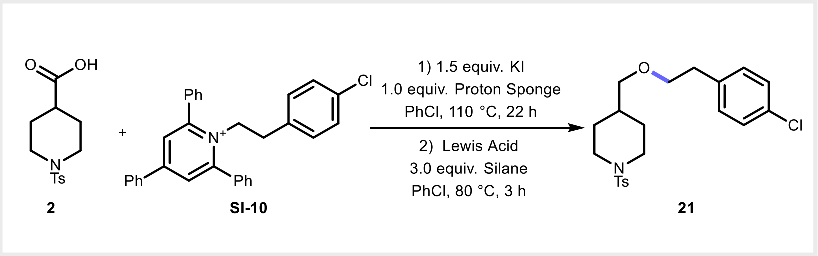

name	molarity	color
INDIUM(III) BROMIDE	0.015	#b83289
SCANDIUM(III) TRIFLATE	0.015	#5502a4
ALUMINUM CHLORIDE	0.015	#8405a7
TRIS(PENTAFLUOROPHENYL)BORANE\	0.015	#48039f
PLATINUM(II) CHLORIDE	0.015	#ce4b75
TETRABUTYLAMMONIUM FLUORIDE HYDRATE	1.5	#b83289
TFA blank	0.1	#41049d
Trifluoroacetic Acid	0.15	#99159f
triethylsilane	0.3	#de6164
Phenylsilane	0.3	#8e0ca4
Ester solution	0.1	#f68d45

Reagents	Solvent	Cstock (M)	Vdose (μL)	Wells	Order Added
Indium (III) Bromide	Chlorobenzene	0.2	25	A,B,C,D 1	1
Scandium (III) Triflate	Chlorobenzene	0.2	25	A,B,C,D 2	1
Aluminum Chloride	Chlorobenzene	0.2	25	A,B,C,D 3	1
trispentafluorophenylborane	Chlorobenzene	0.2	25	A,B,C,D 4	1
platinum (II) chloride	Chlorobenzene	0.05	25	A,B,C,D 5	1
tetrabutyl ammonium fluoride	Chlorobenzene	0.3	25	A,B,C,D 6	1
Acid blank	Chlorobenzene	0.3	25	A,C 1-6	2
trifluoro acetic acid	Chlorobenzene	0.3	25	B,D 1-6	2
triethylsilane	Chlorobenzene	0.9	25	A,B 1-6	3
phenylsilane	Chlorobenzene	0.6	25	C,D 1-6	3
Ester solution	Chlorobenzene	0.3	25	All	4

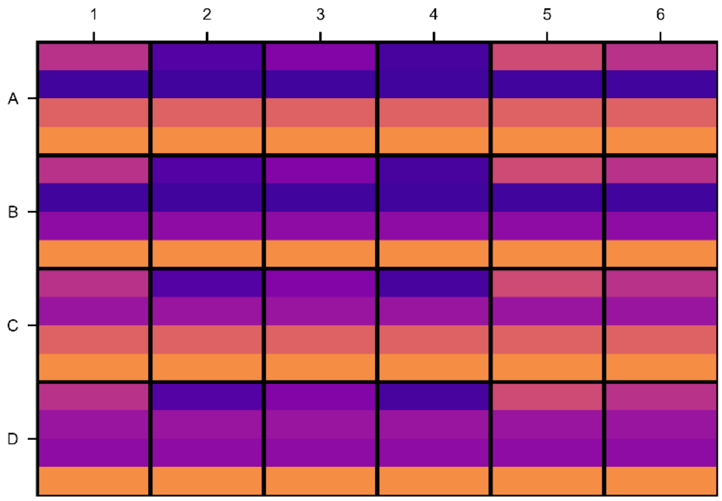

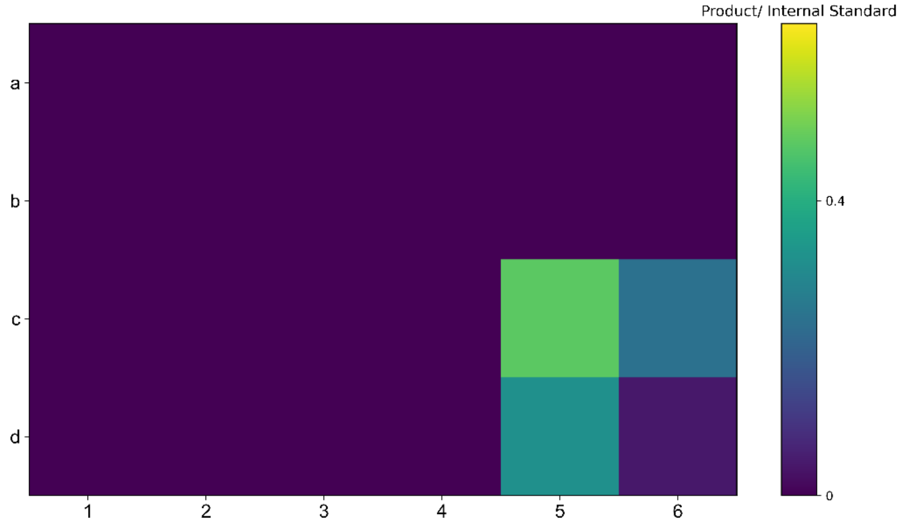

Figure S8. Results of screen examining 6 Lewis acids, 2 silanes, +/- trifluoracetic acid. Platinum chloride and tetrabutylammonium fluoride (TBAF) produced ether product in combination with phenylsilane.

Remote access ligand and silane screen for platinum catalyzed reductive etherification.

This screen was conducted when limitations on the number of people allowed in lab were in place during COVID. To continue research, we conducted a remote access screen where one person was in lab to load the robot with the necessary reagents and the Opentrons was controlled remotely from a laptop.

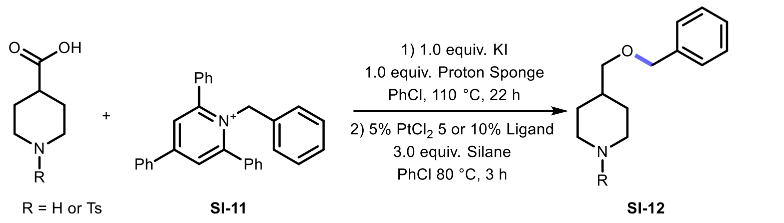

name	molarity	color
N-tosyl isonipectoic acid	0.1	#9511a1
isonipecotic acid	0.1	#9613a1
benzyl kat salt	0.1	#fdab33
PLATINUM(II) CHLORIDE, 99.99+%	0.01	#fbd724
DPPF	0.01	#5002a2
1,2-BIS(DICYCLOHEXYLPHOSPHINO)ETHANE, 98%	0.01	#ca457a
1,2-BIS(DIPHENYLPHOSPHINO)BENZENE 98%	0.01	#8707a6
(R)-BINAP	0.01	#4b03a1
BrettPhos	0.01	#fba238
2-DI-TERT-BUTYLPHOSPHINO-2',4',6'-TRIISOPROPYLBIPHENYL, 97%	0.01	#5c01a6
2-DICYCLOHEXYLPHOSPHINO-2',4',6'-TRIISOPROPYLBIPHENYL, 97%	0.01	#eb7655
ICYCLOHEXYLPHOSPHINO-2',6'-DIISOPROPOXYBIPHENYL, 95% [RUPHOS]	0.01	#c43e7f
AlPhos	0.01	#e4695e
CyJohnPhos	0.01	#c33d80
4,6-BIS(DIPHENYLPHOSPHINO)PHENOXAZINE, 97%	0.01	#48039f
LPHOSPHINO)3,6-DIMETHOXY-2',4',6'-TRIISOPROPYL-1,1'-BIPHENYL, 96%	0.01	#9a169f
(2-BIPHENYLYL)DI-TERT-BUTYLPHOSPHINE, 97%	0.01	#e76f5a
TRI-O-TOLYLPHOSPHINE	0.01	#f2844b
TRI-TERT-BUTYLPHOSPHINE, 98%	0.01	#d14e72
TRIPHENYLPHOSPHINE, 99%	0.01	#e26660
TRIS(4-TRIFLUOROMETHYLPHENYL)PHOSPHINE, 97%	0.01	#fa9c3c
1,3-BIS(DIPHENYLPHOSPHINO)PROPANE, 98.0+%	0.01	#fcd225
ligand blank	0.1	#fdc229
XANTPHOS, 97%	0.01	#f1814d
(OXYDI-2,1-PHENYLENE)BIS(DIPHENYLPHOSPHINE), 98%	0.01	#fec029
CATACXIUM A, 95%	0.01	#fcd225
Me4tButylXphos	0.01	#d9586a
ligand blank 2	0.1	#feba2c
Diphenylsilane	0.3	#d24f71
Phenylsilane, 97+%, Thermo Scientificâ€šÃ'Â¢	0.3	#3e049c

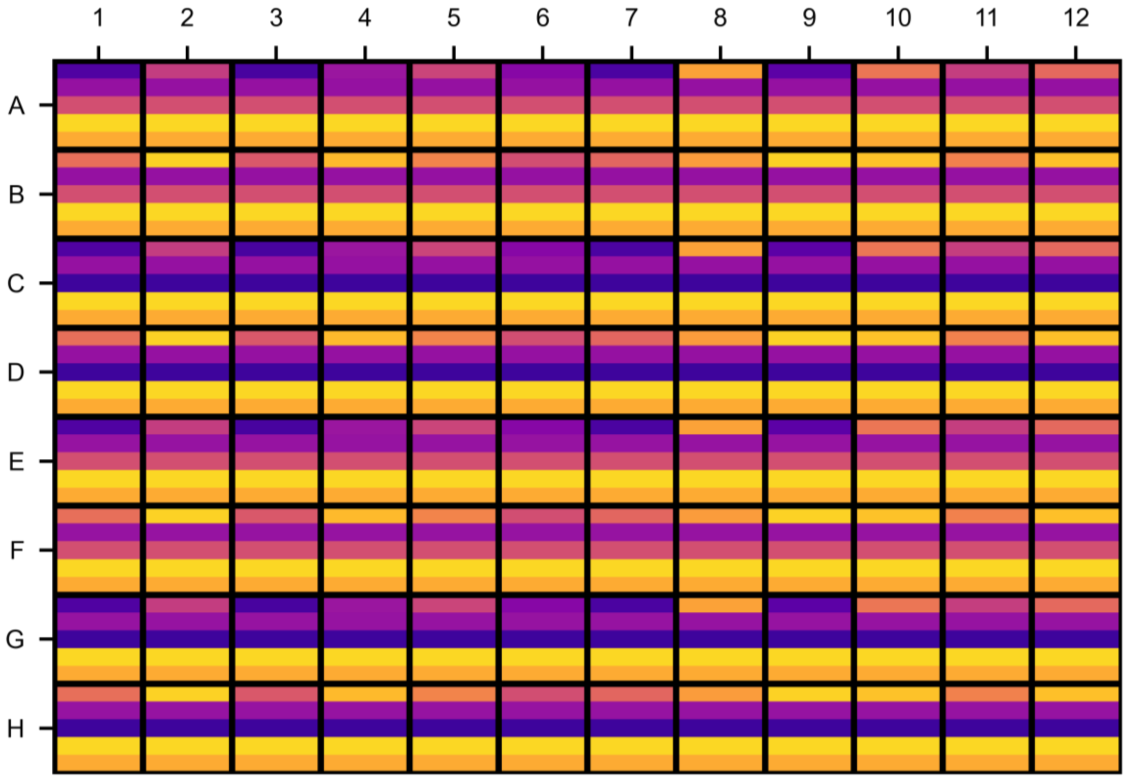
1
2
3
4
5
6
7
8
9
10
11
12
A
B
C
D
E
F
G
H

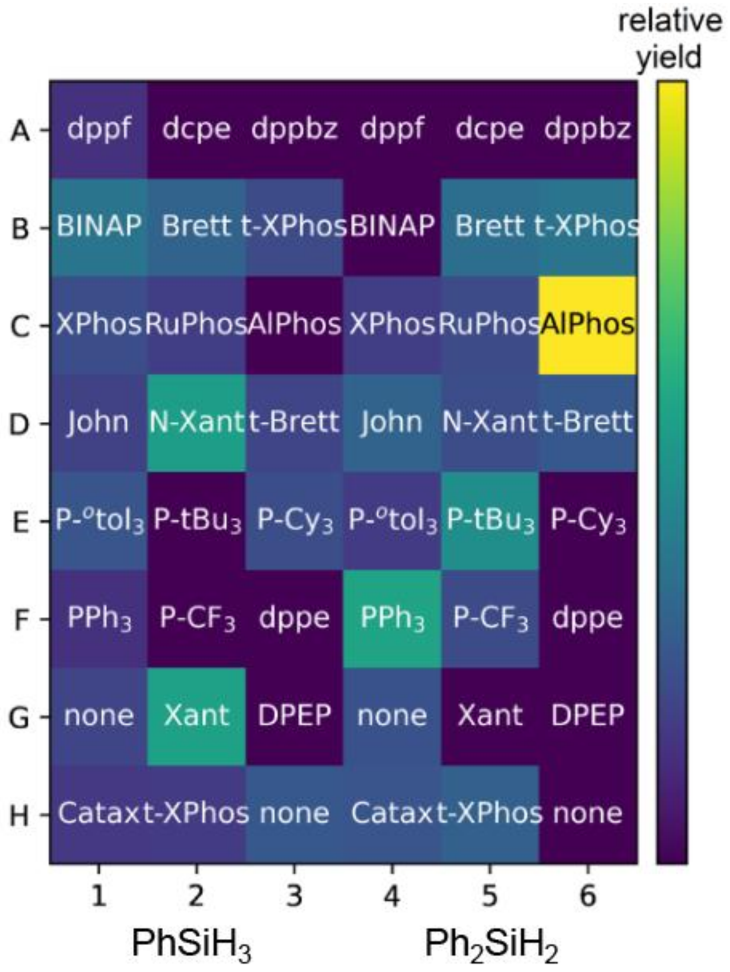

Figure S9. Results of remote access ligand/silane screen. Please note only product SI-15 was observed so only half of the heatmap is displayed.

Scale up of remote access screen

Upon scaleup of the best results from the screen it was revealed the major product was enol ether which has an M-2 mass. Subsequent attempts to reduce this to **SI-15** failed.

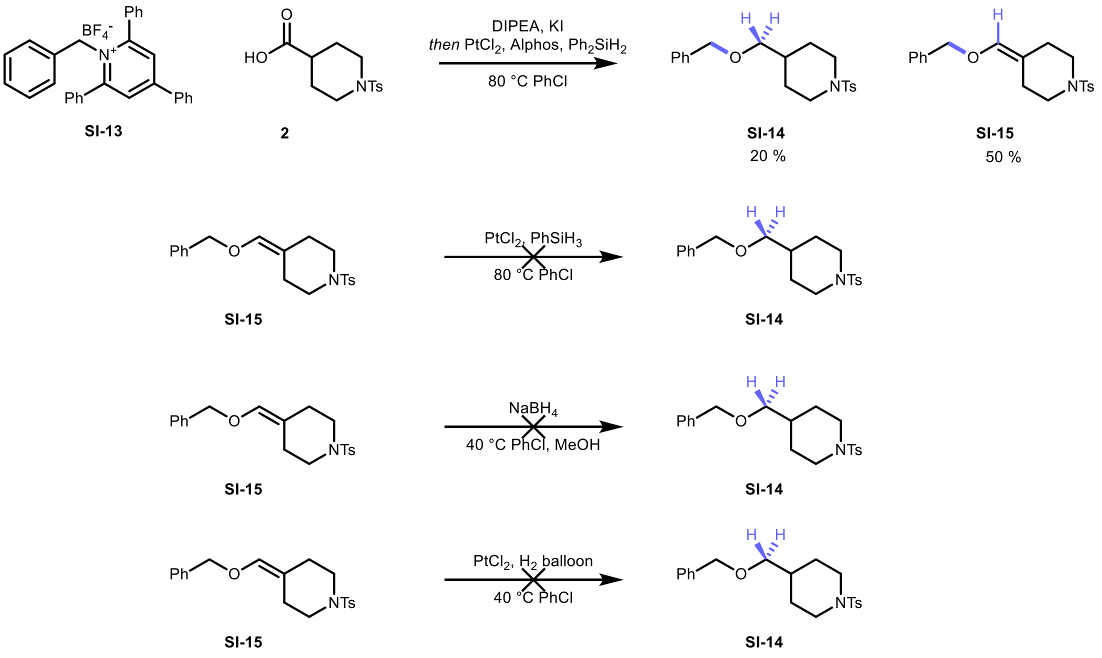

Scheme S2. Results of remote access scaleup and subsequent attempts to reduce enol ether.

Extended Platinum Catalyzed Etherification Optimization

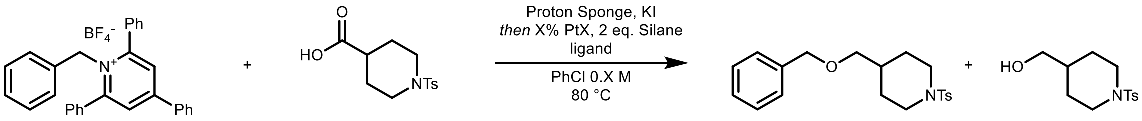

entry	Platinum Salt	Silane	Ligand	Concentration	NMR yield ether (isolated)	NMR yield alcohol
1	$PtCl_2$ (10%)	$PhSiH_3$	None	0.3	61%	35%
2	PtO_2 (10%)	$PhSiH_3$	None	0.3	55%	33%
3	Pt(COD)Me_2 (10%)	$PhSiH_3$	None	0.3	62%	34%
4	$PtCl_2$ (20%)	$PhSiH_3$	None	0.3	42%	52%
5	$PtCl_2$ (1%)	$PhSiH_3$	None	0.3	32%	26%
6	$PtCl_2$ (3%)	$PhSiH_3$	None	0.3	62%	35%
7	$PtCl_2$ (3%)	$PhSiH_3$	PPh_3	0.3	20%	73%
8	$PtCl_2$ (3%)	Ph_2SiH_2	None	0.3	21%	10%
9	$PtCl_2$ (3%)	Ph_2SiH_2	PPh_3	0.3	33%	12%
10	$PtCl_2$ (3%)	TMDS	None	0.3	trace	trace
11	$PtCl_2$ (3%)	$PhSiH_3$	None	0.1	40%	48%
12	$PtCl_2$ (3%)	$PhSiH_3$	PPh_3	0.5	55%	37%

Table S4. Optimizing platinum source, loading, ligand, silane, and concentration.

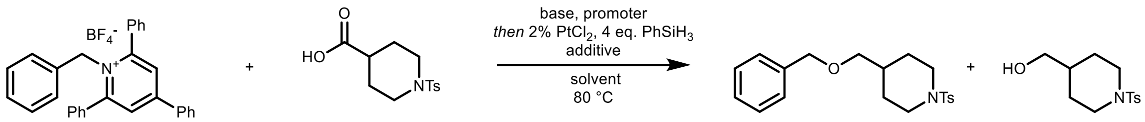

entry	base	Promoter	solvent	additive	NMR yield ether (isolated)	NMR yield alcohol
1	Proton Sponge	KI	PhCl	None	61% (59%)	35%
2	Proton Sponge	BMDM	PhCl	None	Trace	Trace
3	Proton Sponge	KI	DMF	None	0%	0%
4	DIPEA	KI	PhCl	None	61% (60%)	35%
5	DIPEA	KI	PhCl	Water 2 eq	58%	38%
6	DIPEA	KI	PhCl	TMSOTf	59%	37%
7	DIPEA	KI	PhCl	TBAF	40%	54%
8	DIPEA	KI	Dioxane	None	40%	48%
9	DIPEA	KI	Diglyme	None	30%	60%
10	DIPEA	KI	$PhCF_3$	None	65%	37%

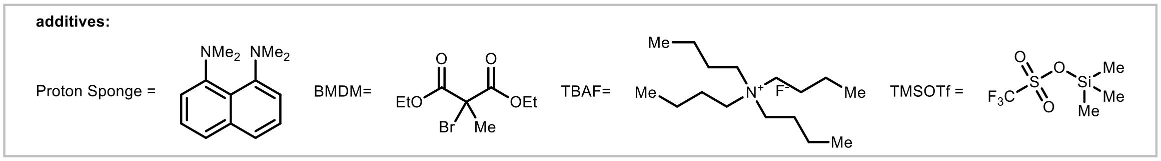

Table S5. Optimization of ester promoter, base, and solvent as well as investigating the effects of additives.

Platinum vs Gallium Comparison

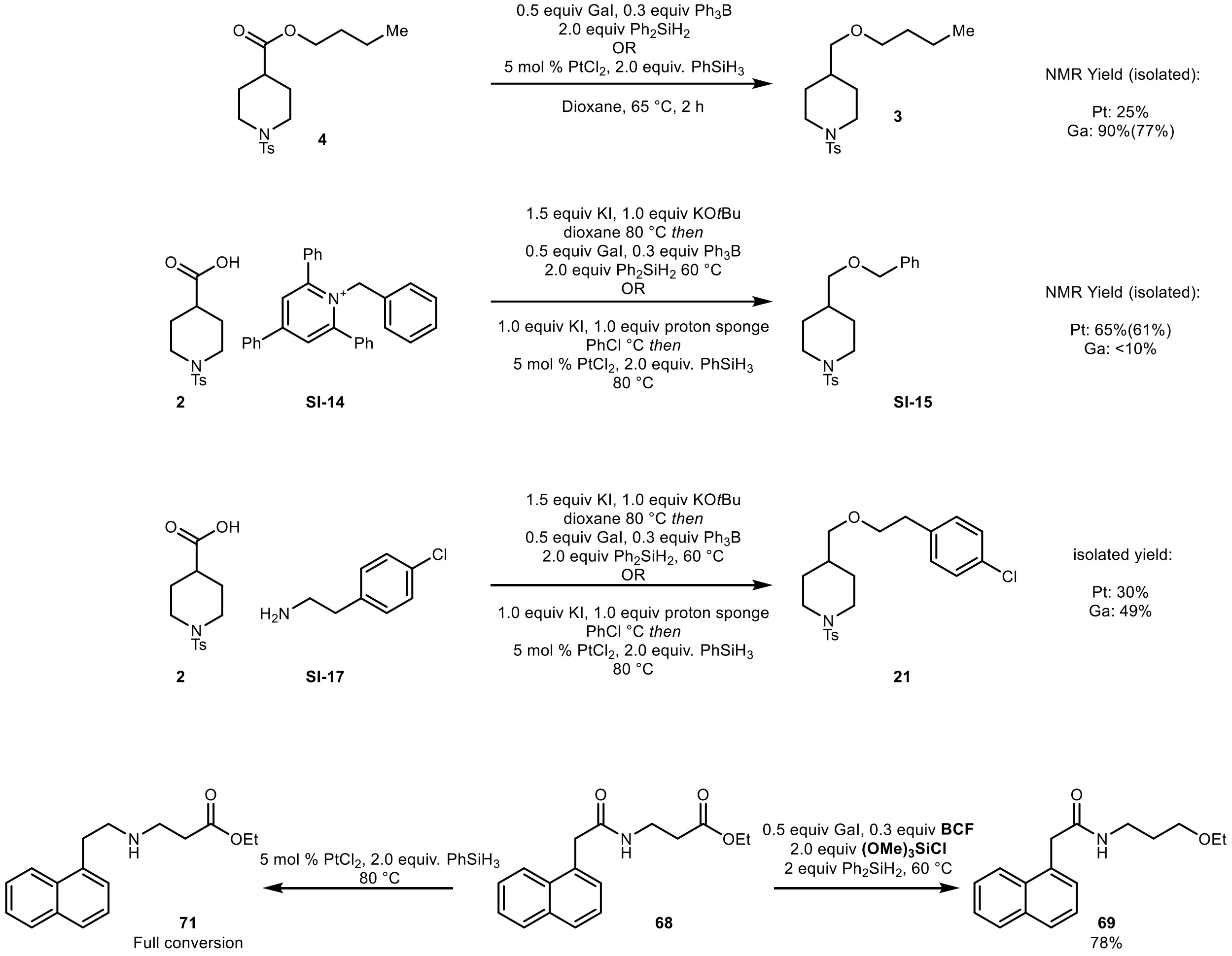

Scheme S3. Comparison of Gallium and Platinum based reduction conditions. Gallium is selective for esters and outperforms platinum in non-benzylic esters.

Comparison of Our Developed Reductive Etherification Method with Previously Reported Etherification Methods

From Isolated Ester

A

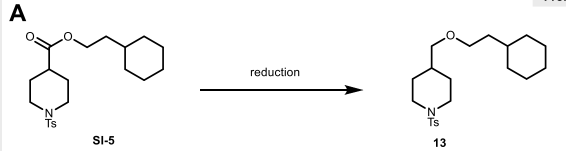

conditions	NMR yield of ether (13)
**a.** 50 mol% GaI_3, 25 mol% $B(C_6F_5)_3$, 2.0 equiv. $(MeO)_3SiCl$, 2.0 equiv. $PhSiH_3$, dioxane, 65 °C, 1.5 h. (our conditions)	>99%
**b.** 1 mol% $GaBr_3$, 1.1 equiv. TMDS, 60 °C, 1 h (Biermann conditions)	84%
**c.** 5 mol% $InBr_3$, 4.0 equiv. Et_3SiH, $CHCl_3$, 60 °C, 1 h. (Sakai conditions)	65%

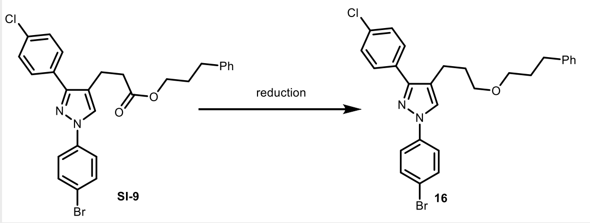

conditions	NMR yield of ether (16)
**a.** 50 mol% GaI_3, 25 mol% $B(C_6F_5)_3$, 2.0 equiv. $(MeO)_3SiCl$, 2.0 equiv. $PhSiH_3$, dioxane, 65 °C, 1.5 h. (Our conditions)	94%
**b.**1 mol% $GaBr_3$, 1.1 equiv. TMDS, 60 °C, 1 h (Biermann conditions)	21%
**c.** 5 mol% $InBr_3$, 4.0 equiv. Et_3SiH, $CHCl_3$, 60 °C, 1 h. (Sakai conditions)	trace

From Free amine and Acid

B

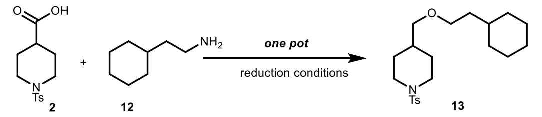

conditions	NMR yield of ether (13)
**a.** 3.0 equiv.GaI_3, 0.3 equiv.$(C_6F_5)_3B$, 2.0 equiv.$(OMe)_3SiCl$, 3.0 equiv.$PhSiH_3$, dioxane, 65 °C, 1.5 h (**Our conditions**)	73%
**b.** 1.0 mol% $GaBr_3$, 1.1 equiv. TMDS, 60 °C, 1 h. (**Biemann conditions**)	0%
**c.** 5.0 mol% $InBr_3$, 4.0 equiv. Et_3SiH, $CHCl_3$, 60 °C, 1 h. (**Sakai conditions**)	0%
**d.** 3.0 equiv. $GaBr_3$, 3.0 equiv. TMDS, dioxane, 60 °C, 1 h	49%
**e.** 3.0 equiv. $InBr_3$, 4.0 equiv. Et_3SiH, dioxane, 60 °C, 1 h	0%

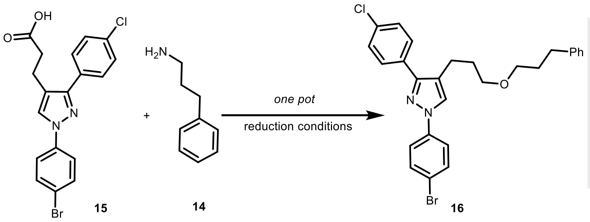

conditions	NMR yield of ether (16)
**a.** 3.0 equiv.GaI_3, 0.3 equiv.$(C_6F_5)_3B$, 2.0 equiv.$(OMe)_3SiCl$, 3.0 equiv.$PhSiH_3$, dioxane, 65 °C, 1.5 h (**Our conditions**)	51%
**b.** 1.0 mol% $GaBr_3$, 1.1 equiv. TMDS, 60 °C, 1 h. (**Biermann conditions**)	0%
**c.** 5.0 mol% $InBr_3$, 4.0 equiv. Et_3SiH, $CHCl_3$, 60 °C, 1 h. (**Sakai conditions**)	0%
**d.** 3.0 equiv. $GaBr_3$, 3.0 equiv. TMDS, dioxane, 60 °C, 1 h	23%
**e.** 3.0 equiv. $InBr_3$, 4.0 equiv. Et_3SiH, dioxane, 60 °C, 1 h	0%

Selecitivity Comparison

C

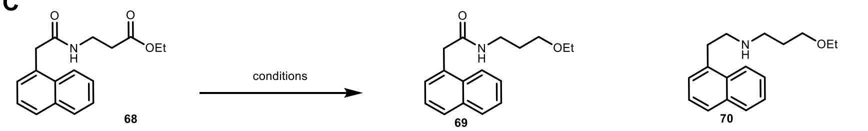

crude NMR analysis

	69:70
**conditions a**	79%:17%
**conditions b**	6%:17%
**conditions c**	nd (<5% conv.)

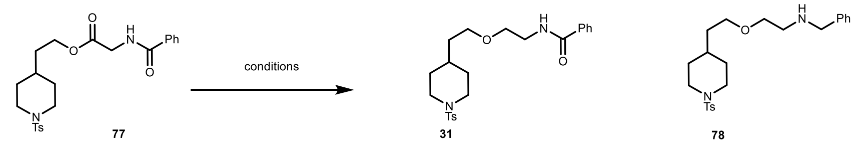

crude NMR analysis

	31:78
**conditions a**	70%:18%
**conditions b**	0%
**conditions c**	0%

Scheme S4. Comparison of our reductive etherification method with previously reported ester to ether reduction methods.

Deaminative Amine–Phenol Etherification Optimization

For entries **1-12** all reagents were added in a single step to try and perform a single step operation. This was met with some success on simpler substrates, but on the more complex drug molecules, yields were unacceptable as a single step operation, and a two-step protocol was developed (entries 13-16)

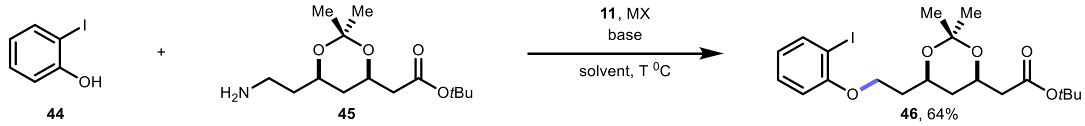

entry	MX	Base	Solvent	Temperature (°C)	NMR yield ether
1	KI	K_2CO_3	Dioxane	110	40%
2		K_2CO_3	DMA	110	15%
3	KBr	K_2CO_3	Dioxane	110	35%
4	KBr	K_2CO_3	DMA	110	14%
5	CsI	K_2CO_3	Dioxane	110	37%
6	CsI	K_2CO_3	DMA	110	18%
7	KI	KOtBu	Dioxane	110	12%
8	KI	LiOtBu	Dioxane	110	10%
9	KI	Cs_2CO_3	Dioxane	110	15%
10	KI	KOtBu	DMA	110	trace
11	KI	LiOtBu	DMA	110	trace
12	KI	Cs_2CO_3	DMA	110	11%
13	KI	K_2CO_3	Dioxane	110 *then* 80	45%
14	KI	K_2CO_3	DMA	110 *then* 80	50%
15	KI	K_2CO_3	1:1 Dioxane:DMA	110 *then* 80	50%
16	KI	K_2CO_3	Dioxane *then* DMA	110 *then* 80	64%

Table S6. Optimization of a two-step deaminative phenolic etherification protocol.

Products Characterization

.

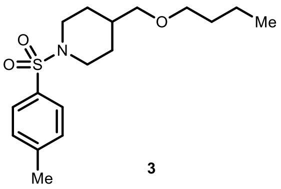

3

3 was prepared on a 0.2 mmol scale from butyl amine and **2** according to general procedure **B** to give 41.9 mg (63%) of a white solid after purification with EtOAc/hexanes.

R_f = 0.32 in 25:75 EtOAc:Hexane

^{1}H NMR (499 MHz, $CDCl_3$) δ 7.64 (d, *J* = 8.3 Hz, 2H), 7.31 (d, *J* = 8.5 Hz, 2H), 3.82 – 3.74 (m, 2H), 3.35 (t, *J* = 6.6 Hz, 2H), 3.20 (d, *J* = 6.5 Hz, 2H), 2.43 (s, 3H), 2.23 (td, *J* = 11.9, 2.6 Hz, 2H), 1.77 (dd, *J* = 13.7, 3.6 Hz, 2H), 1.55 – 1.46 (m, 3H), 1.38 – 1.27 (m, 4H), 0.89 (t, *J* = 7.4 Hz, 3H).

^{13}C NMR (126 MHz, $CDCl_3$) δ 143.5, 134.5, 129.7, 127.9, 77.4, 77.2, 76.9, 75.3, 71.1, 46.3, 35.9, 31.9, 28.7, 21.7, 19.5, 14.0.

HRMS (ESI) Calculated $C_{17}H_{28}NO_3S^+$ $[M+H]^+$: 326.1784, Found 326.1777.

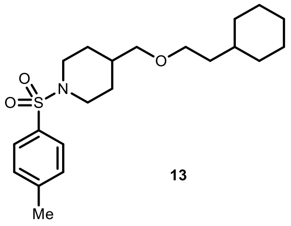

13

13 was prepared on a 0.2 mmol scale from 2-cyclohexylethylamine and **2** according to general procedure **B** to give 41.0 mg (55%) of a white solid after purification with EtOAc/hexane.

R_f = 0.25 in 25:75 EtOAc:Hexane

^{1}H NMR (401 MHz, $CDCl_3$) δ 7.64 (d, *J* = 8.2 Hz, 2H), 7.31 (d, *J* = 8.0 Hz, 2H), 3.78 (d, *J* = 11.6 Hz, 2H), 3.38 (t, *J* = 6.7 Hz, 2H), 3.19 (d, *J* = 6.5 Hz, 2H), 2.43 (s, 3H), 2.23 (td, *J* = 11.9, 2.2 Hz, 2H), 1.76 (d, *J* = 11.2 Hz, 2H), 1.66 (d, *J* = 10.0 Hz, 4H), 1.55 – 1.45 (m, 1H), 1.41 (q, *J* = 6.8 Hz, 2H), 1.33 (tt, *J* = 11.5, 5.9 Hz, 3H), 1.19 (dt, *J* = 19.4, 11.0 Hz, 4H), 0.97 – 0.77 (m, 3H).

^{13}C NMR (101 MHz, CDC_3) δ 143.3, 133.4, 129.5, 127.7, 77.3, 77.0, 76.7, 75.2, 69.2, 46.1, 37.0, 35.7, 34.7, 33.3, 28.6, 26.6, 26.3, 21.5.

HRMS (ESI) Calculated $C_{21}H_{33}NO_3S$ $[M+H]^+$: 380.2259 Found 380.2254.

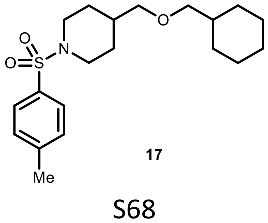

17

17 was prepared on a 0.2 mmol scale from cyclohexylmethylamine and **2** according to general procedure **B** to give 34.8 mg (48%) of a clear oil after purification with EtOAc/hexanes.

R_f = 0.30 in 25:75 EtOAc:Hexane

^{1}H NMR (499 MHz, $CDCl_3$) δ 7.64 (d, *J* = 8.3 Hz, 2H), 7.31 (d, *J* = 7.9 Hz, 2H), 3.78 (dt, *J* = 11.5, 2.2 Hz, 2H), 3.19 (d, *J* = 6.4 Hz, 2H), 3.14 (d, *J* = 6.5 Hz, 2H), 2.43 (s, 3H), 2.23 (td, *J* = 11.9, 2.6 Hz, 2H), 1.76 (dd, *J* = 13.4, 3.4 Hz, 2H), 1.72 – 1.61 (m, 6H), 1.57 – 1.43 (m, 1H), 1.33 (qd, *J* = 12.0, 4.2 Hz, 2H), 1.28 – 1.06 (m, 3H), 0.93 – 0.82 (m, 2H).

^{13}C NMR (126 MHz, $CDCl_3$) δ 143.5, 134.5, 129.7, 127.9, 75.4, 46.3, 38.2, 35.8, 30.2, 28.7, 26.76, 26.0, 21.7.

HRMS (ESI) Calculated $C_{20}H_{32}NO_3S^+$ [M+H] $^+$: 366.2097, Found 366.2097.

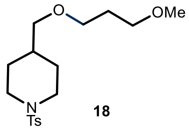

18

18 was prepared on a 0.20 mmol scale according to general procedure **B** from **2** (56.7 mg, 0.20 mmol, 1.0 equiv.) and 3-Methoxypropylamine (20.6 µL, 1.0 equiv.). Isolated in 47% yield (32.1 mg) as colorless liquid after purification by column chromatography (10-30% ethyl acetate in hexane).

^{1}H NMR (401 MHz, $CDCl_3$) δ 7.63 (d, J = 8.4 Hz, 2H), 7.31 (d, J = 8.0 Hz, 2H), 3.79-3.74 (m, 2H), 3.42 (q, J = 6.4 Hz, 4H), 3.31 (s, 3H), 3.21 (d, J = 6.4 Hz, 2H), 2.43 (s, 3H), 2.23 (td, J = 12.0, 2.4 Hz, 2H), 1.82 – 1.74 (m, 4H), 1.53-1.44 (m, 1H), 1.38-1.28 (m, 2H).

^{13}C NMR (101 MHz, $CDCl_3$) δ 143.3, 133.4, 129.5, 127.7, 75.2, 69.6, 68.0, 58.6, 46.1, 35.7, 30.0, 28.5, 21.5.

HRMS (ESI) Calculated $C_{17}H_{28}NO_4S$ [M+H] +: 342.1739, Found 342.1729.

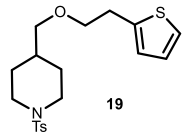

19

19 was prepared on a 0.05 mmol scale from **SI-4** according to general procedure **C** using trimesityl borane to give 12.8 mg (68%) of a yellow oil after purification with EtOAc/hexanes.

Rf = 0.25 in 30:70 EtOAc: Hexane

^{1}H NMR (499 MHz, $CDCl_3$) δ 7.64 (d, *J* = 8.3 Hz, 2H), 7.32 (d, *J* = 8.0 Hz, 2H), 7.12 (dd, *J* = 5.2, 1.2 Hz, 1H), 6.91 (dd, *J* = 5.1, 3.4 Hz, 1H), 6.81 (dd, *J* = 3.4, 1.1 Hz, 1H), 3.78 (dt, *J* = 11.3, 2.9 Hz, 2H), 3.60 (t, *J* = 6.6 Hz, 2H), 3.26 (d, *J* = 6.4 Hz, 2H), 3.04 (td, *J* = 6.6, 0.9 Hz, 2H), 2.43 (s, 3H), 2.23 (td, *J* = 11.9, 2.6 Hz, 2H), 1.77 (dd, *J* = 13.7, 3.7 Hz, 2H), 1.56 – 1.45 (m, 1H), 1.34 (qd, *J* = 11.9, 4.2 Hz, 2H).

^{13}C NMR (126 MHz, $CDCl_3$) δ 143.4, 141.3, 134.3, 129.6, 127.7, 126.6, 125.1, 123.7, 75.3, 71.6, 46.1, 35.7, 30.4, 28.5, 21.5.

HRMS (ESI) Calculated $C_{19}H_{25}NO_3S_2$+ [M+H] +: 379.1276, Found 379.1277.

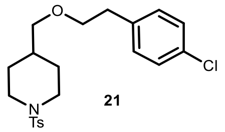

21

21 was prepared on a 0.2 mmol scale from 2-(4-chlorophenyl) ethylamine and **2** according to general procedure **B** to give 40.0 mg (49%) of a white solid after purification with EtOAc/hexanes.

Rf = 0.23 in 25:75 EtOAc: Hexane

^1H NMR (400 MHz, $CDCl_3$) δ 7.63 (d, *J* = 8.3 Hz, 2H), 7.32 (d, *J* = 8.4 Hz, 2H), 7.23 (d, *J* = 8.4 Hz, 2H), 7.11 (d, *J* = 8.4 Hz, 2H), 3.77 (dt, *J* = 11.0, 2.4 Hz, 2H), 3.55 (t, *J* = 6.8 Hz, 2H), 3.21 (d, *J* = 6.4 Hz, 2H), 2.79 (t, *J* = 6.8 Hz, 2H), 2.43 (s, 3H), 2.20 (td, *J* = 11.9, 2.6 Hz, 2H), 1.76 – 1.67 (m, 2H), 1.52 – 1.40 (m, 1H), 1.30 (qd, *J* = 12.4, 4.4 Hz, 2H).

^{13}C NMR (101 MHz, $CDCl_3$) δ 143.5, 137.7, 133.3, 132.1, 130.4, 129.7, 128.5, 127.8, 75.4, 71.81, 46.2, 35.8, 35.7, 28.6, 21.7.

HRMS (ESI) Calculated $C_{21}H_{27}ClNO_3S$+ [M+H] +: 408.1395, Found 408.1392

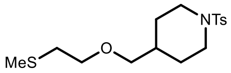

20

20 was prepared on a 0.20 mmol scale according to general procedure **B** from **2** (56.7 mg, 0.20 mmol, 1.0 equiv.) and 3-(Methylthio) propylamine (22.4 µL, 1.0 equiv.). Isolated in 27% yield (19.3 mg) as colorless liquid after purification by column chromatography (10-20% ethyl acetate in hexane).

^1H NMR (401 MHz, $CDCl_3$) δ 7.62 (d, *J* = 8.4 Hz, 2H), 7.30 (d, *J* = 8.0 Hz, 2H), 3.76 (d, *J* = 11.6 Hz, 2H), 3.43 (t, *J* = 6.4 Hz, 2H), 3.20 (d, *J* = 6.4 Hz, 2H), 2.51 (t, *J* = 7.2 Hz, 2H), 2.41 (s, 3H), 2.22 (td, *J* = 12.0, 2.4 Hz, 2H), 2.06 (s, 3H), 1.83-1.73 (m, 4H), 1.52 – 1.48 (m, 1H), 1.36 -1.23 (m, 2H).

^{13}C NMR (101 MHz, $CDCl_3$) δ 143.3, 133.4, 129.5, 127.7, 75.2, 69.4, 46.1, 35.7, 30.9, 29.2, 28.5, 21.5, 15.6.

HRMS (ESI) Calculated $C_{17}H_{28}NO_3S_2$ [M+H] +: 358.1511, Found 358.1505.

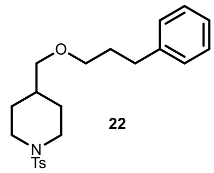

22

22 was prepared on a 0.20 mmol scale according to general procedure **B** from **2** (56.7 mg, 0.20 mmol, 1.0 equiv.) and 3-Phenylpropylamine (28.4 μL, 1.0 equiv.). Isolated in 62% yield (48.1 mg) as colorless liquid after purification by column chromatography (10-20% ethyl acetate in hexane).

^{1}H NMR (401 MHz, $CDCl_3$) δ 7.64 (d, *J* = 8.4 Hz, 2H), 7.31 (d, *J* = 8.0 Hz, 2H), 7.29-7.25 (m, 3H), 7.21-7.17 (m, 3H), 3.77 (d, *J* = 11.6 Hz, 2H), 3.58 (t, *J* = 7.2 Hz, 2H), 3.23 (d, *J* = 6.4 Hz, 2H), 2.83 (t, *J* = 7.2 Hz, 2H), 2.43 (s, 3H), 2.22 (td, *J* = 12.0, 2.4 Hz, 2H), 1.73 (d, *J* = 11.2 Hz, 2H), 1.51 – 1.44 (m, 2H), 1.36 – 1.26 (m, 4H).

^{13}C NMR (101 MHz, $CDCl_3$) δ 143.3, 142.0, 129.6, 128.4,128.3, 127.7, 125.8, 75.1, 70.2, 46.1, 35.8, 32.3, 31.2, 28.6, 21.5.

HRMS (ESI) Calculated $C_{22}H_{30}NO_3S$ $[M+H]^+$: 388.1946, Found 388.1939.

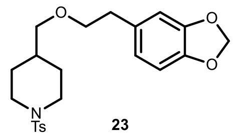

23

23 was prepared on a 0.20 mmol scale according to general procedure **B** from **2** (56.7 mg, 0.20 mmol, 1.0 equiv.) and 3,4-Methylenedioxyphenethylamine hydrochloride (40.3 mg, 1.0 equiv.). Isolated in 51% yield (42.6 mg) as colourless liquid after purification by column chromatography (10-30% ethyl acetate in hexane).

^{1}H NMR (401 MHz, $CDCl_3$) δ 7.64 (d, *J* = 8.4 Hz, 2H), 7.32 (d, *J* = 8.0 Hz, 2H), 6.73 – 6.68 (m, 2H), 6.63-6.61 (m, 1H), 5.91 (s, 2H), 3.78 (d, *J* = 11.2Hz, 2H), 3.53 (t, *J* = 6.8 Hz, 2H), 3.22 (d, *J* = 6.4 Hz, 2H), 2.74 (t, *J* = 6.8 Hz, 2H), 2.43 (s, 3H), 2.22 (td, *J* = 12.0, 2.4 Hz, 2H), 1.74 (d, *J* = 11.2 Hz, 2H), 1.52-1.43 (ddd, *J* = 10.0, 9.2, 4.2 Hz, 1H), 1.36 – 1.27 (m, 2H).

^{13}C NMR (101 MHz, $CDCl_3$) δ 147.5, 143.3, 132.8, 129.6, 127.7, 121.7, 109.3, 108.1, 100.8, 75.2, 72.2, 46.1, 36.0, 35.7, 28.5, 21.5.

HRMS (ESI) Calculated $C_{22}H_{28}NO_5S$ [M+H] +: 418.1688, Found 418.1680.

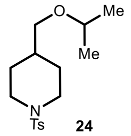

24

24 was prepared on a 0.2 mmol scale from **2** and isopropylamine according to a modified general procedure **B** to give 16.8 mg (27%) of a clear oil after purification with EtOAc/hexanes.

^{1}H NMR (401 MHz, $CDCl_3$) δ 7.64 (d, *J* = 8.0 Hz, 2H), 7.31 (d, *J* = 7.8 Hz, 2H), 3.79 (d, *J* = 11.3 Hz, 2H), 3.48 (dt, *J* = 11.8, 6.1 Hz, 1H), 3.21 (d, *J* = 6.2 Hz, 2H), 2.43 (s, 3H), 2.23 (t, *J* = 10.7 Hz, 2H), 1.78 (d, *J* = 13.5 Hz, 2H), 1.49 – 1.41 (m, 1H), 1.37 – 1.25 (m, 3H), 1.10 (d, *J* = 6.1 Hz, 6H).

^{13}C NMR (151 MHz, $CDCl_3$) δ 143.3 133.3, 129.5, 127.7, 72.5, 71.7, 46.1, 36.0, 28.6, 22.0, 21.5.

HRMS (ESI) Calculated $C_{16}H_{26}NO_3S$ $[M+H]^+$: 312.1633, Found 312.1622.

Note: In this case, the ester step was done at 80 ^0C.

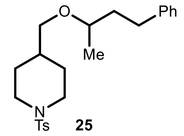

25

25 was prepared on a 0.20 mmol scale according to general procedure **B** from **2** (56.6 mg, 0.20 mmol, 1.0 equiv.) and 3-Amino-1-phenylbutane (32.3 μL, 1.0 equiv.). Isolated in 27% yield (21.7 mg) as white solid after purification by column chromatography (10-20% ethyl acetate in hexane).

Note: In this case, the ester step was done at 80 ^0C.

^1H NMR (600 MHz, $CDCl_3$) δ 7.65 (d, J = 8.4 Hz, 2H), 7.32 (d, J = 7.8 Hz, 2H), 7.29-7.26 (m, 2H), 7.19-7.16 (m, 3H), 3.82 – 3.79 (m, 2H), 3.35 – 3.30 (m, 2H), 3.12 (dd, J = 9.0, 6.6 Hz, 1H), 2.72-2.67 (m, 1H), 2.63-2.58 (m,1H), 2.44 (s, 3H), 2.25 (td, J = 12.0, 2.4 Hz, 2H), 1.85-1.77 (m, 3H), 1.71-1.66 (m, 1H), 1.52-1.45 (m, 1H), 1.40 – 1.32 (m, 2H), 1.12 (d, J = 6.6 Hz, 3H).

^{13}C NMR (150 MHz, $CDCl_3$) δ 143.3, 142.3, 133.3, 129.5, 128.3, 128.3, 127.7, 125.7, 74.7, 72.7, 46.1, 38.3, 36.1, 31.8, 28.8, 28.6, 21.5, 19.5.

HRMS (ESI) Calculated $C_{23}H_{31}NO_3S$+ $[M+H]^+$: 402.2103, Found 402.2098.

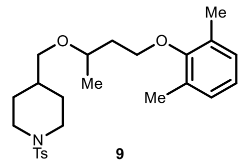

9

8 was prepared on a 0.20 mmol scale according to general procedure **B** from **2** (56.6 mg, 0.20 mmol, 1.0 equiv.) and Mexiletine hydrochloride (43.1 mg, 1.0 equiv.). Isolated in 21% yield (18.1 mg) as white solid after purification by column chromatography (10-30% ethyl acetate in hexane).

Note: In this case, we used 2.0 equiv. *t*BuOK and ester was done at 80 ^0C.

^1H NMR (600 MHz, $CDCl_3$) δ 7.64 (d, J = 8.4 Hz, 2H), 7.31 (d, J = 7.8 Hz, 2H), 6.99 (d, J = 7.2 Hz, 2H), 6.90 (t, J= 7.2 Hz, 1H), 3.79 (d, J = 12.0 Hz, 2H), 3.75 – 3.70 (m, 2H), 3.68 – 3.64 (m, 1H), 3.43 – 3.38 (m, 2H), 2.43 (s, 3H), 2.27 – 2.22 (m, 8H), 1.83 – 1.80 (m, 2H), 1.55 – 1.49 (m, 1H), 1.40 – 1.32 (m, 2H), 1.24 (d, J = 6.0 Hz, 3H).

^{13}C NMR (150 MHz, $CDCl_3$) δ 155.6, 143.3, 130.8, 129.6, 127.7, 123.8, 75.4, 75.0, 73.8, 46.1, 36.1, 28.6, 28.6, 21.5, 17.0, 16.2.

HRMS (ESI) Calculated $C_{24}H_{34}NO_4S$+ [M+H] +: 432.2209, Found 432.2192.

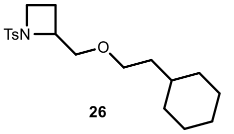

26

26 was prepared on a 0.20 mmol scale according to general procedure **B** from 1-tosylazetidine-2-carboxylic acid (51.1 mg, 0.20 mmol, 1.0 equiv.) and 2-cyclohexyl-ethylamine (25.4 mg, 1.0 equiv.). Isolated in 52% yield (95.0 mg) as colorless liquid after purification by column chromatography (10-30% ethyl acetate in hexane).

^{1}H NMR (401 MHz, $CDCl_3$) δ 7.73 (d, *J* = 8.4 Hz, 2H), 7.36 (d, *J* = 7.6 Hz, 2H), 4.06 – 4.00 (m, 1H), 3.71 (td, *J* = 8.0, 4.0 Hz, 1H), 3.65-3.60 (m, 2H), 3.58 – 3.45 (m, 4H), 2.45 (s, 3H), 2.23-2.14 (m, 1H), 1.98-1.91 (m, 1H), 1.70-1.62 (m, 5H), 1.47-1.42 (m, 2H), 1.37-1.31 (m, 1H), 1.27 – 1.12 (m, 3H), 0.94-0.86 (m, 2H).

^{13}C NMR (101 MHz, $CDCl_3$) δ 143.8, 132.4, 129.6, 128.3, 72.6, 70.0, 62.6, 48.0, 37.0, 34.6, 33.4, 33.4 26.6, 26.3, 21.6, 19.5.

HRMS (ESI) Calculated $C_{19}H_{30}NO_3S$ $[M+H]^+$: 352.1946, Found 352.1936.

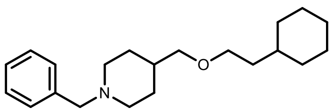

27

27 was prepared on a 0.20 mmol scale according to general procedure **B** from the 1-benzylpiperidine-4-carboxylic acid (43.9 mg, 0.20 mmol, 1.0 equiv.) and 2-cyclohexyl-ethylamine (25.4 mg, 1.0 equiv.). Isolated in 51% yield (32.2 mg) as colorless liquid after purification by column chromatography (10-15% ethyl acetate in hexane).

^{1}H NMR (401 MHz, CDC_{l3}) δ 7.31-7.28 (m, 4H), 7.25 – 7.22 (m, 1H), 3.49 (s, 2H), 3.42 (t, *J* = 6.8 Hz, 2H), 3.23 (d, *J* = 6.8 Hz, 2H), 2.88 (d, *J* = 11.6 Hz, 2H), 1.95 (td, *J* = 11.6, 2.4 Hz, 2H), 1.71 – 1.66 (m, 5H), 1.62 – 1.54 (m, 2H), 1.45 (q, *J* = 6.8 Hz, 2H), 1.41 – 1.33 (m, 1H), 1.31 – 1.09 (m, 6H), 0.96 – 0.85 (m, 2H).

^{13}C NMR (101 MHz, $CDCl_3$) δ 138.7, 129.2, 128.1, 126.8, 76.1, 69.1, 63.5, 53.6, 37.1, 36.3, 34.5, 33.4, 29.4, 26.6, 26.3.

HRMS (ESI) Calculated $C_{21}H_{34}NO$ $[M+H]^{+:}$ 316.2640, Found 316.2635.

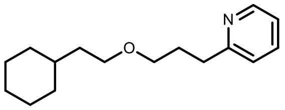

28

28 was prepared on a 0.20 mmol scale according to general procedure **B** from 3-(pyridin-2-yl)propanoic acid (30.2 mg, 0.20 mmol, 1.0 equiv.) and cyclohexyl-ethylamine (25.4 mg, 1.0 equiv.). Isolated in 22% yield (11.0 mg) as colorless liquid after purification by column chromatography (10-35% ethyl acetate in hexane).

^{1}H NMR (401 MHz, $CDCl_3$) δ 8.52 (d, *J* = 4.8 Hz, 1H), 7.58 (td, *J* = 7.6, 2.0 Hz, 1H), 7.16 (d, *J* = 8.0 Hz, 1H), 7.11– 7.08 (m, 1H), 3.44 (td, *J* = 6.8, 3.2 Hz, 4H), 2.89 – 2.84 (m, 2H), 2.01 (q, *J* = 6.4 Hz, 2H), 1.71 – 1.66 (m, 4H), 1.49-1.41 (m, 2H), 1.40 – 1.34 (m, 1H), 1.25 – 1.09 (m, 4H), 0.94-0.85 (m, 2H).

^{13}C NMR (101 MHz, $CDCl_3$) δ 161.9, 149.3, 136.2, 122.8, 120.9, 70.0, 68.8, 37.2, 34.9, 34.6, 33.4, 29.7, 26.6, 26.3.

HRMS (ESI) Calculated $C_{16}H_{26}NO$ [M+H] $^+$: 248.2014, Found 248. 2014.

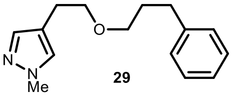

29

29 was prepared on a 0.20 mmol scale according to general procedure **B** from 2-(1-Methyl-1H-pyrazol-4-yl)acetic acid (28.0 mg, 0.20 mmol, 1.0 equiv.) and 3-Phenylpropylamine (28.4 µL, 1.0 equiv.). Isolated in 49% yield (24.0 mg) as colorless liquid after purification by column chromatography (10-25% ethyl acetate in hexane).

^1H NMR (401 MHz, $CDCl_3$) δ 7.67 (d, J = 4.4 Hz, 1H), 7.52 (s, 1H), 7.30 -7.26 (m, 2H), 7.23 – 7.14 (m, 3H), 3.67 (s, 3H), 3.56 (t, J = 6.0 Hz, 2H), 3.44 (t, J = 6.4 Hz, 2H), 2.74 (t, J = 5.6 Hz, 2H), 2.63 (t, J = 7.6 Hz, 2H), 1.90 -1.83 (m, 2H).

^{13}C NMR (101 MHz, $CDCl_3$) δ 141.6, 140.0, 136.0, 128.4, 128.3, 126.0, 119.6, 70.4, 69.7, 37.8, 32.3, 31.1, 24.5.

HRMS (ESI) Calculated $C_{15}H_{21}N_2O$ [M+H] $^+$: 245.1654, Found 245.1645.

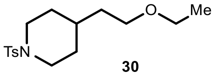

30

30 was prepared on a 0.20 mmol scale according to general procedure **B** from acetic acid (11.4 µL, 0.20 mmol, 1.0 equiv.) and **SI-3** HCl salt (63.8 mg, 1.0 equiv.). Isolated in 40% yield (25.0 mg) as colorless liquid after purification by column chromatography (10-20% ethyl acetate in hexane).

Note: In this case, we used 2.0 equiv. *t*BuOK.

^1H NMR (600 MHz, $CDCl_3$) δ 7.63 (d, J = 8.4 Hz, 2H), 7.31 (d, J = 7.8 Hz, 2H), 3.74 (d, J = 11.4 Hz, 2H), 3.40 (dt, J = 14.4, 7.2 Hz, 4H), 2.43 (s, 3H), 2.25-2.21 (m, 2H), 1.73 (dd, J = 12.2, 1.9 Hz, 2H), 1.48 (q, J = 6.0 Hz, 2H), 1.40 – 1.26 (m, 4H), 1.15 (t, J = 66.0 Hz, 3H).

^{13}C NMR (150 MHz, $CDCl_3$) δ 143.3, 133.2, 129.5, 127.7, 67.7, 66.1, 46.4, 35.8, 32.0, 31.4, 21.5, 15.2.

HRMS (ESI) Calculated $C_{16}H_{26}NO_3S$ [M+H] $^+$: 312.1633, Found 312.1626.

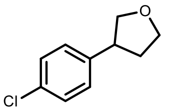

7

9 was prepared on a 0.2 mmol scale from baclofen via a modified general procedure **B** where DIPEA was used instead of KO*t*Bu was used to give 24.8 mg (68%) of a clear oil after purification with EtOAc/hexanes.

Rf = 0.65 in 15:85 EtOAc: Hexane

^{1}H NMR (499 MHz, $CDCl_3$) δ 7.27 (d, *J* = 13.1 Hz, 2H), 7.18 (d, *J* = 8.5 Hz, 2H), 4.11 (t, *J* = 8.1 Hz, 1H), 4.06 (td, *J* = 8.4, 4.6 Hz, 1H), 3.91 (q, *J* = 8.0 Hz, 1H), 3.70 (dd, *J* = 8.5, 7.2 Hz, 1H), 3.38 (p, *J* = 7.7 Hz, 1H), 2.36 (dtd, *J* = 12.4, 7.8, 4.6 Hz, 1H), 1.96 (dq, *J* = 12.4, 7.9 Hz, 1H).

^{13}C NMR (126 MHz, $CDCl_3$) δ 141.5, 132.4, 128.8, 128.7, 74.7, 68.6, 44.5, 34.8.

HRMS (ESI) Calculated $C_{10}H_{12}ClO$ $(M+H)^+$: 183.0577, Found 183.0560.

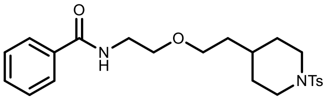

31

31 was prepared on a 0.10 mmol scale from **SI-5** according to general procedure **C** using trispentafluorophenyl boronate to give 29.5 mg (69%) of a white solid after purification with EtOAc.

R_f = 0.28 in 100:0 EtOAc: Hexanes

^{1}H NMR (499 MHz, $CDCl_3$) δ 7.69 (d, *J* = 7.0 Hz, 2H), 7.60 (d, *J* = 8.3 Hz, 2H), 7.48 (t, *J* = 7.4 Hz, 1H), 7.37 (t, *J* = 7.8 Hz, 2H), 7.31 (d, *J* = 8.0 Hz, 2H), 6.49 (t, *J* = 5.5 Hz, 1H), 3.71 (d, *J* = 11.4 Hz, 2H), 3.57 (q, *J* = 5.5 Hz, 2H), 3.52 (t, *J* = 5.1 Hz, 2H), 3.45 (t, *J* = 6.4 Hz, 2H), 2.44 (s, 3H), 2.17 (t, *J* = 11.5 Hz, 2H), 1.70 (d, *J* = 9.5 Hz, 2H), 1.49 (q, *J* = 6.0 Hz, 2H), 1.32 – 1.25 (m, 3H).

^{13}C NMR (126 MHz, $CDCl_3$) δ 167.8, 143.6, 134.4, 133.1, 131.7, 129.7, 128.7, 127.8, 126.9, 69.3, 68.6, 46.5, 39.8, 35.7, 31.6, 21.6.

HRMS (ESI) Calculated $C_{23}H_{31}N_2O_4S$ $[M+H]^+$: 431.2005, Found 431.1995.

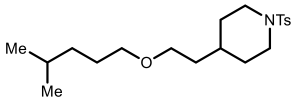

32

32 was prepared on a 0.20 mmol scale according to general procedure **B** from the 4-Methylvaleric acid (25.1 µL, 0.20 mmol, 1.0 equiv.) and **SI-3** (63.8 mg, 1.0 equiv.). Isolated in 54% yield (39.7 mg) as colorless liquid after purification by column chromatography (10-20% ethyl acetate in hexane).

Note: In this case, we used 2.0 equiv. *t*BuOK.

^{1}H NMR (600 MHz, $CDCl_3$) δ 7.63 (d, *J* = 7.8 Hz, 2H), 7.31 (d, *J* = 7.8 Hz, 2H), 3.73 (d, *J* = 11.4 Hz, 2H), 3.38 (t, *J* = 6.0 Hz, 2H), 3.32 (t, *J* = 6.6 Hz, 2H), 2.42 (s, 3H), 2.25-2.21 (m, 2H), 1.73 – 1.71 (m, 2H), 1.54 – 1.46 (m, 5H), 1.36 – 1.27 (m, 3H), 1.17 – 1.14 (m, 2H), 0.85 (d, *J* = 6.6 Hz, 6H).

^{13}C NMR (150 MHz, $CDCl_3$) δ 143.3, 133.1, 129.5, 127.7, 71.3, 67.9, 46.4, 35.7, 35.2, 32.1, 31.4, 27.8, 27.5, 22.5, 21.5.

HRMS (ESI) Calculated $C_{20}H_{33}NO_3S$ $[M+H]^+$: 368.2259, Found 368.2250.

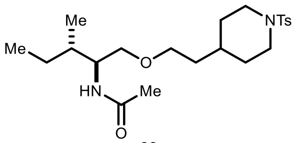

33

33 was prepared on a 0.08 mmol scale from its corresponding ester according to general procedure **C** using trispentfluorophenyl borane to give 10.8 mg (32%) of a white solid after purification with EtOAc.

^{1}H NMR (499 MHz, $CDCl_3$) δ 7.65 (d, *J* = 8.3 Hz, 2H), 7.33 (d, *J* = 8.0 Hz, 2H), 5.54 (d, *J* = 9.3 Hz, 1H), 3.86 (ddt, *J* = 11.9, 8.2, 4.0 Hz, 1H), 3.76 (d, *J* = 11.0 Hz, 2H), 3.47 (dd, *J* = 9.8, 4.1 Hz, 1H), 3.42 (dt, *J* = 9.5, 6.3 Hz, 1H), 3.41 – 3.32 (m, 2H), 2.44 (s, 3H), 2.23 (td, *J* = 11.4, 10.1, 5.4 Hz, 2H), 1.96 (s, 3H), 1.75 – 1.68 (m, 2H), 1.59 (dd, *J* = 6.6, 3.4 Hz, 1H), 1.53 – 1.44 (m, 3H), 1.32 (s, 3H), 1.09 (ddt, *J* = 16.3, 14.1, 7.4 Hz, 1H), 0.91 – 0.84 (m, 6H).

^{13}C NMR (126 MHz, $CDCl_3$) δ 169.67, 143.53, 129.71, 127.89, 77.41, 77.16, 76.91, 70.68, 68.81, 53.01, 46.53, 36.02, 35.80, 32.54, 31.70, 31.55, 25.64, 23.68, 21.66, 15.62, 11.51.

HRMS (ESI) Calculated $C_{22}H_{37}N_2O_4S$ [M+H] $^{+}$: 425.2474, Found 425.2470.

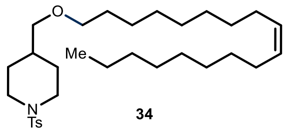

34

34 was prepared on a 0.10 mmol scale according to general procedure **B** from **1** (28.4 mg, 0.10 mmol, 1.0 equiv.) and Oleyl amine (33.0 μL, 1.0 equiv.). Isolated in 52% yield (27.1 mg) as colorless liquid after purification by column chromatography (10-20% ethyl acetate in hexane).

^{1}H NMR (499 MHz, $CDCl_3$) δ 7.64 (d, *J* = 8.0 Hz, 2H), 7.31 (d, *J* = 8.0 Hz, 2H), 5.38 -5.30 (m, 2H), 3.78 (d, *J* = 11.6 Hz, 2H), 3.34 (t, *J* = 6.4 Hz, 2H), 3.20 (d, *J* = 6.4 Hz, 2H), 2.43 (s, 3H), 2.24 (td, *J* = 2.0,1.2 Hz, 2H), 2.03-1.98 (m, 4H), 1.77 (dd, *J* = 1.6, 12.8 Hz, 2H), 1.53-1.44 (m, 3H), 1.37-1.27 (m, 24H), 0.88 (t, *J* = 7.2 Hz, 3H).

^{13}C NMR (126 MHz, $CDCl_3$) δ 143.5, 138.5, 133.5, 129.7, 128.5, 127.9, 127.8, 127.7, 74.7, 73.2, 46.3, 35.9, 28.7, 21.7.

HRMS (ESI) Calculated $C_{31}H_{54}NO_3S$ [M+H] $^{+}$: 520.3824, Found 520.3812.

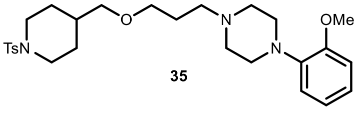

35

35 was prepared on a 0.20 mmol scale according to general procedure **B** from **1** (56.7 mg, 0.20 mmol, 1.0 equiv.) and 3-(4-(2-methoxyphenyl) piperazin-1-yl)propan-1-amine (50.0 mg, 1.0 equiv.). Isolated in 52% yield (52.2 mg) as brown liquid after purification by column chromatography (10-100% ethyl acetate in hexane).

^{1}H NMR (401 MHz, $CDCl_3$) δ 7.64 (d, *J* = 8.0 Hz, 2H), 7.31 (d, *J* = 8.0 Hz, 2H), 7.01 – 6.97 (m, 1H), 6.94 – 6.89 (m, 2H), 6.85 (d, *J* = 8.0 Hz, 1H), 3.85 (s, 3H), 3.78 (d, *J* = 11.6 Hz, 2H), 3.42 (t,

J = 6.4 Hz, 2H), 3.22 (d, *J* = 6.4 Hz, 2H), 3.08 (s, 4H), 2.63 (s, 4H), 2.47 – 2.43 (m, 5H), 2.23 (td, *J* = 11.6, 2.0 Hz, 2H), 1.78 – 1.73 (m, 4H), 1.59 – 1.46 (m, 1H), 1.38-1.25 (m, 2H).

^{13}C NMR (101 MHz, $CDCl_3$) δ 152.3, 143.3, 141.4, 133.4, 129.5, 127.7, 122.8, 121.0, 118.2, 111.2, 75.1, 69.4, 55.5, 55.3, 53.5, 50.6, 46.1, 35.7, 28.5, 27.1, 21.5.

HRMS (ESI) Calculated $C_{27}H_{40}N_3O_4S$ [M+H] $^+$: 502.2740, Found 502.2726.

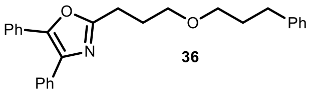

36

36 was prepared on a 0.20 mmol scale according to general procedure **B** from Oxaprozin (58.7 mg, 0.20 mmol, 1.0 equiv.) and 3-Phenylpropylamine (28.4 µL, 1.0 equiv.). Isolated in 48% yield (38.1 mg) as colorless liquid after purification by column chromatography (10-50% ethyl acetate in hexane).

^{1}H NMR (600 MHz, $CDCl_3$) δ 7.76 (s, 1H), 7.70-7.68 (m, 2H), 7.62 – 7.60 (m, 2H), 7.57-7.55 (m, 2H), 7.42 -7.40 (m, 2H), 7.29-7.27 (m, 2H), 7.19 (t, *J* = 7.8 Hz, 3H), 3.46 (t, *J* = 6.0 Hz, 2H), 3.41 (t, *J* = 6.6 Hz, 2H), 2.78 (t, *J* = 7.2 Hz, 2H), 2.69 (t, J = 7.8 Hz 2H), 1.93 -1.88 (m, 4H).

^{13}C NMR (150 MHz, $CDCl_3$) δ 150.7, 141.9, 139.0, 133.8, 132.4, 132.0, 129.0, 128.7, 128.4, 128.3, 126.2, 125.8, 121.6, 120.0, 119.2, 70.0, 69.8, 32.3, 31.3, 30.3, 21.3.

HRMS (ESI) Calculated $C_{27}H_{28}NO_2$ [M+H] $^+$: 398.2120, Found 398.2118.

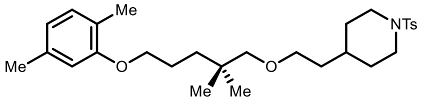

37

37 was prepared on a 0.20 mmol scale according to general procedure **B** from Gemfibrozil (50.1 mg, 0.20 mmol, 1.0 equiv.) and **SI-3** (63.8 mg, 1.0 equiv.). Isolated in 38% yield (38.1 mg) as colorless liquid after purification by column chromatography (10-30% ethyl acetate in hexane).

Note: In this case, we used 2.0 equiv. t_{Bu}OK.

^{1}H NMR (401 MHz, $CDCl_3$) δ 7.62 (d, *J* = 8.0 Hz, 2H), 7.29 (d, *J* = 8.0 Hz, 2H), 7.00 (d, *J* = 7.2 Hz, 1H), 6.66 (d, *J* = 7.6 Hz, 1H), 6.61 (s, 1H), 3.88 (t, *J* = 6.4 Hz, 2H), 3.74 (d, *J* = 11.6 Hz, 2H), 3.38 (t, *J* = 6.0 Hz, 2H), 3.05 (s, 2H), 2.42 (s, 3H), 2.31 (s, 3H), 2.22 (t, *J* = 11.2 Hz, 2H), 2.15 (s, 3H), 1.75 – 1.68 (m, 4H), 1.48 (q, *J* = 6.0 Hz, 2H), 1.39-1.26 (m, 5H), 0.87 (s, 6H).

^{13}C NMR (101 MHz, $CDCl_3$) δ 157.1, 143.2, 136.4, 133.4, 130.2, 129.5, 127.7, 123.5, 120.6, 112.0, 80.0, 68.8, 68.7, 46.4, 35.5, 34.3, 32.4, 31.5, 24.5, 24.2, 21.5, 21.4, 15.7.

HRMS (ESI) Calculated $C_{20}H_{44}NO_4S^+$ [M+H] $^+$: 502.2991, Found 502.2981.

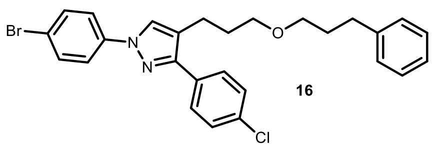

16

16 was prepared on a 0.20 mmol scale according to general procedure **B** from 3-(1-(4-bromophenyl)-3-(4-chlorophenyl)-1H-pyrazol-4-yl)propan-1-amine (78.1 mg, 0.20 mmol, 1.0 equiv.) and 3-Phenylpropylamine (28.4 µL, 1.0 equiv.). Isolated in 51% yield (52.0 mg) as colorless liquid after purification by column chromatography (10-30% ethyl acetate in hexane).

^{1}H NMR (401 MHz, $CDCl_3$) δ 7.65 -7.63 (m, 2H), 7.59 - 7.57 (m, 2H), 7.36 – 7.31 (m, 5H), 7.28-7.24 (m, 3H), 7.19-7.15 (m, 3H), 3.56 (t, *J* = 6.4 Hz, 2H), 3.45 (t, *J* = 6.4 Hz, 2H), 2.97 (t, *J* = 7.6 Hz, 2H), 2.69 (t, *J* = 7.6 Hz, 2H), 2.18- 2.11 (m, 2H), 1.93 -1.86 (m, 2H).

^{13}C NMR (101 MHz, $CDCl_3$) δ 163.3, 142.0, 128.6, 128.5, 128.5, 128.4, 128.3, 128.0, 127.9, 126.4, 125.7, 70.0, 69.6, 32.4, 31.3, 27.3, 25.2.

HRMS (ESI) Calculated $C_{27}H_{27}BrClN_2O$ [M+H]$^{+}$: 509.0995 Found 509.0980.

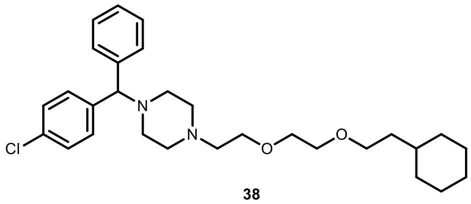

38

38 was prepared on a 0.2 mmol scale from 2-cyclohexylethylamine and cetirizine via a modified general procedure **B** where an extra 1.5 equiv. of GaI_3 was used to give 40.7 mg (42%) of a white solid after purification by preparative HPLC.

^{1}H NMR (499 MHz, $CDCl_3$) δ 7.26 – 7.23 (m, 5H), 7.22 (d, *J* = 4.5 Hz, 4H), 4.02 (s, 1H), 3.74 – 3.69 (m, 2H), 3.54 – 3.49 (m, 2H), 3.43 (t, *J* = 4.6 Hz, 2H), 3.33 (t, *J* = 7.1 Hz, 4H), 3.00 (t, *J* = 4.7 Hz, 2H), 2.87 (t, *J* = 11.8 Hz, 2H), 2.72 (d, *J* = 13.1 Hz, 2H), 2.07 (d, *J* = 12.8 Hz, 3H), 1.64 – 1.49 (m, 6H), 1.28 (q, *J* = 7.0 Hz, 3H), 1.18 (tdd, *J* = 14.0, 6.8, 3.4 Hz, 2H), 1.08 (dd, *J* = 8.3, 2.4 Hz, 2H), 0.77 (qd, *J* = 11.4, 3.5 Hz, 2H).

^{13}C NMR (126 MHz, $CDCl_3$) δ 133.5, 129.2, 129.1, 128.7, 128.0, 127.3, 74.6, 70.5, 69.6, 69.4, 64.9, 53.5, 48.0, 37.0, 34.6, 33.3, 26.4, 26.2.

HRMS (ESI) Calculated $C_{29}H_{42}ClN_2O_2^{+}$ [M+H]$^{+}$: 485.2929, Found 485.2932.

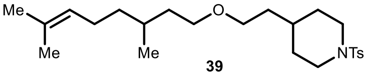

39

39 was prepared on a 0.20 mmol scale according to general procedure **B** from citronellic acid (34.1 mg, 0.20 mmol, 1.0 equiv.) and **SI-3** (63.8 mg, 1.0 equiv.). Isolated in 51% yield (52.0 mg) as colorless liquid after purification by column chromatography (10-30% ethyl acetate in hexane).

Note: In this case, we used 2.0 equiv. *t*BuOK.

^{1}H NMR (600 MHz, $CDCl_3$) δ 7.63 (d, *J* = 7.2 Hz, 2H), 7.31 (d, *J* = 7.8 Hz, 2H), 5.08-5.05 (m, 1H), 3.74 (d, *J* = 11.4 Hz, 2H), 3.41 -3.34 (m, 4H), 2.43 (s, 3H), 2.23 (t, *J* = 11.4 Hz, 2H), 2.00 -1.88 (m, 2H), 1.73 (d, *J* = 11.6 Hz, 2H), 1.66 (s, 3H), 1.56 -1.54 (m, 4H), 1.51 -1.46 (m, 3H), 1.36 – 1.25 (m, 5H), 1.15 – 1.10 (m, 1H), 0.86 (d, *J* = 6.6 Hz, 3H).

^{13}C NMR (150 MHz, $CDCl_3$) δ 143.3, 133.2, 131.1, 129.5, 127.7, 124.7, 69.3, 68.0, 46.4, 37.2, 36.6, 35.8, 32.1, 31.4, 29.6, 25.7, 25.4, 21.5, 19.5, 17.6.

HRMS (ESI) Calculated $C_{24}H_{40}NO_3S$ $[M+H]^+$: 422.2729, Found 422.2720.

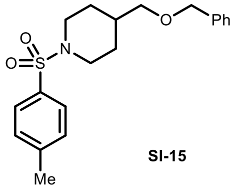

SI-15

4-((benzyloxy)methyl)-1-tosylpiperidine (SI-15)

SI-15 was prepared on a 0.20 mmol scale according to general procedure from **SI-14** and **1** via general procedure A to give 43.8 mg (61%) of a clear oil after purification with EtOAc/hexanes.

R_f = 0.38 in 25:75 EtOAc: hexanes

^{1}H NMR (499 MHz, $CDCl_3$) δ 7.64 (d, *J* = 8.3 Hz, 2H), 7.39 – 7.30 (m, 4H), 7.29 – 7.27 (m, 2H), 4.46 (s, 2H), 3.78 (dt, *J* = 11.5, 2.7 Hz, 2H), 3.28 (d, *J* = 6.5 Hz, 2H), 2.43 (s, 3H), 2.24 (td, *J* = 12.0, 2.6 Hz, 2H), 1.84 – 1.76 (m, 2H), 1.35 (qd, *J* = 12.0, 4.2 Hz, 2H).

^{13}C NMR (126 MHz, $CDCl_3$) δ 143.5, 138.5, 133.5, 129.7, 128.5, 127.9, 127.8, 127.7, 74.7, 73.2, 46.3, 35.9, 28.7, 21.7.

HRMS (ESI) Calculated $C_{20}H_{26}NO_3S^+$ $[M+H]^+$: 360.1628, Found 360.1625.

Characterization of deuterated ether products:

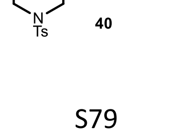

40

40 was prepared on a 0.10 mmol scale according to general procedure **C** from the corresponding ester (34.1 mg, 0.10 mmol, 1.0 equiv.) and Diphenyl(silane-d$_2$) (28.0 μL, 1.0 equiv.). Isolated in 55% yield (21.0 mg) with >95% *d*-incorporation as white solid after purification by column chromatography (10-30% ethyl acetate in hexane).

Note: In this case we used 0.25 equiv. Trimesitylborane.

^{1}H NMR (600 MHz, $CDCl_3$) δ 7.64 (d, *J* = 8.4 Hz, 2H), 7.31 (d, *J* = 7.8 Hz, 2H), 3.78 (d, *J* = 11.4 Hz, 2H), 3.38 (t, *J* = 7.2 Hz, 2H), 2.43 (s, 3H), 2.24 (td, *J* = 12.0, 2.4 Hz, 2H), 1.76 (dd, *J* = 12.6, 1.8 Hz, 2H), 1.67-1.62 (m, 5H), 1.51-1.46 (m, 1H), 1.42 (q, *J* = 6.6 Hz, 2H), 1.35-1.29 (m, 3H), 1.24 – 1.11 (m, 3H), 0.92-0.85 (m, 2H).

^{13}C NMR (150 MHz, $CDCl_3$) δ 143.3, 133.3, 129.5, 127.7, 69.1, 46.1, 37.1, 35.5, 34.6, 33.3, 28.5, 26.6, 26.3, 21.5.

HRMS (ESI) Calculated $C_{21}H_{31}D_2NNaO_3S$ [M+Na]$^+$: 404.2204, Found 404.2190.

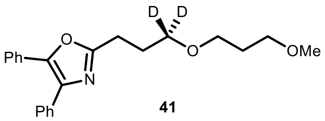

41

41 was prepared on a 0.10 mmol scale according to general procedure **B** from 3-methoxypropyl 3-(4,5-diphenyloxazol-2-yl) propanoate (36.5 mg, 0.10 mmol, 1.0 equiv.) and Diphenyl(silane-*d*$_2$) (28.0 μL, 1.0 equiv.). Isolated in 64% yield (22.6 mg) with >95% *d*-incorporation as colorless liquid after purification by column chromatography (10-30% ethyl acetate in hexane).

Note: In this case, we used 0.25 equiv. $B(C_6F_5)_3$.

^{1}H NMR (600 MHz, $CDCl_3$) δ 7.65 – 7.63 (m, 2H), 7.60 – 7.58 (m, 2H), 7.38 – 7.30 (m, 6H), 3.52 (t, *J* = 6.6 Hz, 2H), 3.46 (t, *J* = 6.6 Hz, 2H), 3.32 (s, 3H), 2.94 (t, *J* = 7.2 Hz, 2H), 2.11 (t, *J* = 7.2 Hz, 2H), 1.85 (q, *J* = 6.6 Hz, 2H).

^{13}C NMR (150 MHz, $CDCl_3$) δ 163.3, 145.1, 134.3, 132.6, 129.2, 128.6, 128.5, 128.3, 128.0, 127.9, 126.4, 69.7, 67.7, 58.6, 30.0, 27.0, 25.0.

HRMS (ESI) Calculated $C_{22}H_{24}D_2NO_3$ [M+H]$^+$: 354.2038, Found 354.2027.

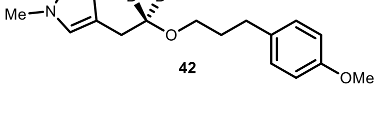

42

42 was prepared on a 0.10 mmol scale according to general procedure **B** from 3-(4-methoxyphenyl) propyl 2-(1-methyl-1H-pyrazol-4-yl)acetate (28.8 mg, 0.10 mmol, 1.0 equiv.) and Diphenyl(silane-*d*$_2$) (28.0 μL, 1.0 equiv.). Isolated in 64% yield (43.2 mg) with >95% *d*-incorporation as colorless liquid after purification by column chromatography (10-30% ethyl acetate in hexane).

Note: In this case, we used 0.25 equiv. $B(C_6F_5)_3$.

^{1}H NMR (401 MHz, $CDCl_3$) δ 7.34 (s, 1H), 7.20 (s, 1H), 7.09 (d, *J* = 8.0 Hz, 2H), 6.82 (d, *J* = 8.4 Hz, 2H), 3.85 (s, 3H), 3.79 (s, 3H), 3.44 (t, *J* = 6.4 Hz, 2H), 2.63 (q, J= 8.0 Hz, 3H), 1.90-1.83 (m, 2H).

^{13}C NMR (101 MHz, $CDCl_3$) δ 157.8, 139.0, 134.0, 129.3, 128.7, 118.4, 113.8, 69.9, 55.2, 38.7, 31.5, 31.4, 24.7.

HRMS (ESI) Calculated $C_{16}H_{21}D_2N_2O_2$ [M+H] $^+$: 277.1885, Found 277.1875.

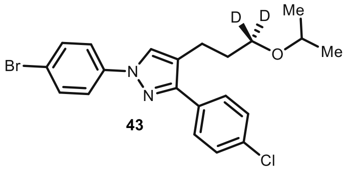

43

43 was prepared on a 0.10 mmol scale according to general procedure **B** from the isopropyl 3-(1-(4-bromophenyl)-3-(4-chlorophenyl)-*1H*-pyrazol-4-yl)propanoate (44.7 mg, 0.10 mmol, 1.0 equiv.) and Diphenyl(silane-*d*$_2$) (28.0 μL, 1.0 equiv.). Isolated in 65% yield (28.3 mg) with >95% *d*-incorporation as colorless liquid after purification by column chromatography (10-20% ethyl acetate in hexane).

Note: In this case, we used 0.25 equiv. $B(C_6F_5)_3$.

^{1}H NMR (600 MHz, $CDCl_3$) δ 7.76 (s, 1H), 7.70 – 7.68 (m, 2H), 7.62 – 7.59 (m, 2H), 7.57 – 7.55 (m, 2H), 7.42 – 7.40 (m, 2H), 3.56-3.52 (m,1H), 2.75 (t, *J* = 7.8 Hz, 2H), 1.87 (t, *J* = 7.8 Hz, 2H), 1.15 (d, *J* = 6.0 Hz, 6H).

^{13}C NMR (101 MHz, $CDCl_3$) δ 150.6, 139.0, 134.3, 133.7, 132.4, 132.0, 129.0, 128.7, 126.2, 121.7, 120.0, 119.2, 71.4, 30.4, 22.2, 21.3.

HRMS (ESI) Calculated $C_{21}H_{21}D_2BrClN_2O$ [M+H] $^+$: 435.0808, Found 435.0788.

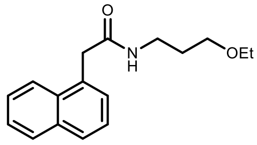

68

68 was prepared on a 0.20 mmol scale from **68** according to general procedure **C** using trispentafluorophenyl borane to give 42.1 mg (78 %) of a white solid after purification with EtOAc.

^{1}H NMR (400 MHz, $CDCl_3$) δ 7.96 – 7.89 (m, 1H), 7.89 – 7.83 (m, 1H), 7.81 (d, *J* = 8.2 Hz, 1H), 7.56 – 7.45 (m, 2H), 7.44 (dd, *J* = 8.2, 7.0 Hz, 1H), 7.37 (dd, *J* = 7.0, 1.3 Hz, 1H), 5.92 (s, 1H), 3.97 (s, 2H), 3.27 – 3.17 (m, 4H), 3.12 (q, *J* = 7.0 Hz, 2H), 1.55 (p, *J* = 6.0 Hz, 2H), 0.89 (t, *J* = 7.0 Hz, 3H).

^{13}C NMR (101 MHz, $CDCl_3$) δ 171.2, 134.0, 132.2, 131.2, 128.9, 128.6, 126.8, 126.3, 125.8, 123.90, 77.5, 77.2, 76.8, 69.0, 66.2, 41.9, 38.3, 28.9, 15.0.

Characterization of phenolic ether products

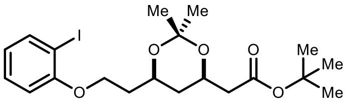

46

46 was prepared on a 0.2 mmol scale from 2-iodophenol and *tert*-butyl 2-((4*R*,6*R*)-6-(2-aminoethyl)-2,2-dimethyl-1,3-dioxan-4-yl)acetate via general procedure **D** to give 61.0 mg (64%) of a clear oil after purification with EtOAc/hexanes.

^{1}H NMR (499 MHz, $CDCl_3$) δ 7.76 (dd, *J* = 7.8, 1.6 Hz, 1H), 7.28 (ddd, *J* = 8.2, 7.3, 1.6 Hz, 1H), 6.82 (dd, *J* = 8.2, 1.3 Hz, 1H), 6.70 (td, *J* = 7.6, 1.3 Hz, 1H), 4.30 (qdd, *J* = 11.8, 5.2, 2.4 Hz, 2H), 4.14 (td, *J* = 8.8, 4.8 Hz, 1H), 4.12 – 4.04 (m, 1H), 2.44 (dd, *J* = 15.1, 7.2 Hz, 1H), 2.32 (dd, *J* = 15.1, 6.0 Hz, 1H), 2.04 – 1.87 (m, 2H), 1.67 (dt, *J* = 12.7, 2.5 Hz, 1H), 1.46 (s, 3H), 1.44 (s, 9H), 1.36 (s, 3H), 1.34 – 1.23 (m, 1H).

^{13}C NMR (126 MHz, $CDCl_3$) δ 170.4, 157.6, 139.5, 129.6, 122.6, 112.3, 99.0, 86.8, 80.7, 66.4, 65.7, 65.0, 42.9, 36.8, 35.9, 30.2, 28.3, 20.0

HRMS (ESI) Calculated $C_{20}H_{29}INaO_5^+$ $[M+Na]^+$: 499.0952, Found 499.0944.

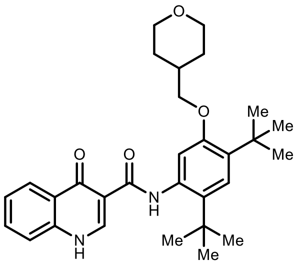

47

Compound **47** was prepared on a 0.20 mmol scale from ivacaftor and 4-tetrahydropyranyl methanamine via general procedure **D** to give 39.2 mg (40%) of a white solid after purification by preparative LC.

^{1}H NMR (400 MHz, $CDCl_3$) δ 11.91 (s, 1H), 8.87 (s, 1H), 8.59 (dd, *J* = 8.3, 1.6 Hz, 1H), 7.76 (ddd, *J* = 8.7, 7.1, 1.6 Hz, 1H), 7.55 – 7.46 (m, 2H), 7.27 (s, 2H), 7.15 (s, 1H), 4.13 (d, *J* = 7.2 Hz, 2H), 3.91 (dd, *J* = 11.5, 3.8 Hz, 2H), 3.26 (td, *J* = 11.1, 2.6 Hz, 2H), 2.16 (s, 1H), 1.56 – 1.42 (m, 13H), 1.38 (s, 9H).

^{13}C NMR (101 MHz, $CDCl_3$) δ 176.8, 153.5, 148.7, 139.3, 134.0, 133.2, 133.2, 132.8, 128.1, 128.0, 125.4, 125.1, 125.1, 116.4, 116.0, 111.5, 67.2, 59.7, 34.9, 34.8, 34.5, 31.0, 30.4, 29.8.

HRMS (ESI) Calculated $C_{30}H_{39}N_2O_4^+$ $[M+H]^+$: 491.2904, Found 491.2914

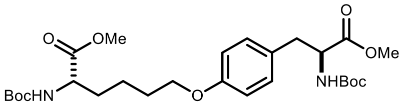

48

Compound **48** was prepared on a 0.10 mmol scale from *N*-α-Boc lysine methyl ester hydrochloride and boc tyrosine methyl ester via general procedure **D** to give 18.6 mg (35%) of a white solid after purification by preparative LC.

^{1}H NMR (400 MHz, $CDCl_3$) δ 7.01 (d, *J* = 8.4 Hz, 2H), 6.79 (d, *J* = 8.5 Hz, 2H), 5.03 (d, *J* = 8.5 Hz, 1H), 4.95 (d, *J* = 8.1 Hz, 1H), 4.53 (q, *J* = 6.6 Hz, 1H), 4.32 (q, *J* = 6.3 Hz, 1H), 3.91 (t, *J* = 6.3 Hz, 2H), 3.74 (s, 3H), 3.71 (s, 3H), 3.01 (t, *J* = 6.8 Hz, 2H), 1.84 (dd, *J* = 20.4, 7.5 Hz, 2H), 1.76 – 1.62 (m, 4H), 1.53 (q, *J* = 7.8 Hz, 1H), 1.44 (s, 10H), 1.42 (s, 9H).

^{13}C NMR (101 MHz, $CDCl_3$) δ 172.6, 158.2, 130.4, 128.0, 114.6, 79.9, 67.5, 54.7, 53.5, 52.5, 52.4, 37.6, 32.7, 31.7, 28.9, 28.5, 22.2.

HRMS (ESI) Calculated $C_{27}H_{42}N_2NaO_9^+$ $[M+Na]^+$: 561.2783, Found 561.2763

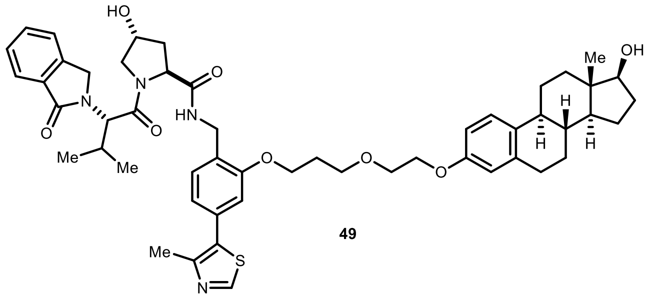

49

Compound **49** was prepared on a 0.10 mmol scale from (*8R*,*9S*,*13S*,*14S*,*17S*)-3-(2-(2-aminoethoxy)ethoxy)-13-methyl-7,8,9,11,12,13,14,15,16,17-decahydro-*6H*-cyclopenta[*a*]phenanthren-17-ol hydrochloride and estradiol via general procedure **D** to give 22.5 mg (25%) of a white solid after purification by preparative LC.

^{1}H NMR (499 MHz, $CDCl_3$) δ 8.73 (s, 1H), 7.79 (d, *J* = 7.6 Hz, 1H), 7.54 (td, *J* = 7.5, 1.1 Hz, 1H), 7.44 (dd, *J* = 12.0, 7.9 Hz, 2H), 7.32 (dd, *J* = 9.6, 6.8 Hz, 2H), 7.16 (d, *J* = 8.6 Hz, 1H), 6.97 (dd, *J* = 7.7, 1.6 Hz, 1H), 6.91 (d, *J* = 1.6 Hz, 1H), 6.68 (dd, *J* = 8.6, 2.8 Hz, 1H), 6.61 (d, *J* = 2.7 Hz, 1H), 4.77 (d, *J* = 6.3 Hz, 1H), 4.74 (s, 1H), 4.65 (t, *J* = 7.9 Hz, 1H), 4.53 (d, *J* = 5.8 Hz, 1H), 4.51 – 4.45 (m, 2H), 4.44 (d, *J* = 6.1 Hz, 1H), 4.16 (td, *J* = 6.2, 1.9 Hz, 2H), 4.09 (t, *J* = 4.5 Hz, 2H), 3.82 (dd, *J* = 5.6, 4.0 Hz, 2H), 3.78 (t, *J* = 6.0 Hz, 2H), 3.73 (t, *J* = 8.5 Hz, 1H), 3.64 (dd, *J* = 11.5, 3.5 Hz, 1H), 3.50 (s, 2H), 2.80 (dd, *J* = 12.7, 6.2 Hz, 2H), 2.54 (s, 3H), 2.41 (dt, *J* = 11.0, 6.5 Hz, 2H), 2.30 (ddd, *J* = 13.5, 10.0, 5.5 Hz, 4H), 2.22 – 2.15 (m, 7H), 2.15 – 2.07 (m, 1H), 2.08 – 2.00 (m, 2H), 1.94 (dt, *J* = 12.5, 3.3 Hz, 1H), 1.89 – 1.82 (m, 1H), 1.72 – 1.64 (m, 1H), 1.49 (ddd, *J* = 20.8, 12.0, 5.5 Hz, 2H), 1.42 – 1.13 (m, 8H), 0.90 (dd, *J* = 6.6, 3.6 Hz, 6H), 0.77 (s, 3H).

^{13}C NMR (126 MHz, $CDCl_3$) δ 170.4, 170.3, 169.6, 156.8, 156.6, 150.5, 142.1, 138.0, 132.9, 132.0, 131.8, 131.6, 129.4, 128.0, 127.9, 126.5, 126.3, 123.8, 122.9, 121.5, 114.6, 112.1, 112.1,

81.9, 70.0, 69.6, 67.9, 67.4, 65.1, 58.7, 58.4, 55.9, 50.0, 47.5, 43.9, 43.2, 38.9, 38.8, 36.7, 35.6, 30.9, 30.6, 29.8, 29.6, 28.7, 27.2, 26.3, 23.1, 19.1, 19.0, 15.9, 11.1.

HRMS (ESI) Calculated $C_{52}H_{65}N_4O_8S^+$ [M+H] $^+$: 945.4518, Found 945.4506

Characterization of alkyl halide products.

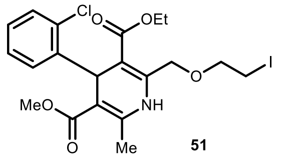

51

Compound **51** was prepared on a 0.20 mmol scale from amlodipine besylate and potassium iodide via general procedure **E** to give 74.7 mg (72%) of a yellow oil after purification with EtOAc/hexanes.

R_f = 0.65 in 25:75 EtOAc:hexanes

^{1}H NMR (499 MHz, $CDCl_3$) δ 7.39 (dd, *J* = 7.8, 1.7 Hz, 1H), 7.24 (d, *J* = 8.4 Hz, 2H), 7.15 (t, *J* = 7.5 Hz, 1H), 7.08 – 7.03 (m, 1H), 5.42 (s, 1H), 4.80 (q, *J* = 16.1 Hz, 2H), 4.05 (tt, *J* = 10.7, 5.4 Hz, 2H), 3.82 (qt, *J* = 11.0, 5.9 Hz, 2H), 3.63 (s, 3H), 3.38 (t, *J* = 5.9 Hz, 2H), 2.39 (s, 3H), 1.19 (t, *J* = 7.1 Hz, 3H).

^{13}C NMR (126 MHz, $CDCl_3$) δ 168.0, 167.2, 145.7, 144.9, 144.0, 132.4, 131.5, 129.3, 127.4, 126.9, 104.0, 101.6, 71.1, 67.6, 59.8, 50.8, 37.2, 19.6, 14.3, 3.6.

HRMS (ESI) Calculated $C_{20}H_{24}ClIN_4O_5^+$ [M+H]$^+$: 520.0382, Found 520.0388

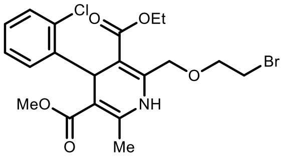

52

Compound **52** was prepared on a 0.20 mmol scale from amlodipine besylate and potassium bromide via general procedure **E** to give 81.1 mg (86%) of a yellow oil after purification with EtOAc/hexanes.

R_f = 0.65 in 25:75 EtOAc:hexanes

^{1}H NMR (400 MHz, $CDCl_3$) δ 7.38 (dd, *J* = 7.8, 1.7 Hz, 1H), 7.25 (s, 1H), 7.23 (dd, *J* = 7.9, 1.4 Hz, 1H), 7.14 (td, *J* = 7.5, 1.4 Hz, 1H), 7.04 (ddd, *J* = 7.8, 1.7, 0.0 Hz, 1H), 5.41 (s, 1H), 4.76 (dd, *J* = 16.2, 11.5 Hz, 2H), 4.04 (qt, *J* = 7.2, 3.6 Hz, 2H), 3.96 – 3.82 (m, 2H), 3.61 (s, 3H), 3.59 (t, *J* = 5.3 Hz, 2H), 2.36 (s, 3H), 1.18 (t, *J* = 7.1 Hz, 3H).

^{13}C NMR (101 MHz, $CDCl_3$) δ 168.1, 167.3, 145.8, 145.1, 144.2, 132.5, 131.6, 129.4, 127.5, 127.0, 104.1, 101.7, 71.1, 67.9, 60.0, 51.0, 37.4, 37.3, 31.3, 19.64, 19.62, 14.4.

HRMS (ESI) Calculated $C_{20}H_{24}ClBrN_4O_5^+$ [M+H]$^+$: 472.0521, Found 472.0519

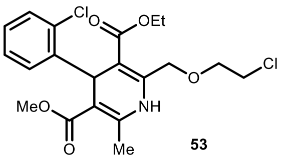

53

Compound **53** was prepared on a 0.20 mmol scale from amlodipine besylate and lithium chloride via general procedure **E** to give 30.8 mg (36%) of a yellow oil after purification with EtOAc/hexanes.

R_f = 0.63 in 25:75 EtOAc:hexanes

^{1}H NMR (400 MHz, $CDCl_3$) δ 7.38 (dd, *J* = 7.7, 1.7 Hz, 1H), 7.23 (dd, *J* = 8.0, 1.4 Hz, 3H), 7.14 (td, *J* = 7.5, 1.4 Hz, 1H), 7.04 (td, *J* = 7.6, 1.7 Hz, 1H), 5.41 (s, 1H), 4.79 (dd, *J* = 16.1, 14.8 Hz, 2H), 4.05 (tt, *J* = 7.1, 3.7 Hz, 2H), 3.91 – 3.76 (m, 2H), 3.74 (t, *J* = 4.7 Hz, 2H), 3.62 (s, 3H), 2.35 (s, 3H), 1.19 (t, *J* = 7.1 Hz, 3H).

^{13}C NMR (101 MHz, $CDCl_3$) δ 168.1, 167.4, 145.8, 145.1, 144.2, 132.5, 131.6, 129.4, 127.5, 127.0, 104.1, 101.8, 71.4, 68.0, 50.9, 43.4, 37.4, 19.6, 14.4.

HRMS (ESI) Calculated $C_{20}H_{24}Cl_2N_4O_5^+$ [M+H]$^+$: 428.1026, Found 428.1016

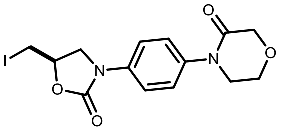

54

Compound **54** was prepared on a 0.20 mmol scale from (*S*)-4-(4-(5-(aminomethyl)-2-oxooxazolidin-3-yl)phenyl)morpholin-3-one and KI via general procedure **E** to give 54.5 mg (68%) of a yellow oil after purification with EtOAc/hexanes.

R_f = 0.27 in 100:0 EtOAc:hexanes

^{1}H NMR (499 MHz, $CDCl_3$) δ 7.60 (d, *J* = 8.9 Hz, 2H), 7.36 (d, *J* = 8.9 Hz, 2H), 4.74 (tdd, *J* = 8.4, 5.9, 3.9 Hz, 1H), 4.34 (s, 2H), 4.18 (t, *J* = 8.9 Hz, 1H), 4.04 (dd, *J* = 5.6, 4.5 Hz, 2H), 3.80 (dd, *J* = 9.2, 6.0 Hz, 1H), 3.75 (dd, *J* = 9.9, 5.2 Hz, 3H), 3.48 (dd, *J* = 10.4, 3.9 Hz, 1H), 3.37 (dd, *J* = 10.4, 8.2 Hz, 1H).

^{13}C NMR (126 MHz, $CDCl_3$) δ 166.80, 136.51, 126.23, 119.15, 71.19, 68.57, 64.11, 51.01, 49.65, 5.97.

HRMS (ESI) Calculated $C_{14}H_{16}IN_2O_4^+$ [M+H]$^+$: 403.0149, Found 403.0132

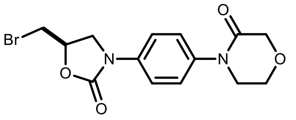

55

Compound **55** was prepared on a 0.20 mmol scale from (*S*)-4-(4-(5-(aminomethyl)-2-oxooxazolidin-3-yl) phenyl)morpholin-3-one and KBr via general procedure **E** to give 52.6 mg (75%) of a yellow oil after purification with EtOAc/hexanes.

R_f = 0.25 in 100:0 EtOAc:hexanes

^{1}H NMR (499 MHz, $CDCl_3$) δ 7.60 (d, *J* = 9.0 Hz, 2H), 7.36 (d, *J* = 8.9 Hz, 2H), 4.97 – 4.80 (m, 1H), 4.34 (s, 2H), 4.18 (t, *J* = 8.9 Hz, 1H), 4.04 (dd, *J* = 5.6, 4.5 Hz, 2H), 3.93 (dd, *J* = 9.2, 5.8 Hz, 1H), 3.76 (dd, *J* = 5.9, 4.2 Hz, 2H), 3.65 (dd, *J* = 10.8, 3.8 Hz, 1H), 3.57 (dd, *J* = 10.8, 7.4 Hz, 1H).

^{13}C NMR (126 MHz, $CDCl_3$) δ 166.8, 153.8, 137.4, 136.5, 126.2, 119.1, 70.6, 68.6, 64.1, 49.6, 49.2, 32.5.

HRMS (ESI) Calculated $C_{14}H_{16}BrN_2O_4^+$ [M+H]$^+$: 355.0288, Found 355.0231.

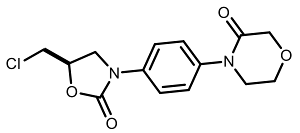

56

Compound **56** was prepared on a 0.20 mmol scale from (*S*)-4-(4-(5-(aminomethyl)-2-oxooxazolidin-3-yl) phenyl) morpholin-3-one and LiCl via general procedure **E** to give 20.0 mg (32%) of a yellow oil after purification with EtOAc/hexanes.

R_f = 0.22 in 100:0 EtOAc:hexanes

^{1}H NMR (499 MHz, $CDCl_3$) δ 7.59 (d, *J* = 8.6 Hz, 3H), 7.36 (d, *J* = 8.7 Hz, 2H), 4.88 (ddd, *J* = 9.8, 7.7, 4.9 Hz, 1H), 4.34 (s, 2H), 4.16 (t, *J* = 9.0 Hz, 1H), 4.04 (dd, *J* = 5.6, 4.5 Hz, 2H), 3.96 (dd, *J* = 9.1, 5.7 Hz, 1H), 3.84 – 3.69 (m, 4H).

^{13}C NMR (126 MHz, $CDCl_3$) δ 154.0, 137.5, 136.7, 126.4, 119.3, 71.0, 68.7, 64.2, 49.8, 48.3, 44.7

HRMS (ESI) Calculated $C_{14}H_{16}ClN_2O_4^+$ [M+H]$^+$: 311.0793, Found 311.0793.

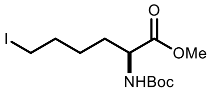

57

Compound **57** was prepared on a 0.20 mmol scale from boc lysine methyl ester hydrochloride and KI via general procedure **E** to give 34.8 mg (47%) of yellow oil after purification with EtOAc/hexanes.

R_f = 0.35 in 25:75 EtOAc:hexanes

^{1}H NMR (499 MHz, $CDCl_3$) δ 5.03 (d, *J* = 8.0 Hz, 1H), 4.32 (q, *J* = 14.4, 7.5, 6.9 Hz, 1H), 3.76 (s, 3H), 3.18 (t, *J* = 6.9 Hz, 2H), 1.84 (qp, *J* = 14.2, 7.4 Hz, 4H), 1.71 – 1.60 (m, 1H), 1.53 – 1.41 (m, 11H).

^{13}C NMR (126 MHz, $CDCl_3$) δ 173.1, 155.3, 80.0, 53.1, 52.3, 32.7, 31.7, 28.3, 26.2, 6.1.

HRMS (ESI) Calculated $C_{12}H_{22}INaNO_4^+$ $[M+H]^+$: 394.0486, Found 394.0455

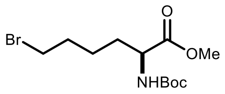

58

methyl (*S*)-2-((*tert*-butoxycarbonyl) amino)-6-bromohexanoate (60)

Compound **58** was prepared on a 0.20 mmol scale from boc lysine methyl ester hydrochloride and KBr via general procedure **E** to give 27.0 mg (42%) of yellow oil after purification with EtOAc/hexanes.

R_f = 0.30 in 25:75 EtOAc:hexanes

^{1}H NMR (499 MHz, $CDCl_3$) δ 5.02 (d, *J* = 8.4 Hz, 1H), 4.31 (q, *J* = 13.0, 6.9 Hz, 1H), 3.75 (s, 3H), 3.39 (t, *J* = 6.7 Hz, 2H), 1.96 – 1.77 (m, 4H), 1.70 – 1.59 (m, 1H), 1.55 – 1.47 (m, 2H), 1.44 (s, 9H).

^{13}C NMR (126 MHz, $CDCl_3$) δ 173.0, 155.4, 80.0, 53.1, 52.3, 33.2, 32.0, 31.9, 28.3, 23.8.

HRMS (ESI) Calculated $C_{12}H_{22}BrNaNO_4^+$ $[M+H]^+$: 346.0624, Found 346.0622

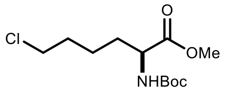

59

Compound **59** was prepared on a 0.20 mmol scale from boc lysine methyl ester hydrochloride and LiCl via general procedure **E** to give 12.3 mg (22%) of yellow oil after purification with EtOAc/hexanes.

R_f = 0.30 in 25:75 EtOAc:hexanes

^{1}H NMR (499 MHz, $CDCl_3$) δ 5.02 (d, *J* = 8.2 Hz, 1H), 4.31 (q, *J* = 7.4 Hz, 1H), 3.74 (s, 3H), 3.52 (t, *J* = 6.5 Hz, 2H), 1.89 – 1.71 (m, 3H), 1.72 – 1.60 (m, 1H), 1.56 – 1.45 (m, 2H), 1.44 (s, 9H).

^{13}C NMR (126 MHz, $CDCl_3$) δ 173.3, 80.1, 53.3, 52.5, 44.7, 32.2, 32.1, 28.5, 22.7.

HRMS (ESI) Calculated $C_{12}H_{22}ClNaNO_4^+$ $[M+H]^+$: 302.1130, Found 302.1133

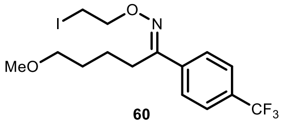

60

Compound **60** was prepared on a 0.20 mmol scale from the triphenylpyridinium salt of fluvoxamine and KI via a modified general procedure **E** where DMA was used as a solvent to give 80.0 mg (92%) of a tan solid after purification with EtOAc/hexanes.

Note: The triphenylpyrylium salt was used because fluvoxamine is supplied as the maleate salt which when subjected to these reaction conditions generated the corresponding ester.

R_f = 0.75 in 20:80 EtOAc:hexanes

^{1}H NMR (499 MHz, cdcl$_3$) δ 7.74 (d, *J* = 8.2 Hz, 3H), 7.62 (d, *J* = 7.9 Hz, 4H), 4.42 (t, *J* = 6.8 Hz, 4H), 3.44 – 3.36 (m, 6H), 2.81 (t, *J* = 7.5 Hz, 2H), 1.70 – 1.59 (m, *J* = 3.3 Hz, 7H).

^{13}C NMR (126 MHz, cdcl$_3$) δ 158.32, 138.86, 131.13, 126.08 (q, *J* = 197.7 Hz), 125.40 (q, *J* = 3.8 Hz), 122.90, 74.24, 72.25, 58.60, 29.59, 26.35, 23.19, 2.88.

^{19}F NMR (376 MHz, cdcl$_3$) δ -62.77.

HRMS (ESI) Calculated $C_{15}H_{20}F_3INO_2^+$ [M+H]$^+$: 430.0485, Found 430.0485

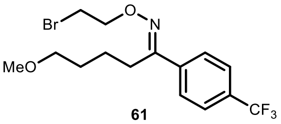

61

Compound **61** was prepared on a 0.20 mmol scale from the triphenylpyridinium salt of fluvoxamine and KI via a modified general procedure **E** where DMA was used as a solvent to give 67.8 mg (89%) of a tan solid after purification with EtOAc/hexanes.

Note: The triphenylpyrylium salt was used because fluvoxamine is supplied as the maleate salt which when subjected to these reaction conditions generated the corresponding ester.

R_f = 0.75 in 20:80 EtOAc:hexanes

^{1}H NMR (499 MHz, cdcl$_3$) δ 7.74 (d, *J* = 8.1 Hz, 1H), 7.62 (d, *J* = 7.9 Hz, 1H), 4.47 (t, *J* = 6.2 Hz, 2H), 3.62 (t, *J* = 6.2 Hz, 2H), 3.42 – 3.36 (m, 2H), 3.32 (s, 3H), 2.84 – 2.78 (m, 2H), 1.69 – 1.58 (m, 3H).

^{13}C NMR (126 MHz, cdcl$_3$) δ 158.58, 139.01, 126.08 (q, *J* = 197.7 Hz), 125.40 (q, *J* = 3.8 Hz), 73.69, 72.40, 58.74, 30.21, 29.71, 26.45, 23.29.

^{19}F NMR (376 MHz, cdcl$_3$) δ -62.77.

HRMS (ESI) Calculated $C_{15}H_{20}F_3BrNO_2^+$ [M+H]$^+$: 382.0624, Found 382.0630.

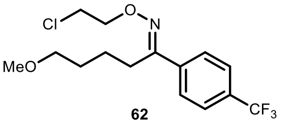

62

Compound **62** was prepared on a 0.20 mmol scale from the triphenylpyridinium salt of fluvoxamine and KI via a modified general procedure **E** where DMA was used as a solvent to give 23.7 mg (35%) of a tan solid after purification with EtOAc/hexanes.

Note: The triphenylpyrylium salt was used because fluvoxamine is supplied as the maleate salt which when subjected to these reaction conditions generated the corresponding ester.

R_f = 0.75 in 20:80 EtOAc:hexanes

^{1}H NMR (499 MHz, cdcl$_3$) δ 7.74 (d, *J* = 7.8 Hz, 2H), 7.62 (d, *J* = 8.2 Hz, 2H), 4.41 (t, *J* = 5.9 Hz, 2H), 3.78 (t, *J* = 5.8 Hz, 2H), 3.38 (t, *J* = 6.5 Hz, 1H), 3.32 (s, 3H), 2.81 (t, *J* = 7.3 Hz, 2H), 1.80 – 1.45 (m, 4H).

^{13}C NMR (126 MHz, cdcl$_3$) δ 158.41, 138.86, 131.13, 130.87, 126.08 (q, *J* = 197.7 Hz), 125.40 (q, *J* = 3.8 Hz), 122.90, 73.74, 72.23, 58.57, 42.27, 29.51, 26.26, 23.09.

^{19}F NMR (470 MHz, cdcl$_3$) δ -62.79.

HRMS (ESI) Calculated $C_{15}H_{20}F_3ClNO_2^+$ [M+H]$^+$: 338.1129, Found 338.1135.

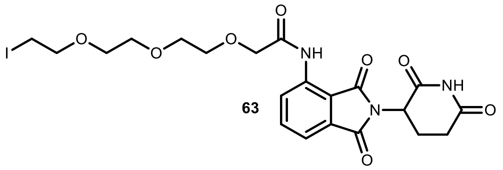

63

Compound **63** was prepared on a 0.05 mmol scale from 2-(2-(2-(2-Aminoethoxy)ethoxy)ethoxy)-*N*-(2-(2,6-dioxopiperidin-3-yl)-1,3-dioxoisoindolin-4-yl)acetamide hydrochloride, and KI via a modified general procedure **E** where TPP$^+$ BF4$^-$ was replaced with 2,4,6-tris(4-(trifluoromethyl)phenyl)pyrylium tetrafluoroborate to give 23.0 mg (80%) of a white solid after purification with EtOAc.

^{1}H NMR (499 MHz, CDCl$_3$) δ 10.46 (s, 1H), 8.86 (d, *J* = 8.4 Hz, 1H), 8.25 (s, 1H), 7.73 (t, *J* = 7.9 Hz, 1H), 7.58 (d, *J* = 7.3 Hz, 1H), 4.96 (dd, *J* = 12.4, 5.4 Hz, 1H), 4.22 (s, 2H), 3.87 – 3.79 (m, 5H), 3.73 (t, *J* = 6.8 Hz, 2H), 3.71 – 3.68 (m, 2H), 3.68 – 3.62 (m, 2H), 3.24 (t, *J* = 6.8 Hz, 2H), 2.98 – 2.87 (m, 1H), 2.86 – 2.70 (m, 2H), 2.21 – 2.13 (m, 1H).

^{13}C NMR (126 MHz, CDCl$_3$) δ 170.9, 169.6, 168.6, 167.9, 166.9, 136.9, 136.5, 131.5, 125.4, 119.0, 116.3, 72.1, 71.7, 71.2, 70.9, 70.7, 70.3, 49.4, 31.5, 22.8, 3.1.

HRMS (ESI) Calculated $C_{21}H_{25}IN_3O_8^+$ [M+H]$^+$: 574.0681, Found 574.0688.

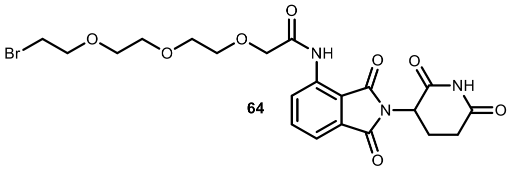

64

Compound **64** was prepared on a 0.05 mmol scale from 2-(2-(2-(2-Aminoethoxy)ethoxy)ethoxy)-*N*-(2-(2,6-dioxopiperidin-3-yl)-1,3-dioxoisoindolin-4-yl)acetamide hydrochloride, and KI via a modified general procedure **E** where TPP$^+$BF$_4^-$ was replaced with 2,4,6-tris(4-(trifluoromethyl)phenyl)pyrylium tetrafluoroborate to give 23.0 mg (80%) of a white solid after purification with EtOAc.

^{1}H NMR (499 MHz, $CDCl_3$) δ 10.47 (s, 1H), 8.87 (d, *J* = 8.4 Hz, 1H), 8.32 (s, 1H), 7.74 (t, *J* = 7.9 Hz, 1H), 7.59 (d, *J* = 7.3 Hz, 1H), 4.97 (dd, *J* = 12.4, 5.3 Hz, 1H), 4.22 (s, 2H), 3.83 (q, *J* = 1.7 Hz, 4H), 3.80 (t, *J* = 6.2 Hz, 3H), 3.75 (t, *J* = 5.8 Hz, 1H), 3.73 – 3.70 (m, 3H), 3.70 – 3.66 (m, 3H), 3.62 (t, *J* = 5.8 Hz, 1H), 3.46 (t, *J* = 6.2 Hz, 2H), 2.96 – 2.71 (m, 4H), 2.21 – 2.13 (m, 1H).

^{13}C NMR (126 MHz, $CDCl_3$) δ 170.8, 169.4, 168.4, 167.9, 166.8, 136.8, 136.3, 131.4, 125.3, 118.8, 116.1, 71.6, 71.4, 71.2, 71.1, 70.71, 70.69, 70.6, 70.5, 49.2, 42.8, 31.4, 30.4, 22.7.

HRMS (ESI) Calculated $C_{21}H_{25}BrN_3O_8^+$ [M+H]$^+$: 526.0820, Found 526.0833.

Computational Details

Reaction pathways were evaluated using the double-ended Growing String Method (GSM) reaction discovery tool, developed in the Zimmerman group.[4] GSM identifies minimum energy reaction paths and transition states without prior knowledge of their geometries.[5,6] After obtaining an initial reaction path from GSM, transition state geometries were refined by increasing the node density near the transition region and tightening the convergence tolerance (CONV_TOL) to 0.0002.

All geometries (intermediates and transition states) were optimized using the spin-unrestricted B3LYP density functional and the 6-31G* basis set.[7,18] To obtain free energy corrections at 338 K within the rigid-rotor and harmonic oscillator approximation of enthalpy and entropy, frequency calculations were conducted at the same level of theory for all stationary points. Vibrational frequencies below 50 cm^{-1} were adjusted to 50 cm^{-1}.[19,10] Single-point computations were performed using the continuum solvation model based on the solute charge density (SMD)[11] for 1,4-dioxane, the B3LYP-D3 exchange functional, and the diffuse, polarized, triple-zeta quality 6-311++G(d,p) basis set. Reported energies are from these single point energies, plus free energy corrections. All simulations were performed using Q-CHEM 5.2.[12]

Background and mechanistic hypotheses

Pairs of reagents used in our transformations have been previously used to mediate different reactions that involve carbonyl groups. For example, Devery and Zimmerman groups have shown that a Lewis acid ($FeCl_3$), in the presence of TMSCl, activates a carbonyl group through binding a silyl cation at the carbonyl oxygen rather than the Lewis acid itself (Figure 10, A).[13]

On the other hand, $B(C_6F_5)_3$ has long been known to activate Si–H bonds, in which the resulting borate can react with a carbonyl group to reduce it into an alcohol via hydride transfer (Figure 10,

B).[14] Similarly, $GaBr_3$ has been shown to be able to reduce esters into their corresponding ethers with silanes as reducing agents (Figure 10, C).[16]

Based on these precedents, the order of reagent addition (GaI_3 and $B(C_6F_5)_3$, followed by $Si(OMe)_3Cl$, then Ph_2SiH_2) and NMR studies we assumed the following :

1) Once $Si(OMe)_3Cl$ is added to GaI_3 and $B(C_6F_5)_3$, it interacts with GaI_3, forming $(MeO)_3Si^+$, analogous to Figure 10, A and supported by NMR.

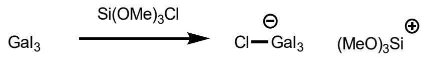

2) Ph_2SiH_2 interacts with $B(C_6F_5)_3$ which forms Ph_2HSi^+ and $H–B(C_6F_5)_3^-$ as in Figure SX B.

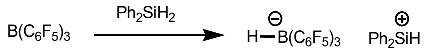

3) Silyl cations activate both substrate carbonyl groups.

4) Hydride transfer occurs from $H–B(C_6F_5)_3^-$ to one carbonyl.

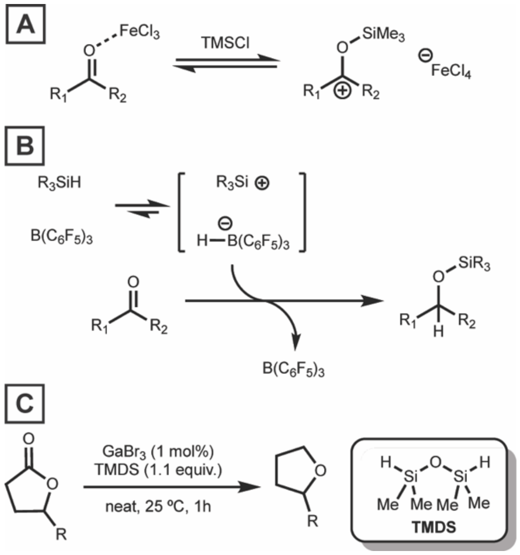

Figure S10. *Precedents of carbonyl group transformations mediated by analogous reagents used in our reaction. (A) Carbonyl activation by silyl cation which was generated by Lewis-acid reacting with a silyl chloride.[15] (B) Reduction of carbonyls by $B(C_6F_5)_3$ and silanes through Si–H activation.[16] (C) Reduction of ester carbonyl with $GaBr_3$ catalyst and silane reductant.[17]*

From this mechanistic hypothesis, the plausibility of 3) was first evaluated by comparing the free energy difference between the free- and silyl-cation bound substrates. Then, barriers of hydride transfer from $H–B(C_6F_5)_3^-$ to silyl cation-activated substrate **68** were computed to gain insight into the most plausible activation mode and regioselectivity.

Substrate activation and borate complexation

As in hypotheses 1) and 2), two silyl cations form directly from the silicon reagents used in the reaction. These can either bind to the amide or ester carbonyl groups, resulting in four different possible mono- and bis-silylated complexes. To these four activated substrates, H–B$(C_6F_5)_3^-$ can approach either the amide or the ester, resulting in a total of eight activated substrate-borate complexes. Their relative free energies are compared below.

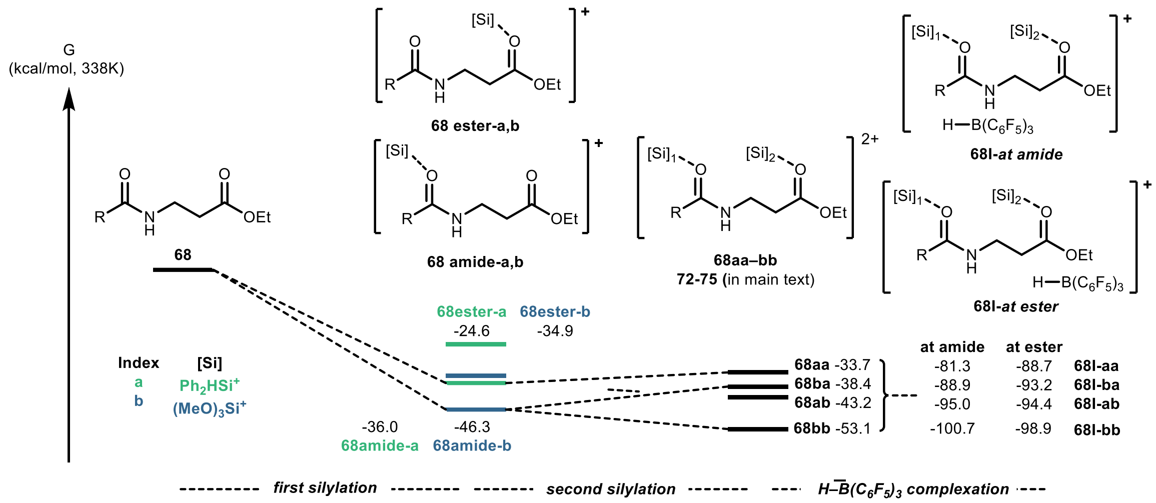

Figure S11. *Energy diagram of silyl cation-activated substrate* ***68****. Values correspond to relative free energies in kcal/mol computed at B3LYP-D3/6-311++G(d,p)/SMD(1,4-dioxane)//B3LYP/6-31G* level of theory.* ***68aa****,* ***68ab****,* ***68ba*** *and* ***68bb*** *correspond to* ***72****,* ***73****,* ***74*** *and* ***75*** *in the main text, respectively.*

By binding to a lone pair, silyl cations can stabilize substrate **68** by 25-46 kcal/mol. Due to the higher partial charge of the carbonyl oxygen of the amide than the ester, the degree of stabilization is greater when the cation first binds to the amide carbonyl (-46.3 vs -34.9 kcal/mol from blue bars and -36.0 vs. -24.6 kcal/mol from green bars when the binding silyl cations are $(MeO)_3Si^+$ and Ph_2SiH^+, respectively). Compared to the first binding, the benefit of a second silyl cation binding to the remaining carbonyl is smaller, due to penalty in entropy. When the second binding cation is Ph_2SiH^+, the complexes are destabilized regardless of the first binding cation (**68aa** vs. **68amide-a** and **68ba** vs **68amide-b**) while the opposite is true for $(MeO)_3Si^+$ (**68ab** vs. **68amide-a** and **68bb** vs **68amide-b**). Specifically, **68bb**, which has $(MeO)_3Si^+$ binding at both carbonyls, is the most thermodynamically stable. This suggests that out of the four bis-silylated substrates, **68bb** is the most likely entity to be present during the reaction.

The hydride source H–B$(C_6F_5)_3^-$ then can be electrostatically attracted to the activated substrates, further stabilizing the overall system. Substrates with Ph_2SiH^+ bound to the ester showed greater stabilization than when the borate was placed near the ester than the amide (by 7.4 and 4.3 kcal/mol for **68I-aa** and **68I-ba**, respectively). The opposite was the case when $(MeO)_3Si^+$ is activating the ester although the difference in free energy was smaller (favoring the amide by 0.6 and 1.8 kcal/mol for **68I-ba** and **68I-bb**, respectively). Overall, introducing the borate into the

system decreases the energy difference between complexes and thus necessitates the comparison of the barriers of hydride transfer in these possibilities.

Hydride transfer

With activated carbonyls, complexes **68I-aa~bb** can undergo hydride transfer from H–B(C_6F_5)$_3^-$, which is generated from the reaction between B(C_6F_5)$_3$ and Ph_2SiH_2 as described in 2) above. To evaluate the selective reduction of **68I**'s ester over amide, barriers of hydride transfer to each functional group were simulated.

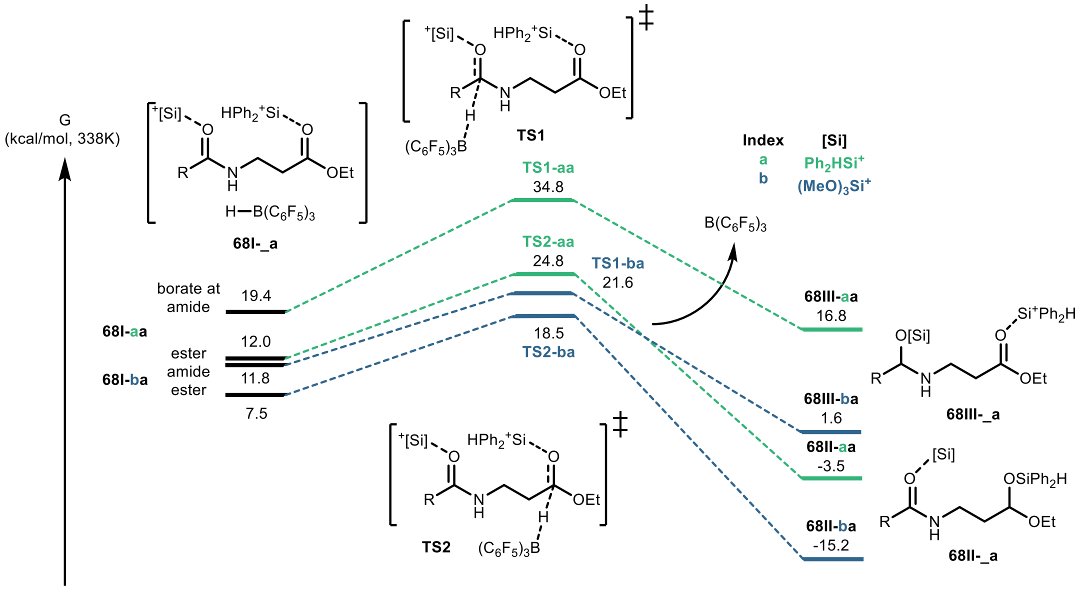

Figure S12. *Energy diagram of bis-silylated complexes **68I-aa** and **68I-ba**, which have Ph_2SiH^+ binding at the ester carbonyl, undergoing hydride transfer from H–B$^-$(C_6F_5)$_3$. The values shown are relative to the most stable complex **68I-bb** at amide (Figure11). a and b correspond to complexes with Ph_2SiH^+ and $(MeO)_3Si^+$, respectively, whereas the first and second indices denote binding to amide and ester, respectively.*

First, **68I-aa** and **68I-ba**, which have Ph_2SiH^+ bound to the ester carbonyl, were considered (Figure 12). With respect to the most stable complex from Figure 11, hydride transfer to the ester was more viable than to the amide both kinetically (**TS2** vs. **TS1**) and thermodynamically (**68II** vs **68III**) for both **68I-aa** and **68I-ba**. The lowest barrier compared to **68I-bb** was 18.5 kcal/mol for the reduction of the ester from **68I-ba**. As complexes with $(MeO)_3Si^+$ at the ester (**68I-ab** and **68I-bb**) have more energetically favorable starting points, whether hydride transfer to these complexes could result in lower barriers needed to be evaluated.

Accordingly, hydride transfer to two other bis-silylated complexes, **68I-ab** and **68I-bb** were analogously computed next (Figure 13). Similar to the previous observation (except for the starting points of **68I-bb** depending on which carbonyl the borate is located at), the pathway of hydride transfer to the amide (**TS1**) lies above that of the ester (**TS2**) for both activation modes. Compared to the previous examples, three barriers of hydride transfer except **TS1-ab** are lower with $(MeO)_3Si^+$ activating the ester (8.3~11.2 vs. 18.5 kcal/mol). This is due to the starting complexes being more thermodynamically stable (6.3 kcal/mol is the highest in Figure13 while

7.5 kcal/mol is the lowest in Figure 12), combined with notably lower barrier of hydride transfer to the ester than the previous complexes (5.5 and 6.5 kcal/mol for **TS2-ab** and **TS2-bb**, respectively, when compared to the respective starting complex, while the analogous values of **TS2-ba** and **TS2-bb** is 11.0 and 12.8 kcal/mol, respectively). These aspects suggest the importance of $(MeO)_3Si^+$ bound to the ester. The resulting complex is not only more thermodynamically favorable, but the hydride transfer processes also become kinetically more feasible than when Ph_2SiH^+ activates the ester. Together with **68bb** being the most likely mode of activation (Figure 13), **68II-bb** being the most thermodynamically stable across all possible products and the overall barrier of ester hydride transfer being the lowest, it is likely that $(MeO)_3Si^+$ is also bound to the amide.

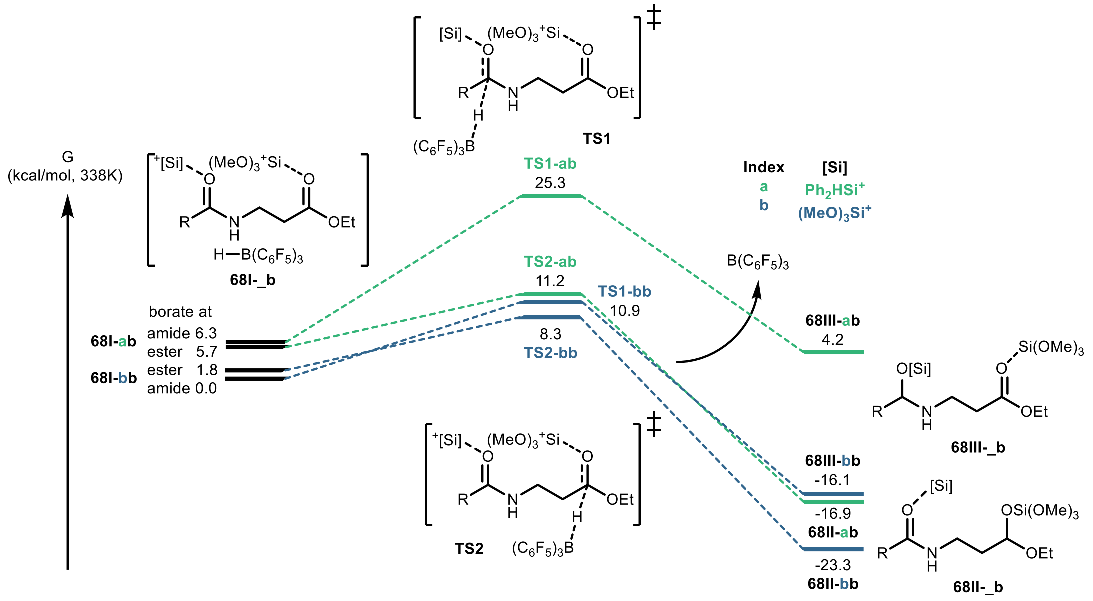

Figure S13. *Energy diagram of bis-silylated complexes **68I-ab** and **68I-bb**, which has $(MeO)_3Si^+$ binding at the ester carbonyl, undergoing hydride transfer from H–$B^-(C_6F_5)_3$. a and b correspond to complexes with Ph_2SiH^+ and $(MeO)_3Si^+$, respectively, whereas the first and second indices denote binding to amide and ester, respectively.*

In summary, $(MeO)_3Si^+$ may play an important role in substrate activation toward selective hydride transfer to the ester. Binding of $(MeO)_3Si^+$ to either carbonyl is thermodynamically beneficial compared to Ph_2SiH^+ (Figure 13). In addition, $(MeO)_3Si^+$ activating both amide and ester resulted in both the lowest overall barrier and most thermodynamically stable product. These results support the two entries in the order of addition studies where the amine product (amide being reduced over the ester) is not observed when $(MeO)_3SiCl$ is initially added with GaI_3 (entries 2 and 4 in Table 3). In addition, this complies with the detection of a peak with a mass value that corresponds to $(MeO)_3Si$–O–$SiPh_2H$ by LC/MS, which can form by Ph_2SiH^+ activating the siloxy group of **68II-bb** *en route* to the ether. However, other activation modes may also be operating as the silyl cations change throughout the reaction. This is supported by the detection of MeCl and MeI by GC/MS, multiple carbonyl peaks in the ^{13}C-NMR (Figure 4C) and observation of the amine product in some order of addition studies.

References:

1. Liao, J.; Basch, C. H.; Hoerrner, M. E.; Talley, M. R.; Boscoe,B. P.; Tucker, J. W.; Garnsey, M. R.; Watson, M. P. Deaminative Reductive Cross-Electrophile Couplings of Alkylpyridinium Salts and Aryl Bromides. *Org. Lett.* **2019**, *21*, 2941−2946.

2. Nyfeler, E.; Renaud, P. Decarboxylative Radical Azidation Using MPDOC and MMDOC Esters. *Org. Lett.* **2008**, *10*, 985−988.

3. Tsymbal, A. V.; Bizzini, L. D.; MacMillan, D. W. C. Nickel Catalysis via SH2 Homolytic Substitution: The Double Decarboxylative Cross-Coupling of Aliphatic Acids. *J. Am. Chem. Soc.* **2022**,*144*, 21278−21286.

4. a) Zimmerman, P. M. *J.* Automated discovery of chemically reasonable elementary reaction steps*. Comp. Chem*. **2013**, *34*, 1385-1392. b) Zimmerman, P. M. Navigating molecular space for reaction mechanisms: an efficient, automated procedure. *Molecular Simulation*, **2015**, *41*, 43-54.

5. Zimmerman, P. M. *J.* Single-Ended Transition State Finding with the Growing String Method*. Comp. Chem*. **2015**, *36*, 601-611.

6. a) Zimmerman, P. M. *J.* Growing string method with interpolation and optimization in internal coordinates: Method and examples. *Chem. Phys*. **2013**, *138*, 184102. b) Zimmerman, P. M. *J.* Reliable Transition State Searches Integrated with the Growing String Method. *Chem. Theory and Comp*. **2013**, *9*, 3043-3050.

7. a) Becke, A. D. Density-functional thermochemistry. III. The role of exact exchange. *J. Chem. Phys*. **1993**, *98*, 5648–5652. b) Lee, C.; Yang, W.; Parr, R. G. Development of the Colle-Salvetti correlation-energy formula into a functional of the electron density. *Phys. Rev. B*, **1988**, *37*, 785-789. c) Vosko, S. H.; Wilk, L.; Nusair, M. Accurate spin-dependent electron liquid correlation energies for local spin density calculations: a critical analysis. *Can. J. Phys*. **1980**, *58*, 1200-1211. d) Stephens, P. J.; Devlin, F. J.; Chabalowski, C. F.; Frisch, M. J. Ab Initio Calculation of Vibrational Absorption and Circular Dichroism Spectra Using Density Functional Force Fields. *J. Phys. Chem*. **1994**, *98*, 11623–11627.

8. a) Dunning, T. H.; Hay, P. J. in 'Modern Theoretical Chemistry', Vol. 3, ed. H. F. Schaefer, Plenum, New York (1977) 1. b) Hay, P. J.; Wadt, W. R. *J.* Ab initio effective core potentials for molecular calculations. Potentials for K to Au including the outermost core orbitals. *Chem. Phys*. **1985**, *82*, 299–310.

9. Chai, J. D.; Head-Gordon, M. S. Long-range corrected hybrid density functionals with damped atom–atom dispersion corrections. *Phys. Chem. Chem. Phys*, **2008**, *10*, 6615–6620.

10. a) Dunning, T. Jr. Gaussian basis sets for use in correlated molecular calculations. I. The atoms boron through neon and hydrogen. *J. Chem. Phys*. **1989**, *90*, 1007–1023. b) Balabanov, N. B.; Peterson, K. A. Systematically convergent basis sets for transition metals. I. All-electron correlation consistent basis sets for the $3d$ elements Sc–Zn. *J. Chem. Phys*. **2005**, *123*, 064107.

11. Marenich, A. V.; Cramer, C. J.; Truhlar, D. G. Universal Solvation Model Based on Solute Electron Density and on a Continuum Model of the Solvent Defined by the Bulk Dielectric Constant and Atomic Surface Tensions. *J. Chem. Phys. B*. **2009**, *113*, 6378–6396.

12. Epifanovsky, E. *et al.* Software for the frontiers of quantum chemistry: An overview of developments in the Q-Chem 5 package. *J. Chem. Phys*. **2021**, *155*, 084801.

13. Devery, J. J. *et al*. Controlling Catalyst Behavior in Lewis Acid-Catalyzed Carbonyl-Olefin Metathesis. *J. Am. Chem. Soc*. **2023**, *145*, 13069–13080.

14. Parks, D. J.; Blackwell, J. M.; Piers, W. E. Studies on the Mechanism of $B(C_6F_5)_3$-Catalyzed Hydrosilation of Carbonyl Functions. *J. Org. Chem*. **2000**, *65*, 3090–3098.

15. Biermann, U.; Metzger, J. O. Synthesis of Ethers by $GaBr_3$-Catalyzed Reduction of Carboxylic Acid Esters and Lactones by Siloxanes. *ChemSusChem,* **2013**, *7*, 644–649.

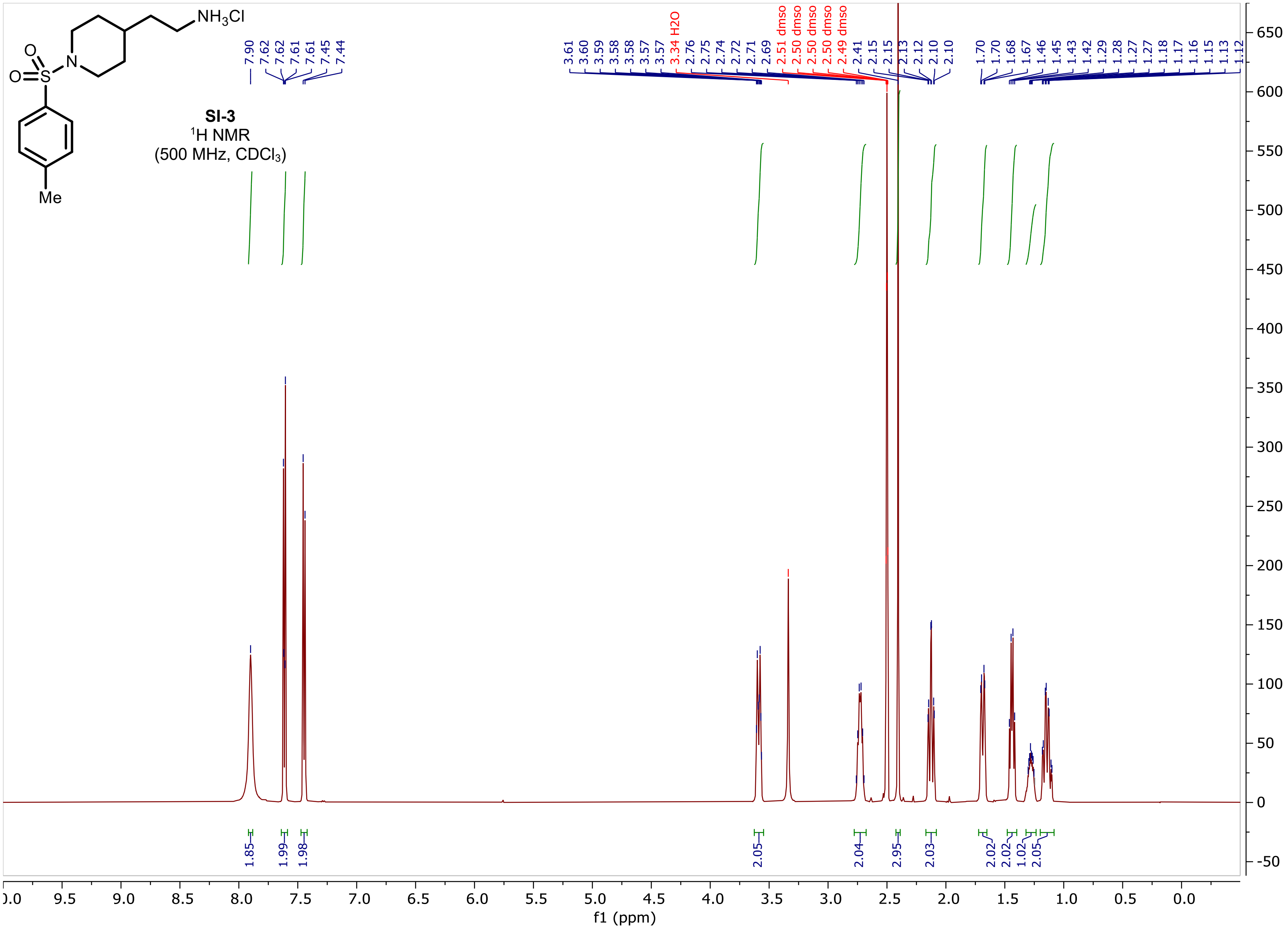
NH3Cl
O
O=S
N
Me
SI-3
1H NMR
(500 MHz, CDCl3)
f1 (ppm)

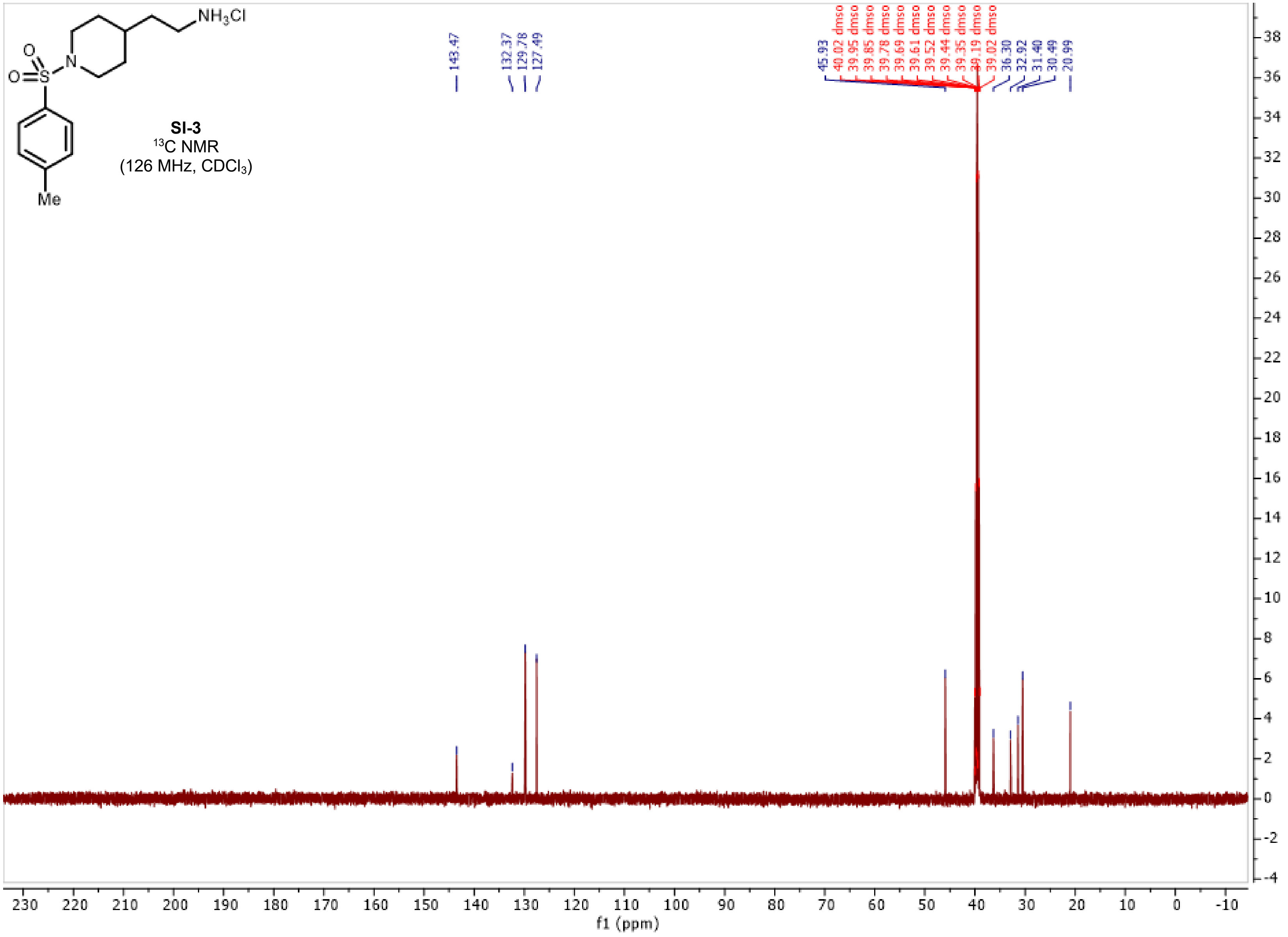
NH3Cl
SI-3
13C NMR
(126 MHz, CDCl3)
Me
143.47
132.37
129.78
127.49
45.93
40.02 dmso
39.95 dmso
39.85 dmso
39.78 dmso
39.69 dmso
39.61 dmso
39.52 dmso
39.44 dmso
39.35 dmso
39.19 dmso
39.02 dmso
36.30
32.92
31.40
30.49
20.99
f1 (ppm)

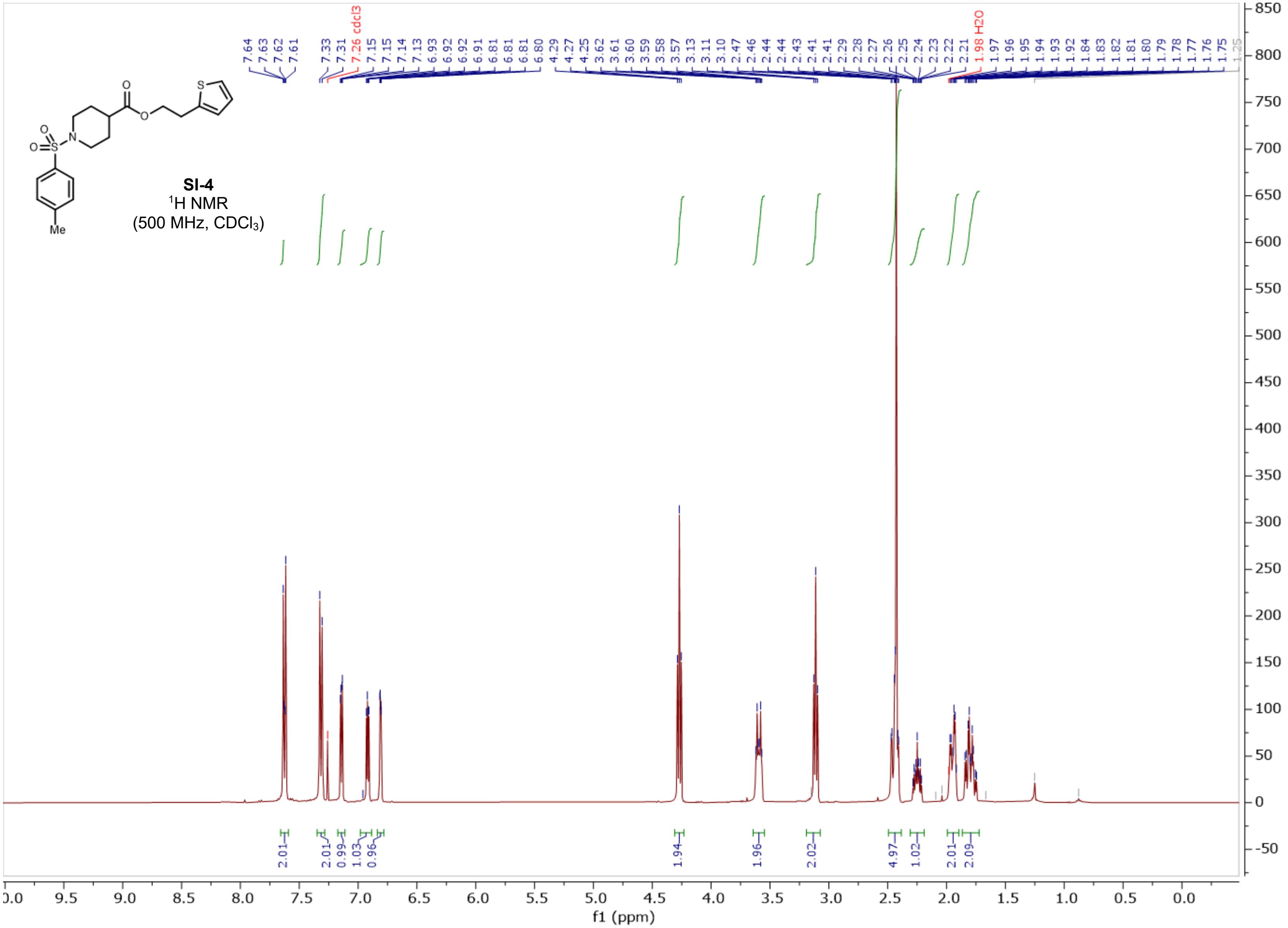
SI-4
1H NMR
(500 MHz, CDCl3)
7.64 7.63 7.62 7.61 7.33 7.31 7.26 cdcl3 7.15 7.15 7.14 7.13 6.93 6.92 6.92 6.91 6.81 6.81 6.81 6.80 4.29 4.27 4.25 3.62 3.61 3.60 3.59 3.58 3.57 3.13 3.11 3.10 2.47 2.46 2.44 2.44 2.43 2.41 2.41 2.29 2.28 2.27 2.26 2.25 2.24 2.23 2.22 2.21 1.98 H2O 1.97 1.96 1.95 1.94 1.93 1.92 1.84 1.83 1.82 1.81 1.80 1.79 1.78 1.77 1.76 1.75
2.01 2.01 0.99 1.03 0.96 1.94 1.96 2.02 4.97 1.02 2.01 2.09
f1 (ppm)

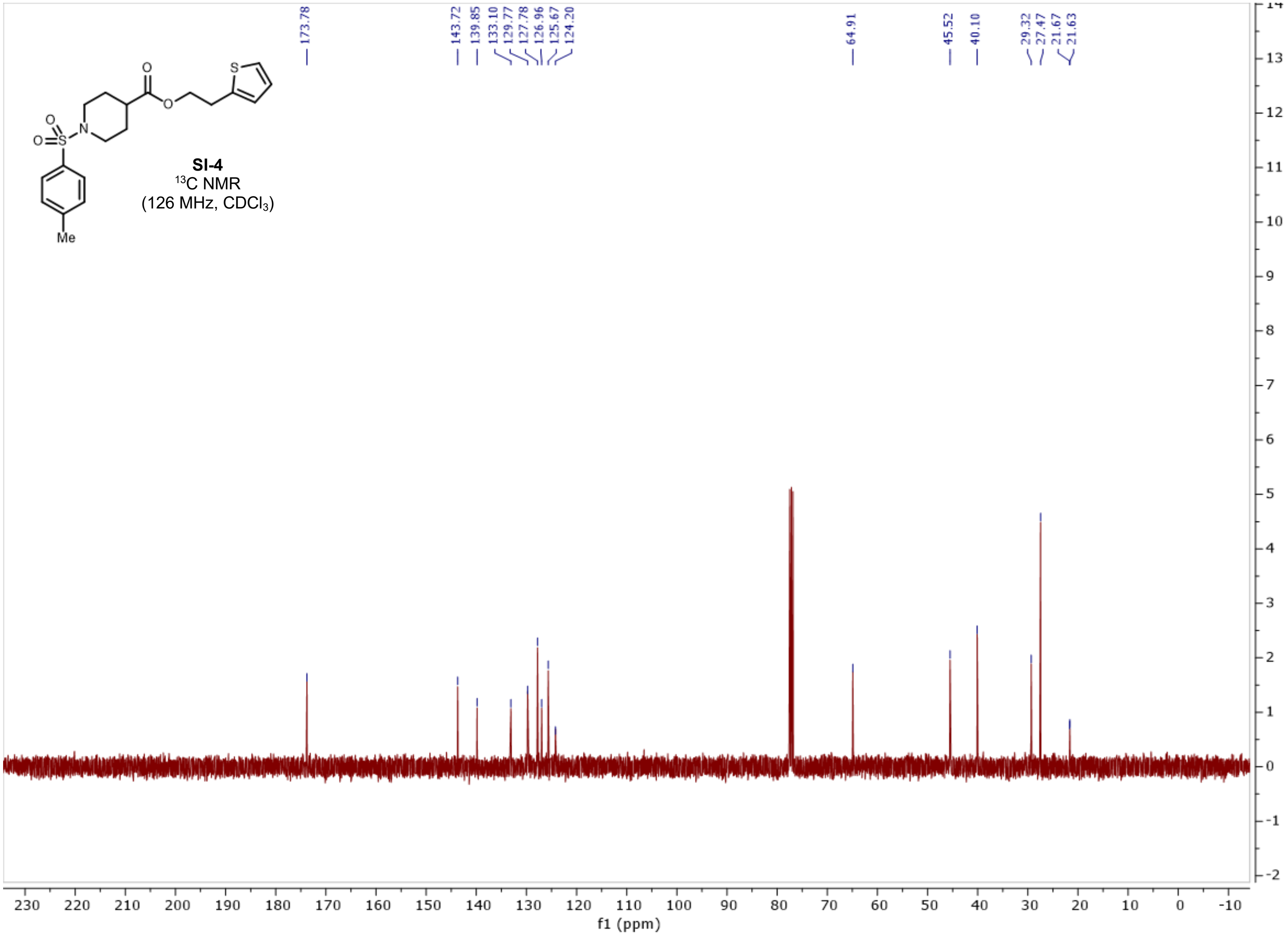

O
S
O
O=S
N
Me
SI-4
13C NMR
(126 MHz, CDCl3)
173.78
143.72
139.85
133.10
129.77
127.78
126.96
125.67
124.20
64.91
45.52
40.10
29.32
27.47
21.67
21.63
f1 (ppm)

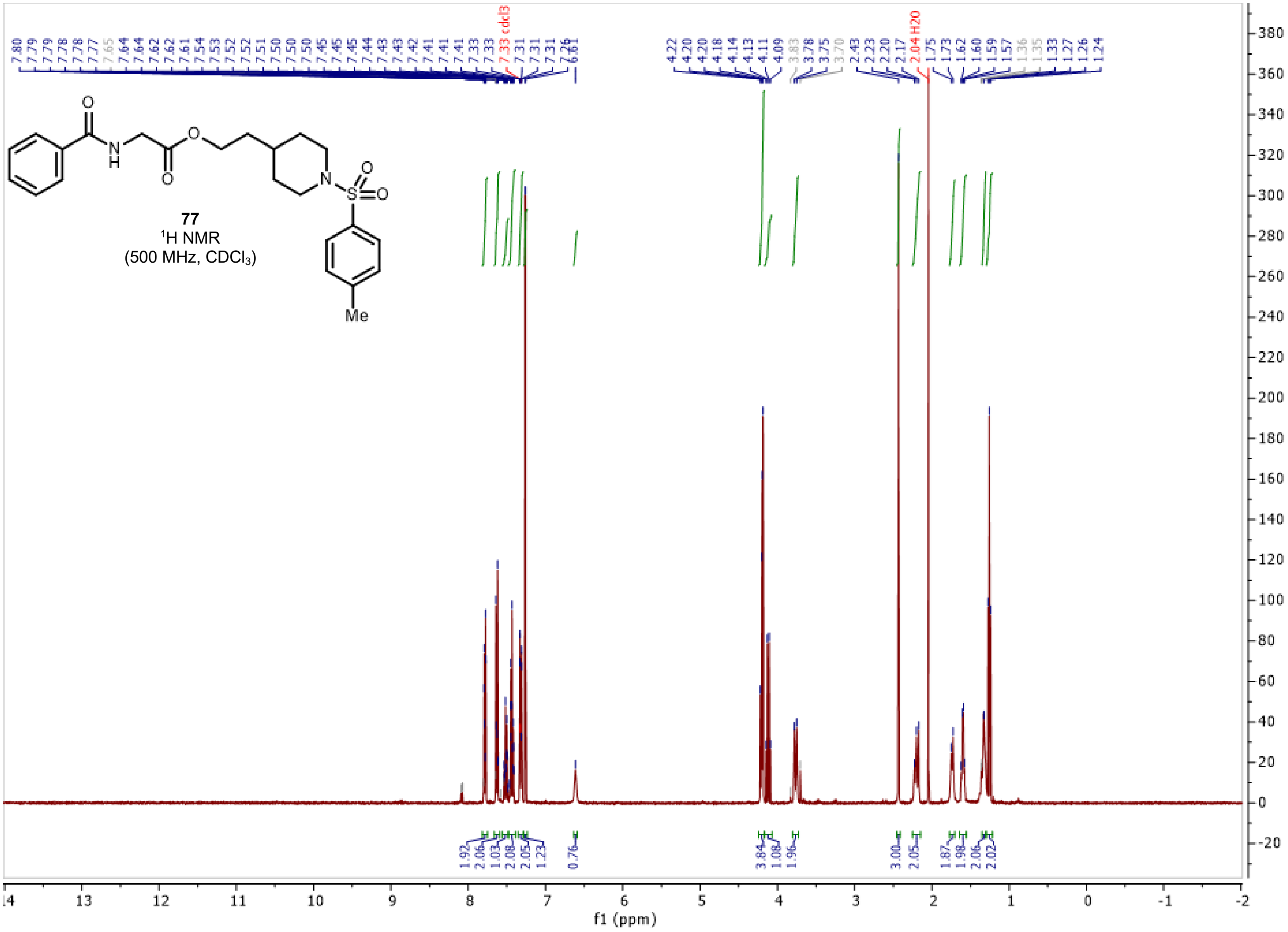
77
1H NMR
(500 MHz, CDCl3)
Me
f1 (ppm)

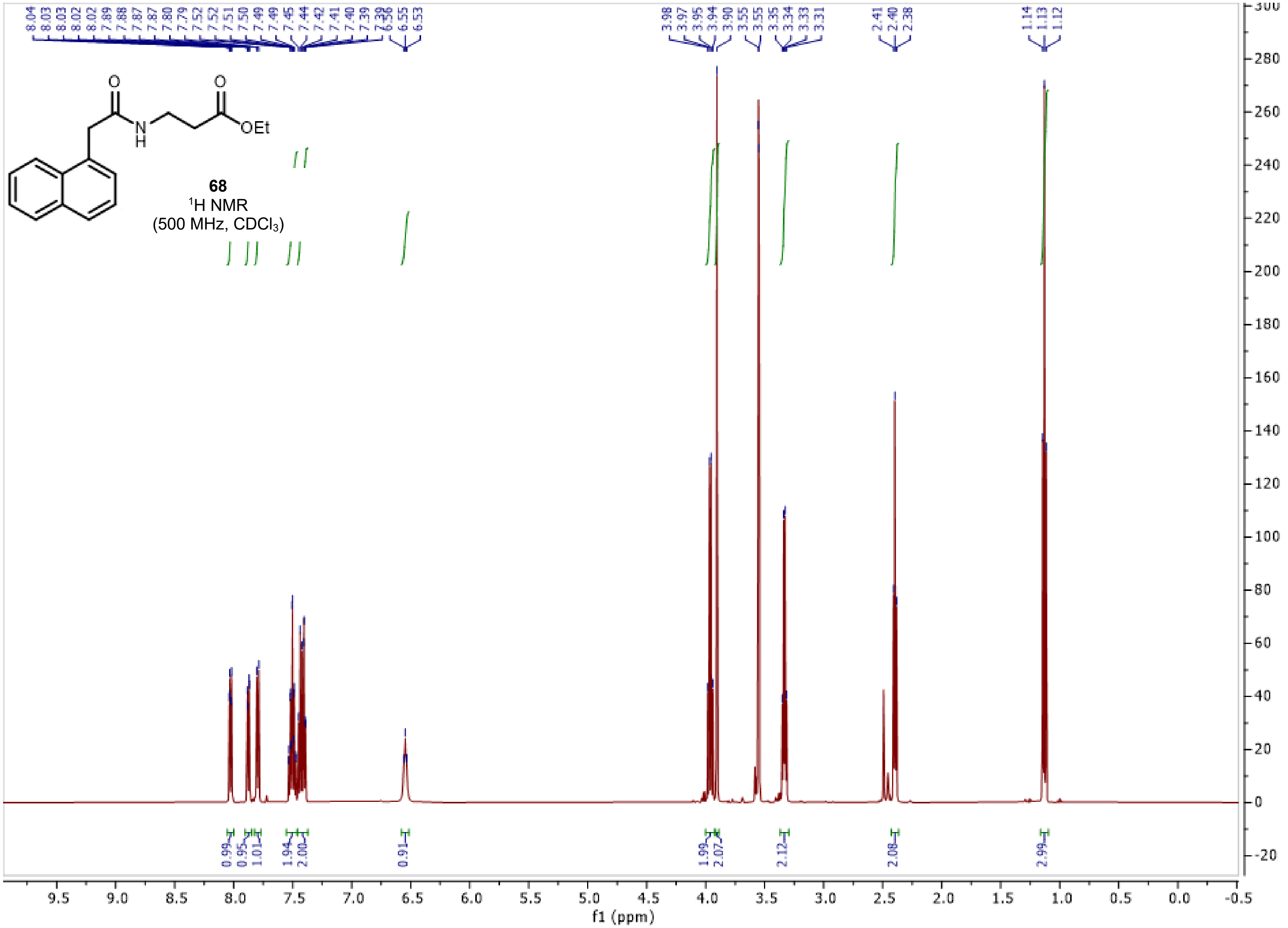
68
1H NMR
(500 MHz, CDCl3)
OEt
f1 (ppm)

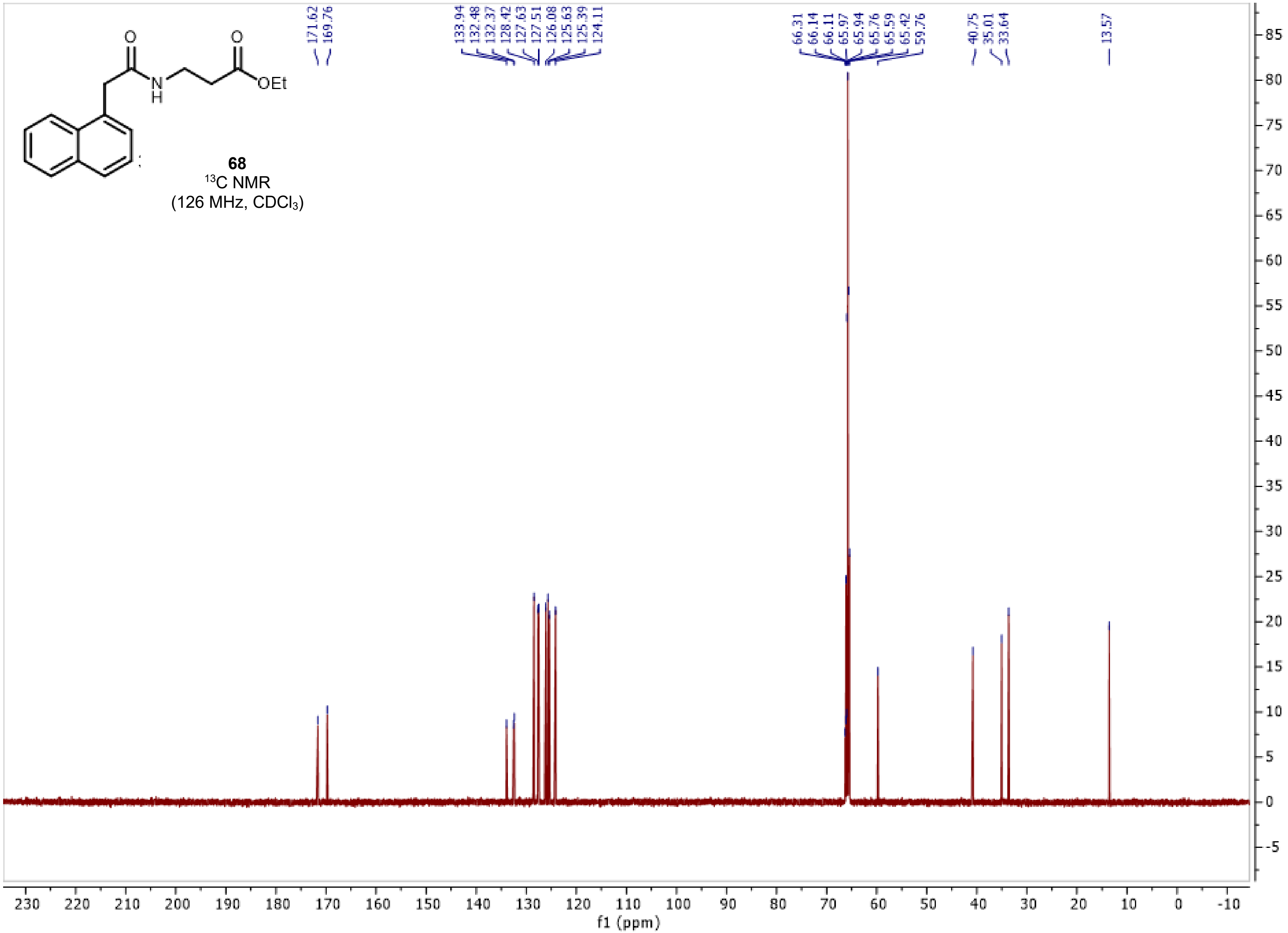

68
13C NMR
(126 MHz, CDCl3)
171.62
169.76
133.94
132.48
132.37
128.42
127.63
127.51
126.08
125.63
125.39
124.11
66.31
66.14
66.11
65.97
65.94
65.76
65.59
65.42
59.76
40.75
35.01
33.64
13.57
f1 (ppm)

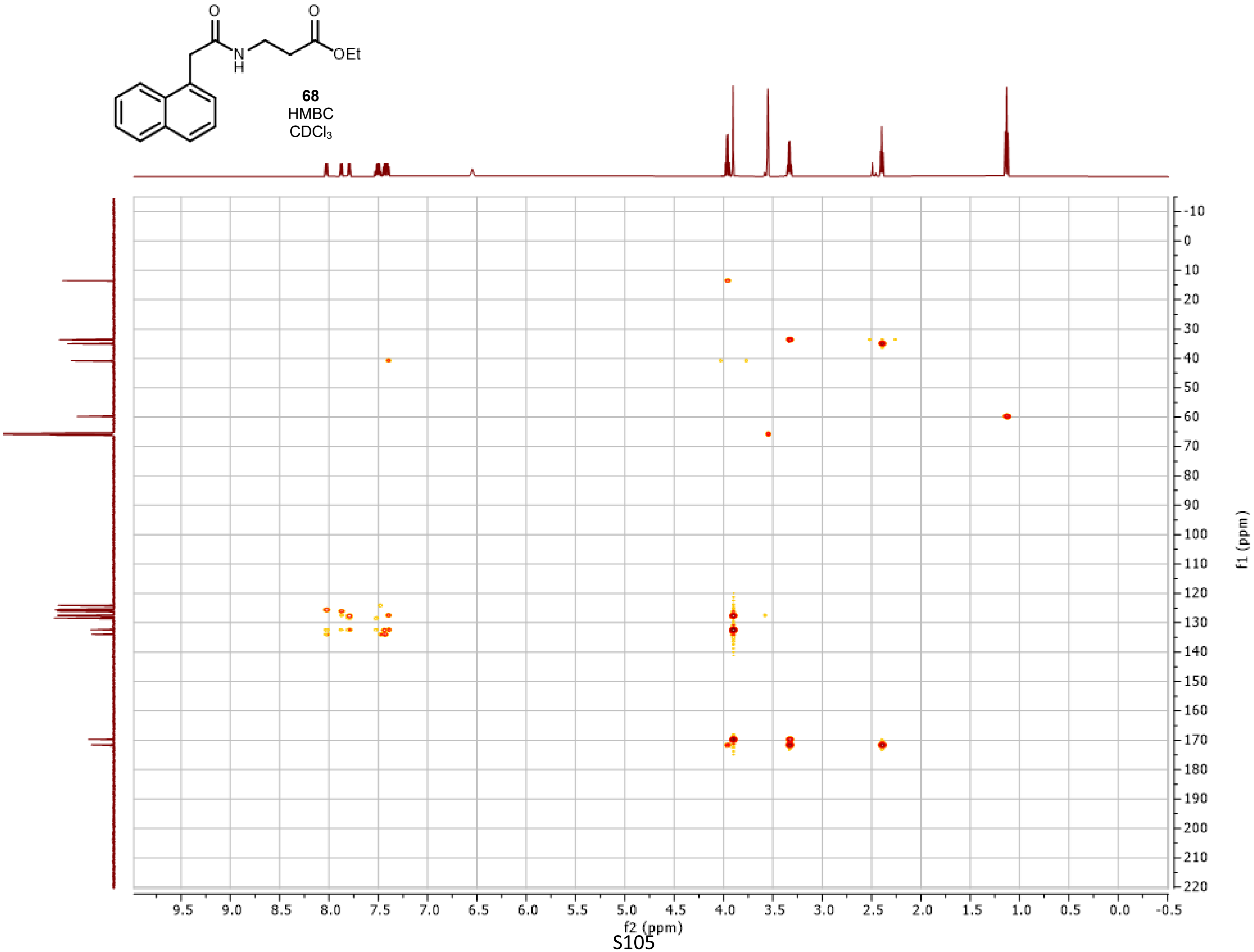
O
O
N
H
OEt
68
HMBC
CDCl3
f1 (ppm)
f2 (ppm)

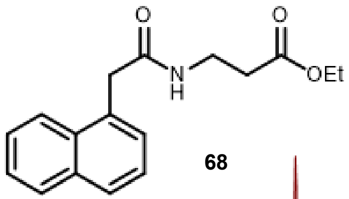

O
O
N
H
OEt

68

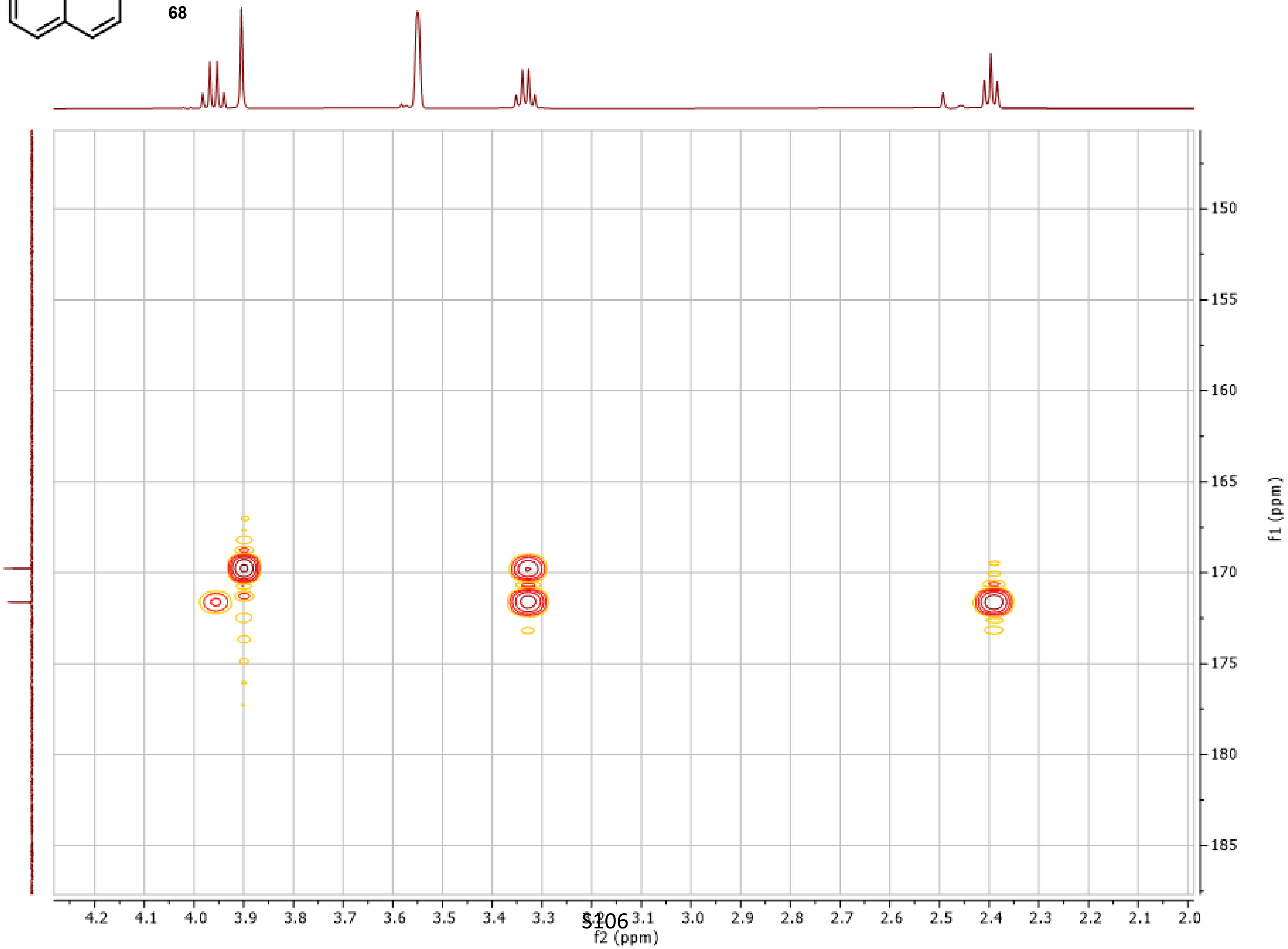

150
155
160
165
170
175
180
185
f1 (ppm)
4.2
4.1
4.0
3.9
3.8
3.7
3.6
3.5
3.4
3.3
3.1
3.0
2.9
2.8
2.7
2.6
2.5
2.4
2.3
2.2
2.1
2.0
f2 (ppm)

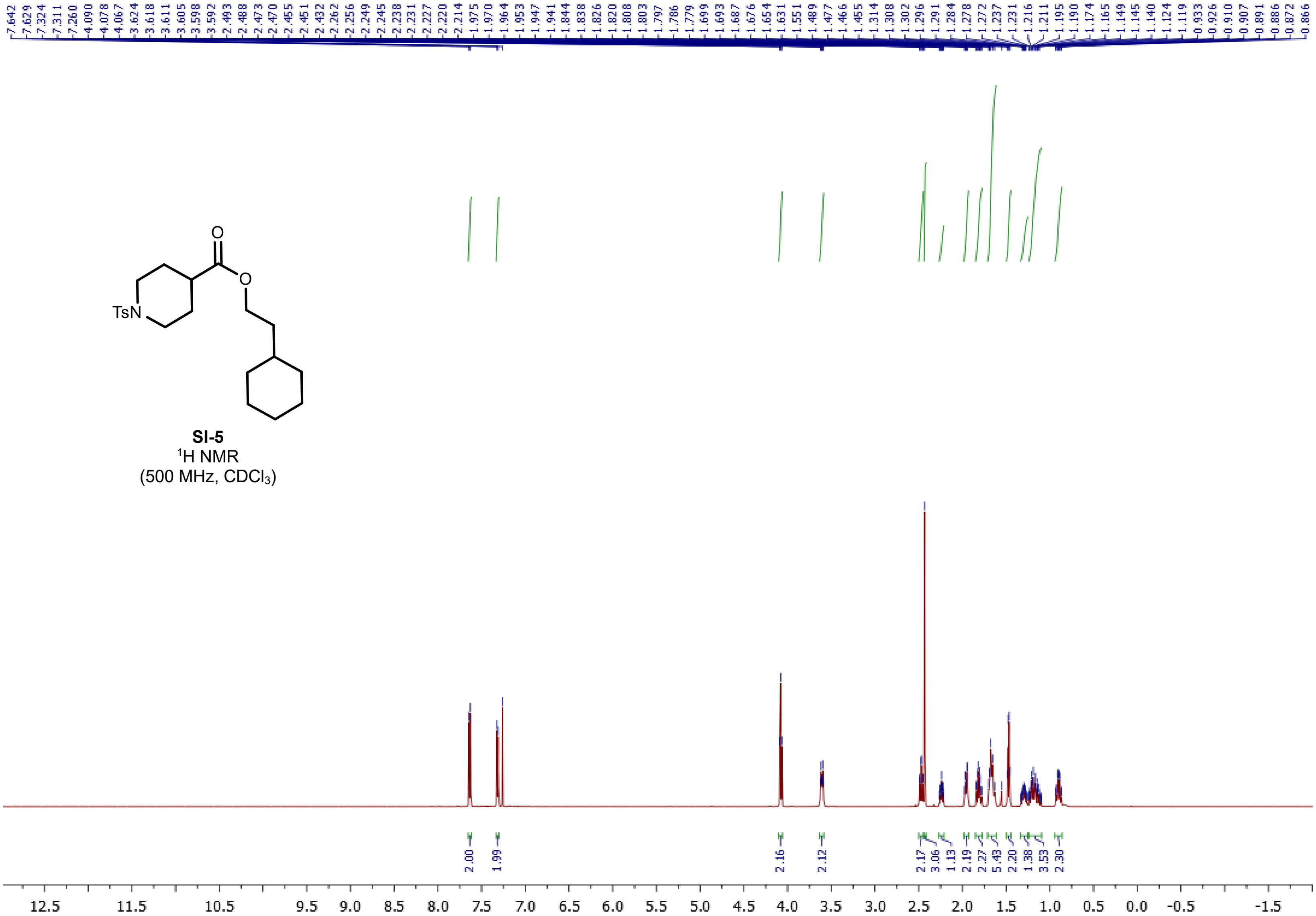

SI-5
^{1}H NMR
(500 MHz, $CDCl_3$)

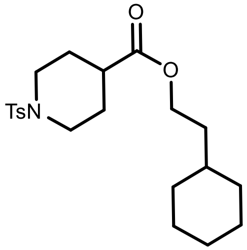

SI-5
^{13}C NMR
(125 MHz, $CDCl_3$)

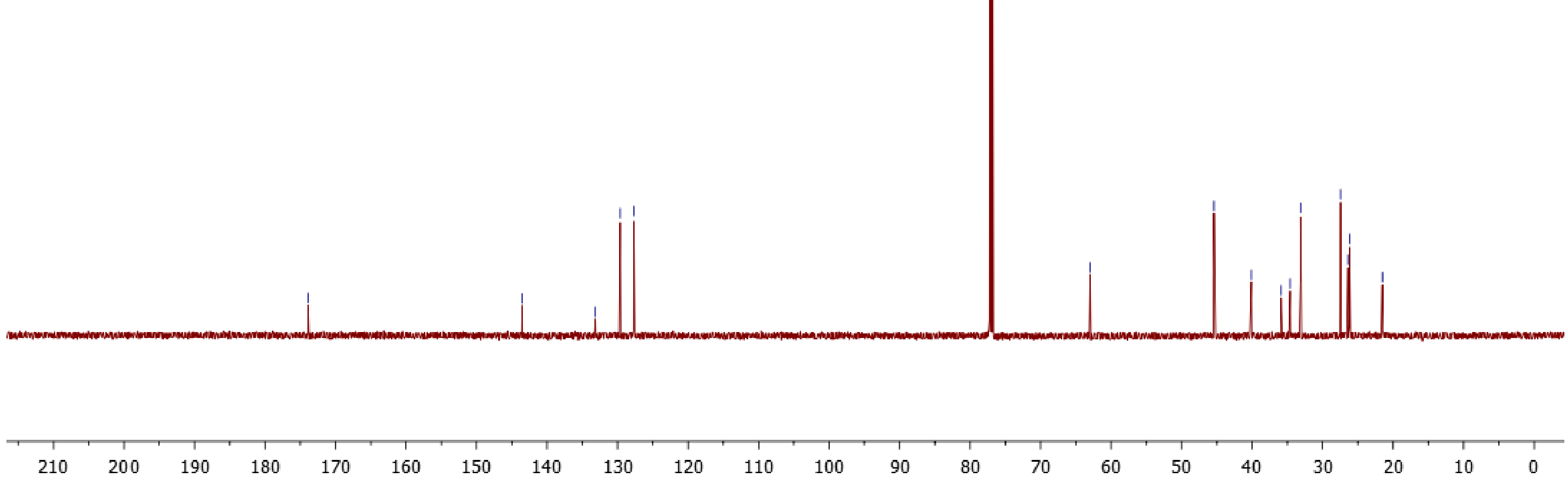

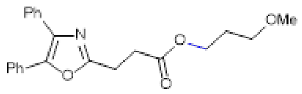

SI-6
^{1}H NMR
(500 MHz, $CDCl_3$)

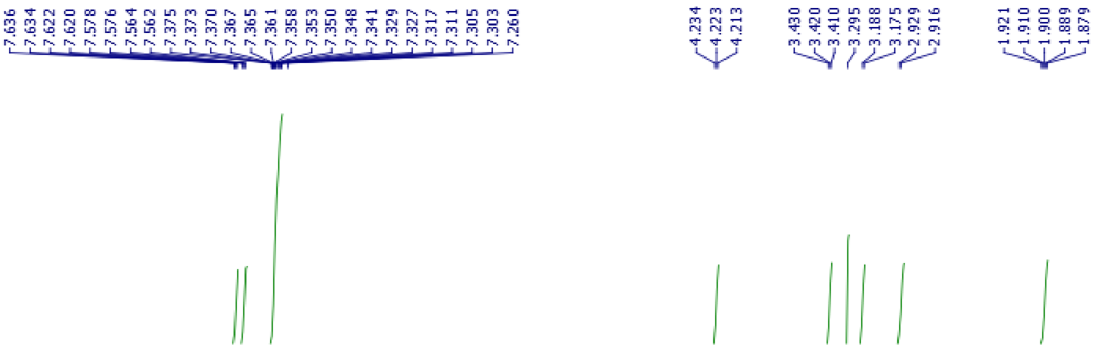

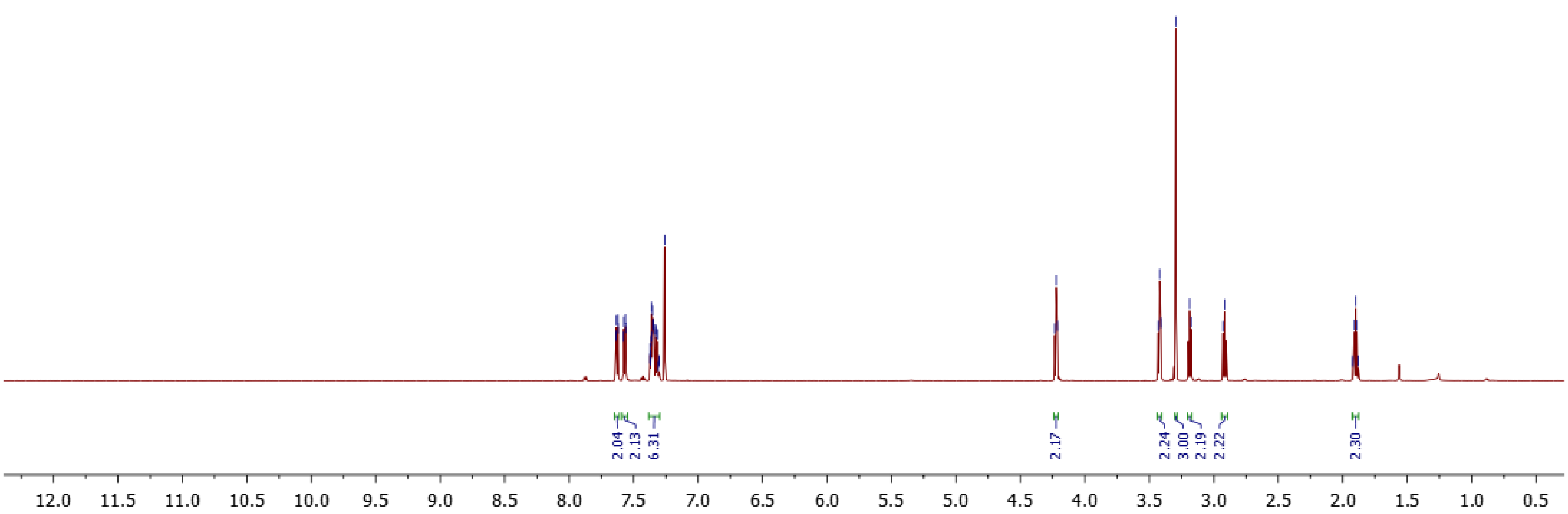

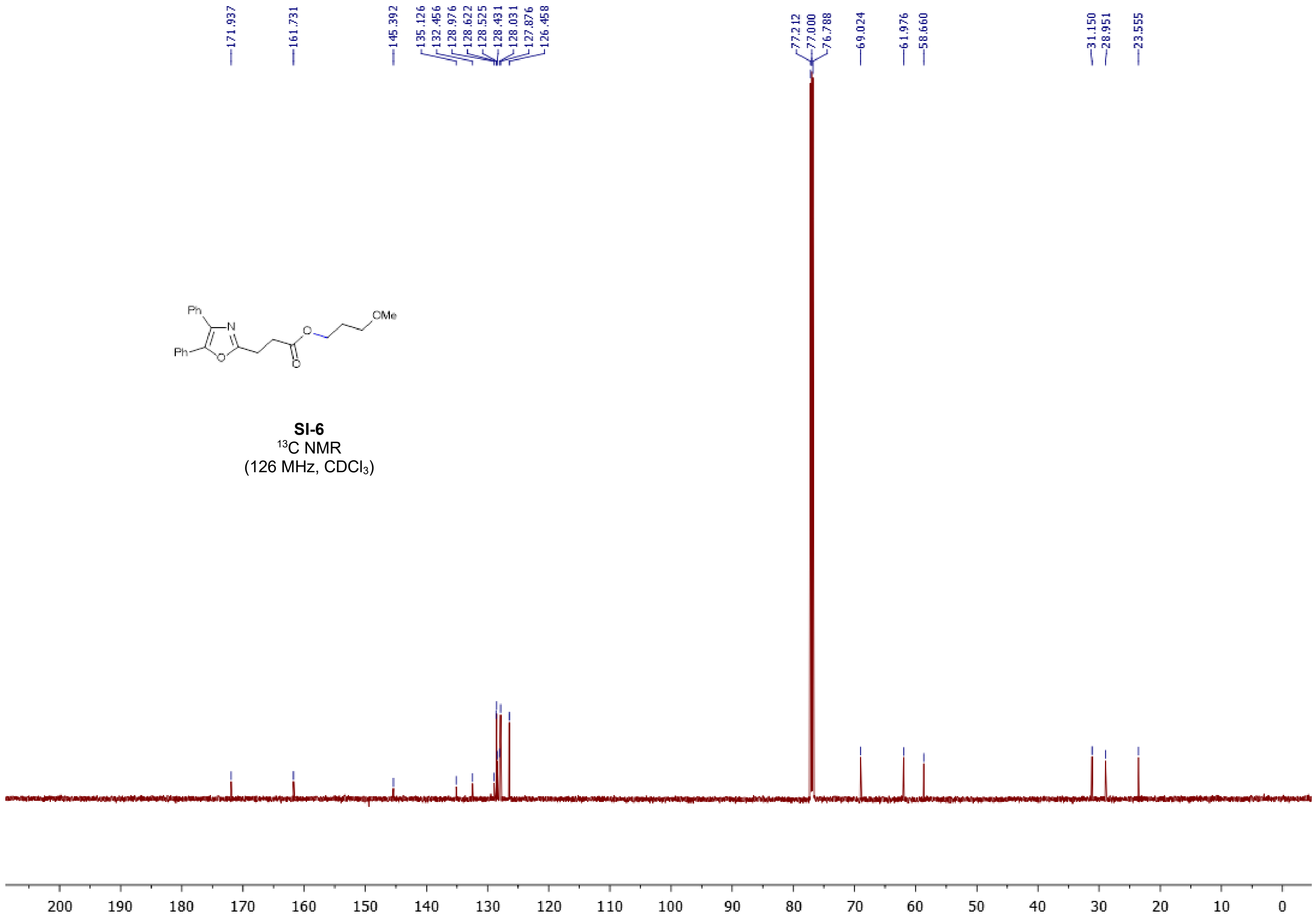

SI-6
^{13}C NMR
(126 MHz, $CDCl_3$)

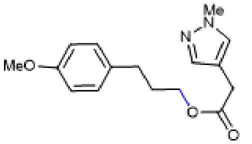

SI-7
^1H NMR
(500 MHz, $CDCl_3$)

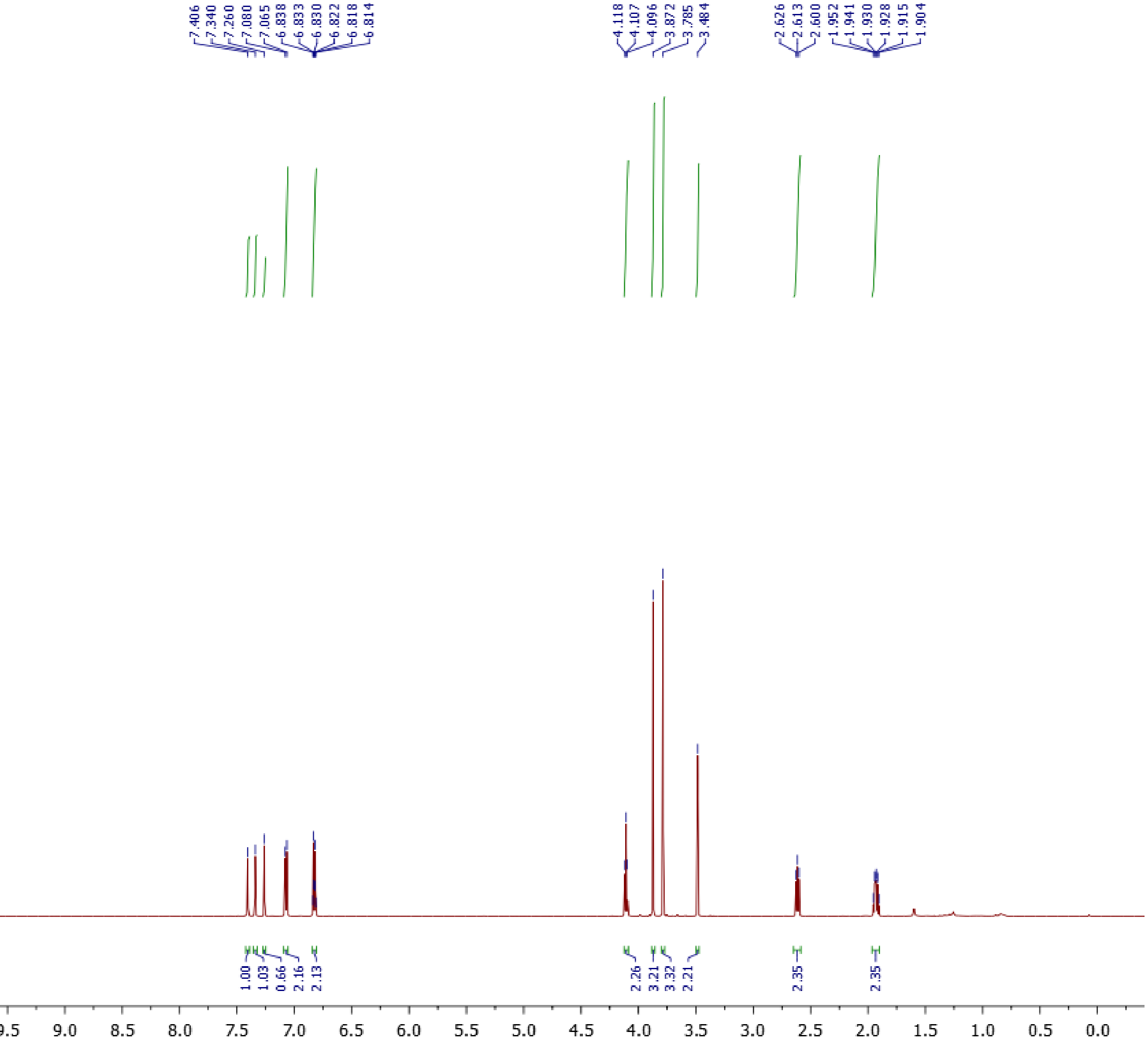

SI-8

SI-7
^{13}C NMR
(126 MHz, $CDCl_3$)

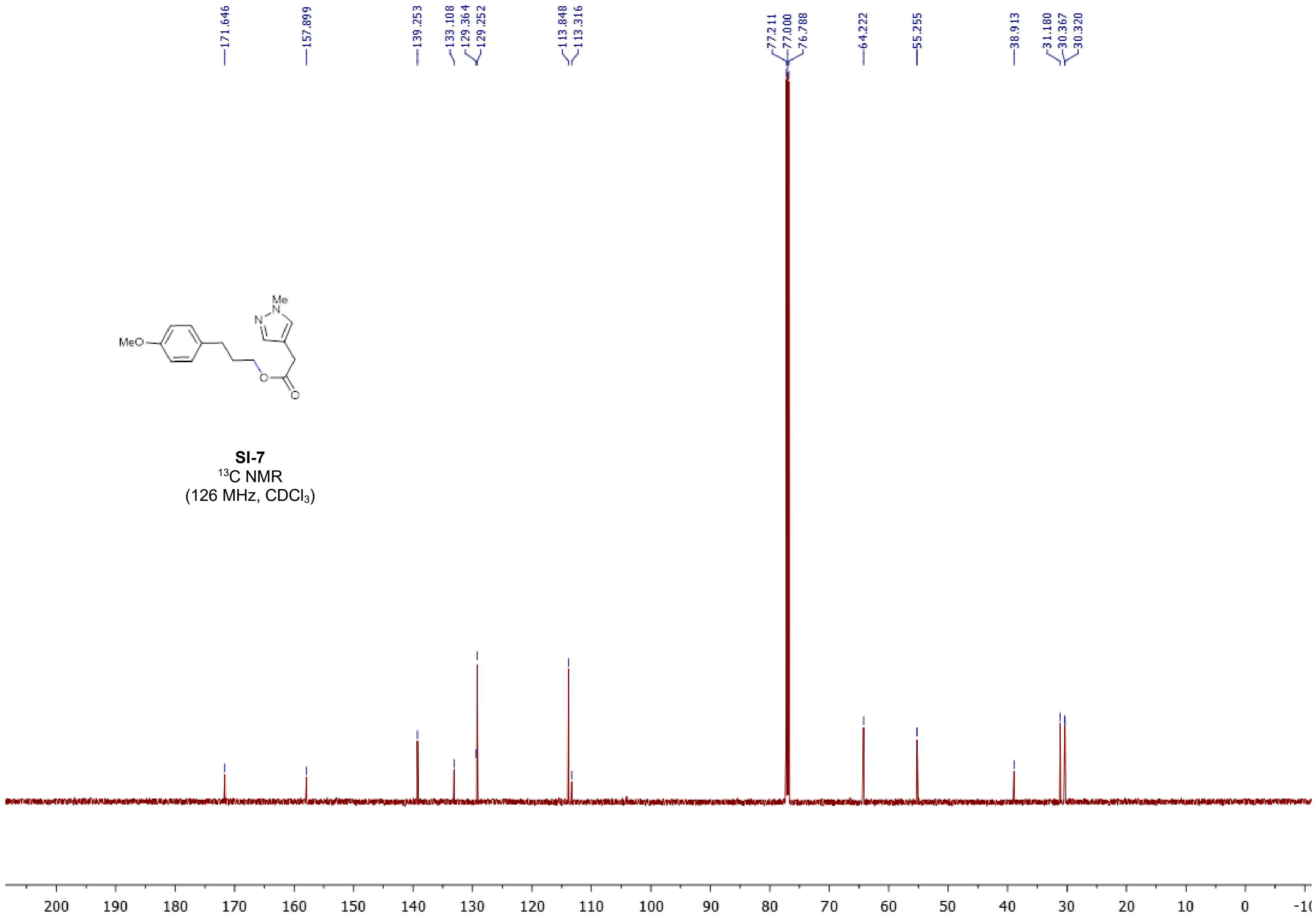

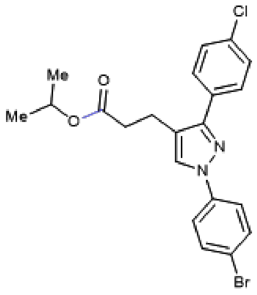

SI-8
^{1}H NMR
(500 MHz, $CDCl_3$)

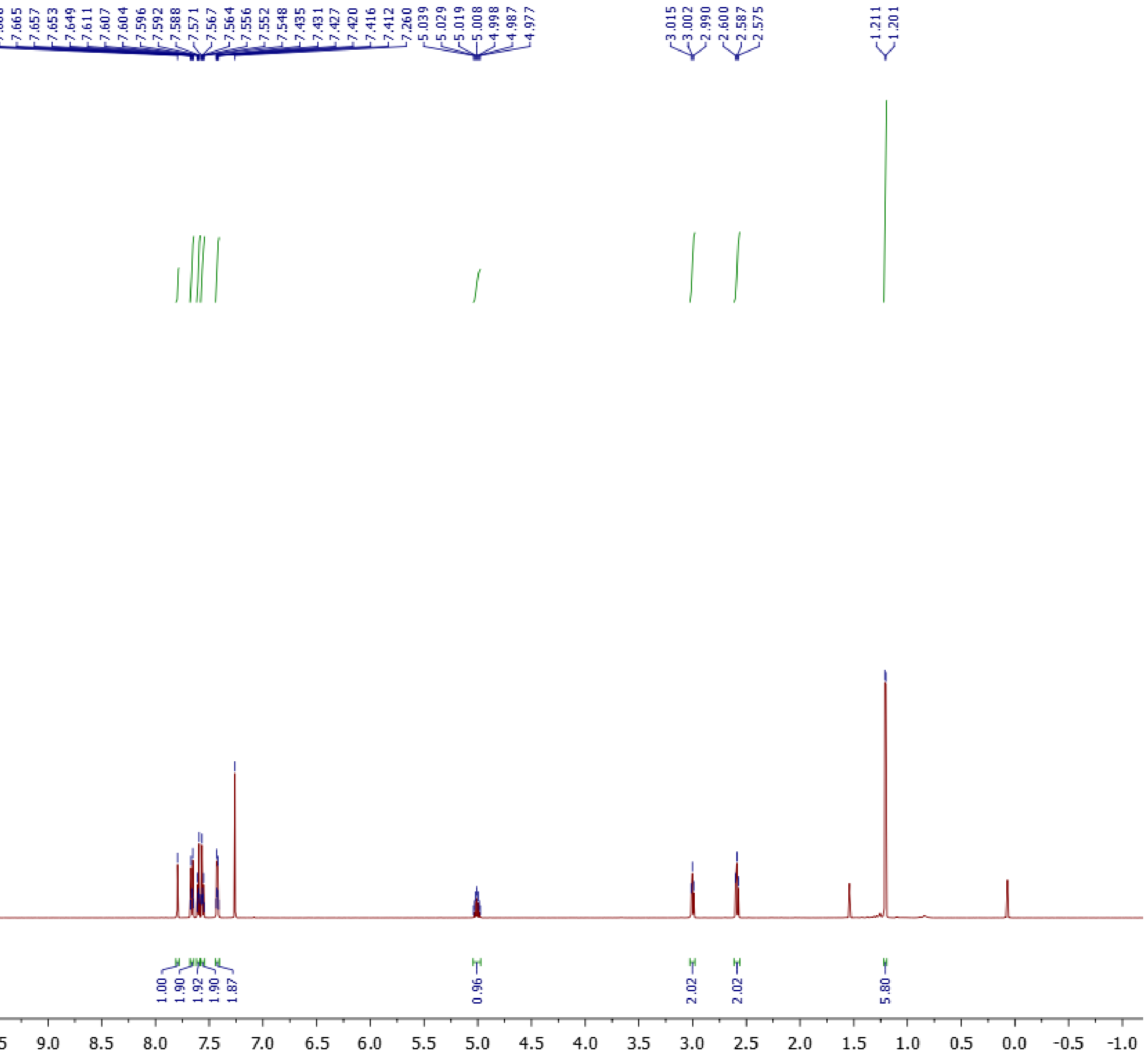

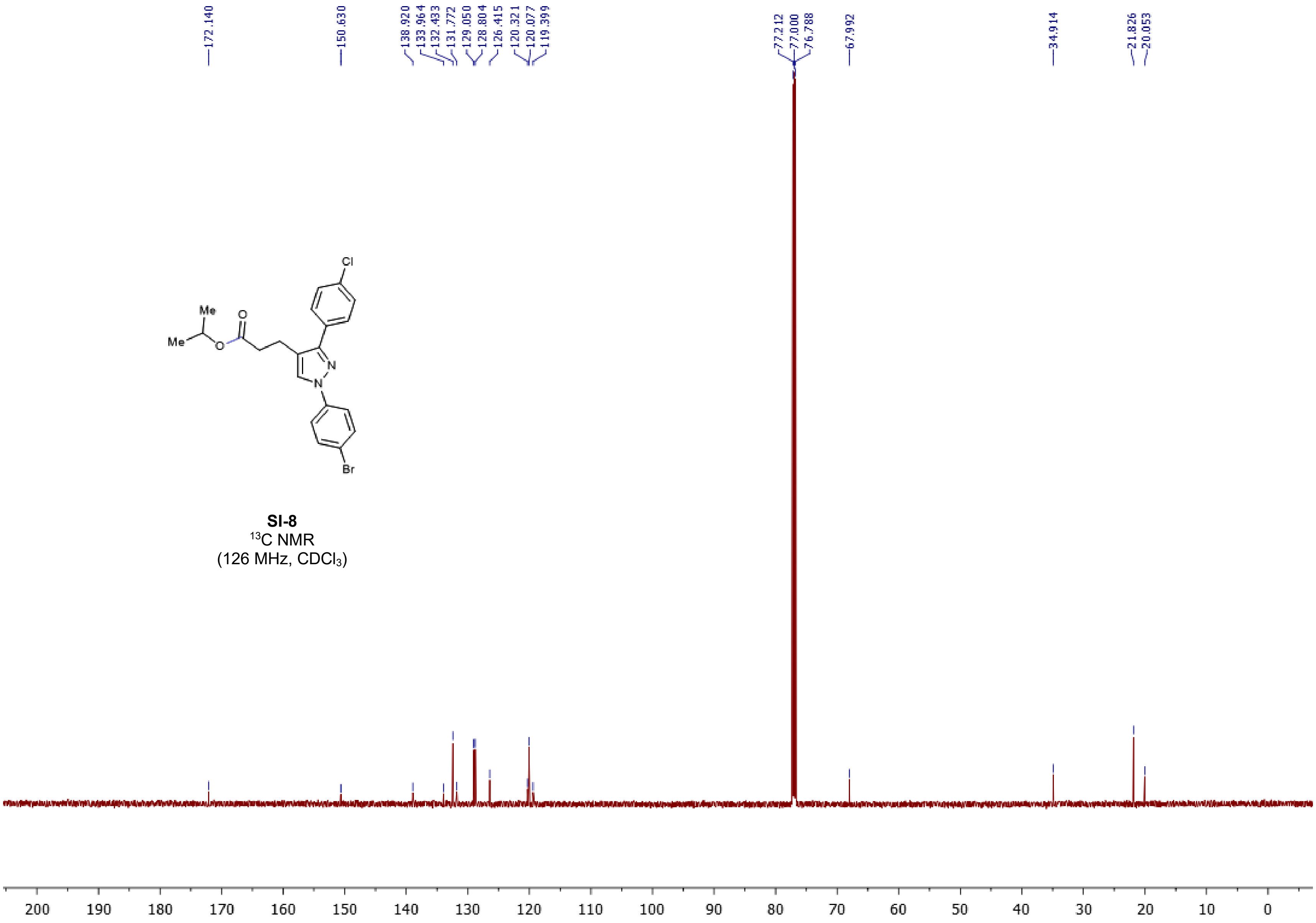

SI-8
^{13}C NMR
(126 MHz, $CDCl_3$)

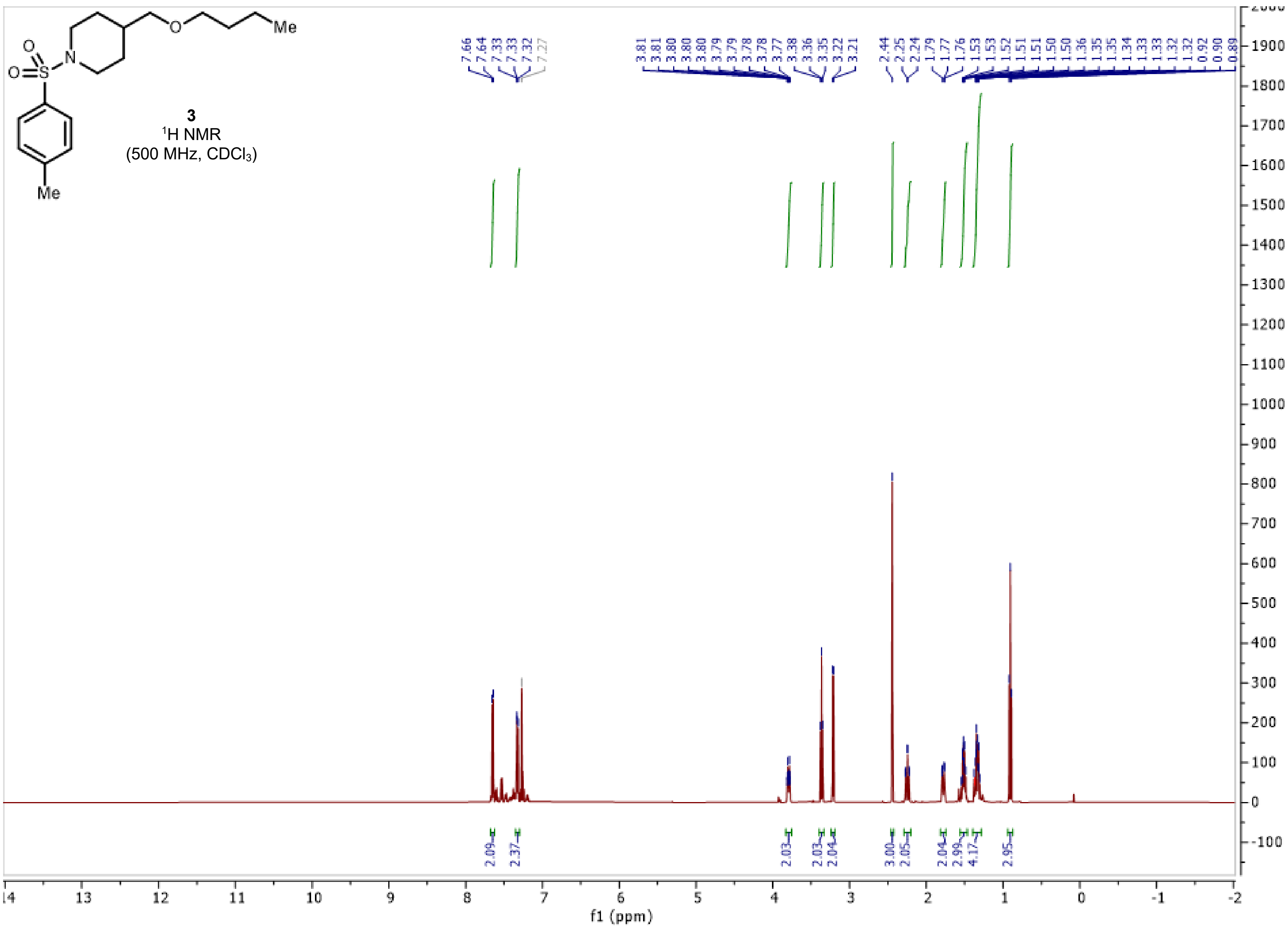
3
¹H NMR
(500 MHz, CDCl3)
f1 (ppm)

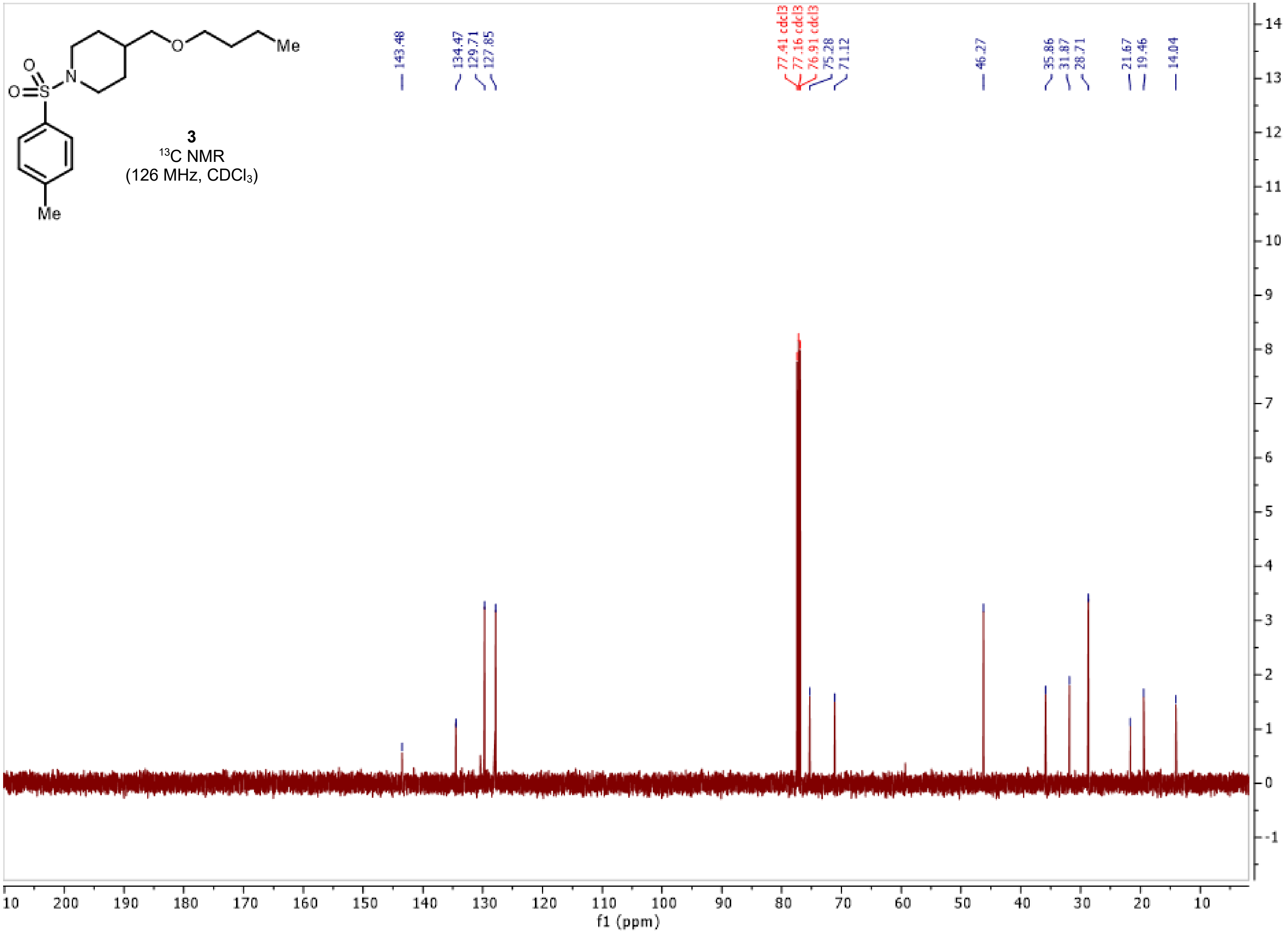
O
O
S
N
O
Me
Me
3
^{13}C NMR
(126 MHz, $CDCl_3$)
143.48
134.47
129.71
127.85
77.41 cdcl3
77.16 cdcl3
76.91 cdcl3
75.28
71.12
46.27
35.86
31.87
28.71
21.67
19.46
14.04
f1 (ppm)

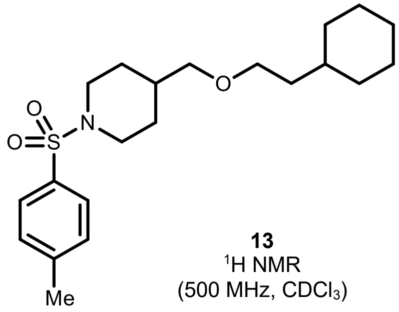

13
^{1}H NMR
(500 MHz, $CDCl_3$)

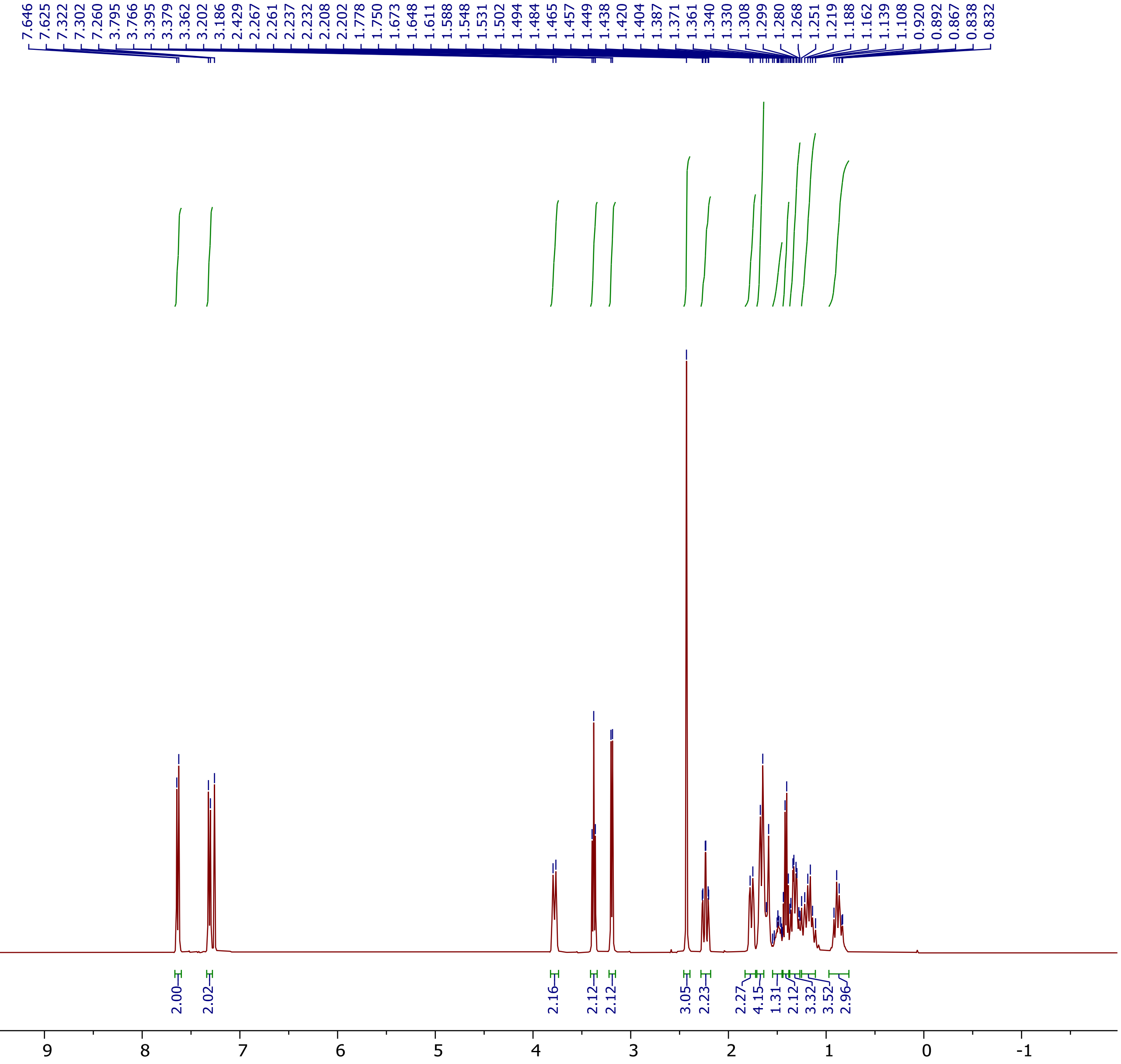

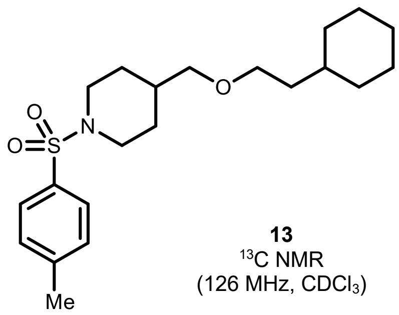

13
^{13}C NMR
(126 MHz, $CDCl_3$)

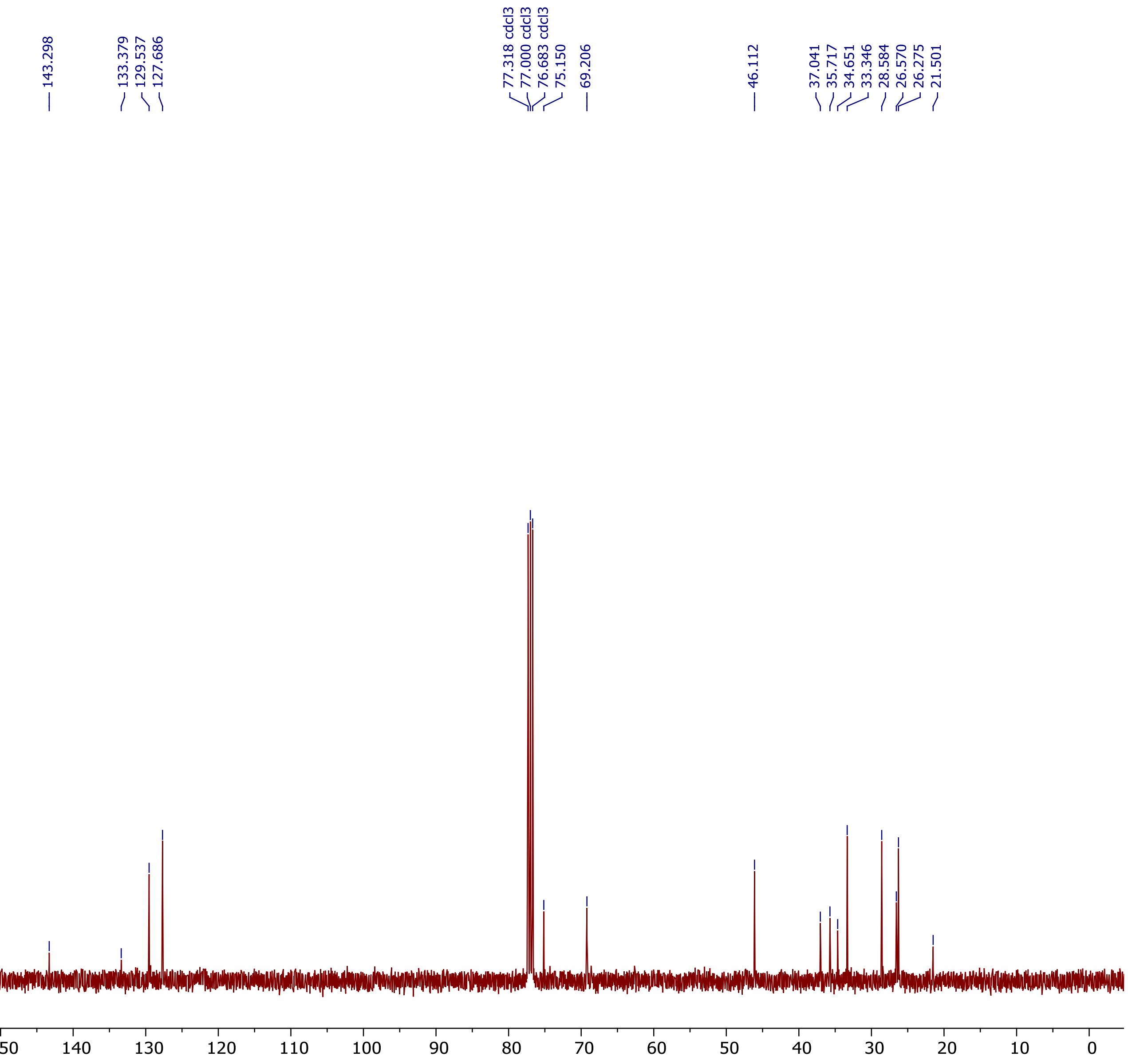

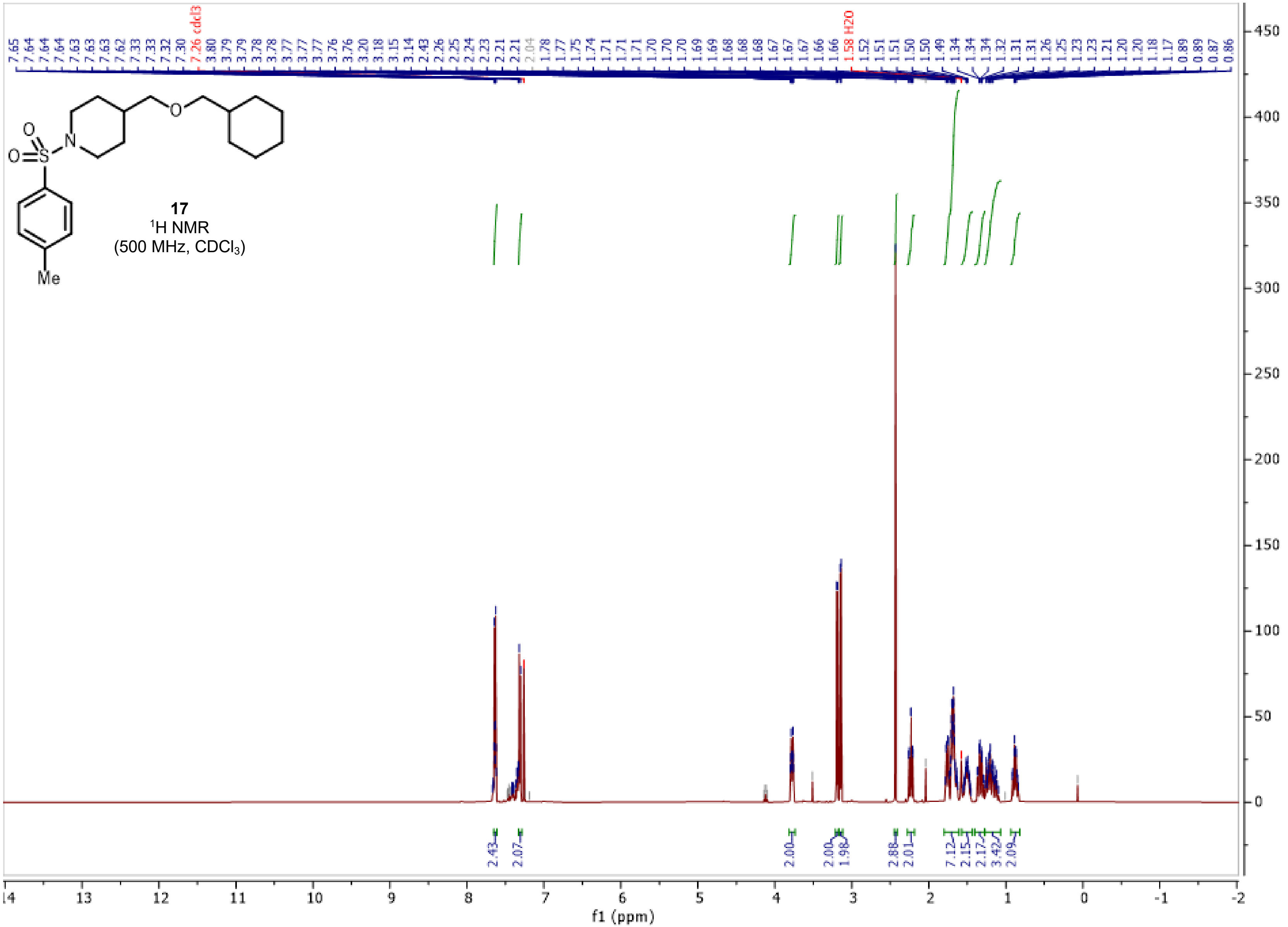
17
1H NMR
(500 MHz, CDCl3)
Me
f1 (ppm)

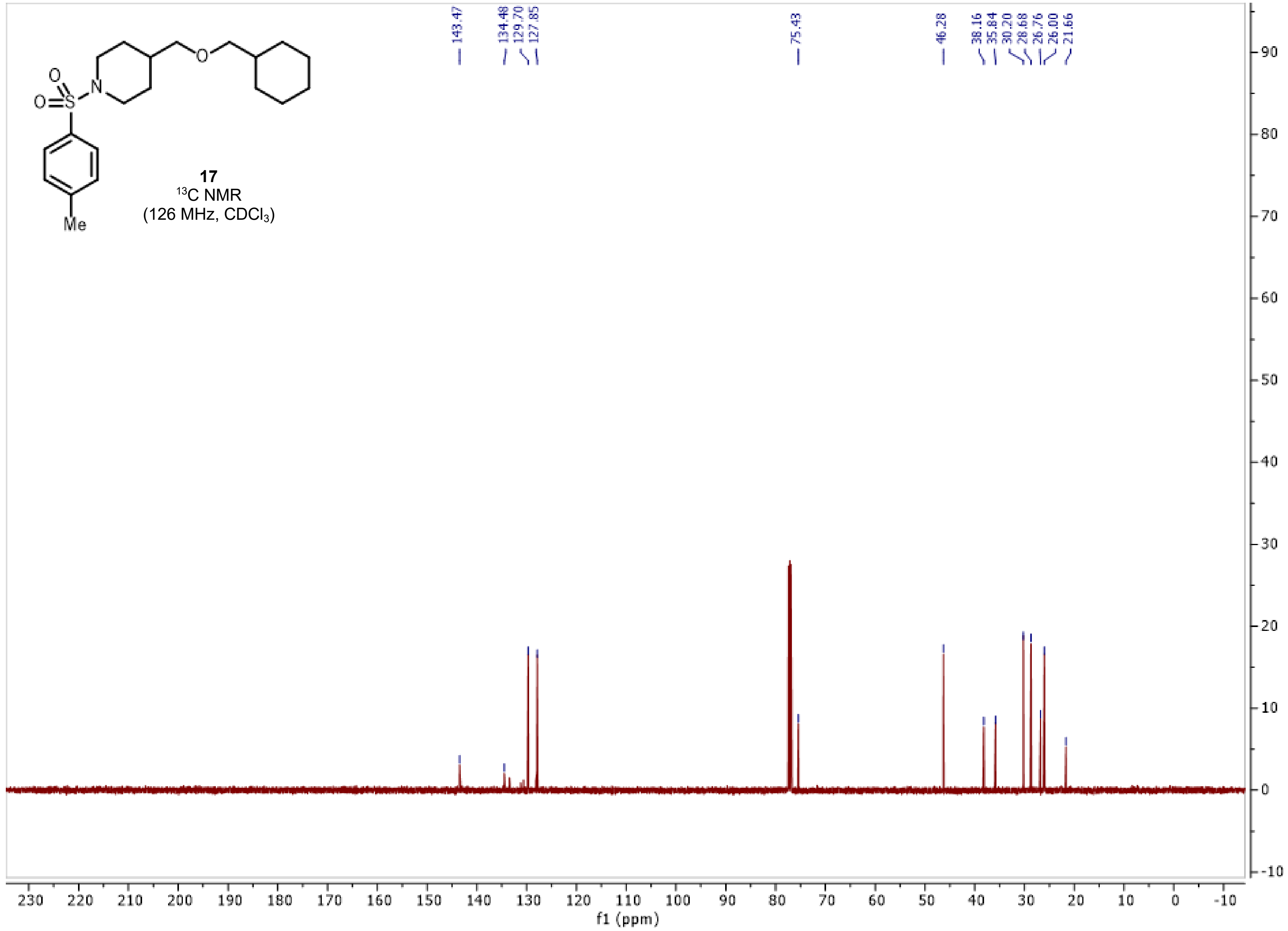
17
13C NMR
(126 MHz, CDCl3)
143.47
134.48
129.70
127.85
75.43
46.28
38.16
35.84
30.20
28.68
26.76
26.00
21.66
f1 (ppm)

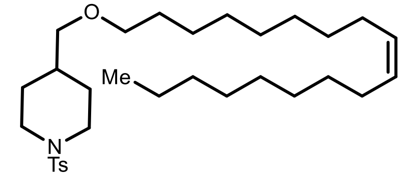

34

^{1}H NMR

(500 MHz, $CDCl_3$)

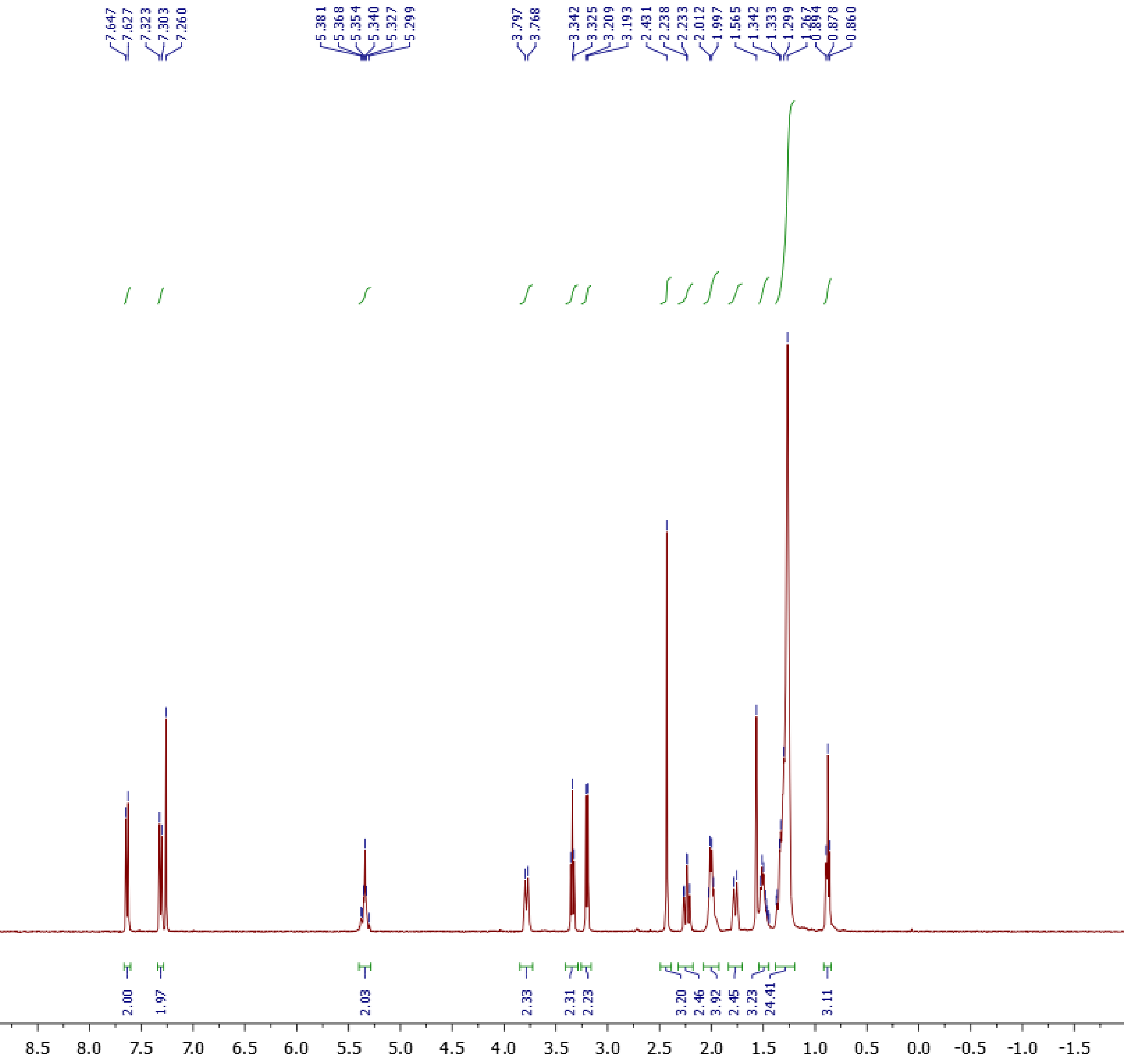

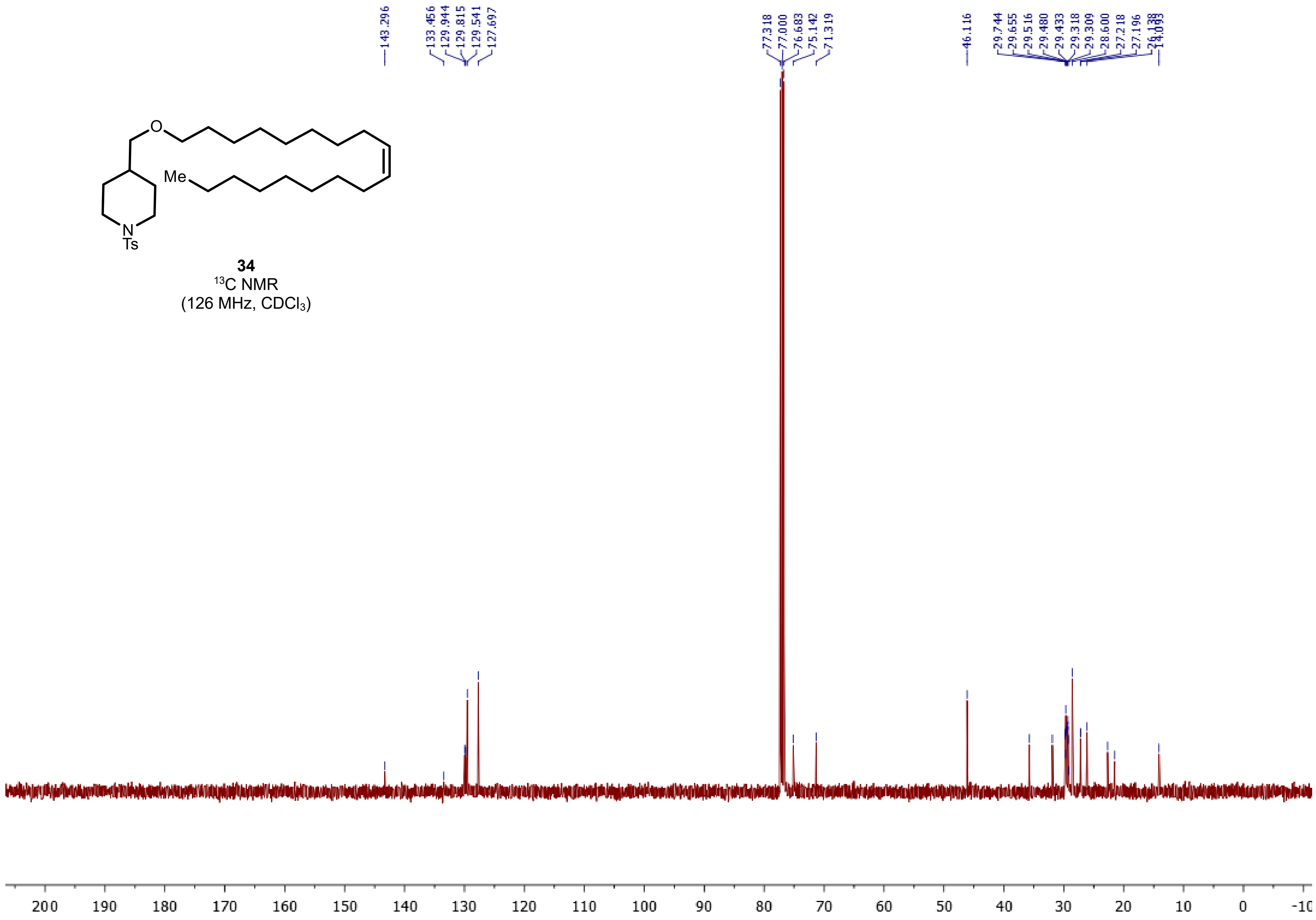

143.296
133.456
129.944
129.815
129.541
127.697
77.318
77.000
76.683
75.142
71.319
46.116
29.744
29.655
29.516
29.480
29.433
29.318
29.309
28.600
27.218
27.196
O
Me
N
Ts
34
^{13}C NMR
(126 MHz, $CDCl_3$)
200
190
180
170
160
150
140
130
120
110
100
90
80
70
60
50
40
30
20
10
0

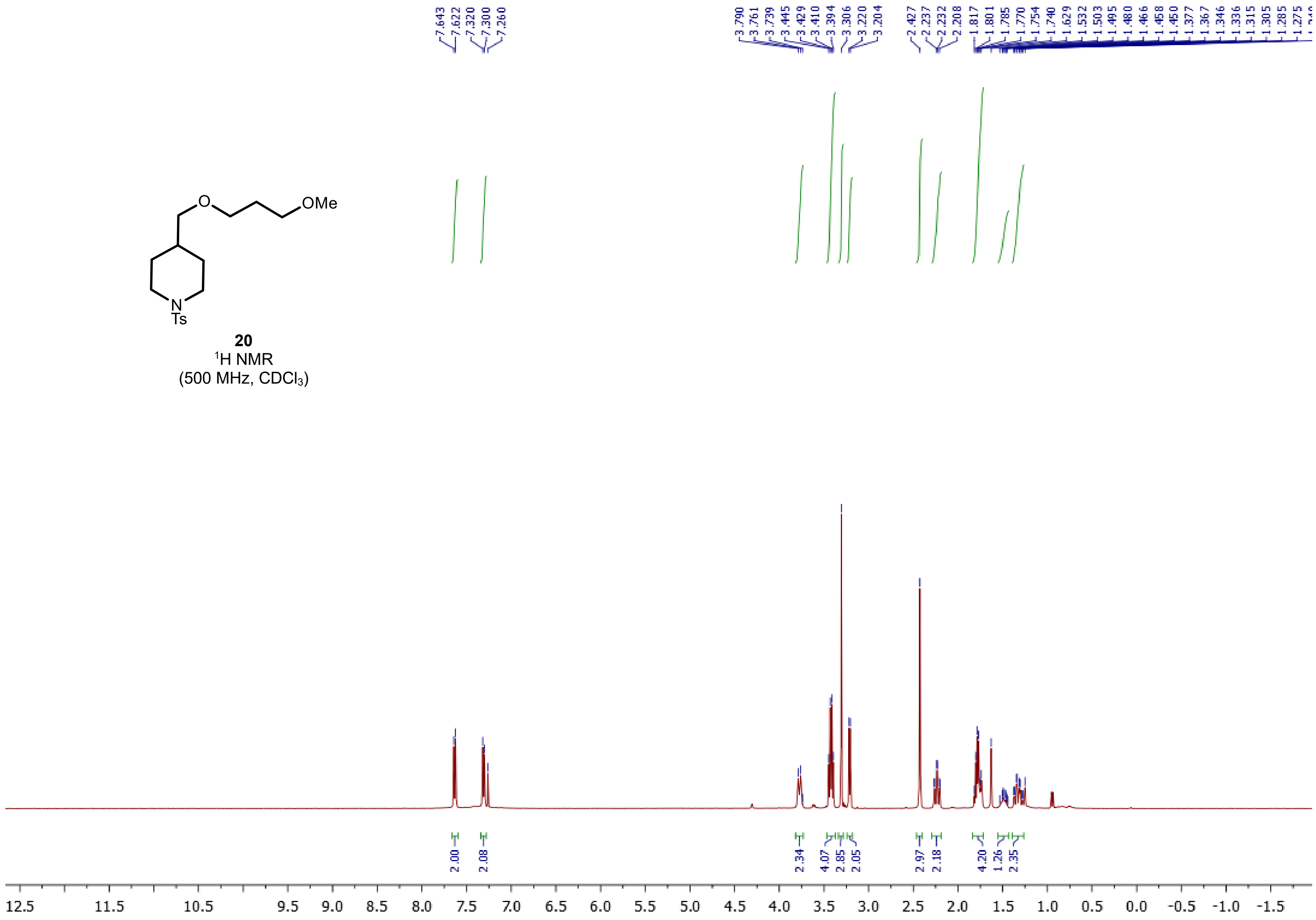

20
^{1}H NMR
(500 MHz, $CDCl_3$)

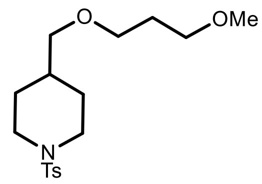

20
^{13}C NMR
(126 MHz, $CDCl_3$)

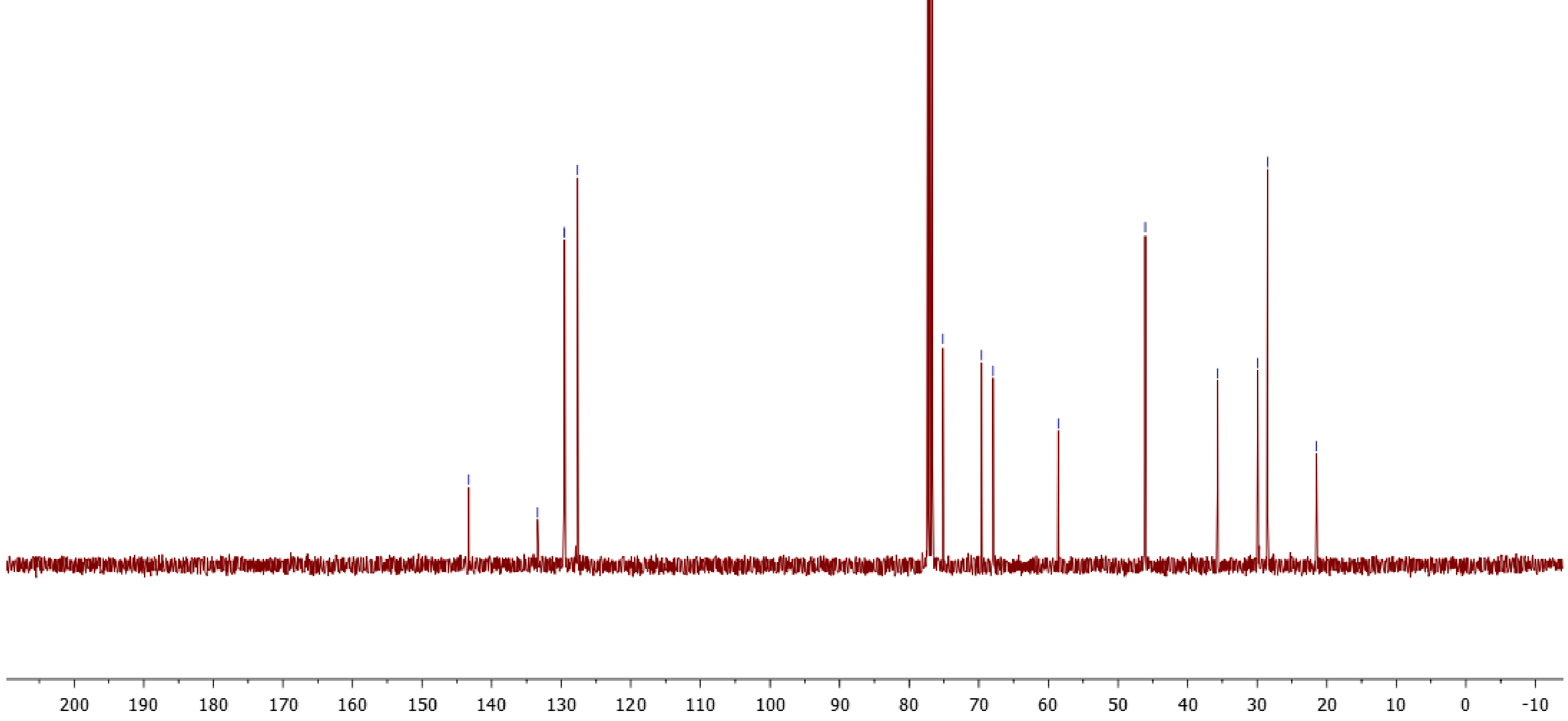

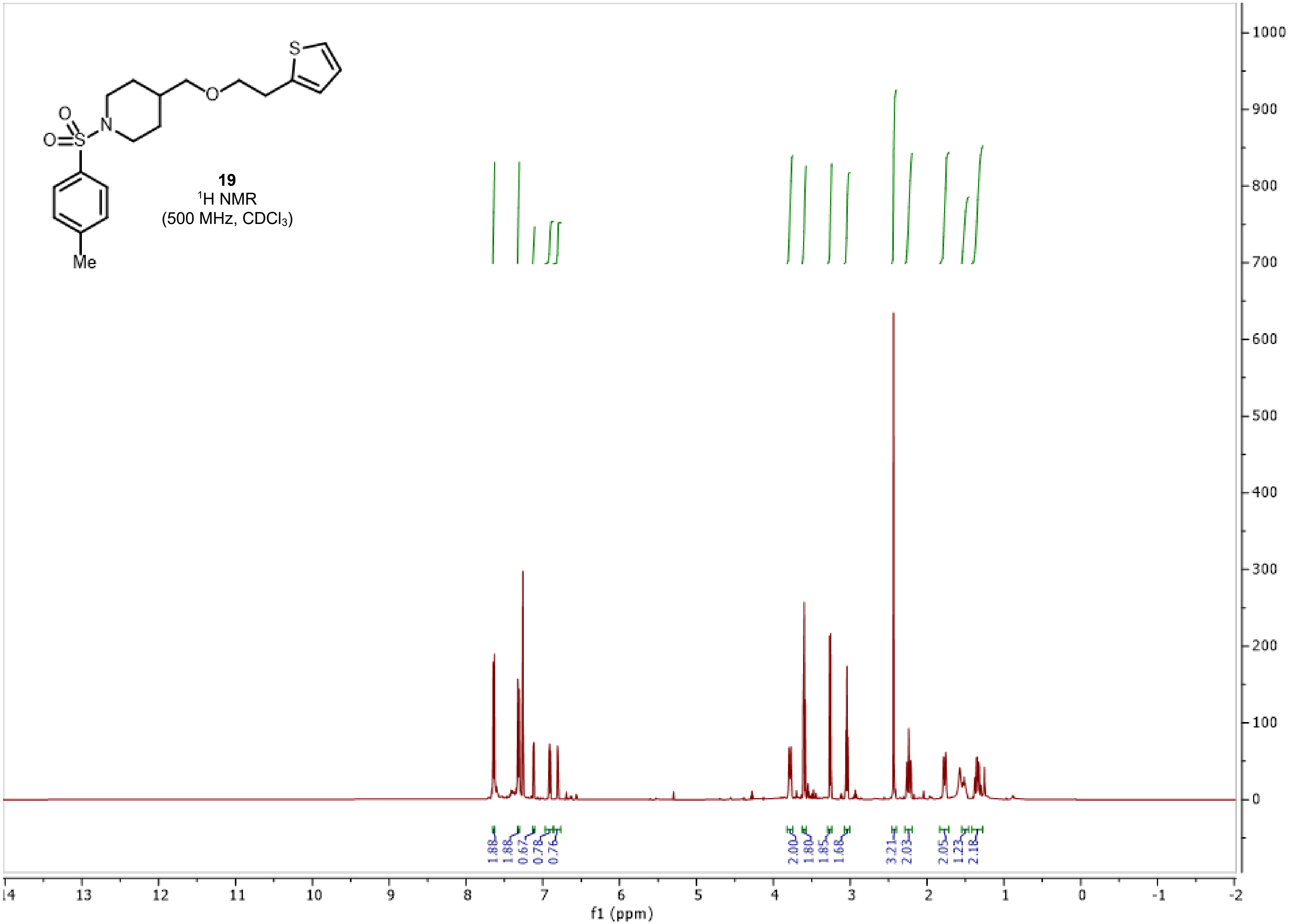

19
^{1}H NMR
(500 MHz, $CDCl_3$)
f1 (ppm)

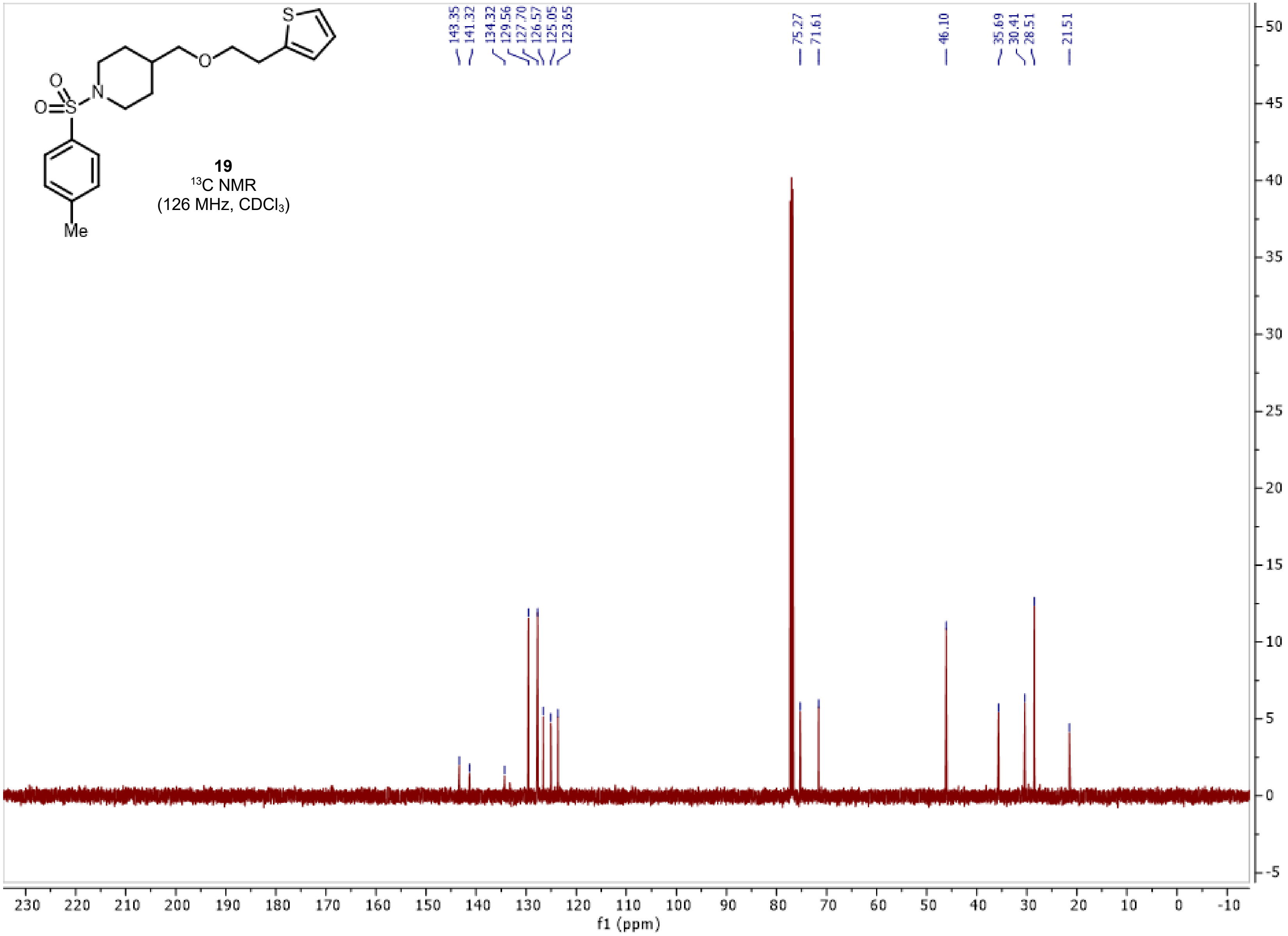
19
^{13}C NMR
(126 MHz, $CDCl_3$)
143.35
141.32
134.32
129.56
127.70
126.57
125.05
123.65
75.27
71.61
46.10
35.69
30.41
28.51
21.51
f1 (ppm)

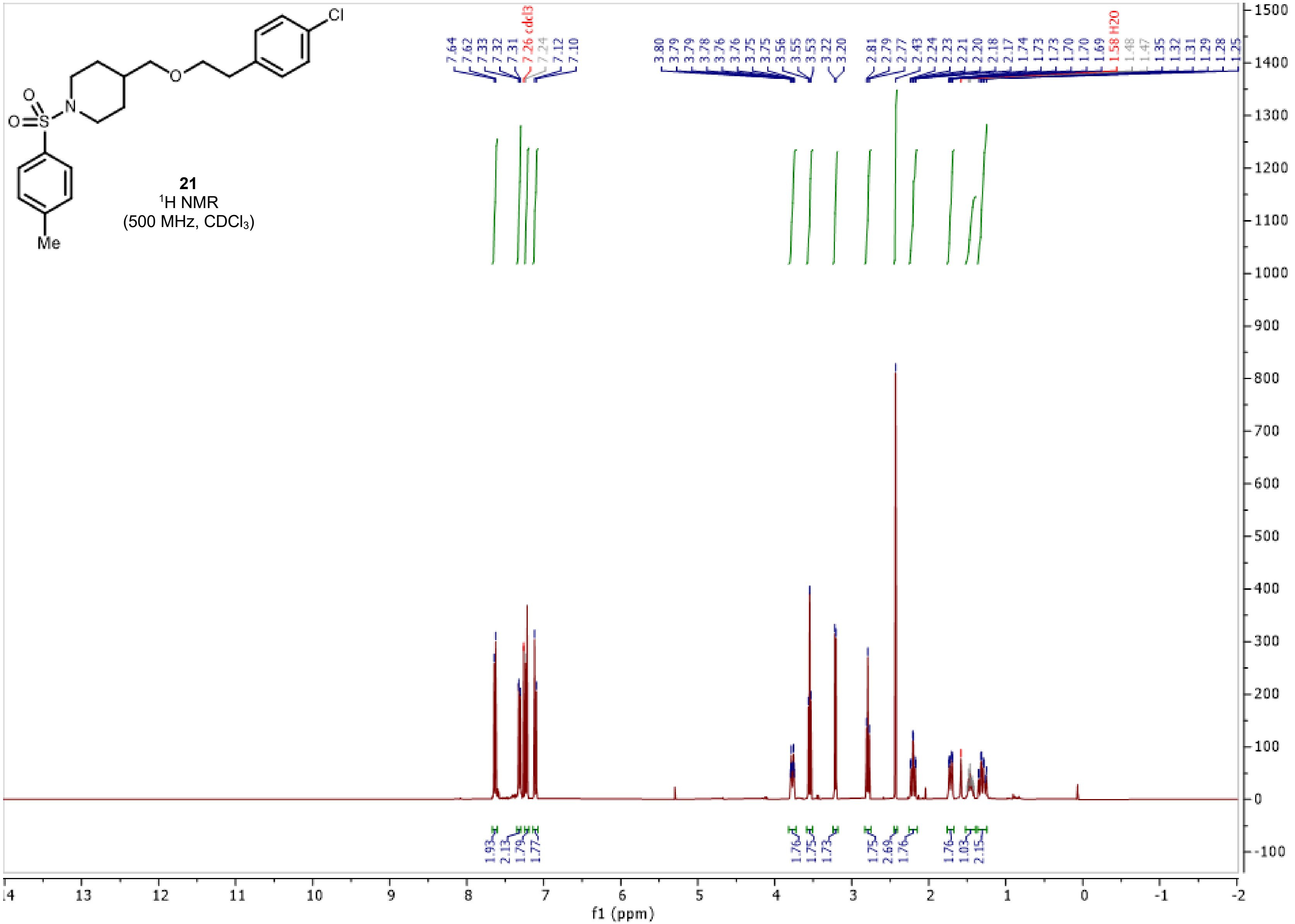
Cl
O
O
O
S
N
Me
21
¹H NMR
(500 MHz, CDCl3)
7.64 7.62 7.33 7.32 7.31 7.26 cdcl3 7.24 7.12 7.10
3.80 3.79 3.79 3.78 3.76 3.76 3.75 3.75 3.56 3.55 3.53 3.22 3.20
2.81 2.79 2.77 2.43 2.24 2.23 2.21 2.20 2.18 2.17 1.74 1.73 1.73 1.70 1.70 1.69 1.58 H2O 1.48 1.47 1.35 1.32 1.31 1.29 1.28 1.25
1.93 2.13 1.79 1.77 1.76 1.75 1.73 1.75 2.69 1.76 1.76 1.03 2.15
f1 (ppm)

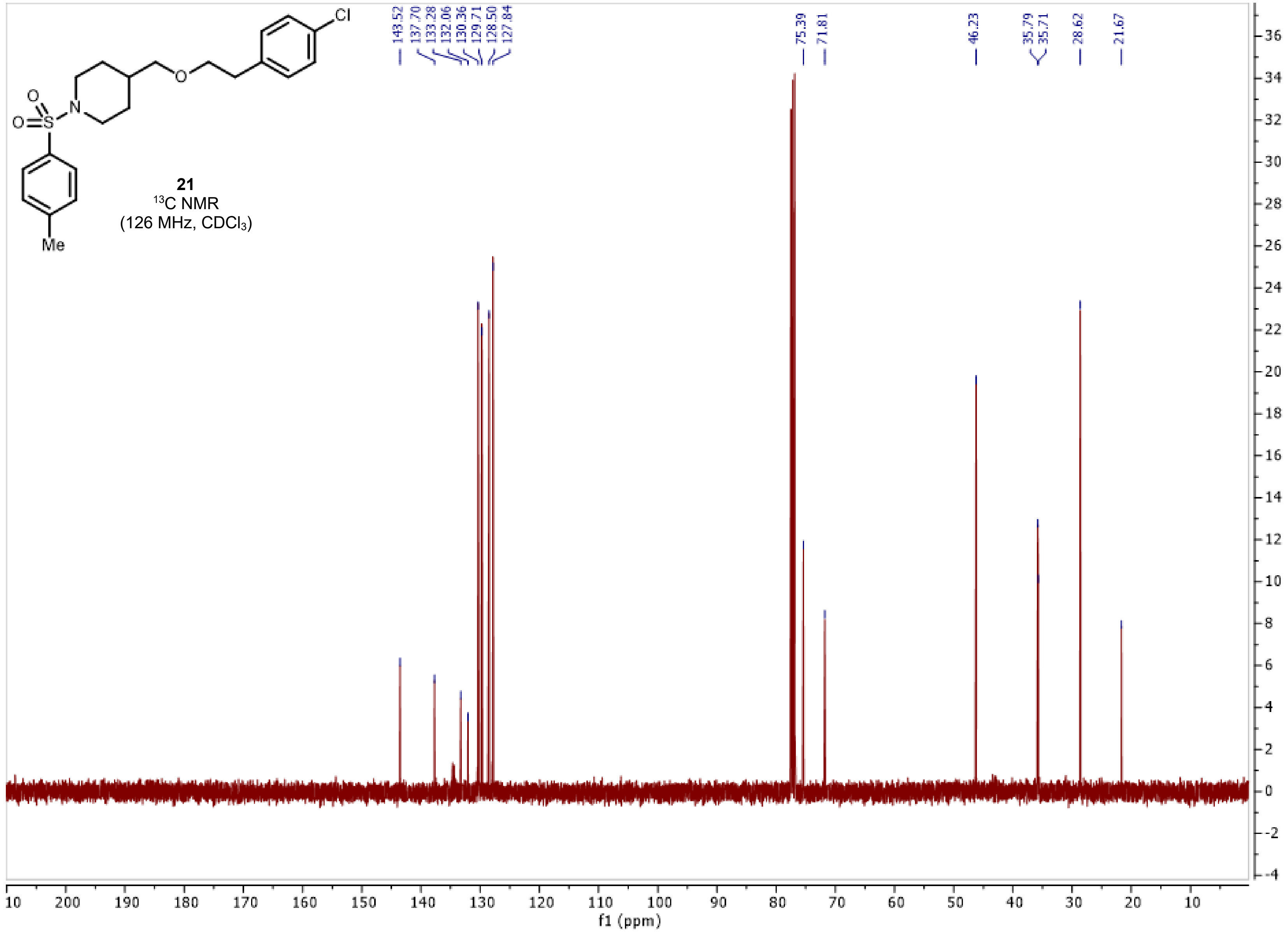
21
13C NMR
(126 MHz, CDCl3)
143.52
137.70
133.28
132.06
130.36
129.71
128.50
127.84
75.39
71.81
46.23
35.79
35.71
28.62
21.67
f1 (ppm)

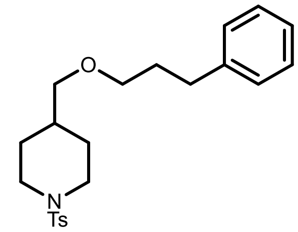

22
^{1}H NMR
(500 MHz, $CDCl_3$)

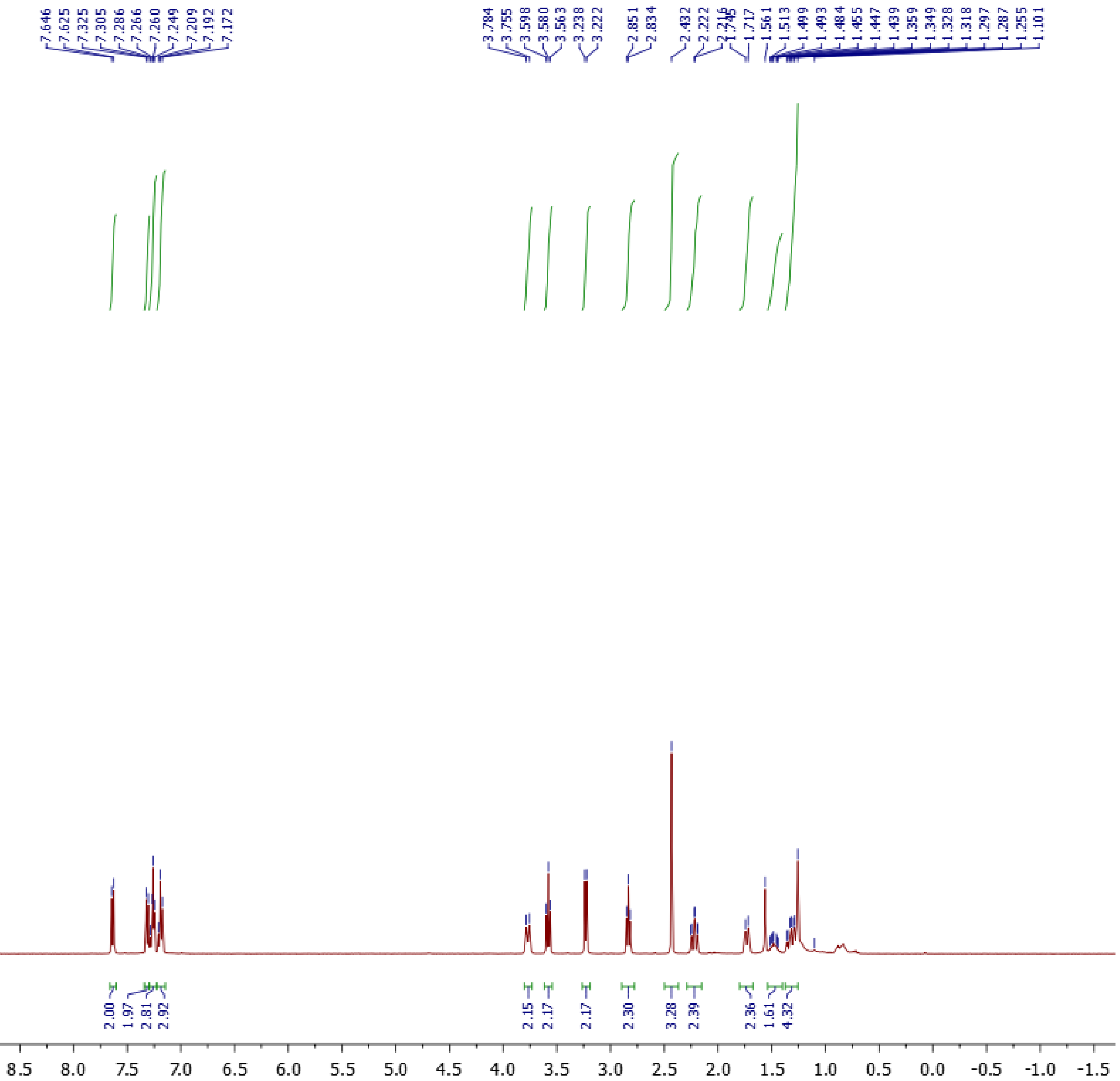

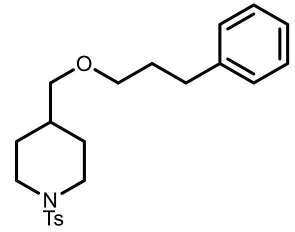

22
^{13}C NMR
(126 MHz, $CDCl_3$)

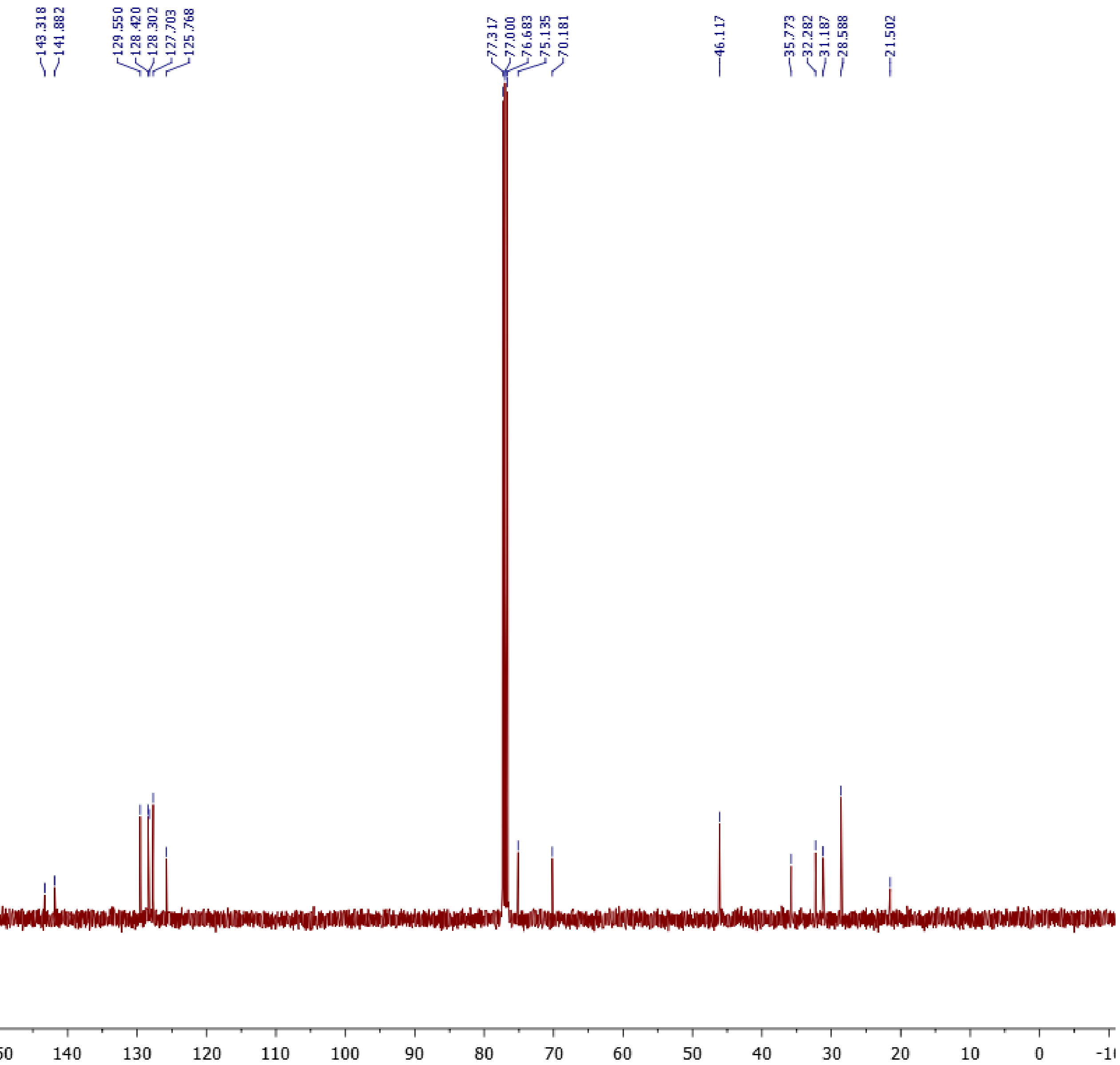

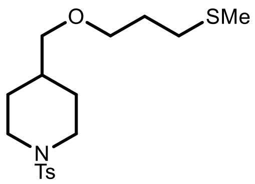

20
^{1}H NMR
(500 MHz, $CDCl_3$)

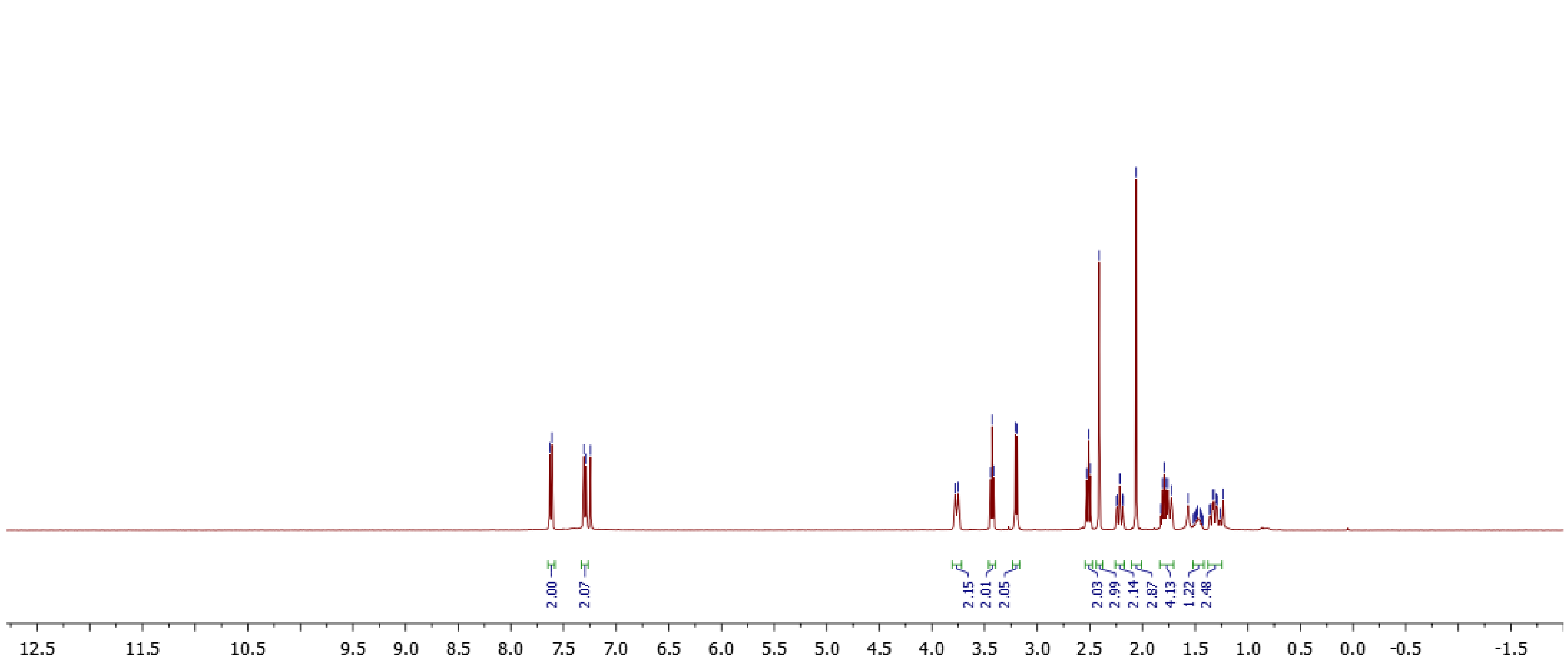

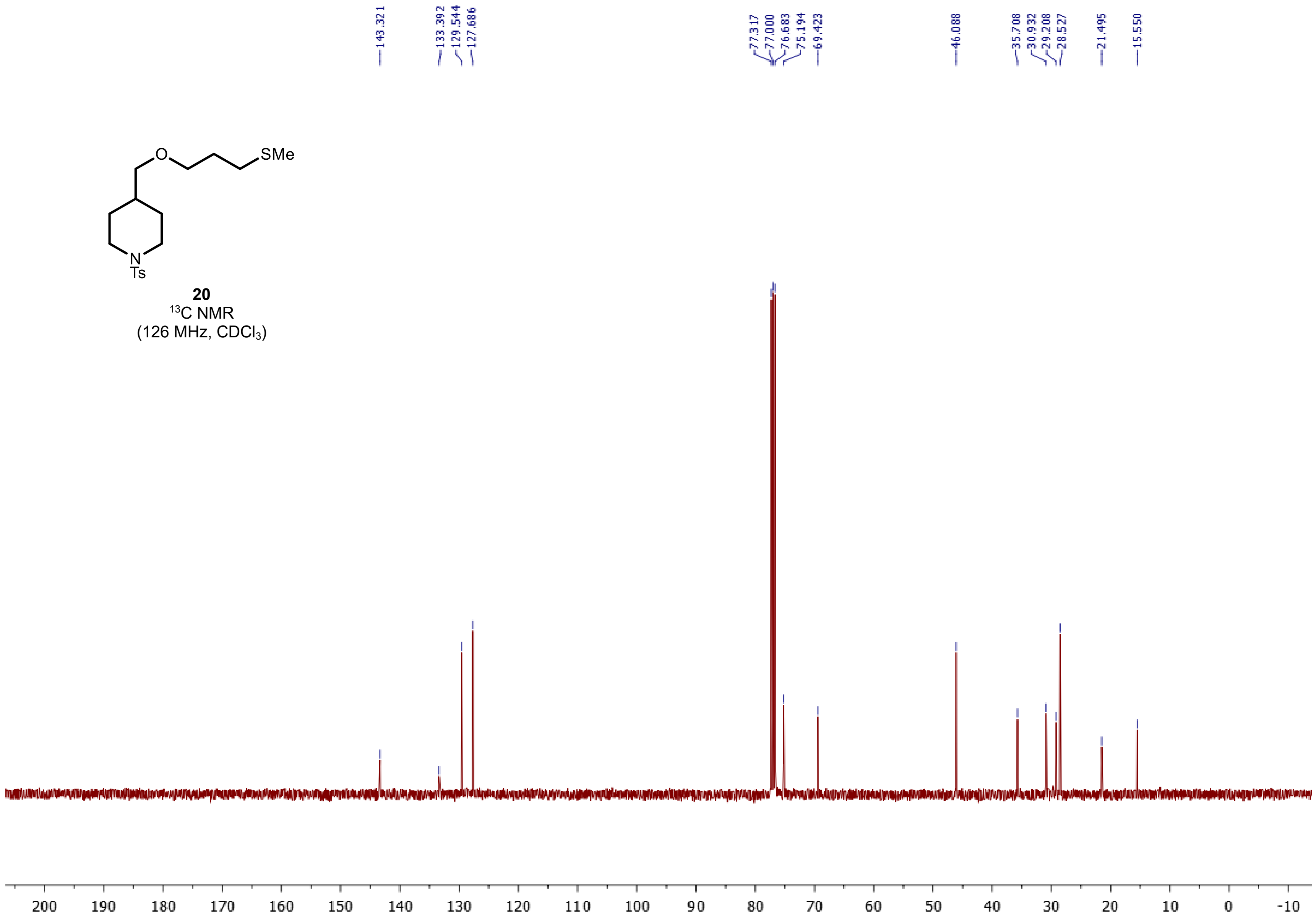
SMe
O
N
Ts
20
13C NMR
(126 MHz, CDCl3)
143.321
133.392
129.544
127.686
77.317
77.000
76.683
75.194
69.423
46.088
35.708
30.932
29.208
28.527
21.495
15.550
200 190 180 170 160 150 140 130 120 110 100 90 80 70 60 50 40 30 20 10 0 -10

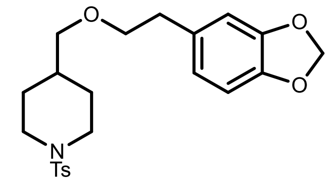

23
^{1}H NMR
(500 MHz, $CDCl_3$)

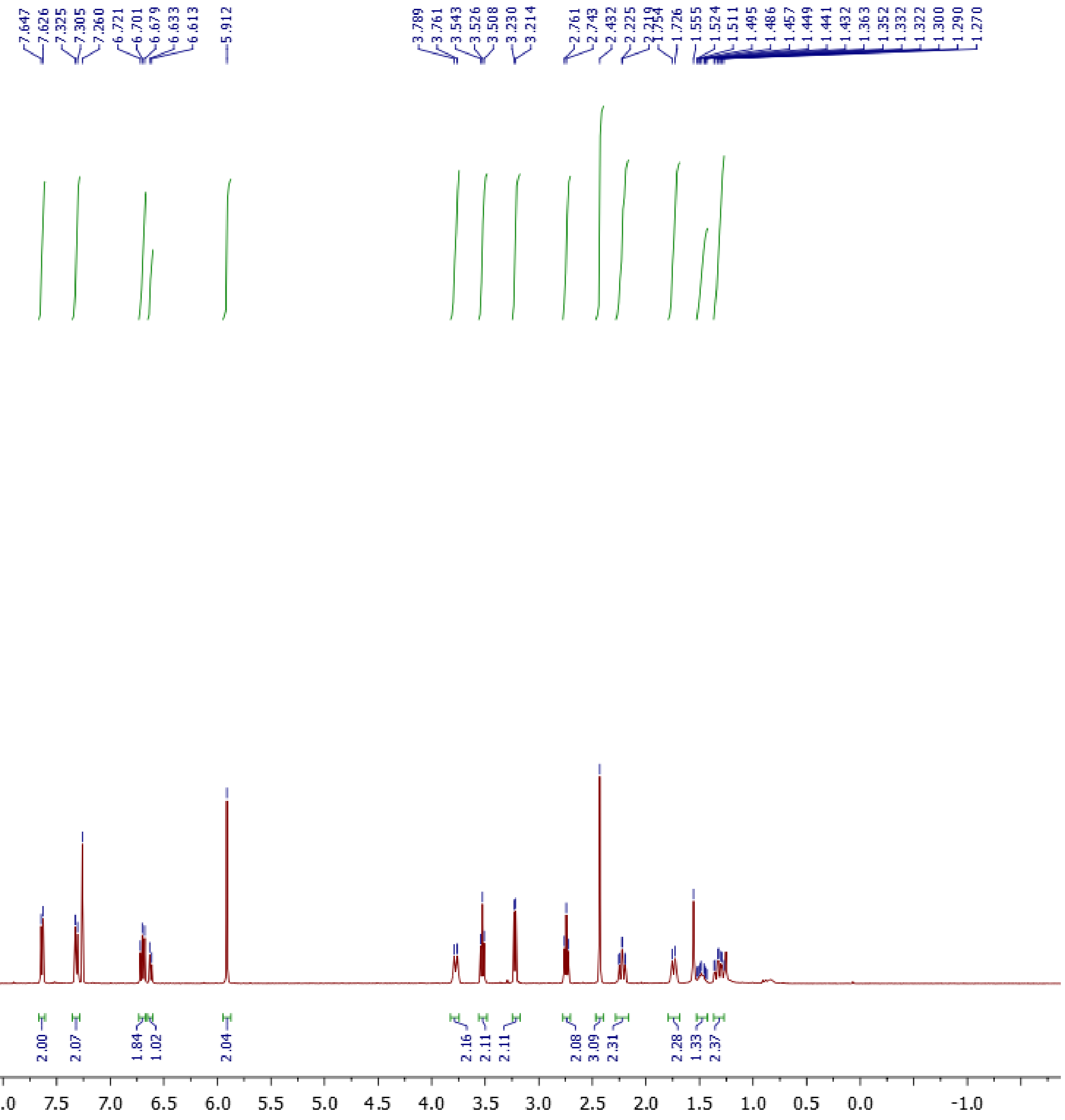

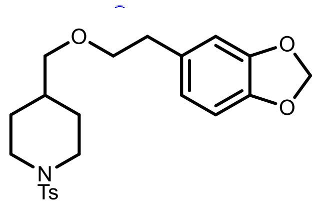

23
^{13}C NMR
(126 MHz, $CDCl_3$)

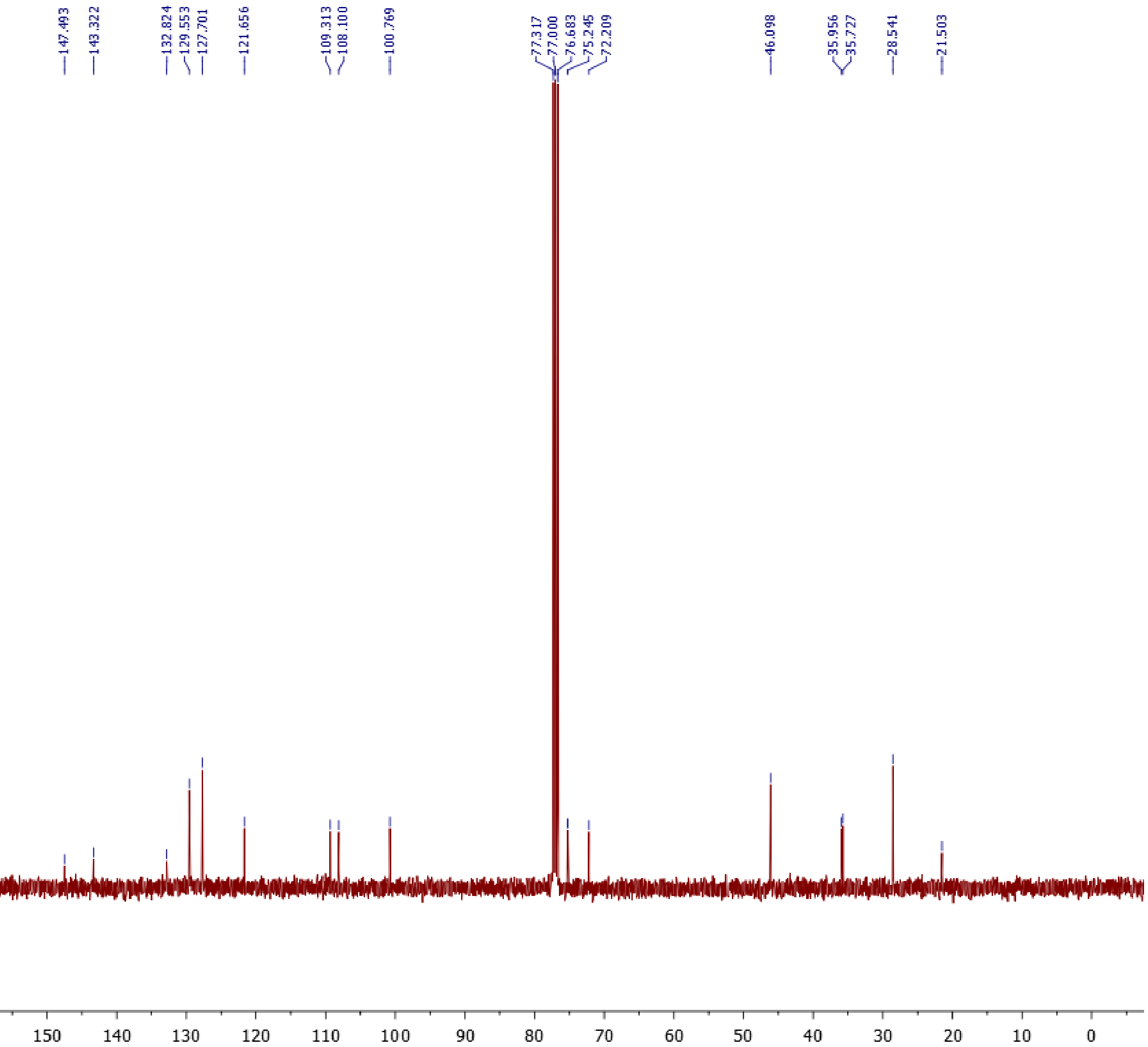

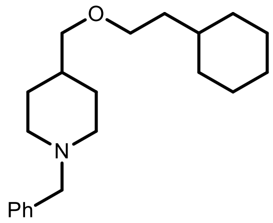

27
^{1}H NMR
(500 MHz, $CDCl_3$)

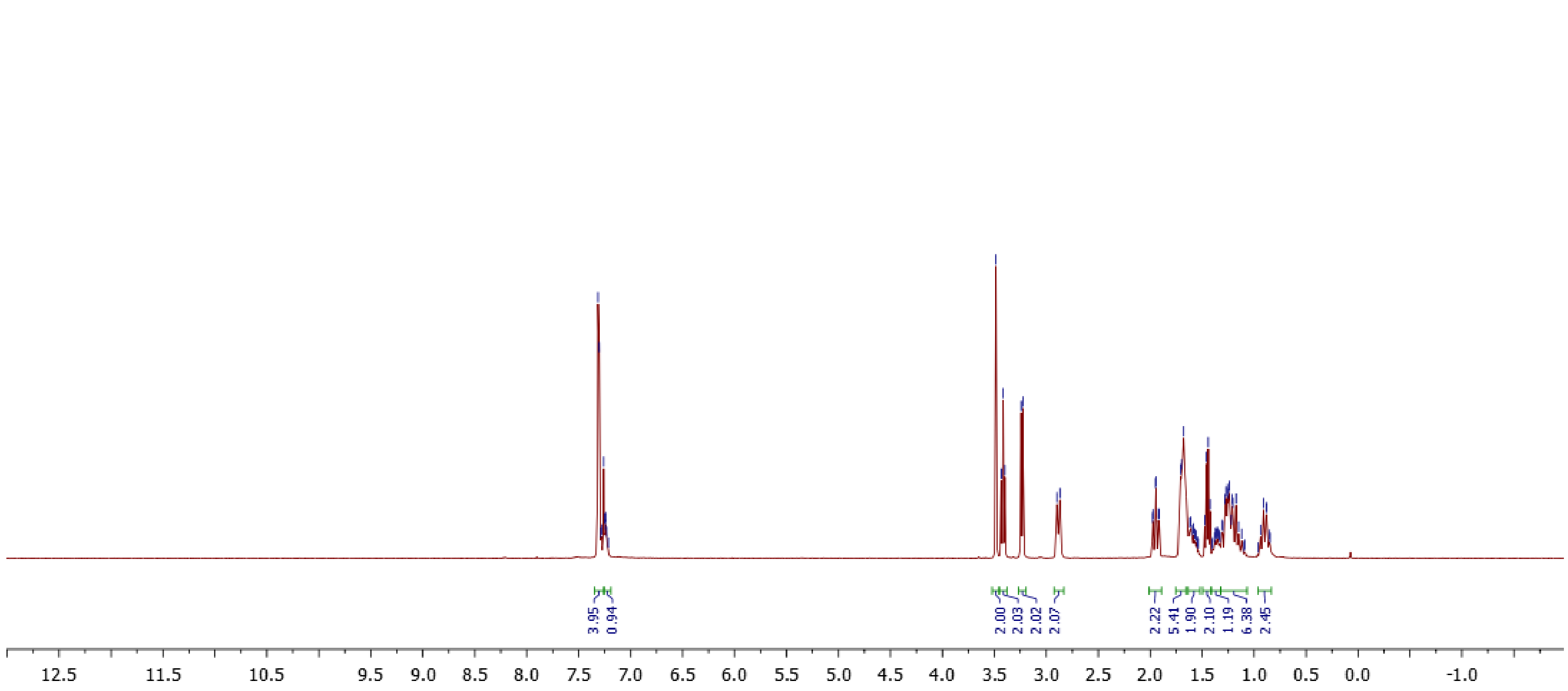

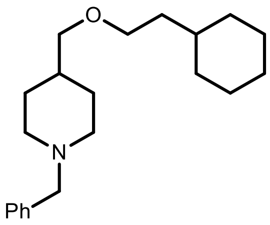

27
^{13}C NMR
(126 MHz, $CDCl_3$)

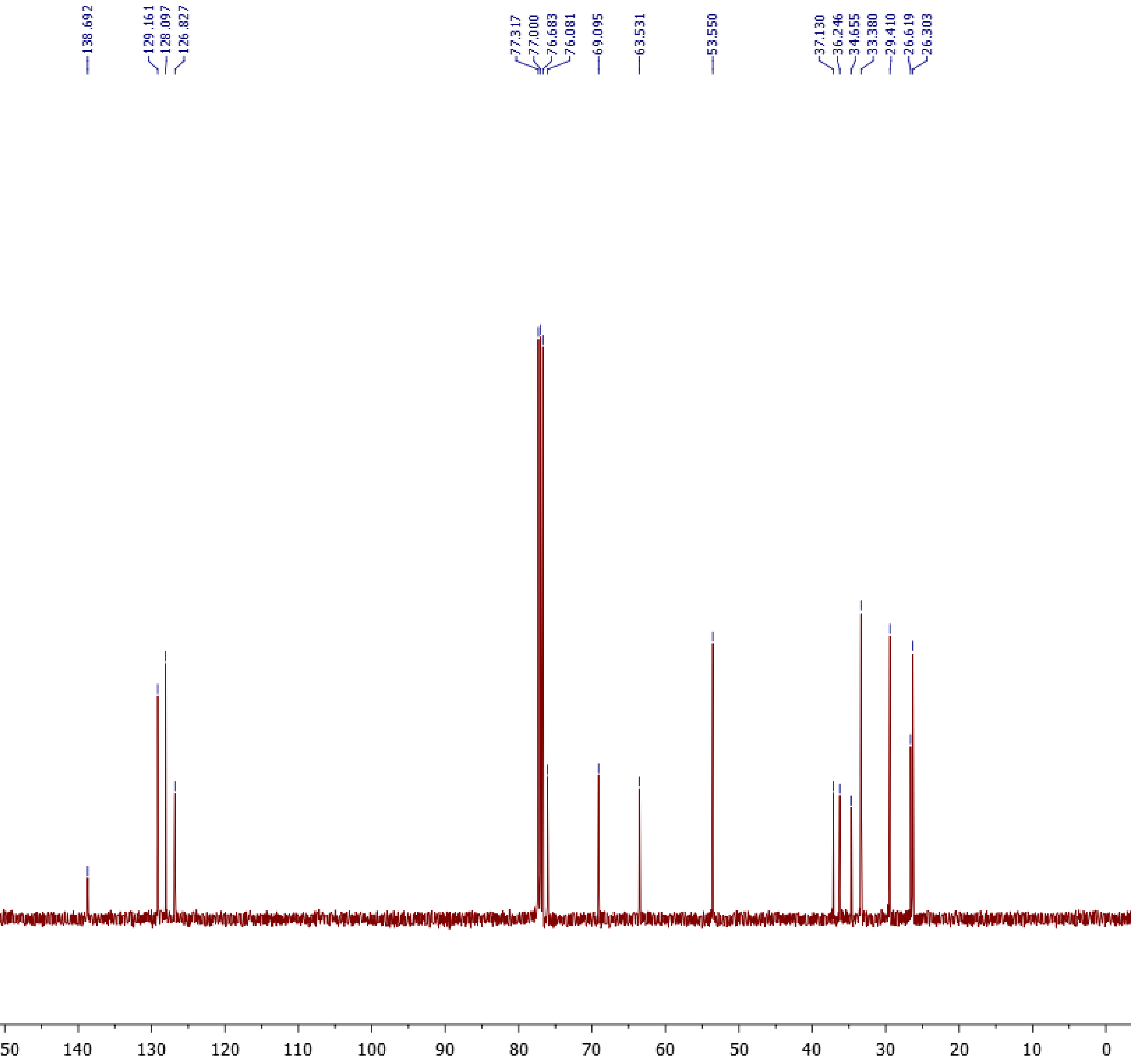

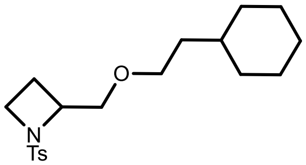

26
^{1}H NMR
(500 MHz, $CDCl_3$)

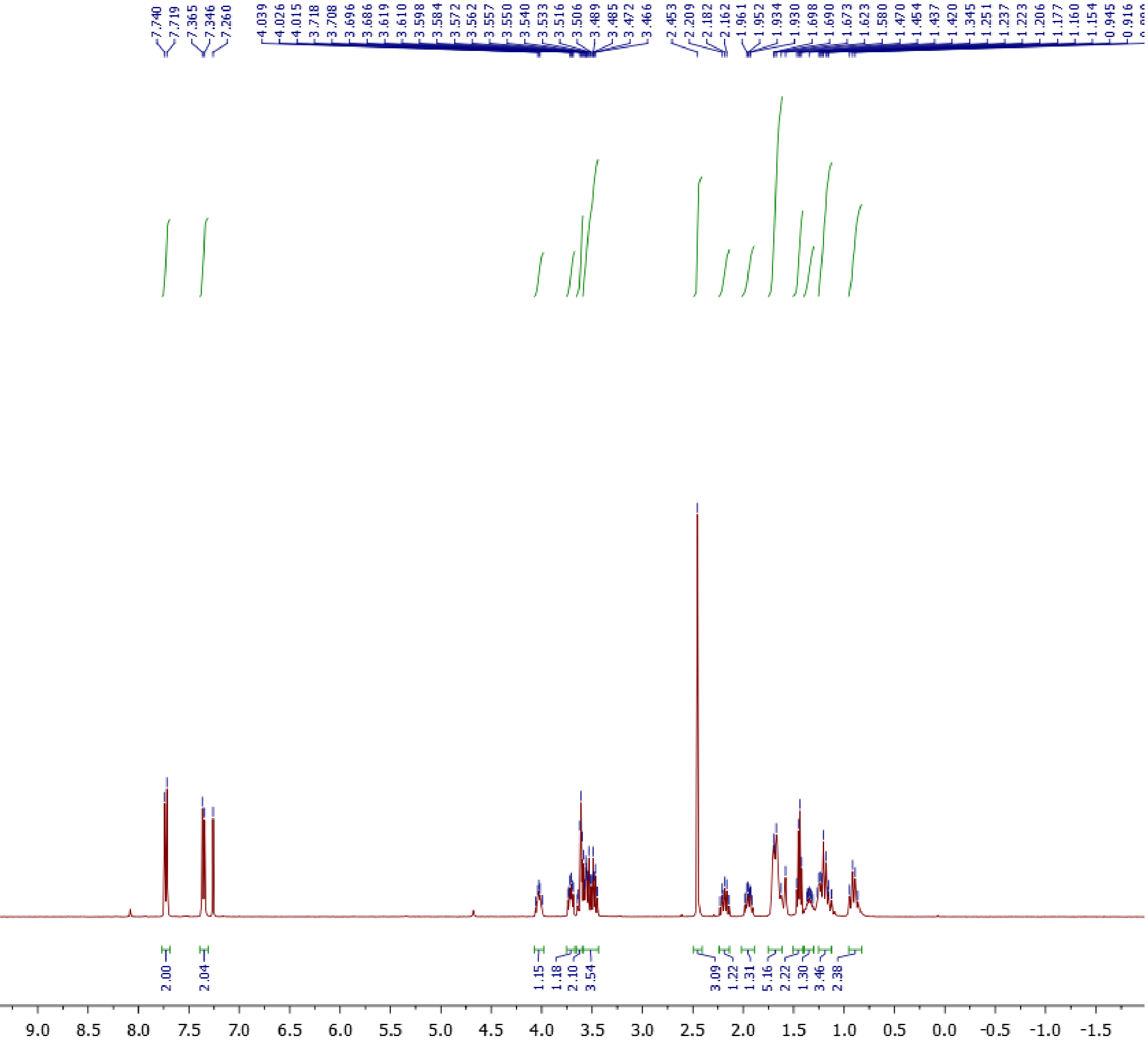

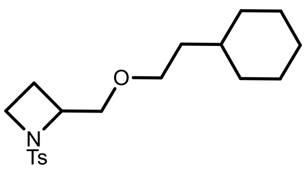

26
^{13}C NMR
(126 MHz, $CDCl_3$)

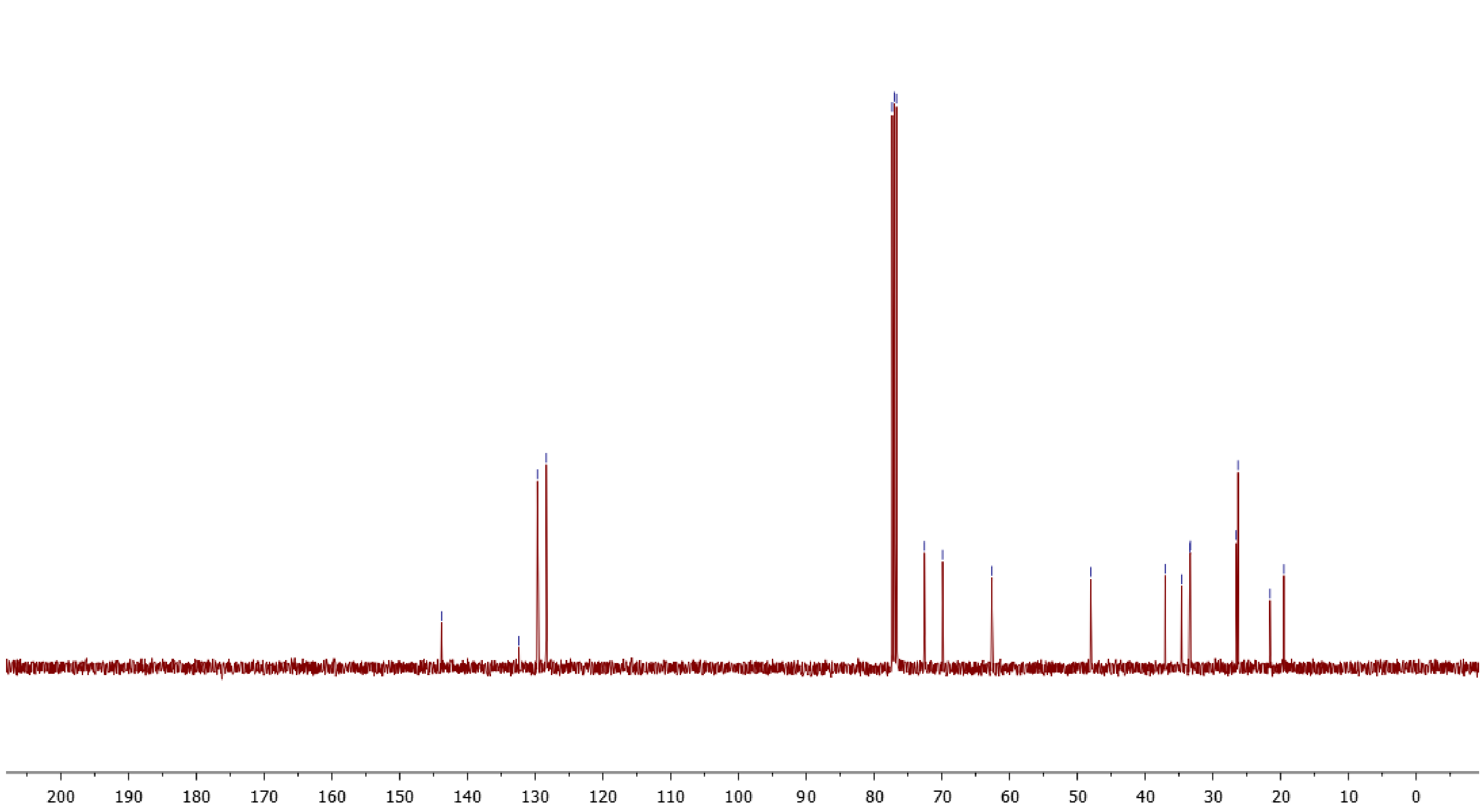

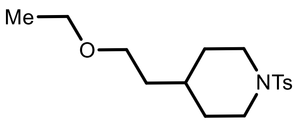

30
^{1}H NMR
(500 MHz, $CDCl_3$)

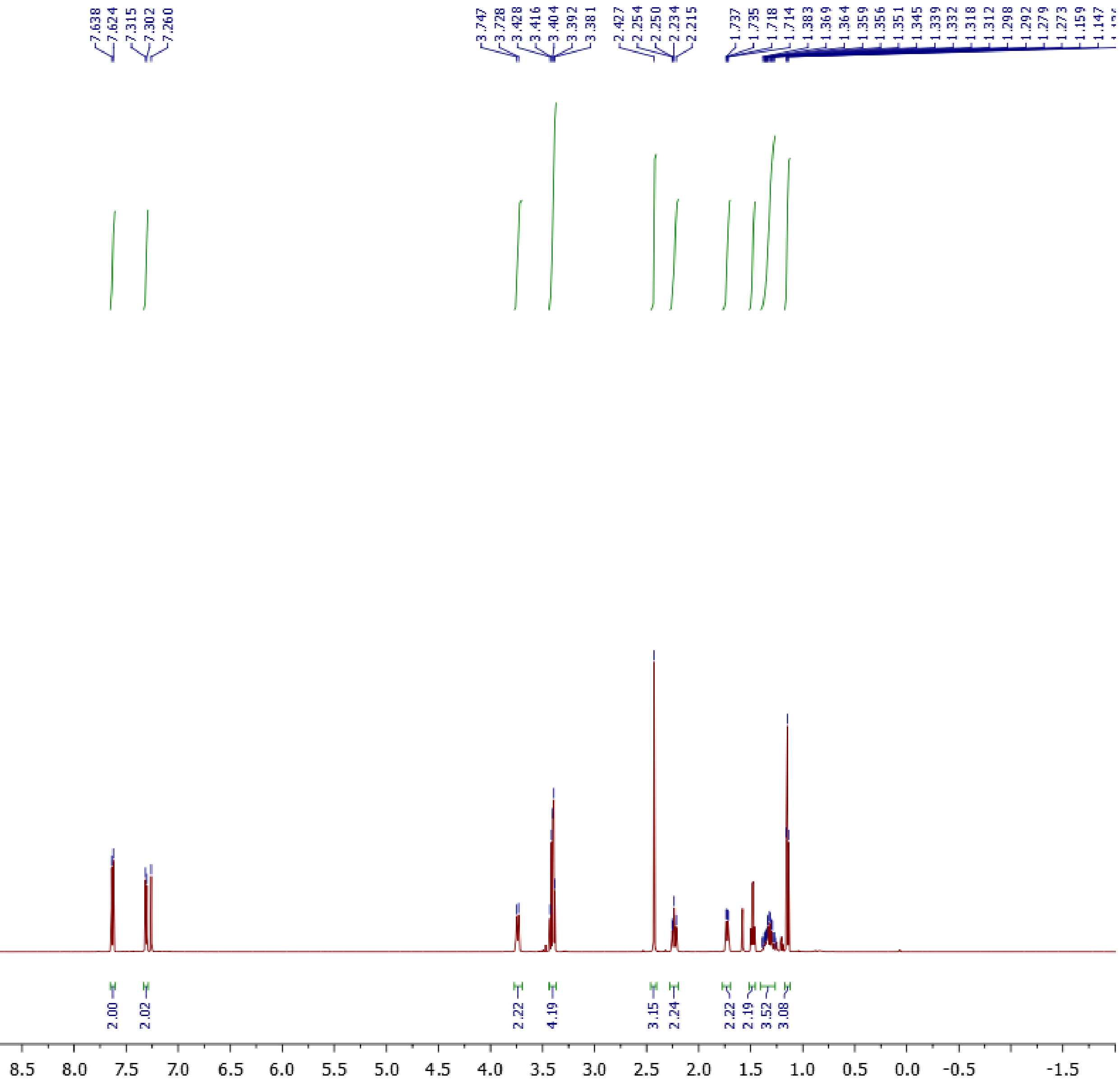

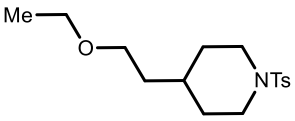

30
^{13}C NMR
(126 MHz, $CDCl_3$)

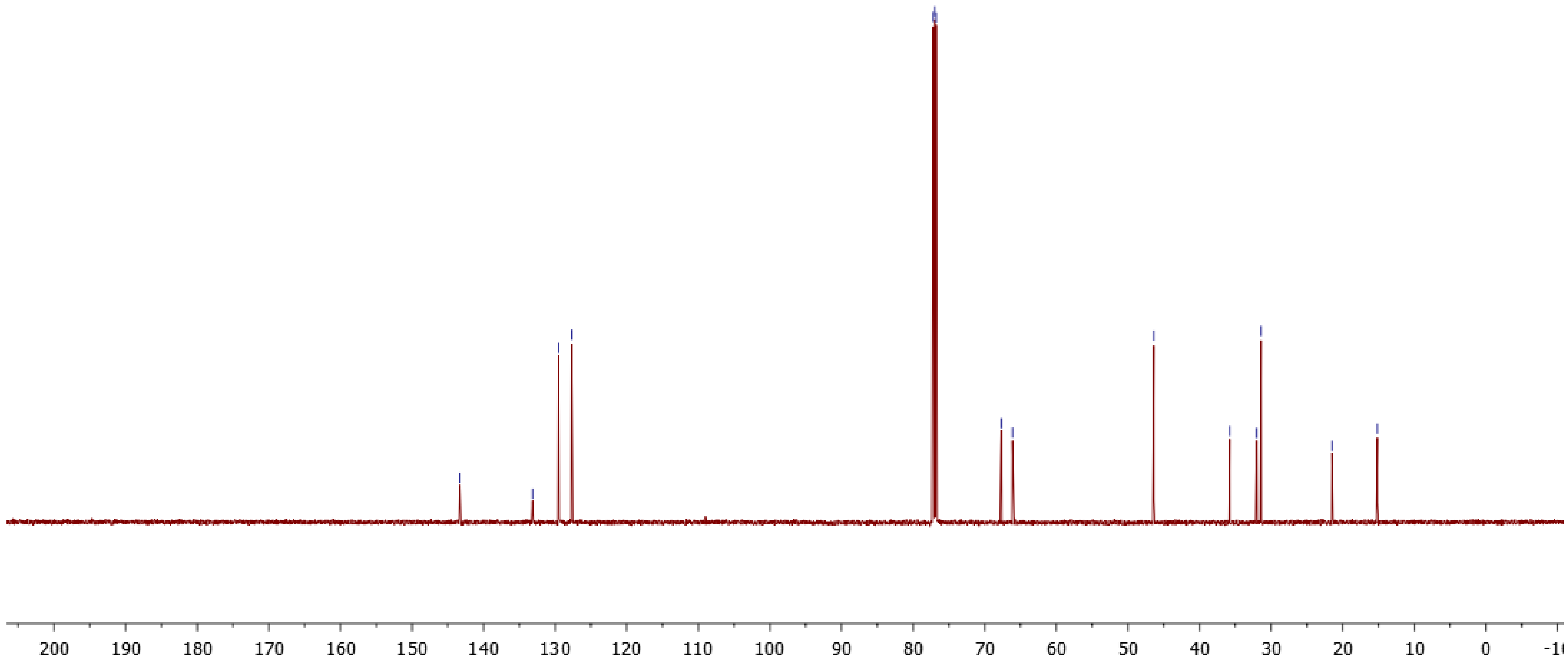

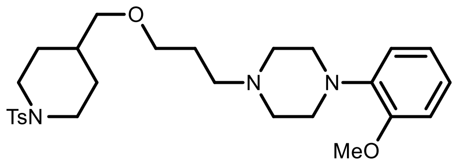

35
^1H NMR
(500 MHz, $CDCl_3$)

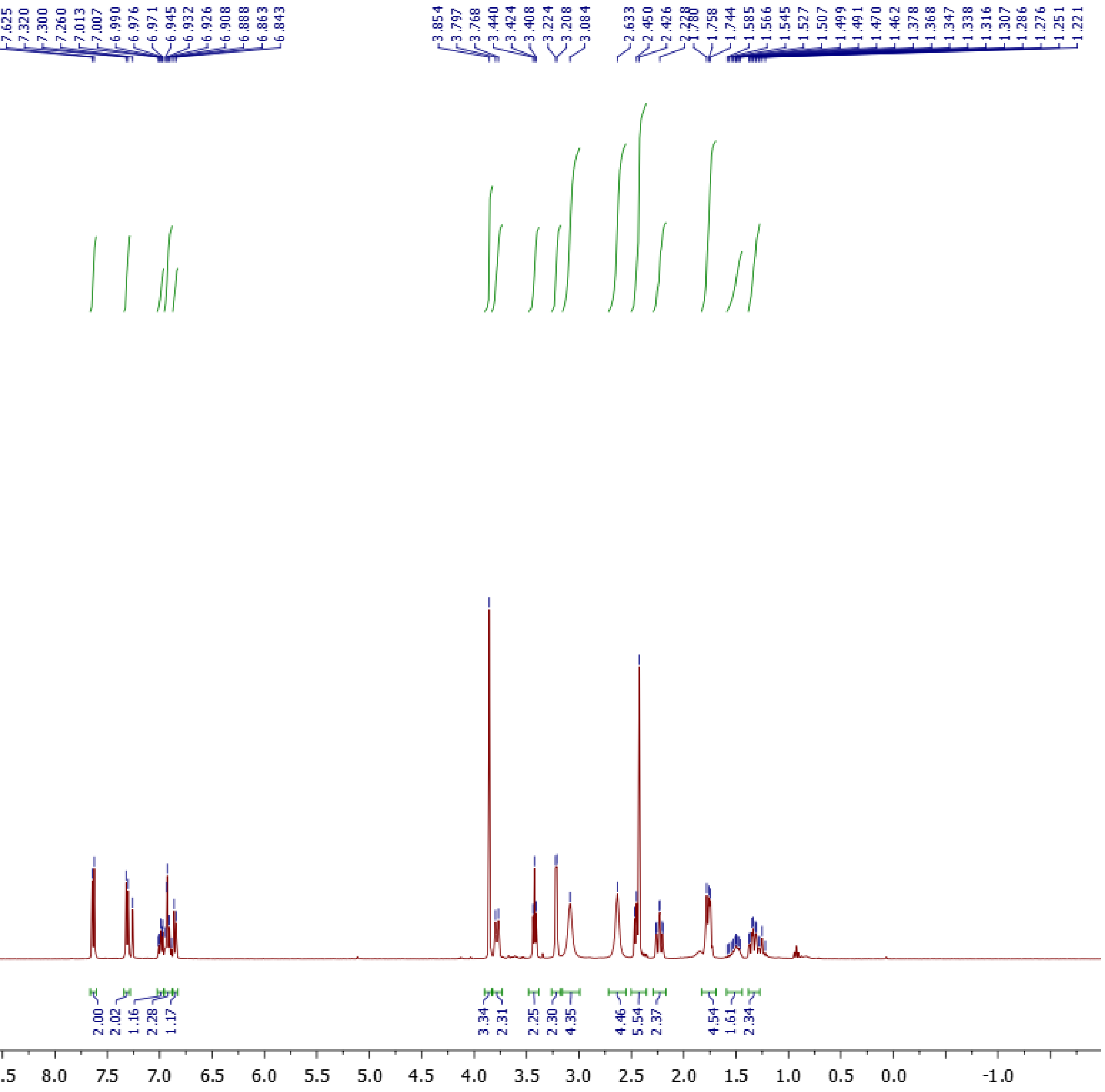

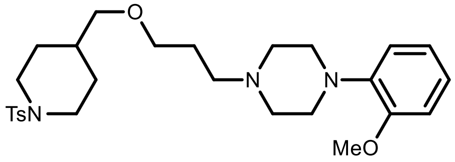

35

^{13}C NMR

(126 MHz, $CDCl_3$)

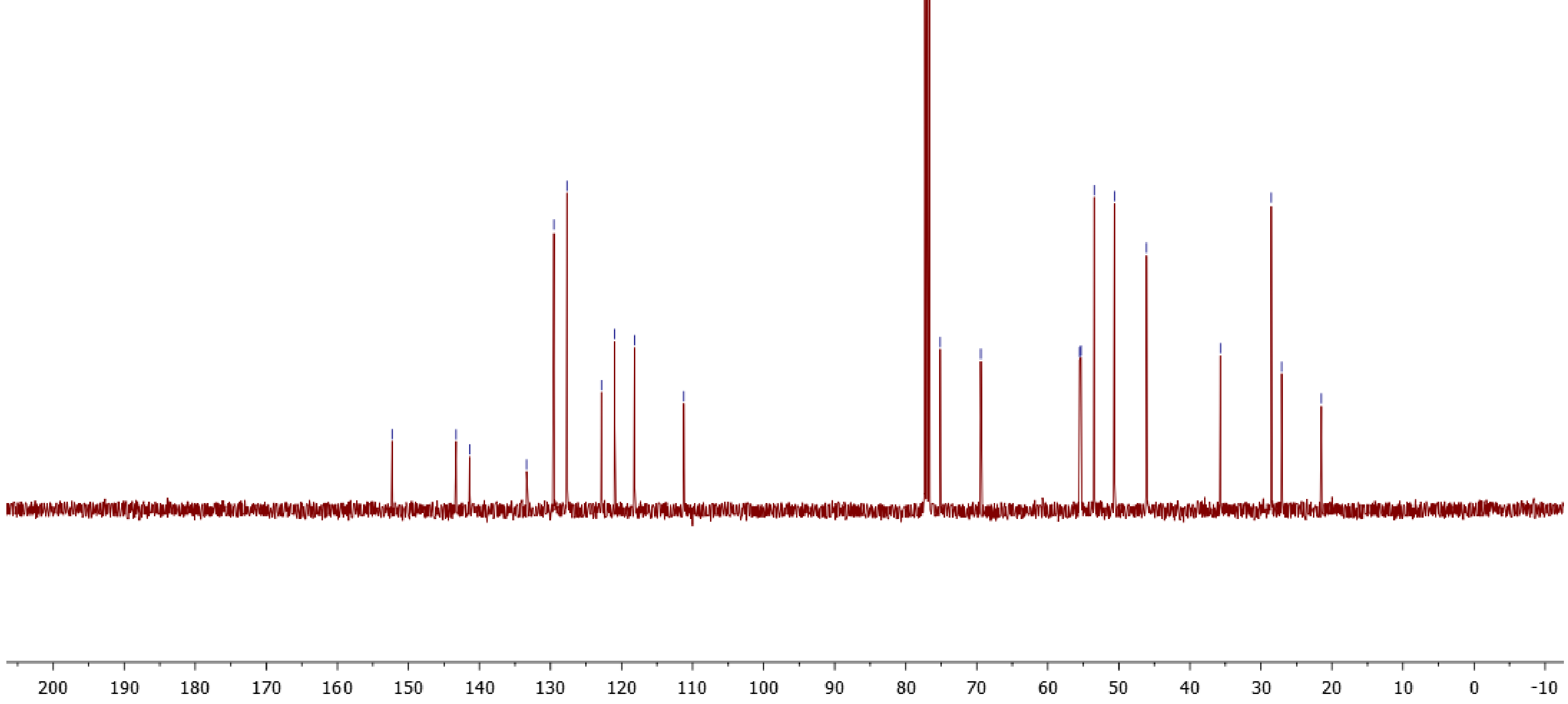

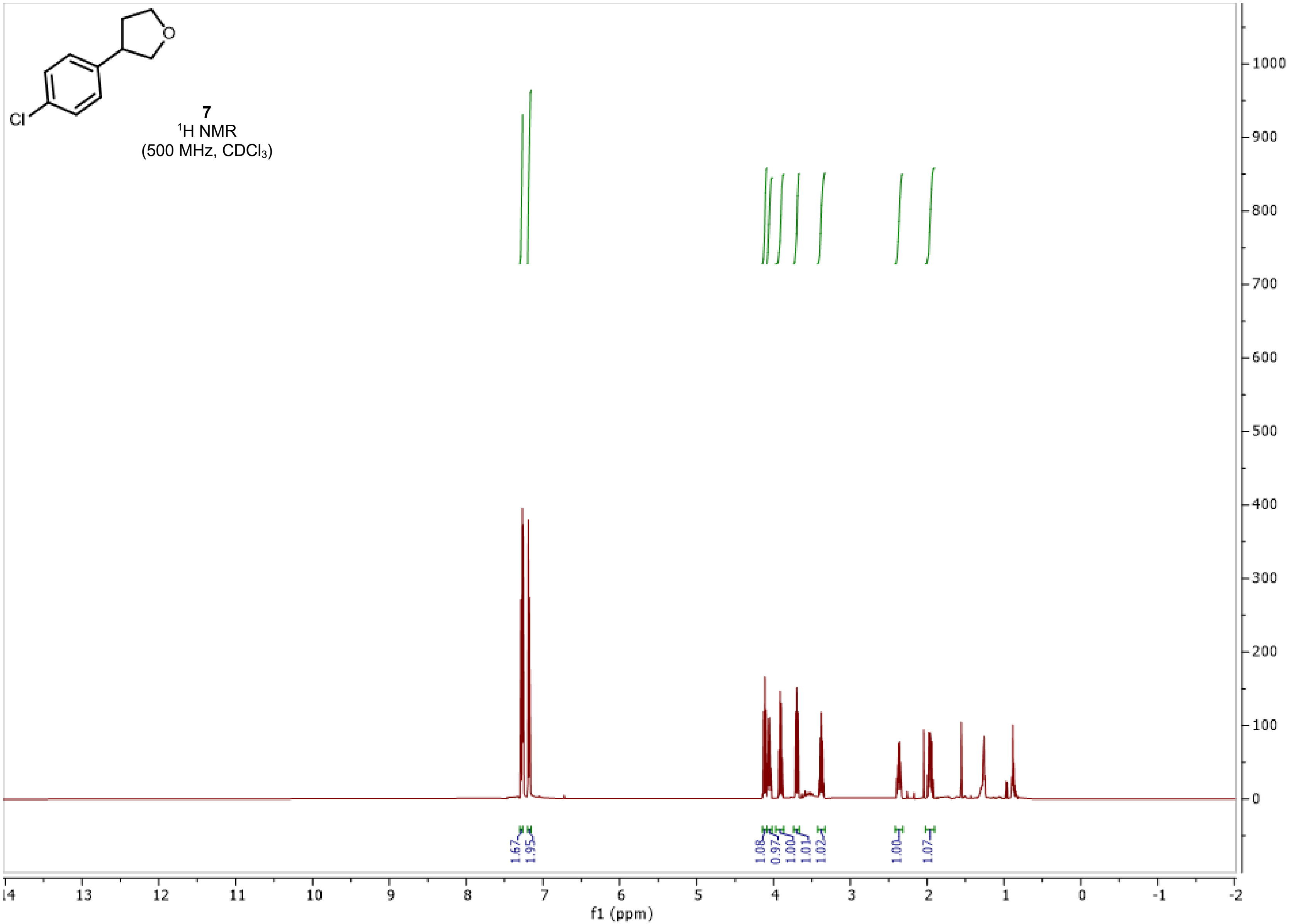

Cl
O
7
1H NMR
(500 MHz, CDCl3)
f1 (ppm)

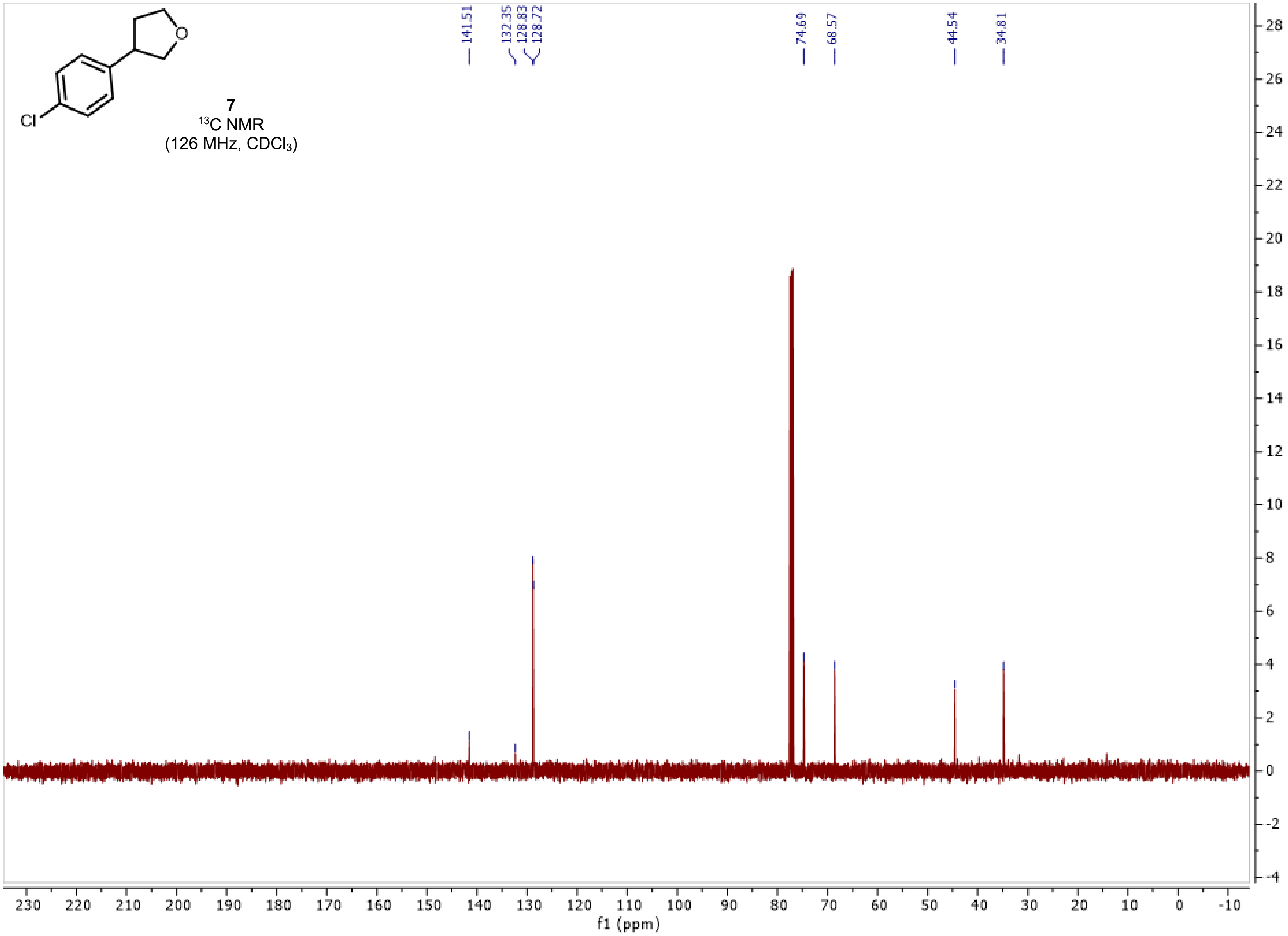

7
13C NMR
(126 MHz, CDCl3)
141.51
132.35
128.83
128.72
74.69
68.57
44.54
34.81
f1 (ppm)

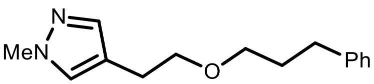

29
^{1}H NMR
(500 MHz, $CDCl_3$)

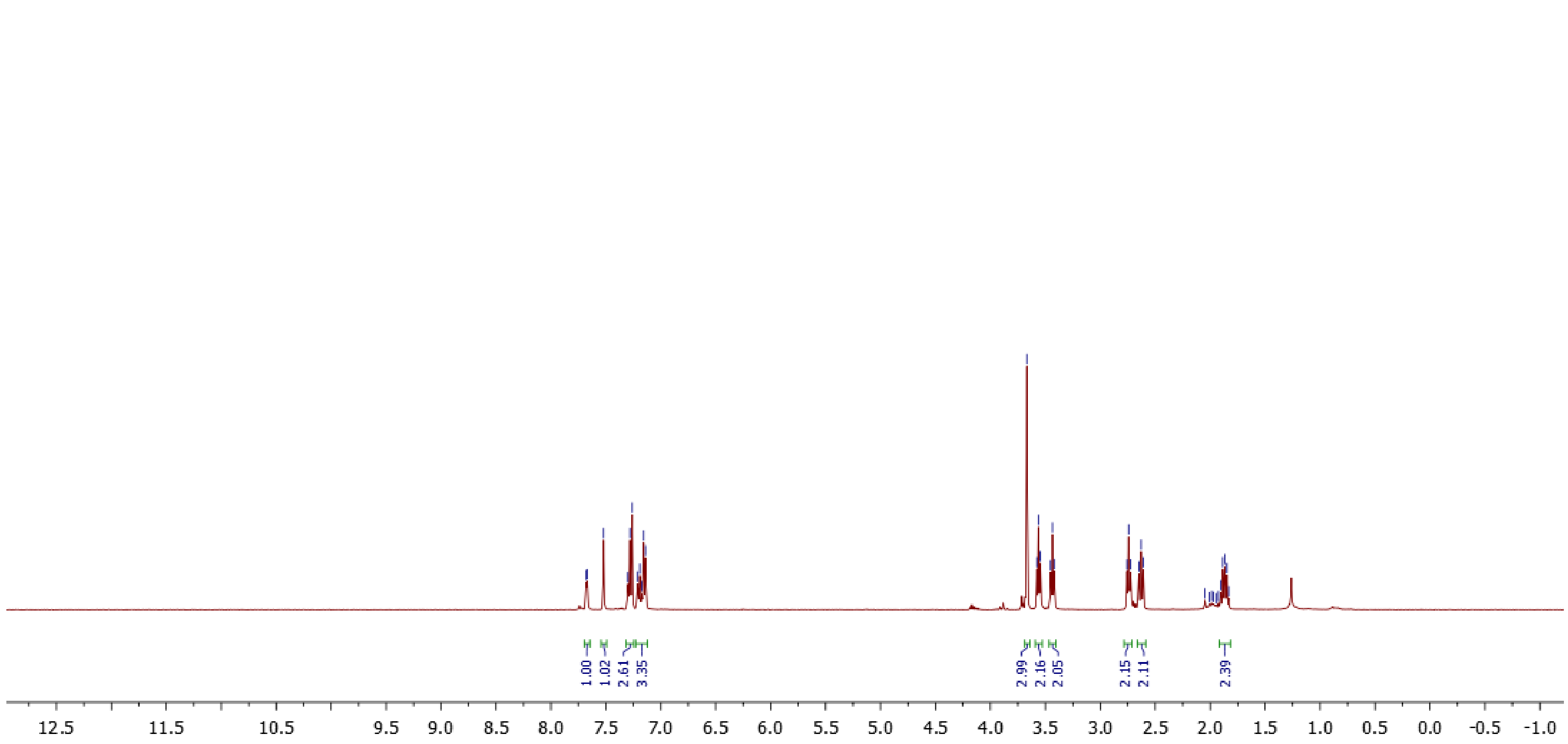

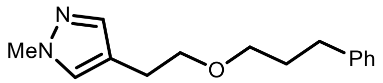

29
^{13}C NMR
(126 MHz, $CDCl_3$)

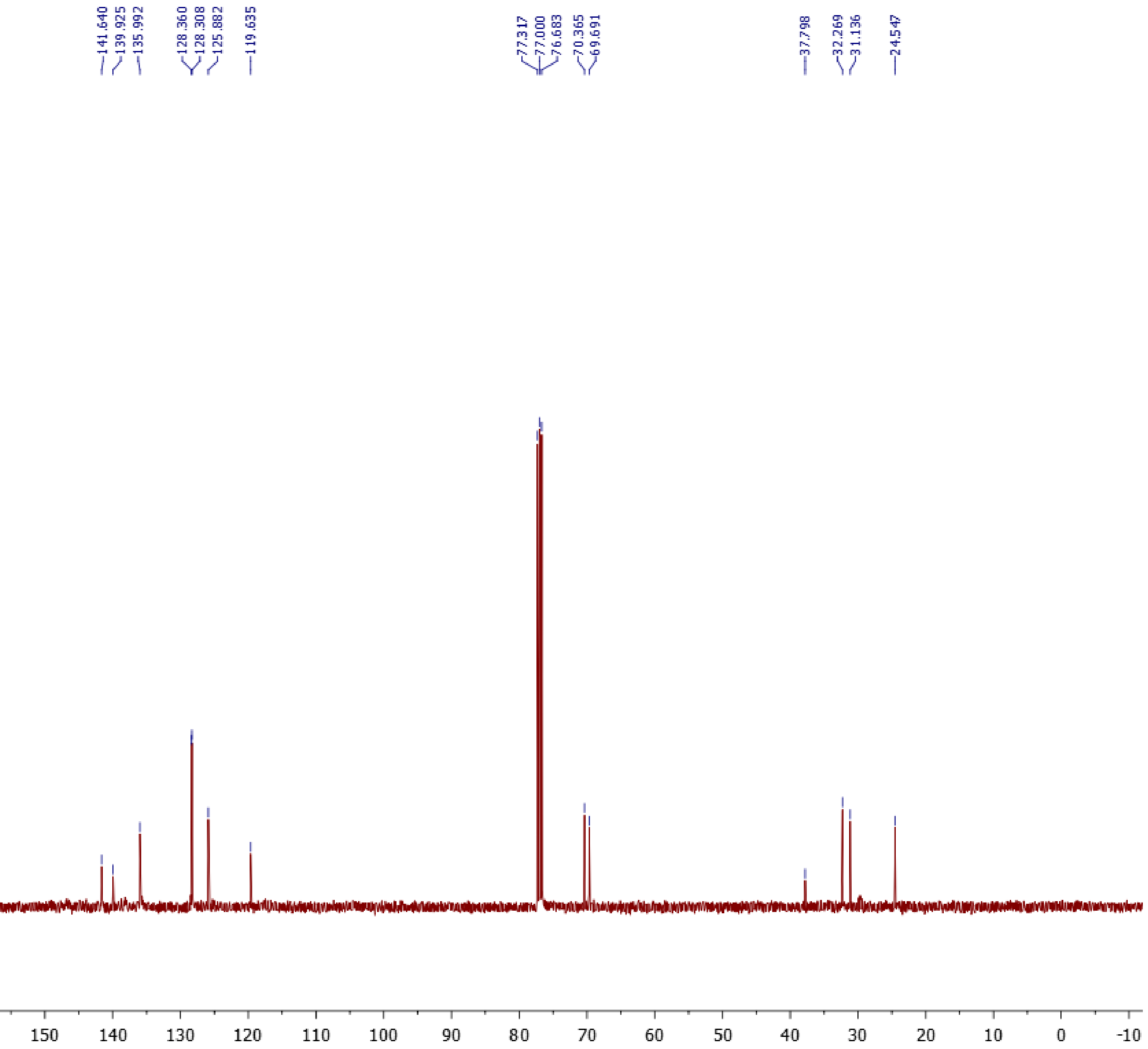

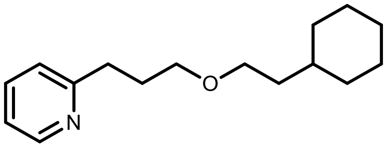

28
^{1}H NMR
(500 MHz, $CDCl_3$)

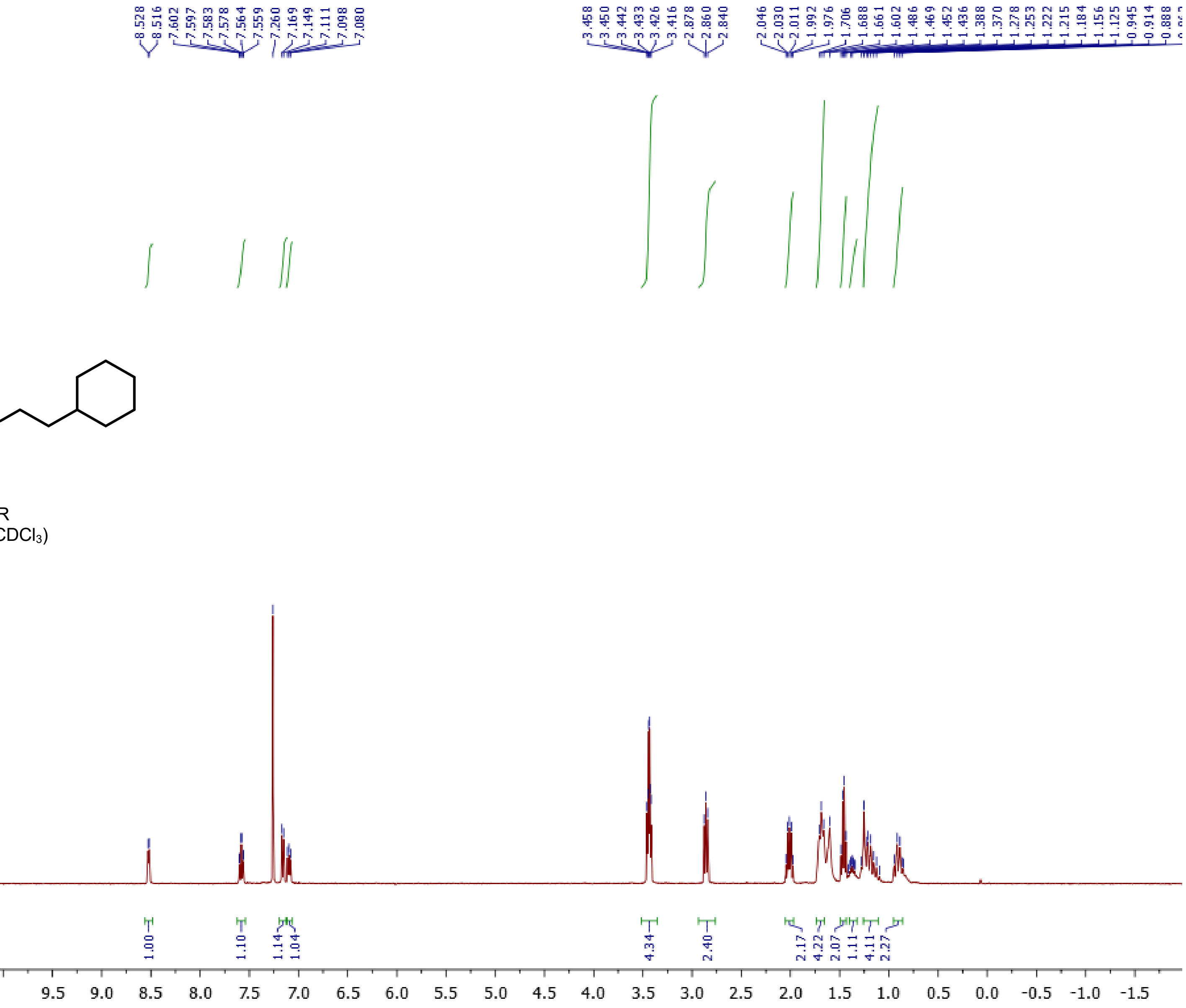

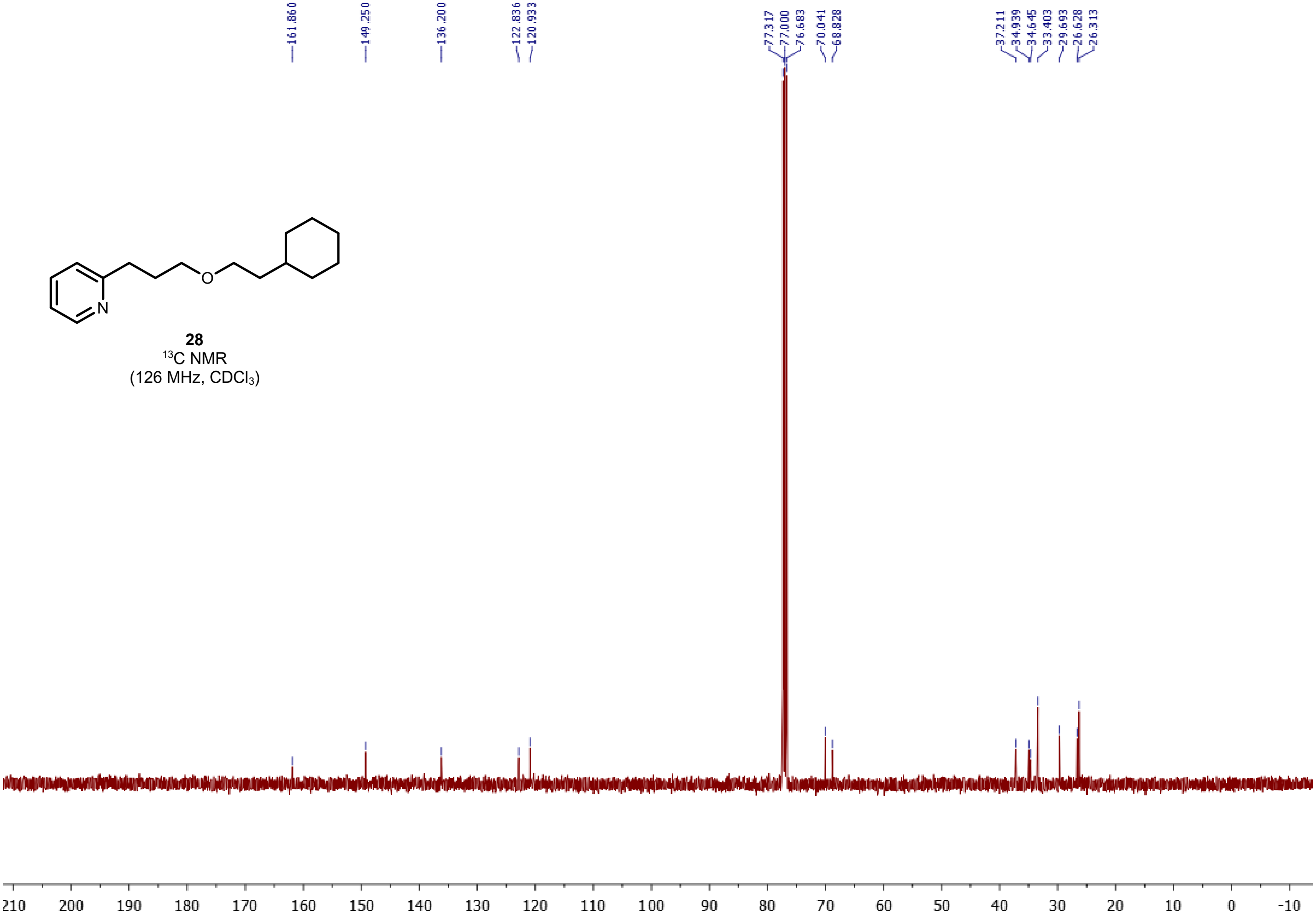

28
^{13}C NMR
(126 MHz, $CDCl_3$)

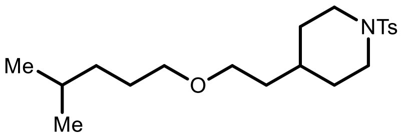

32
^{1}H NMR
(500 MHz, $CDCl_3$)

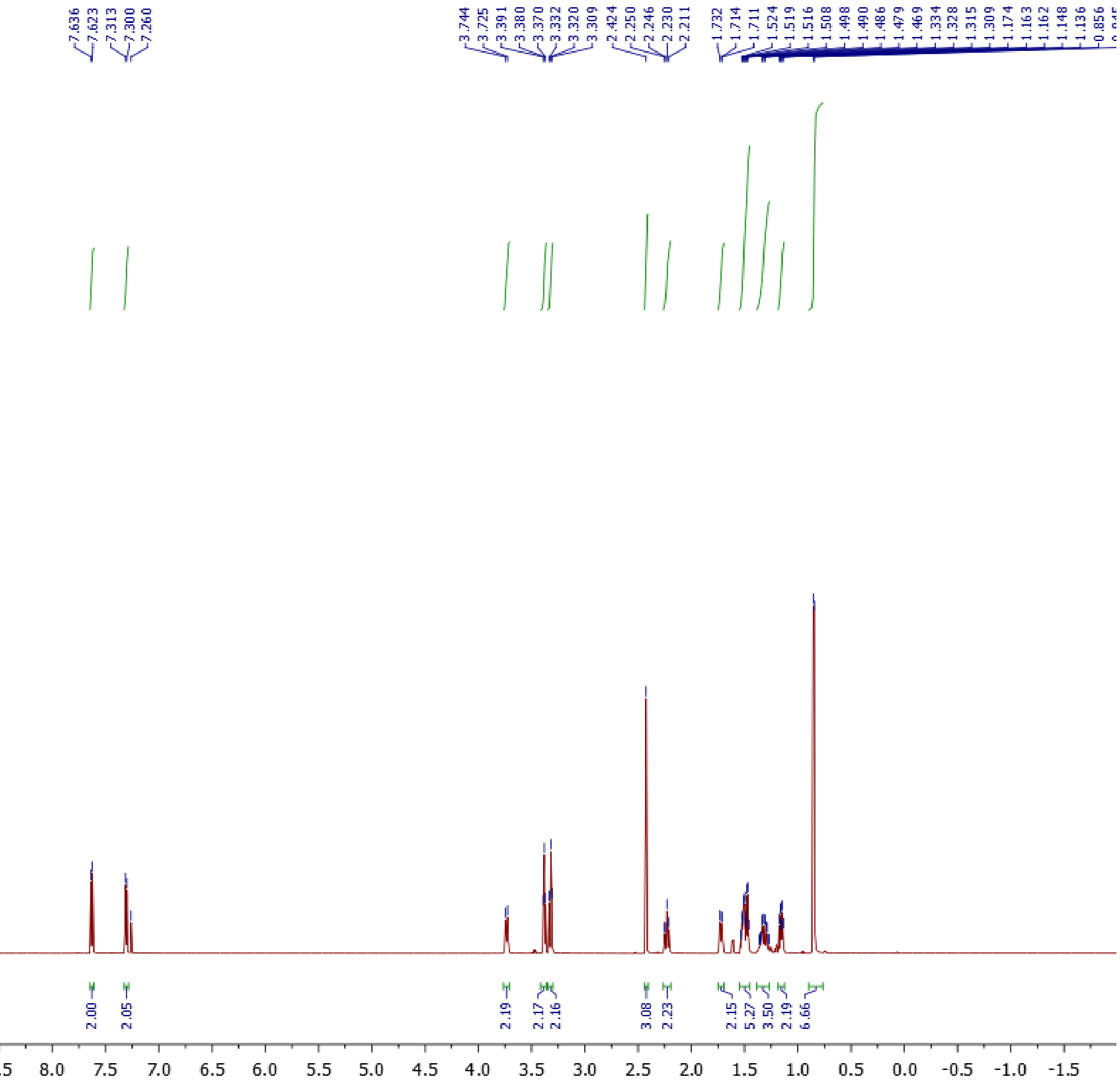

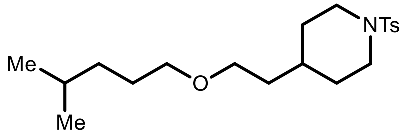

32
^{13}C NMR
(126 MHz, $CDCl_3$)

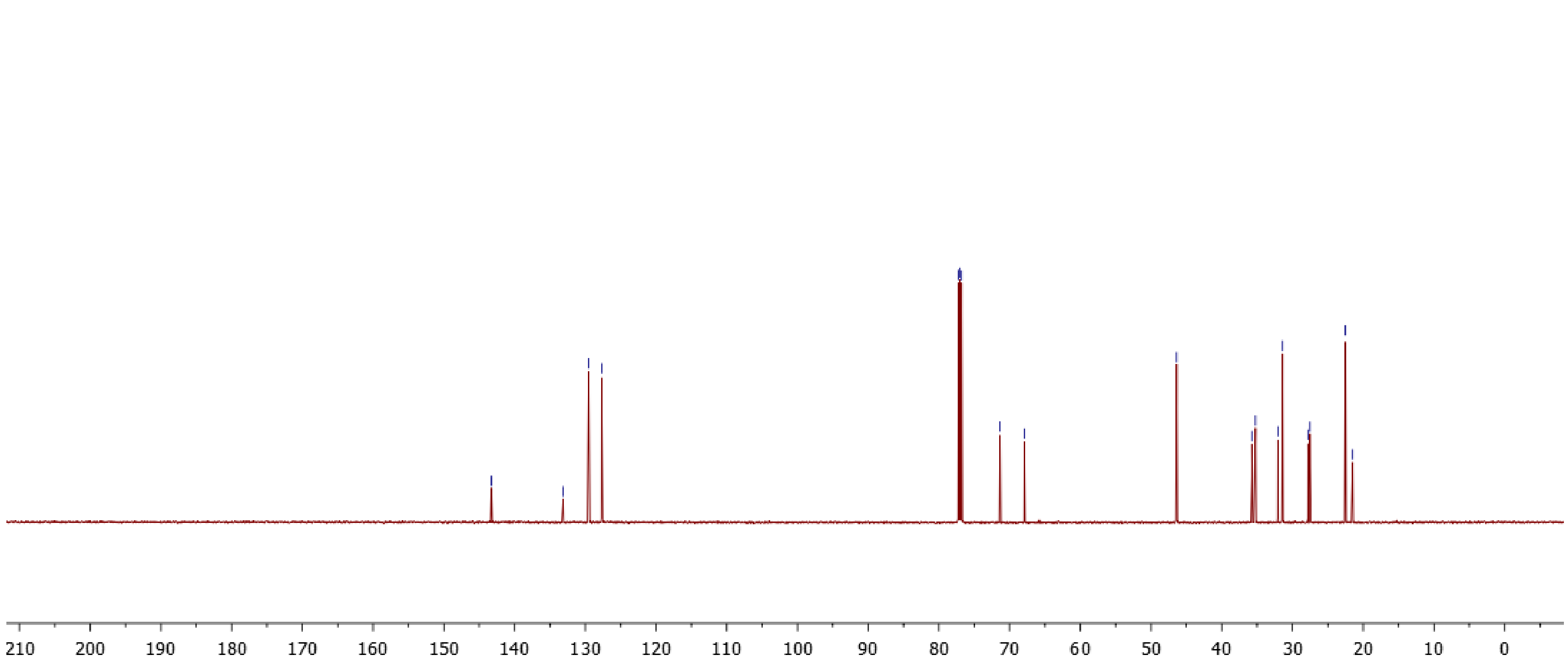

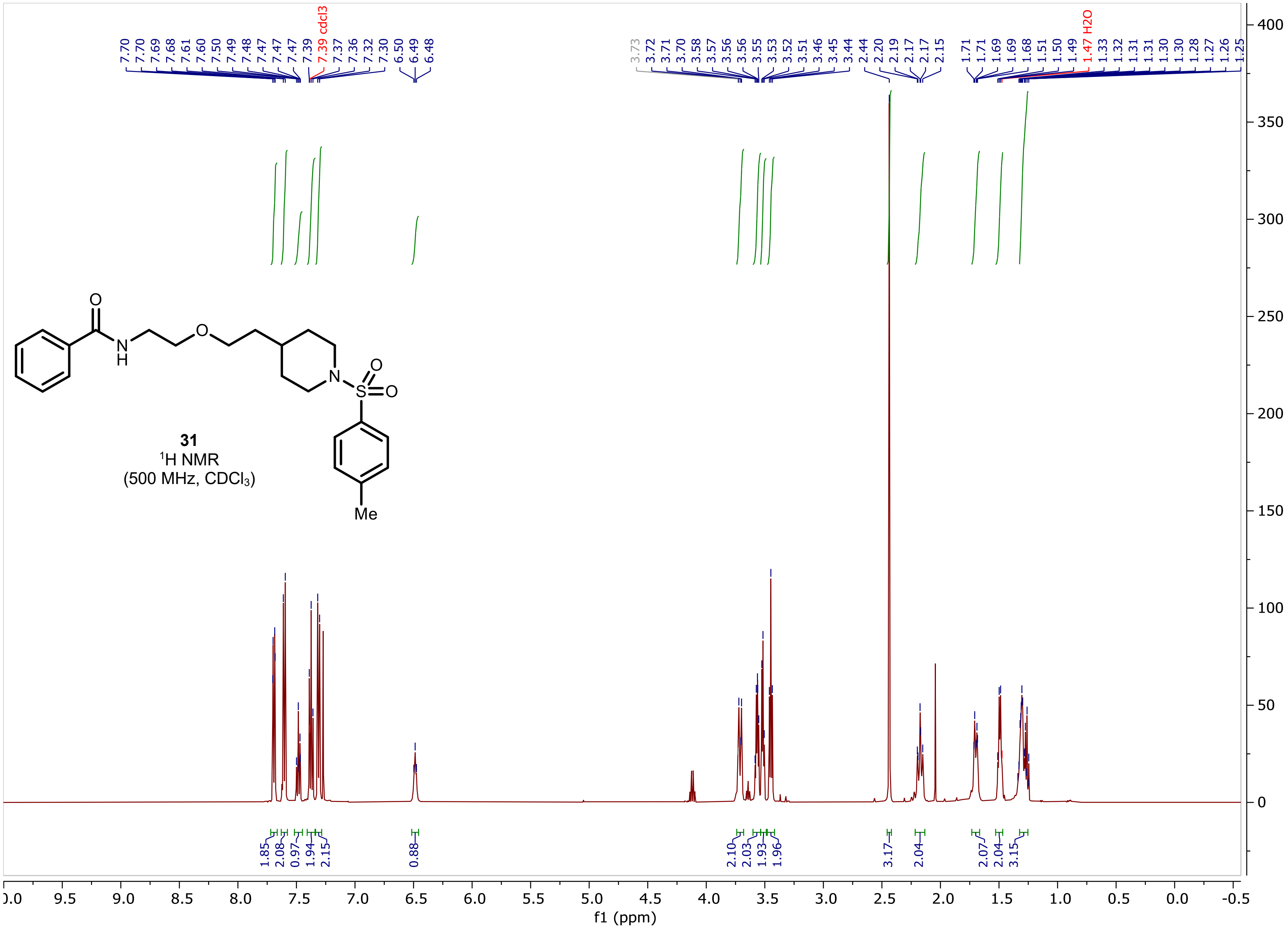
31
1H NMR
(500 MHz, CDCl3)
Me
f1 (ppm)
7.39 cdcl3
1.47 H2O

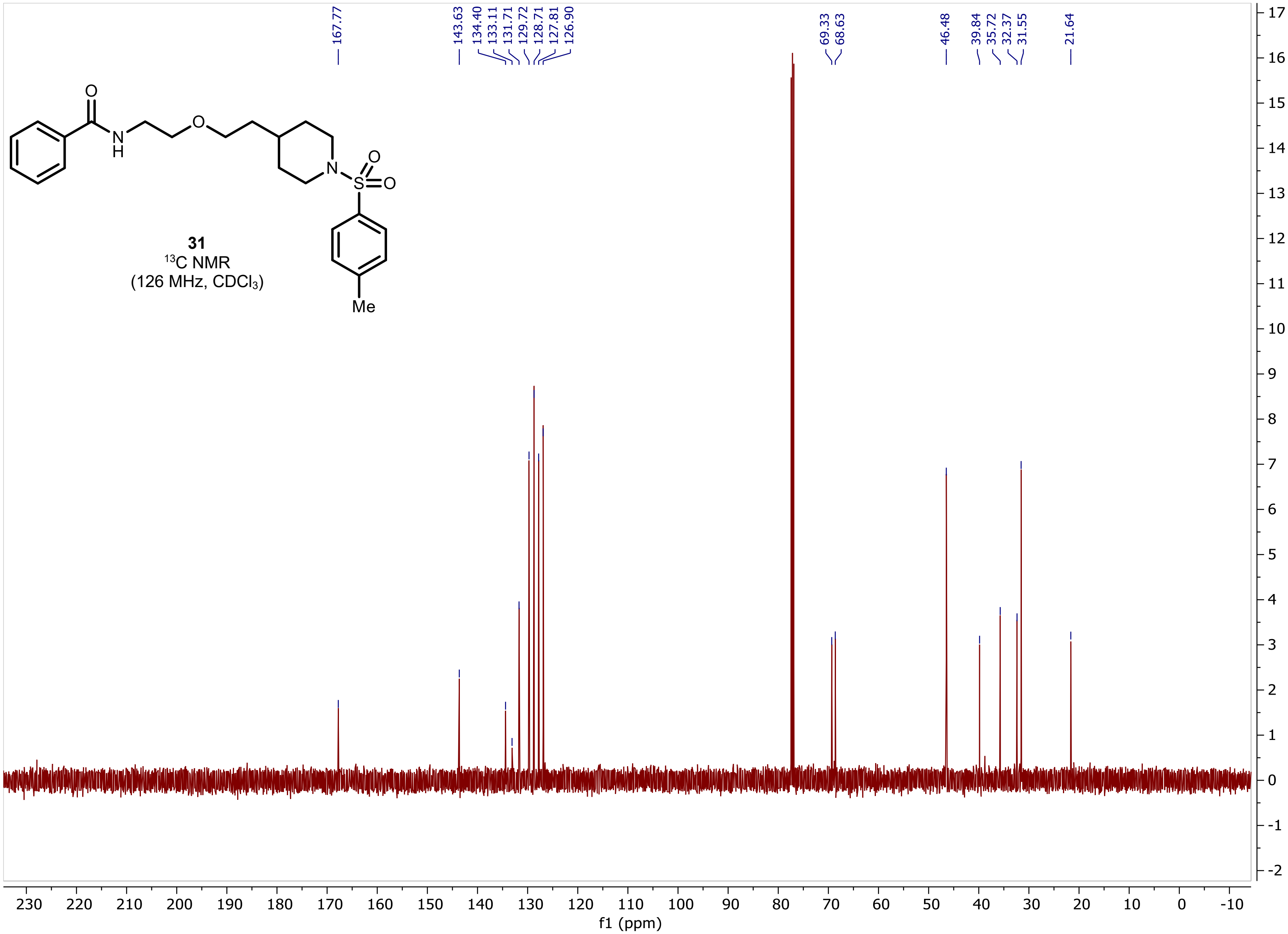

31
^{13}C NMR
(126 MHz, $CDCl_3$)
O
N
H
O
N
S
O
O
Me
167.77
143.63
134.40
133.11
131.71
129.72
128.71
127.81
126.90
69.33
68.63
46.48
39.84
35.72
32.37
31.55
21.64
f1 (ppm)

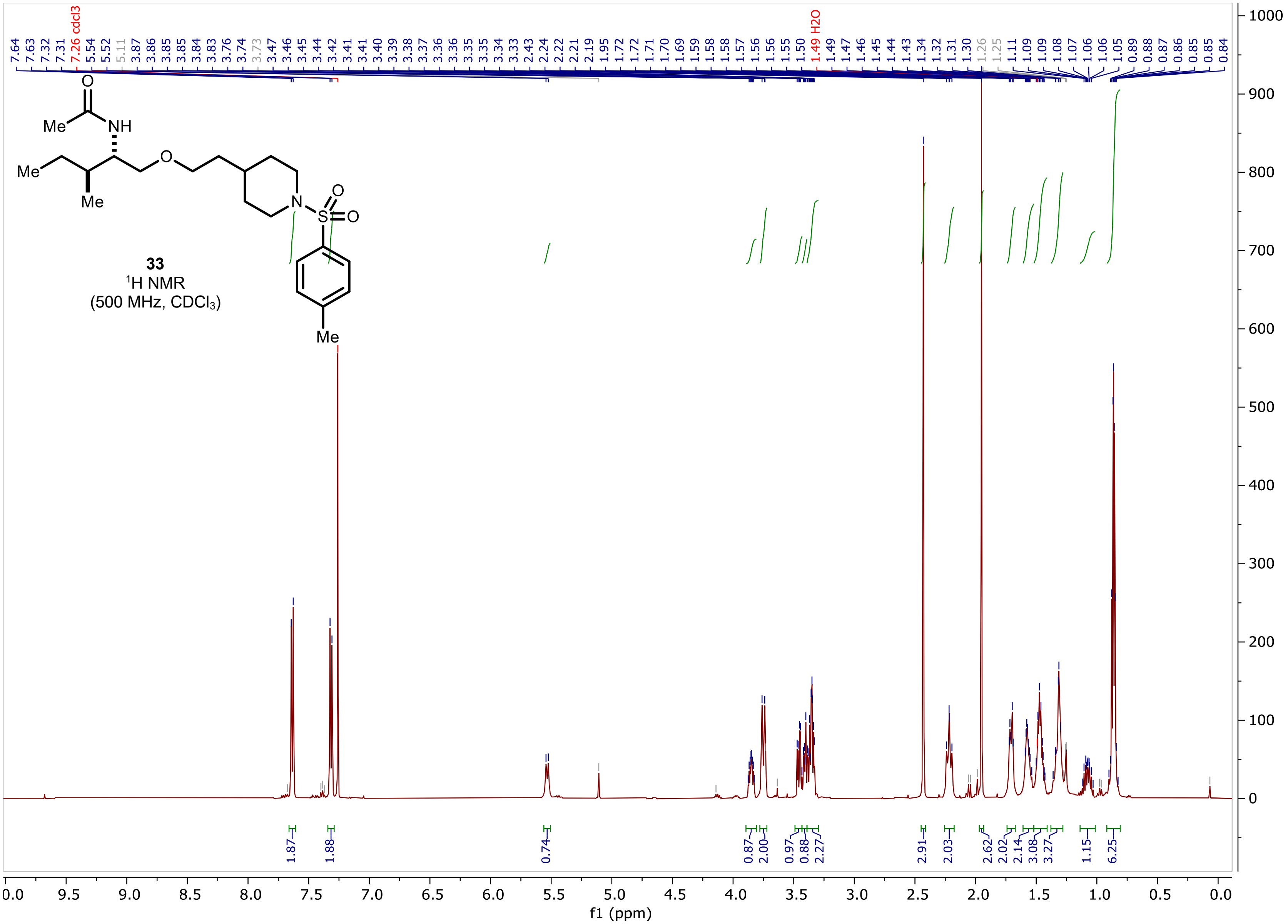
33
^{1}H NMR
(500 MHz, CDCl$_3$)
f1 (ppm)

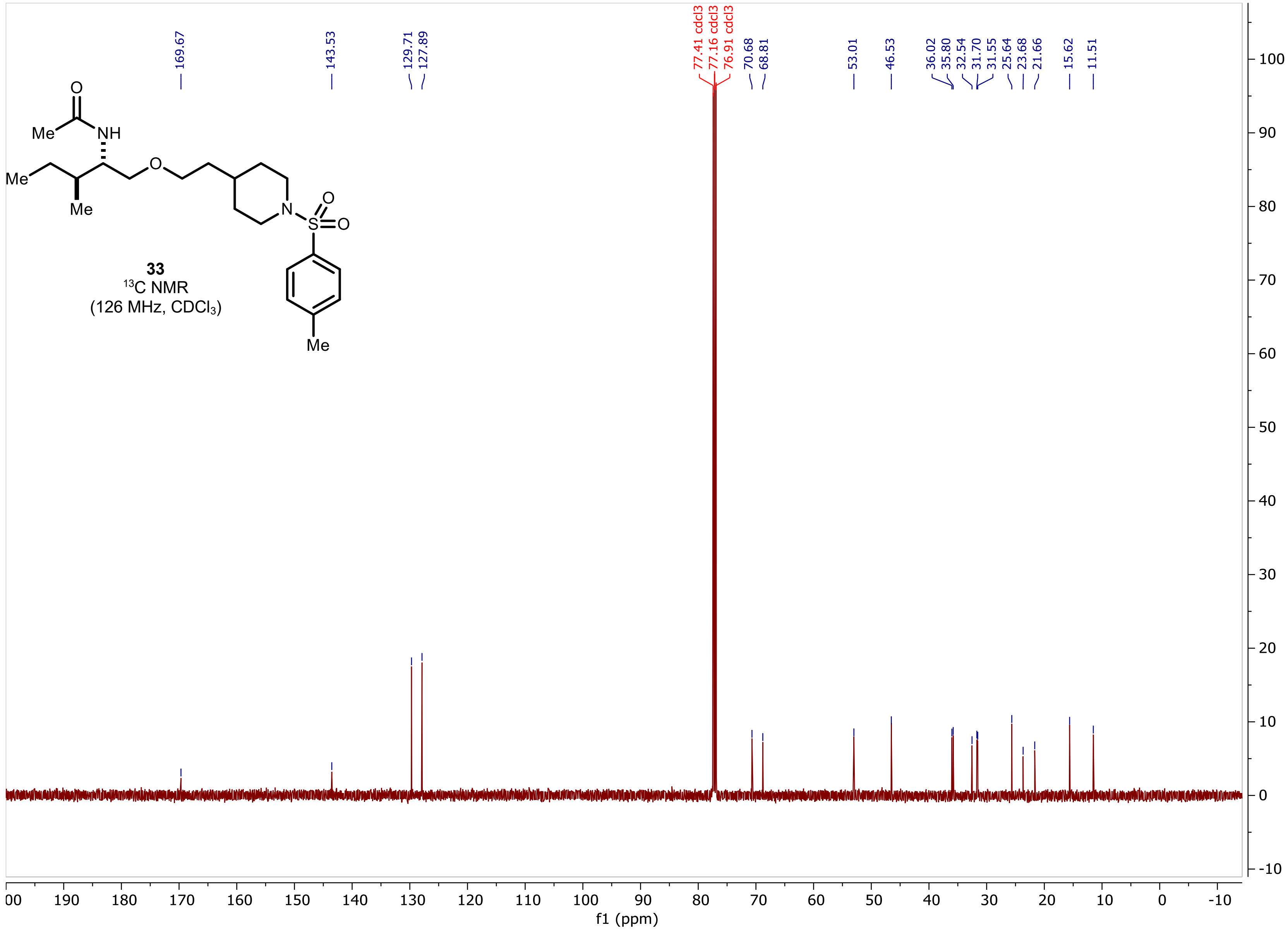

33
^{13}C NMR
(126 MHz, CDCl$_3$)
169.67
143.53
129.71
127.89
77.41 cdcl3
77.16 cdcl3
76.91 cdcl3
70.68
68.81
53.01
46.53
36.02
35.80
32.54
31.70
31.55
25.64
23.68
21.66
15.62
11.51
f1 (ppm)

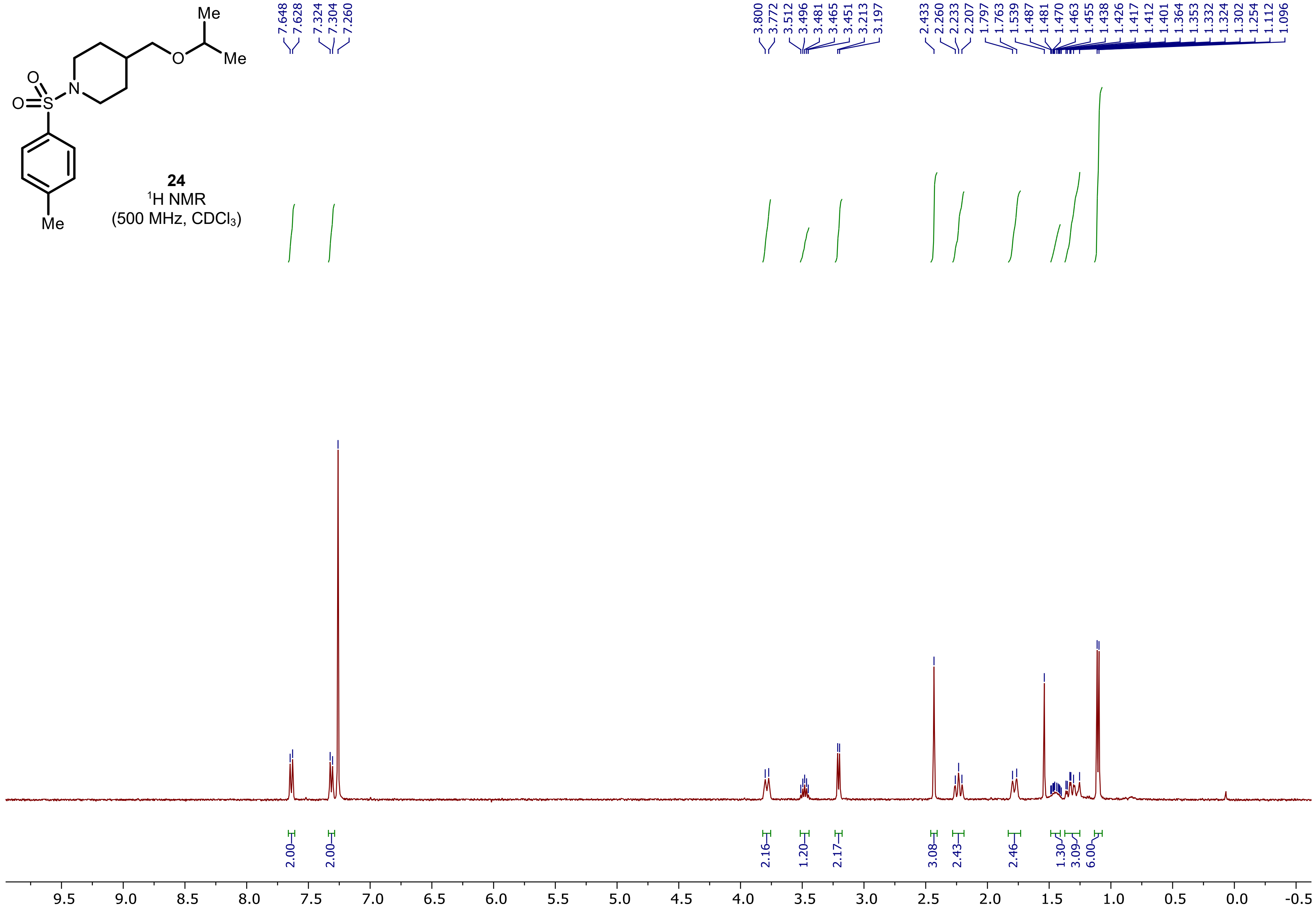

24
^{1}H NMR
(500 MHz, $CDCl_3$)

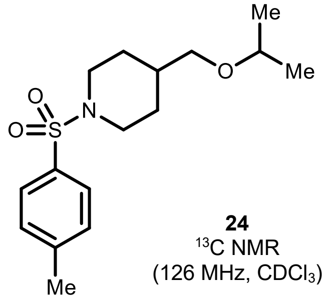

24
^{13}C NMR
(126 MHz, $CDCl_3$)

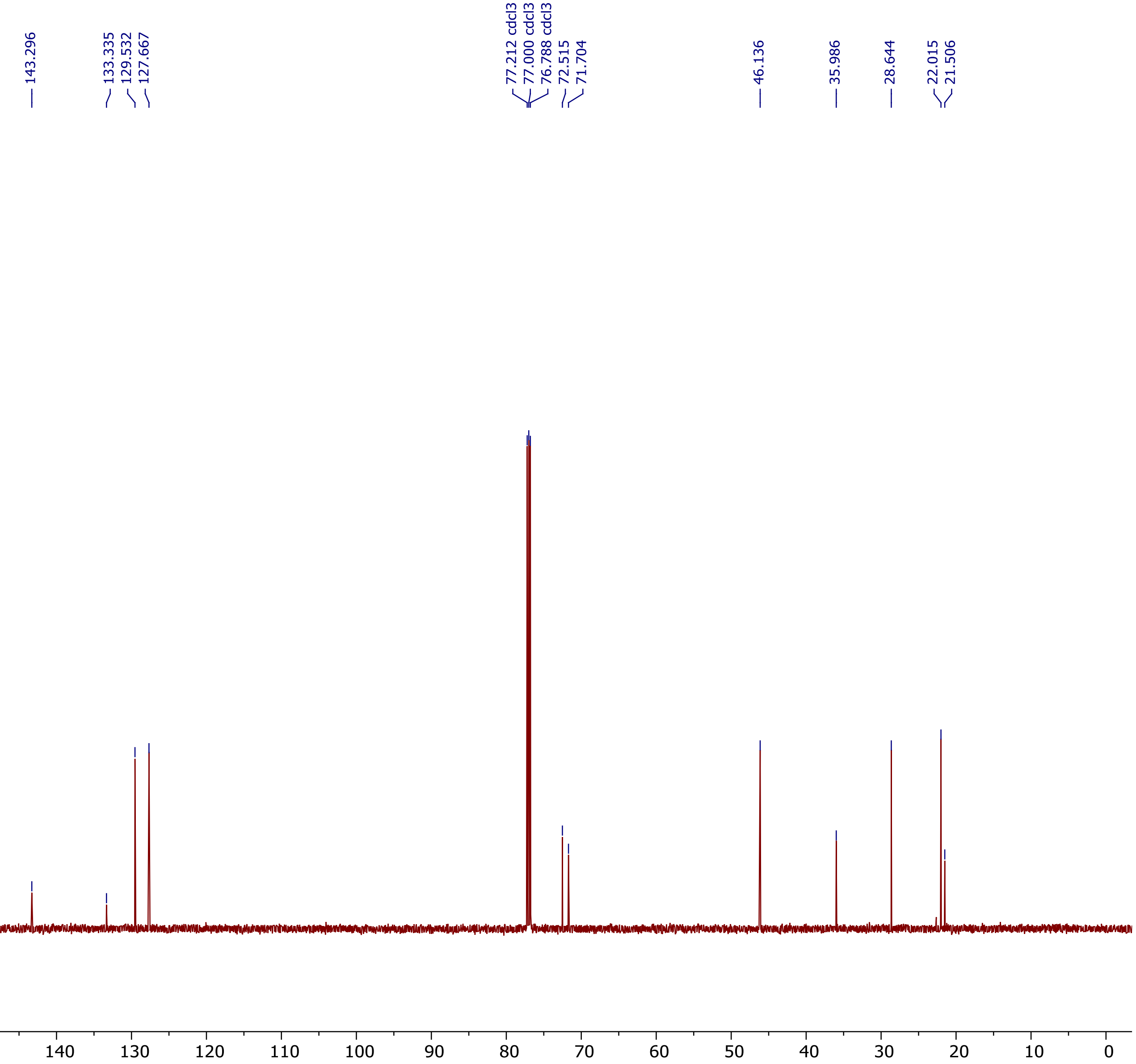

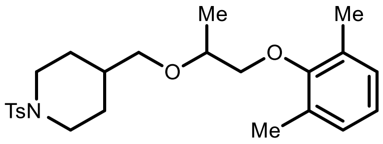

9
^{1}H NMR
(500 MHz, $CDCl_3$)

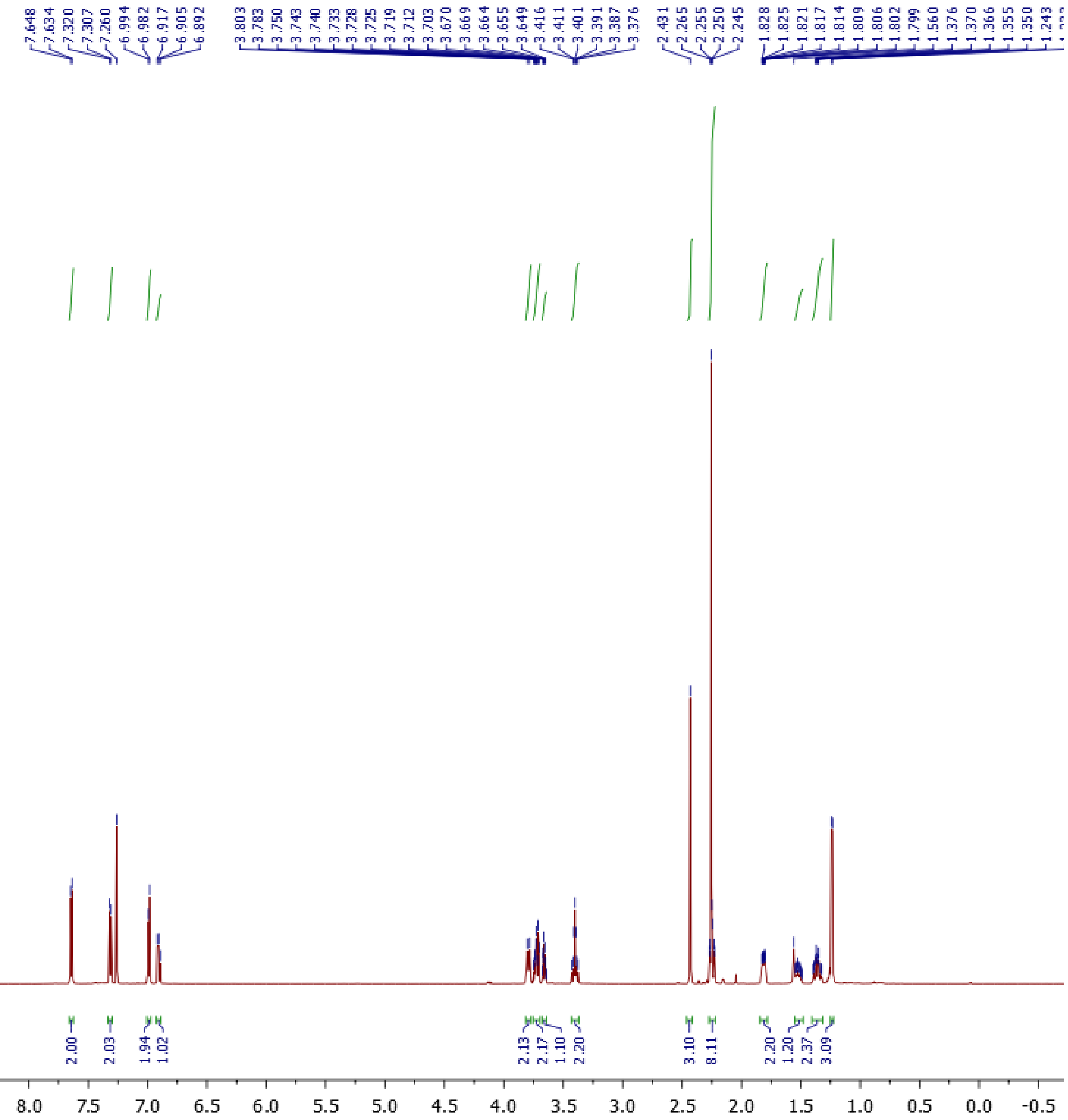

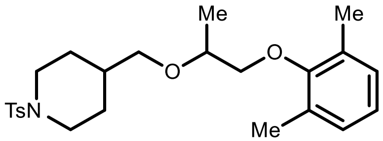

9
^{13}C NMR
(126 MHz, $CDCl_3$)

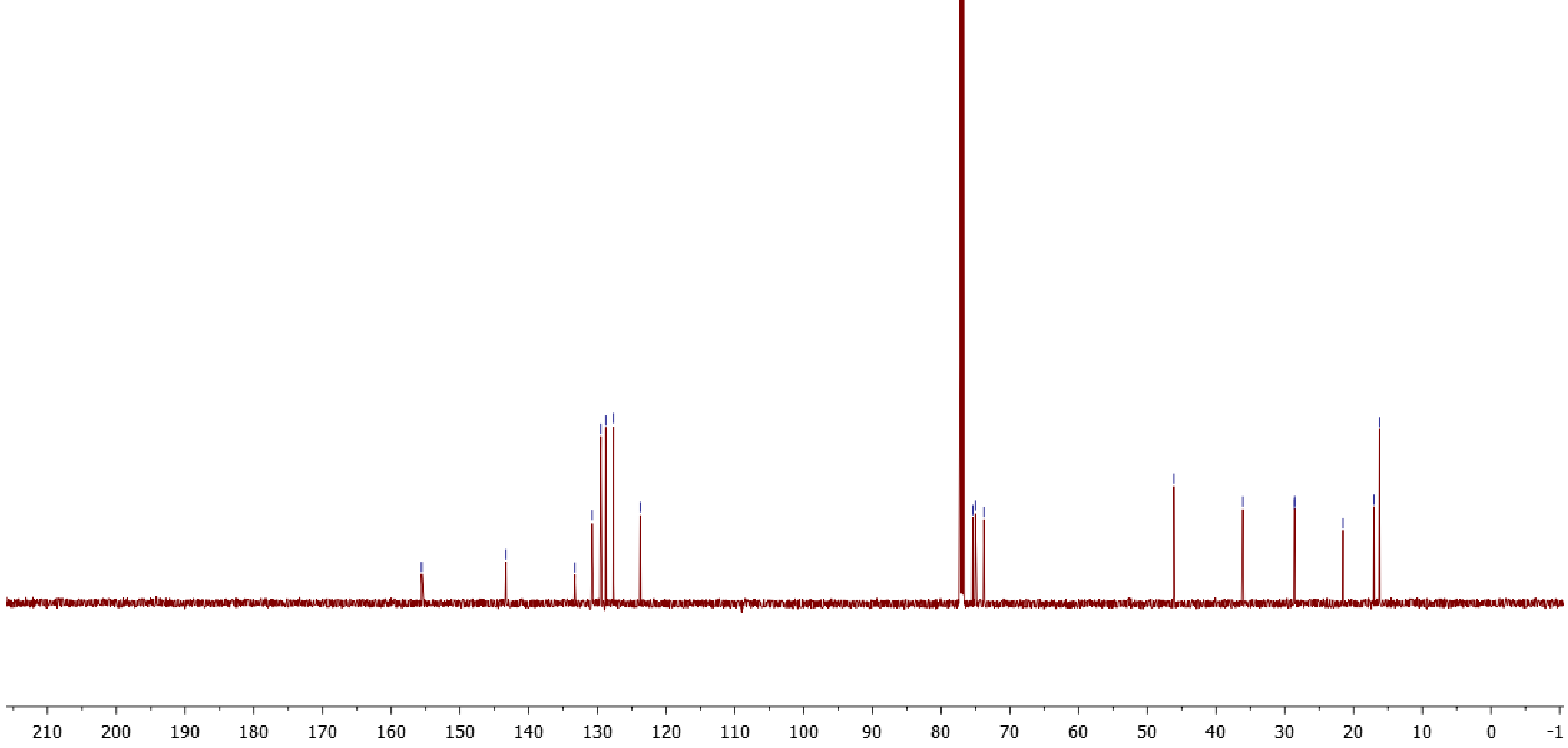

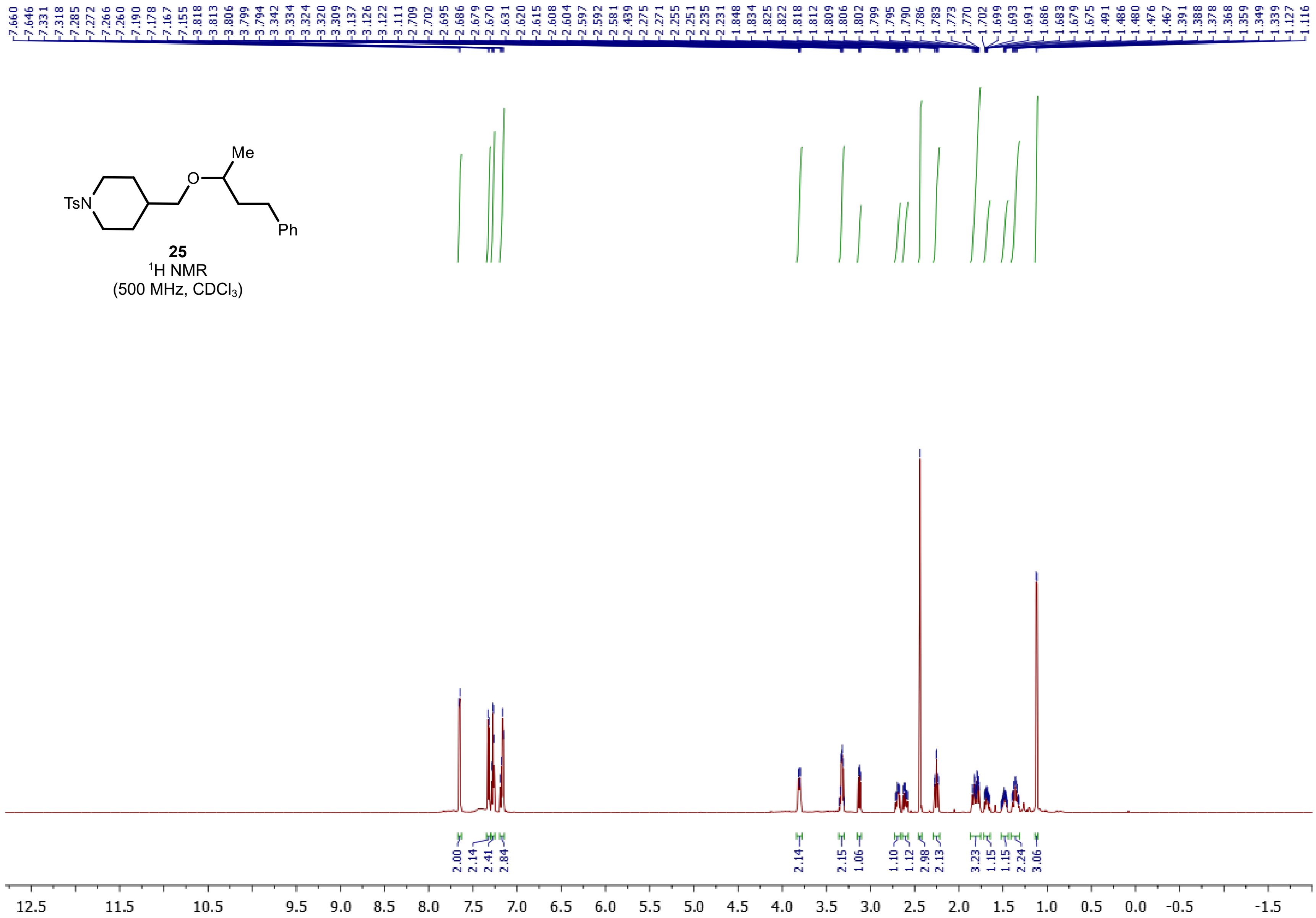
7.660
7.646
7.331
7.318
7.285
7.272
7.266
7.260
7.190
7.178
7.167
7.155
3.818
3.813
3.806
3.799
3.794
3.342
3.334
3.324
3.320
3.309
3.137
3.126
3.122
3.111
2.709
2.702
2.695
2.686
2.679
2.670
2.631
2.620
2.615
2.608
2.604
2.597
2.592
2.581
2.439
2.275
2.271
2.255
2.251
2.235
2.231
1.848
1.834
1.825
1.822
1.818
1.812
1.809
1.806
1.802
1.799
1.795
1.790
1.786
1.783
1.773
1.770
1.702
1.699
1.693
1.691
1.686
1.683
1.679
1.675
1.491
1.486
1.480
1.476
1.467
1.391
1.388
1.378
1.368
1.359
1.349
1.339
1.127
1.116
Me
O
TsN
Ph
25
1H NMR
(500 MHz, CDCl3)
2.00
2.14
2.41
2.84
2.14
2.15
1.06
1.10
1.12
2.98
2.13
3.23
1.15
1.15
2.24
3.06
12.5
11.5
10.5
9.5
9.0
8.5
8.0
7.5
7.0
6.5
6.0
5.5
5.0
4.5
4.0
3.5
3.0
2.5
2.0
1.5
1.0
0.5
0.0
-0.5
-1.5

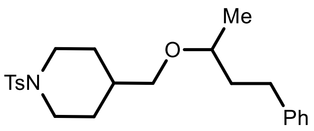

25
^{13}C NMR
(126 MHz, $CDCl_3$)

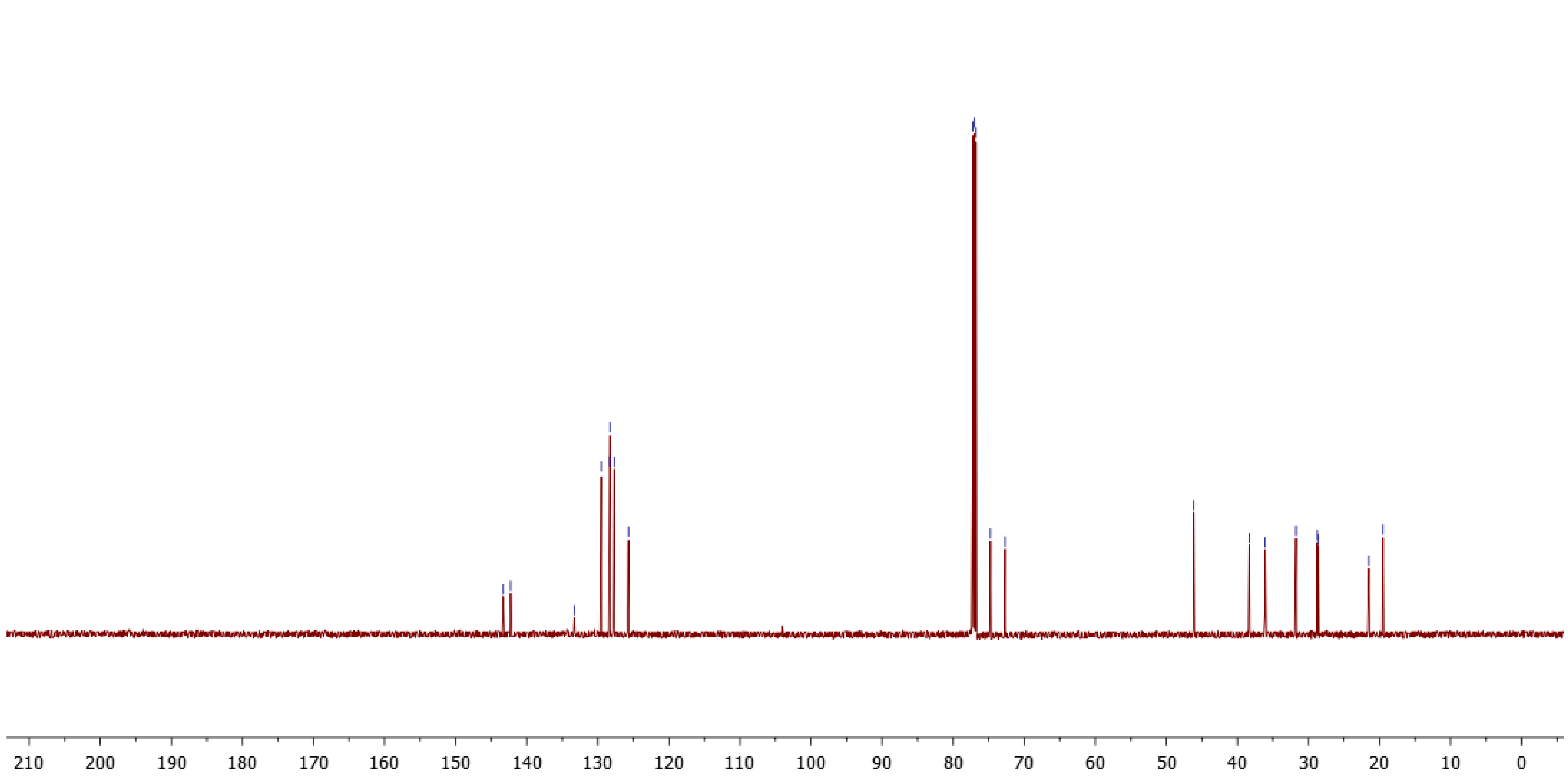

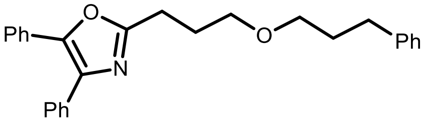

29
^{1}H NMR
(500 MHz, $CDCl_3$)

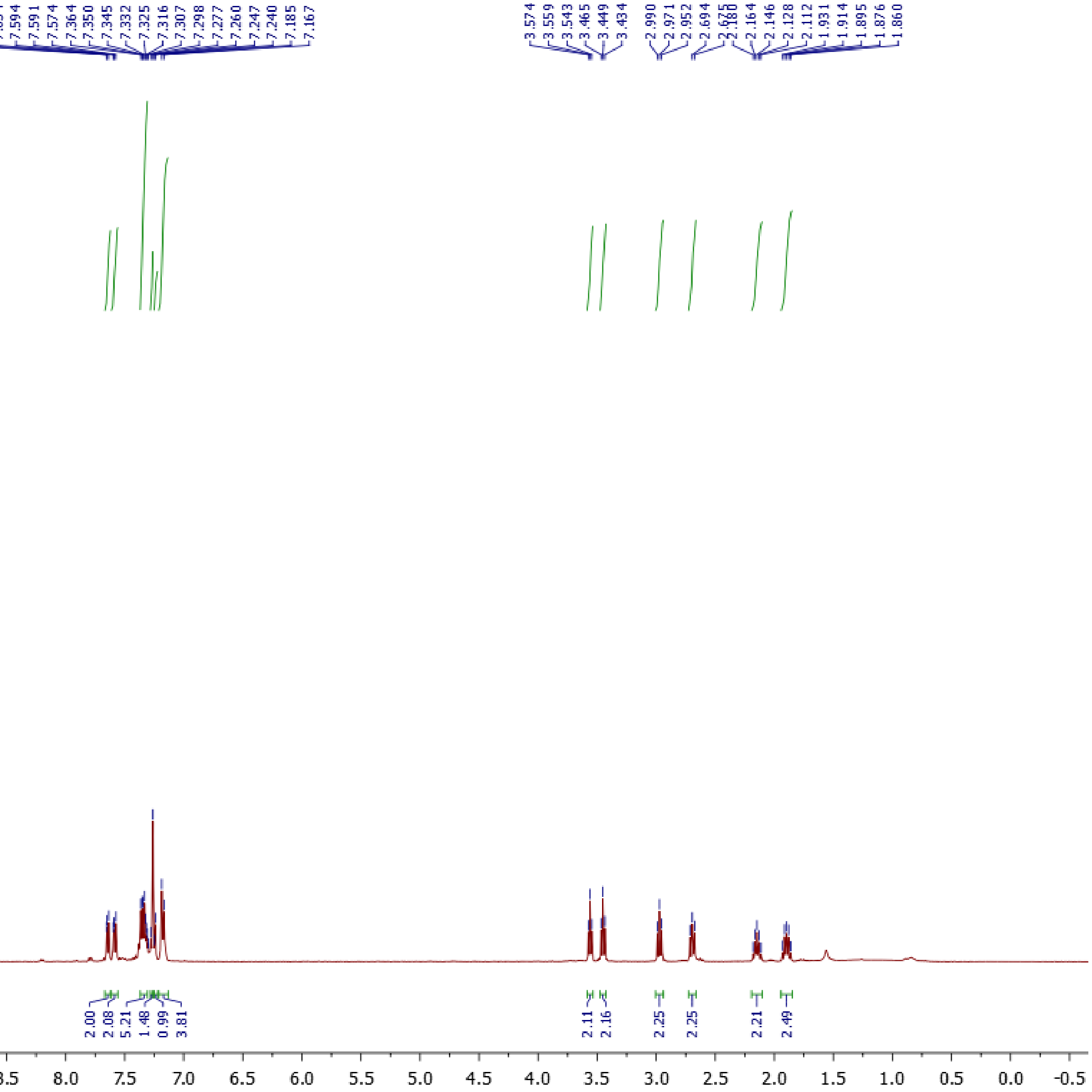

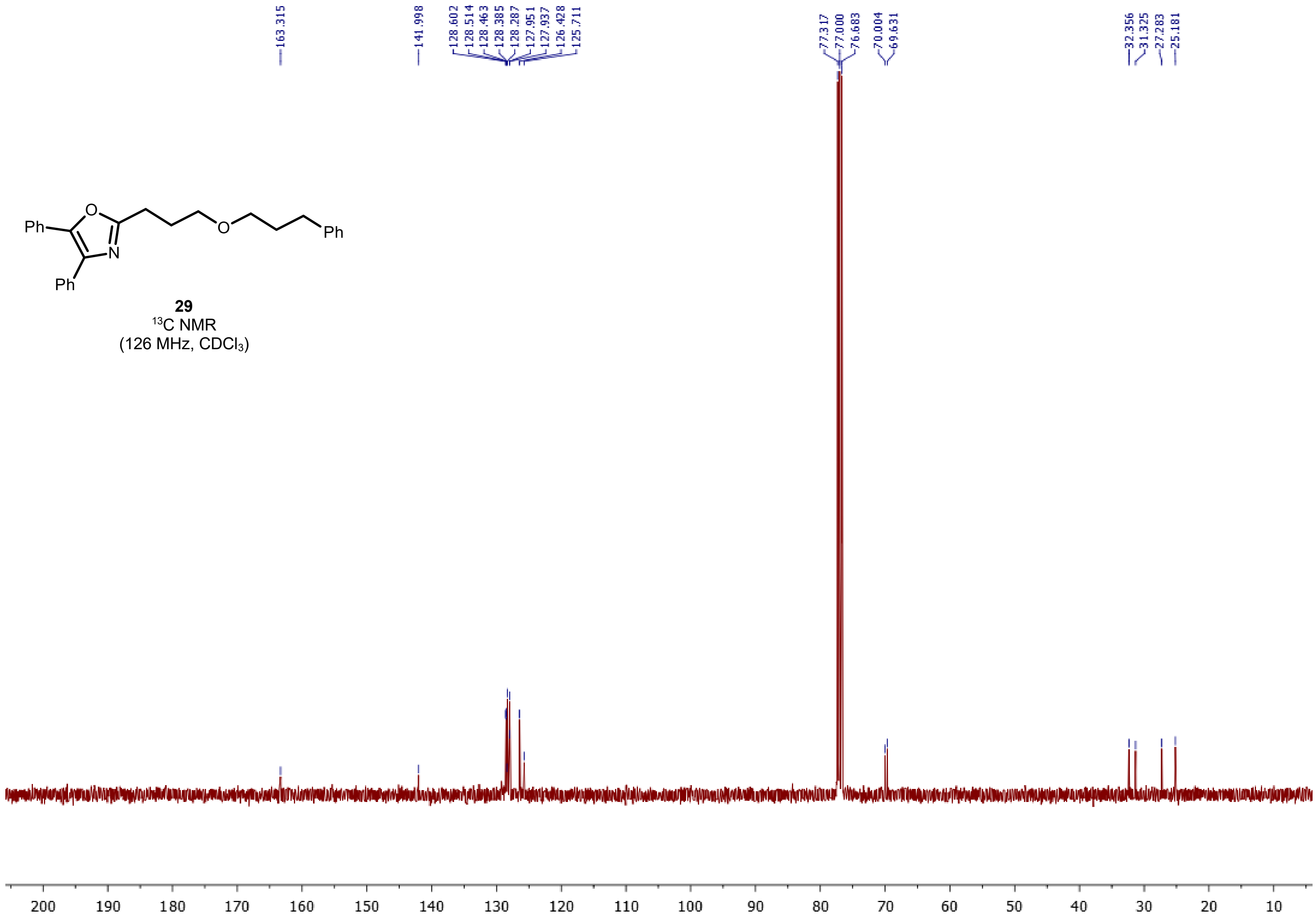

29
^{13}C NMR
(126 MHz, $CDCl_3$)

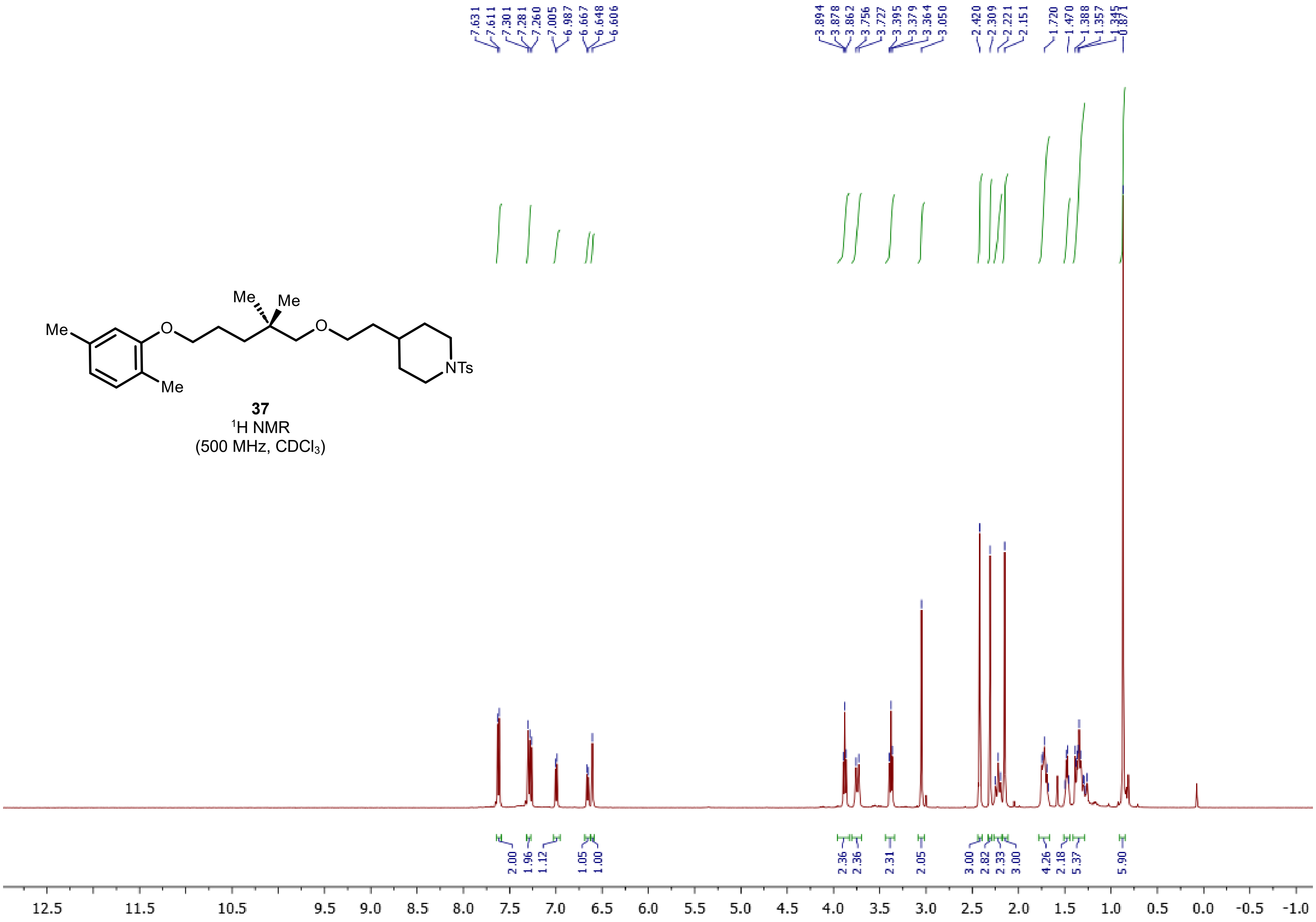

37
^{1}H NMR
(500 MHz, $CDCl_3$)

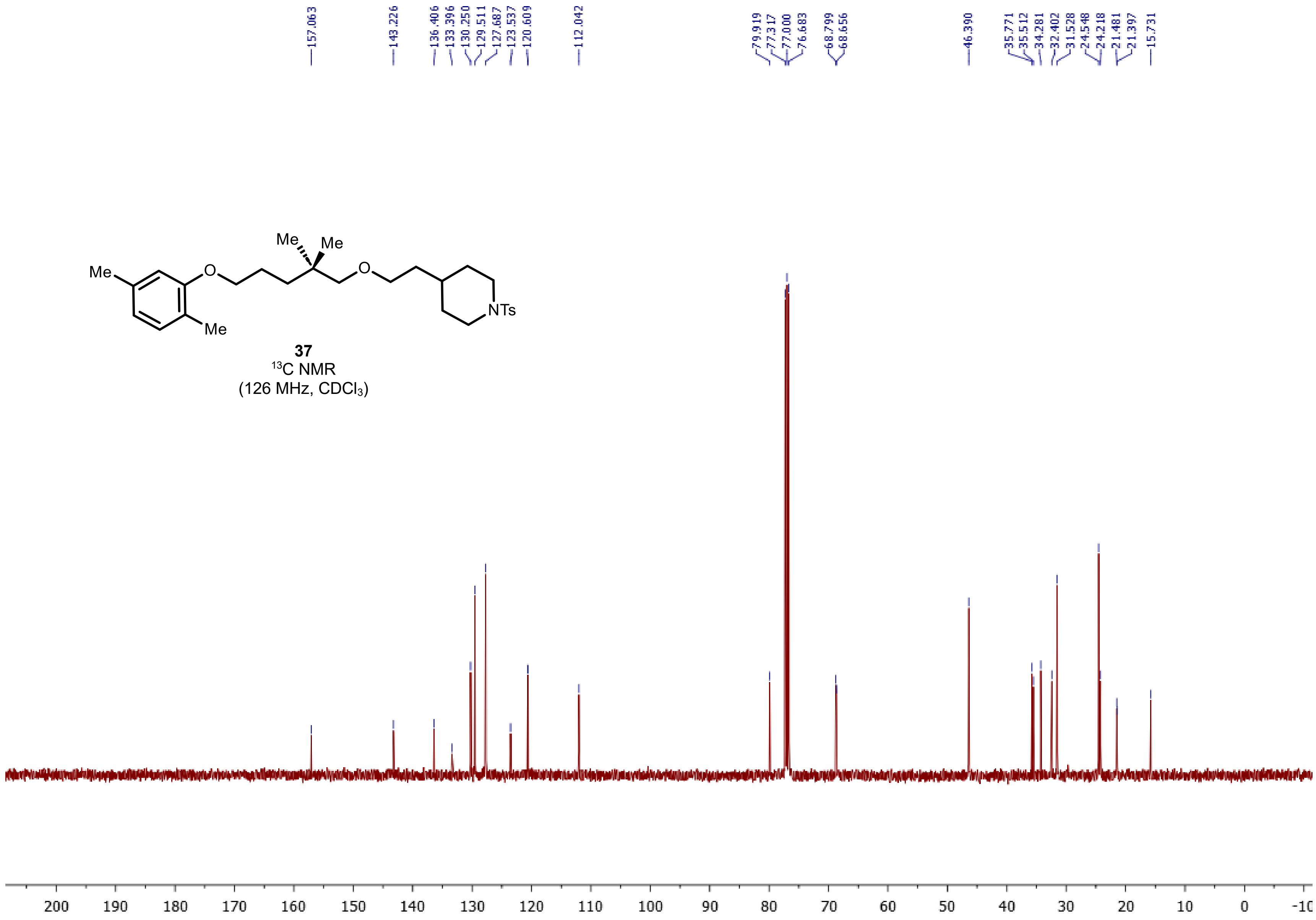
157.063
143.226
136.406
133.396
130.250
129.511
127.687
123.537
120.609
112.042
79.919
77.317
77.000
76.683
68.799
68.656
46.390
35.771
35.512
34.281
32.402
31.528
24.548
24.218
21.481
21.397
15.731
Me
Me
Me
O
O
NTs
Me
37
13C NMR
(126 MHz, CDCl3)
200 190 180 170 160 150 140 130 120 110 100 90 80 70 60 50 40 30 20 10 0 -10

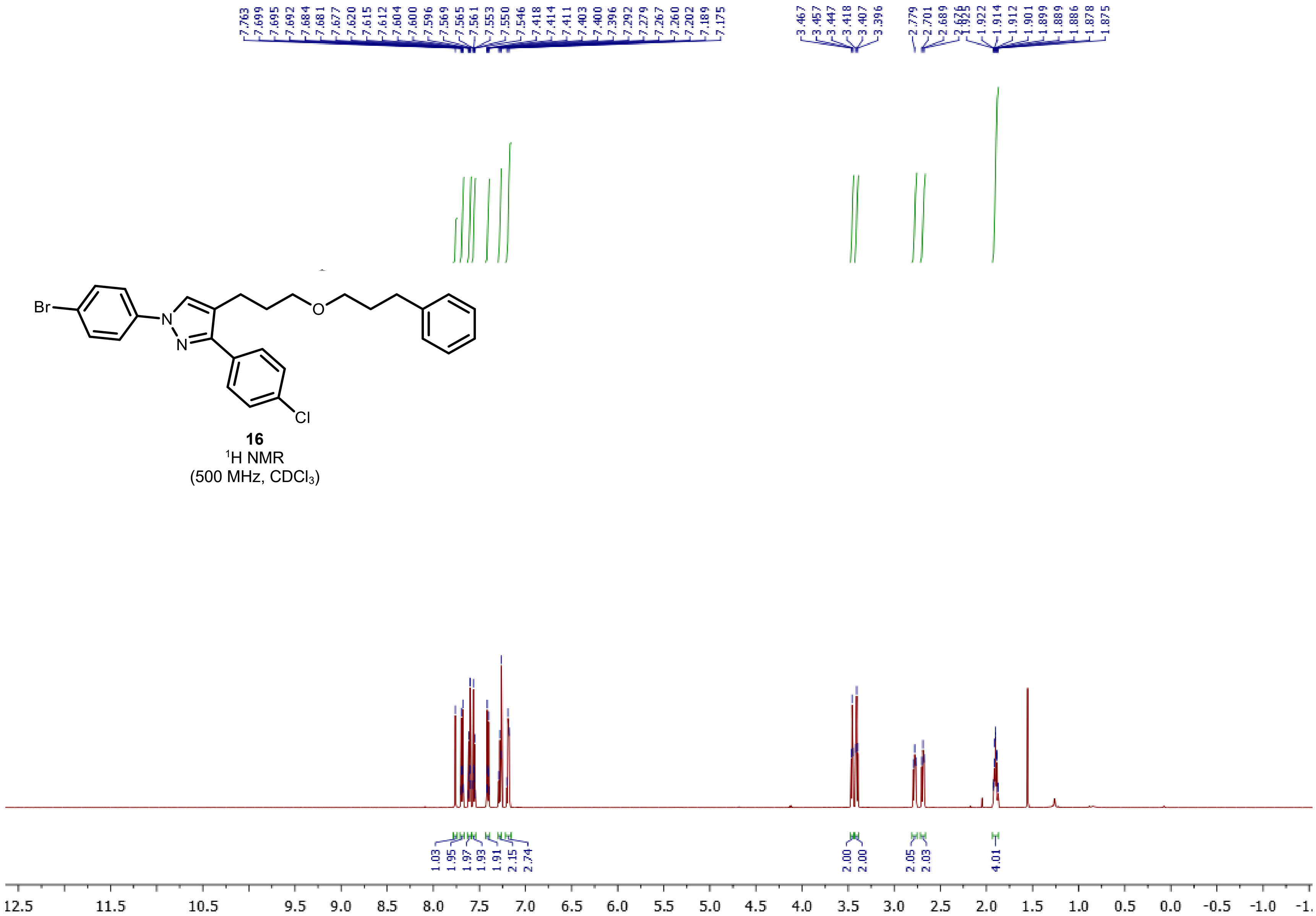

16
^{1}H NMR
(500 MHz, $CDCl_3$)

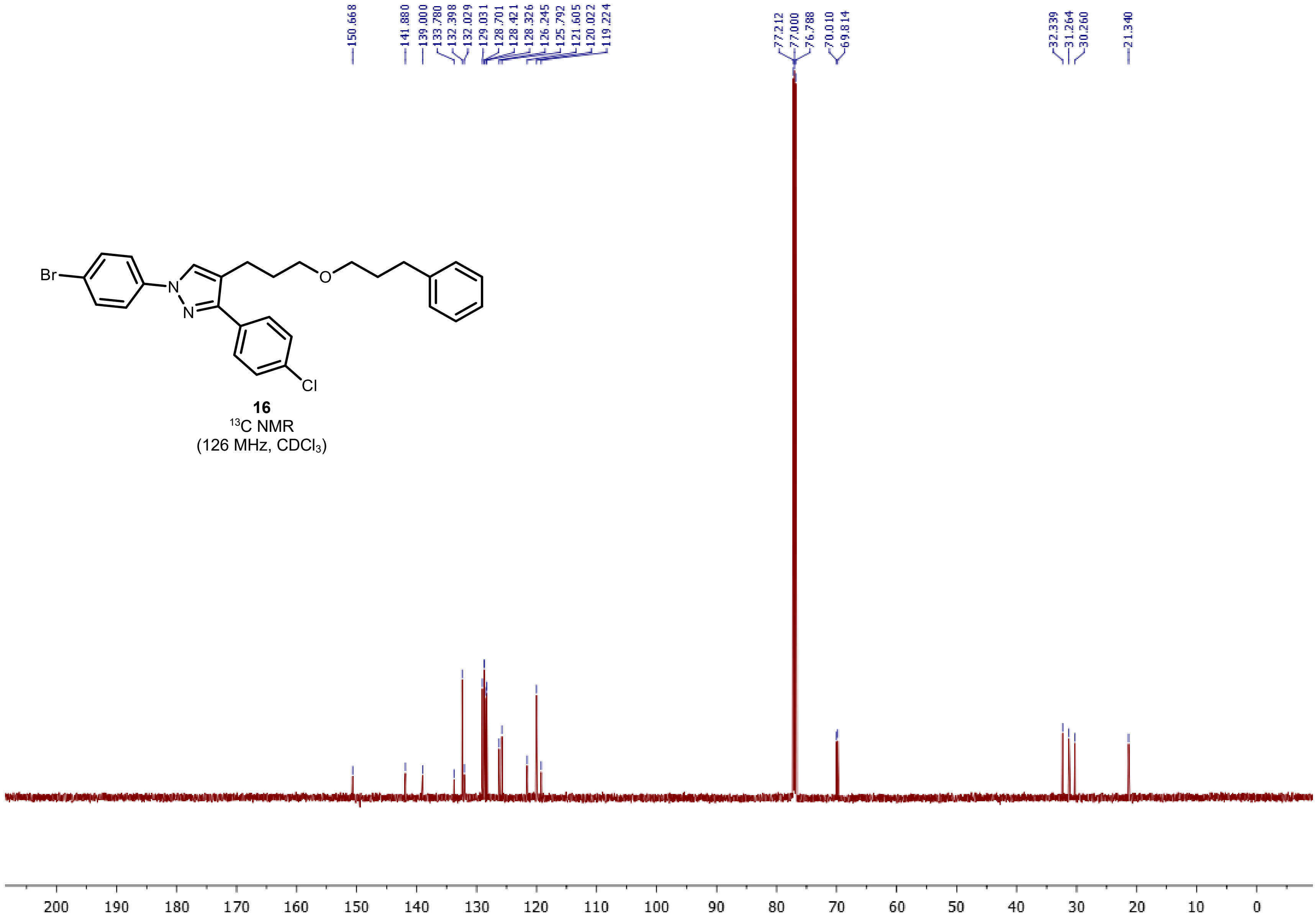

16
^{13}C NMR
(126 MHz, $CDCl_3$)

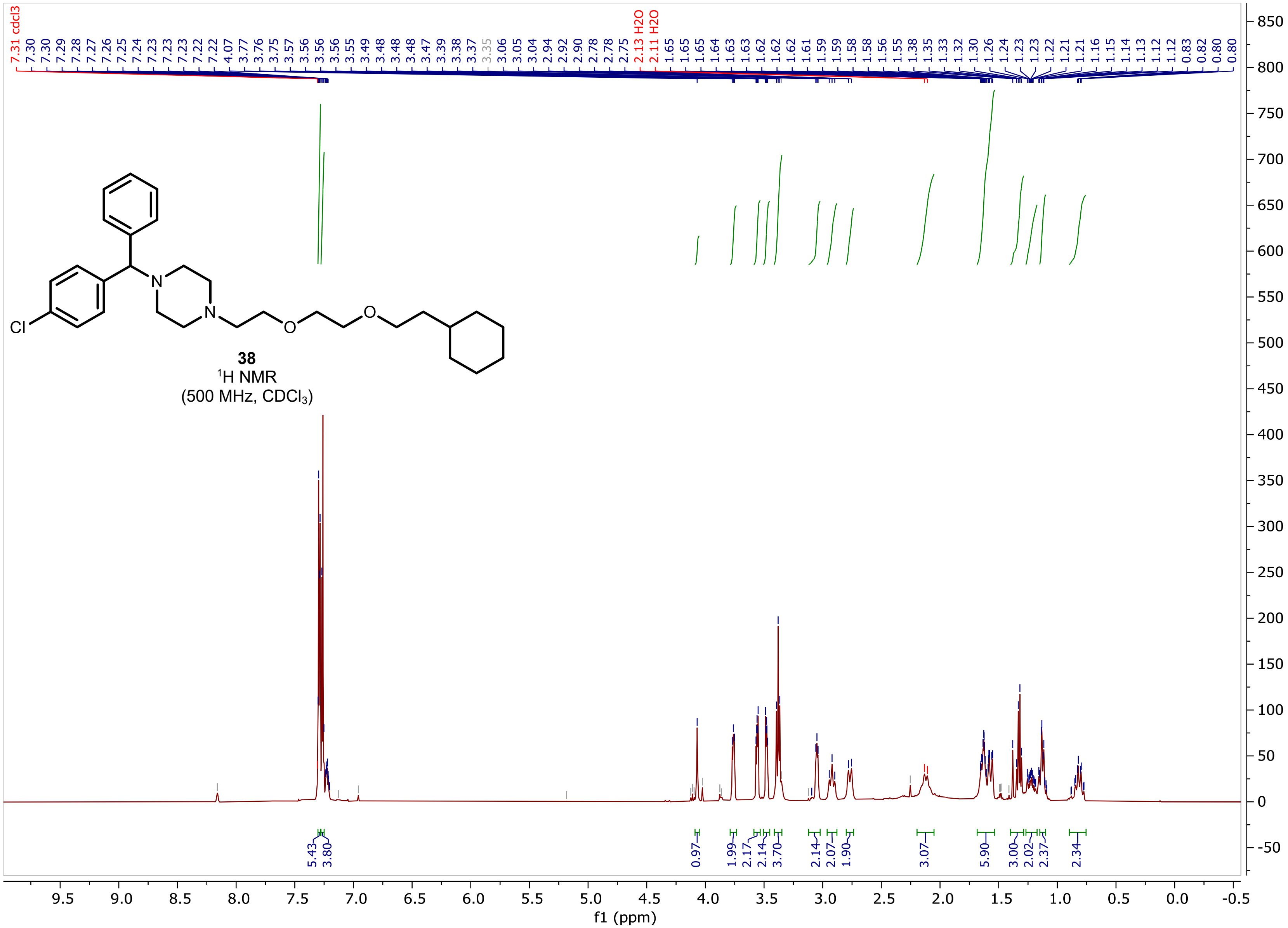
38
¹H NMR
(500 MHz, CDCl₃)
f1 (ppm)

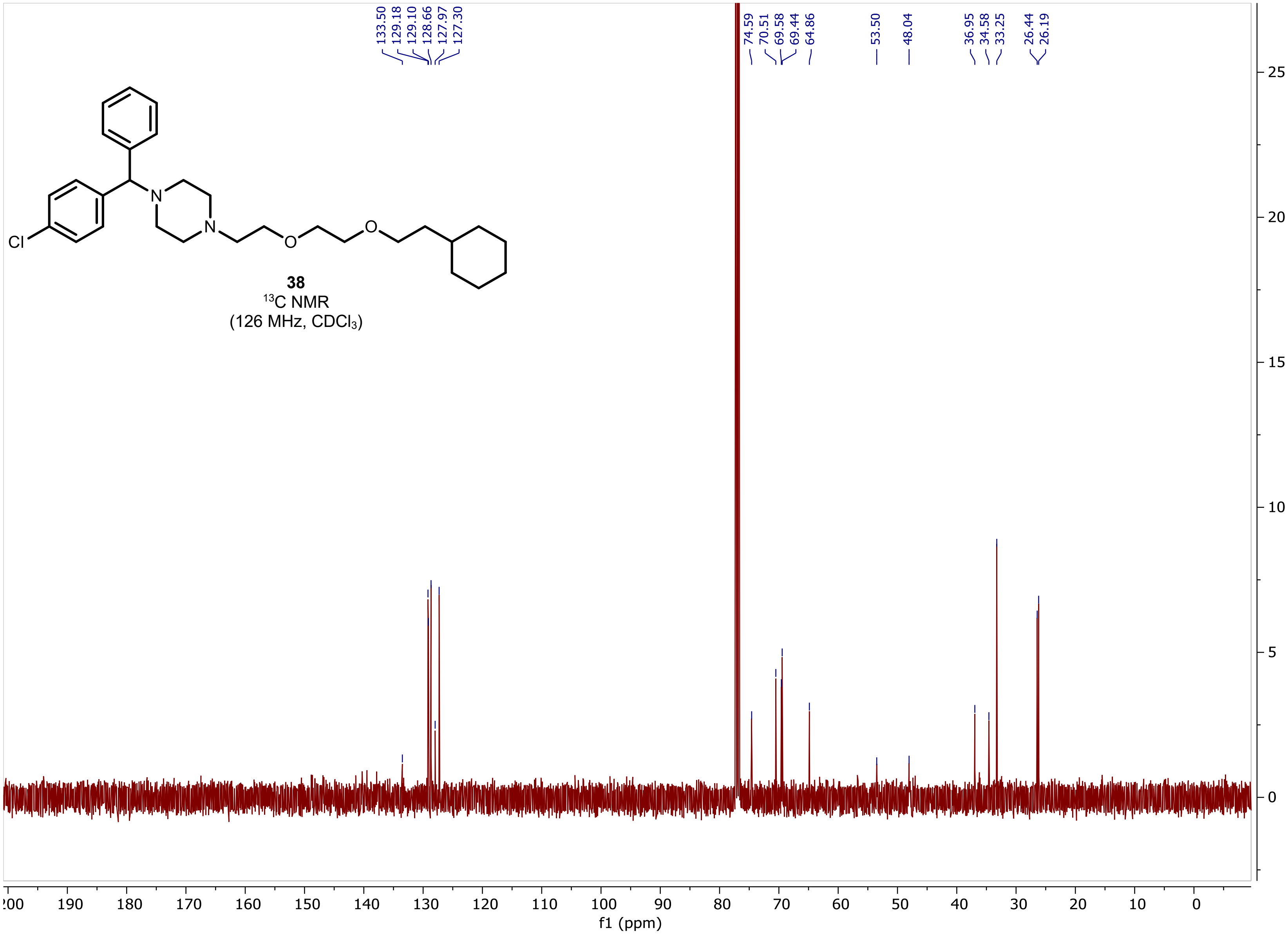
Cl
N
N
O
O
38
^{13}C NMR
(126 MHz, $CDCl_3$)
133.50
129.18
129.10
128.66
127.97
127.30
74.59
70.51
69.58
69.44
64.86
53.50
48.04
36.95
34.58
33.25
26.44
26.19
f1 (ppm)

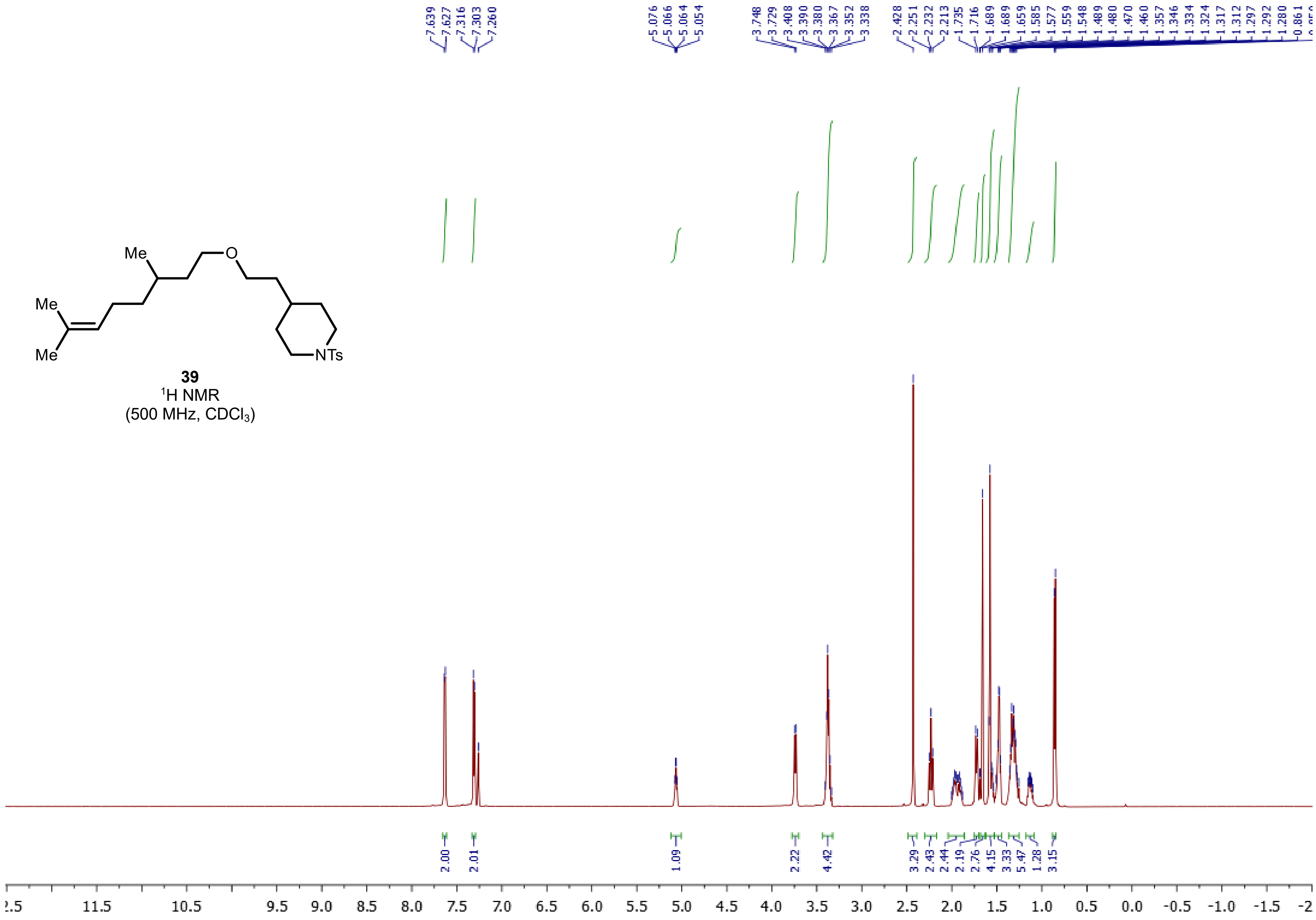
Me
Me
Me
O
NTs
39
1H NMR
(500 MHz, CDCl3)

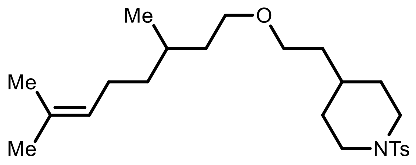

39
^{13}C NMR
(126 MHz, $CDCl_3$)

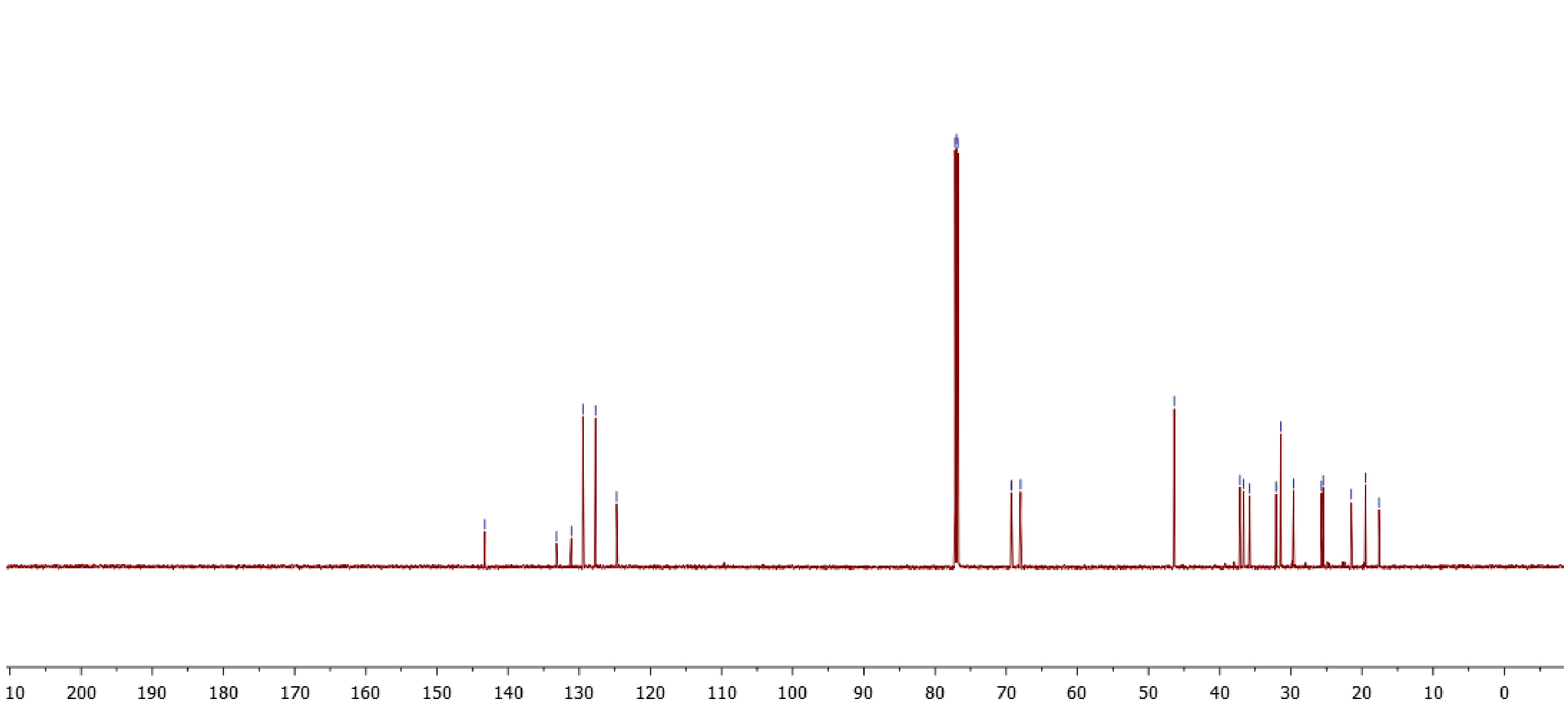

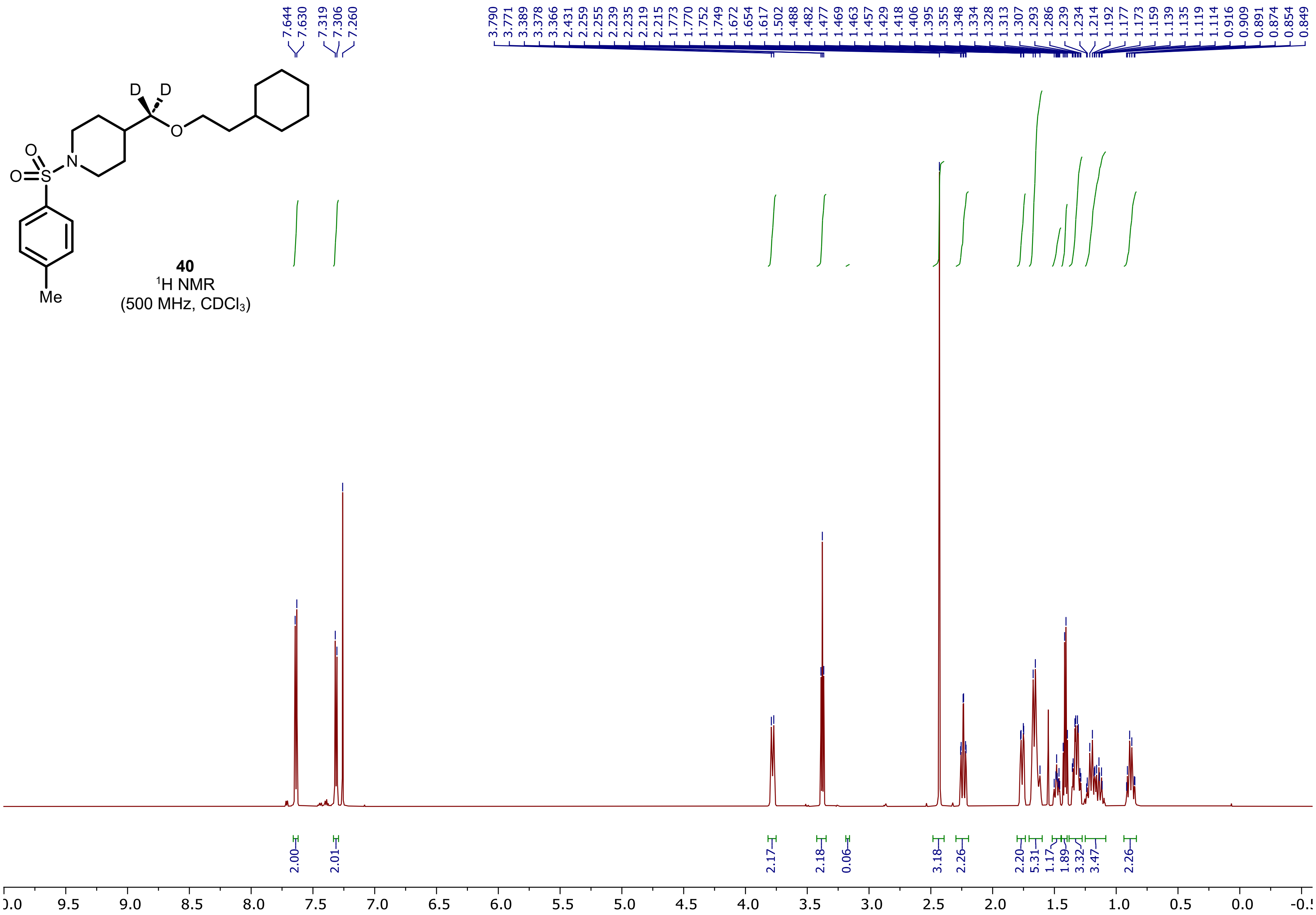
D
D
O
O
O
S
N
Me
40
^{1}H NMR
(500 MHz, $CDCl_3$)
7.644
7.630
7.319
7.306
7.260
3.790
3.771
3.389
3.378
3.366
2.431
2.259
2.255
2.239
2.235
2.219
2.215
1.773
1.770
1.752
1.749
1.672
1.654
1.617
1.502
1.488
1.482
1.477
1.469
1.463
1.457
1.429
1.418
1.406
1.395
1.355
1.348
1.334
1.328
1.313
1.307
1.293
1.286
1.239
1.234
1.214
1.192
1.177
1.173
1.159
1.139
1.135
1.119
1.114
0.916
0.909
0.891
0.874
0.854
0.849
2.00
2.01
2.17
2.18
0.06
3.18
2.26
2.20
5.31
1.17
1.89
3.32
3.47
2.26
9.5
9.0
8.5
8.0
7.5
7.0
6.5
6.0
5.5
5.0
4.5
4.0
3.5
3.0
2.5
2.0
1.5
1.0
0.5
0.0

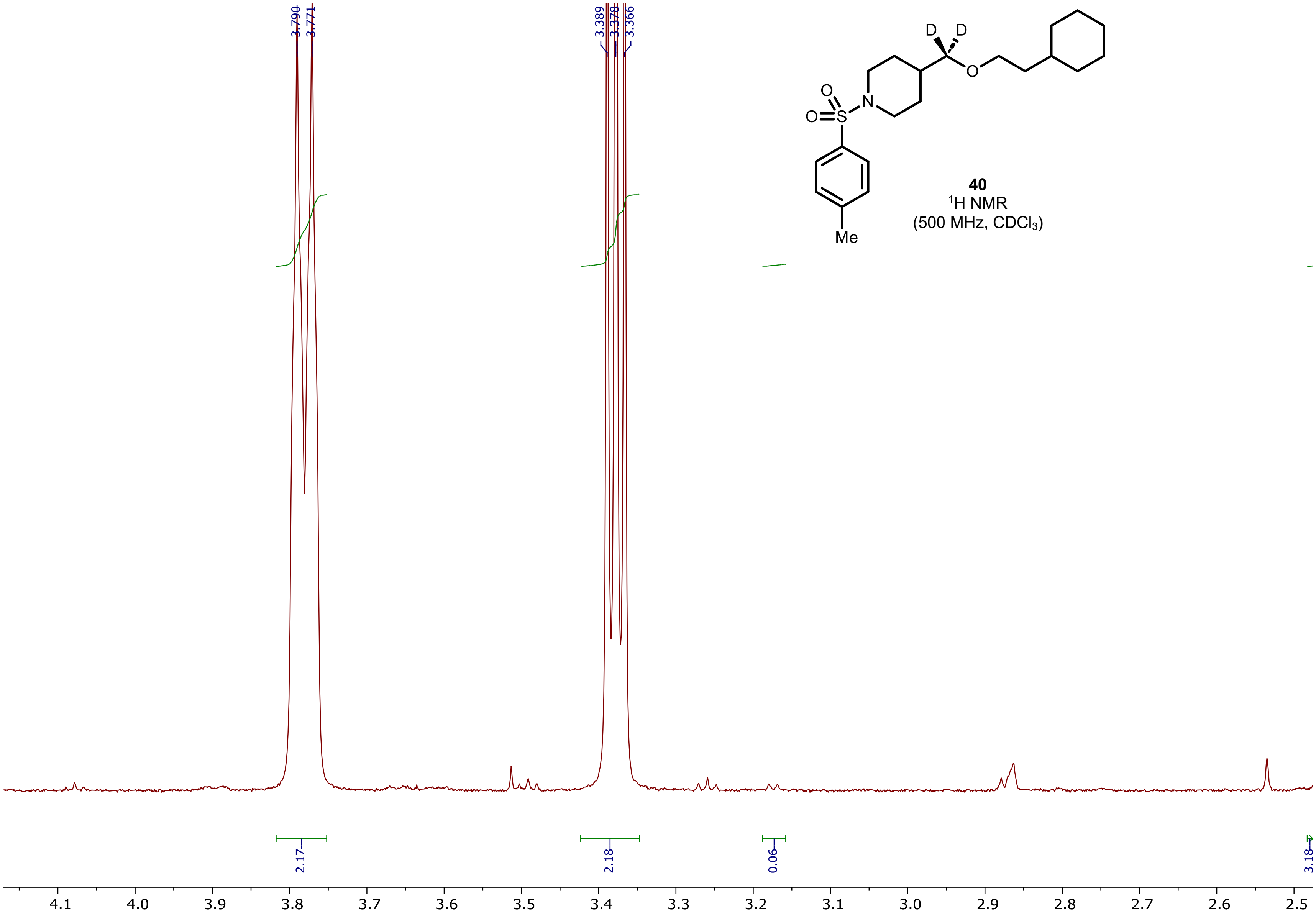
3.790
3.771
3.389
3.378
3.366
D
D
O
N
O
S
O
Me
40
¹H NMR
(500 MHz, CDCl₃)
2.17
2.18
0.06
3.18
4.1
4.0
3.9
3.8
3.7
3.6
3.5
3.4
3.3
3.2
3.1
3.0
2.9
2.8
2.7
2.6
2.5

Zoomed in ^{1}H NMR of **3.82** where CD_2 would be. The H's at 3.17 integrate to ~3% of what they would if it were full H incorporation.

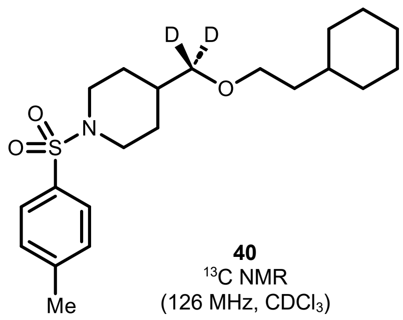

40

^{13}C NMR

(126 MHz, $CDCl_3$)

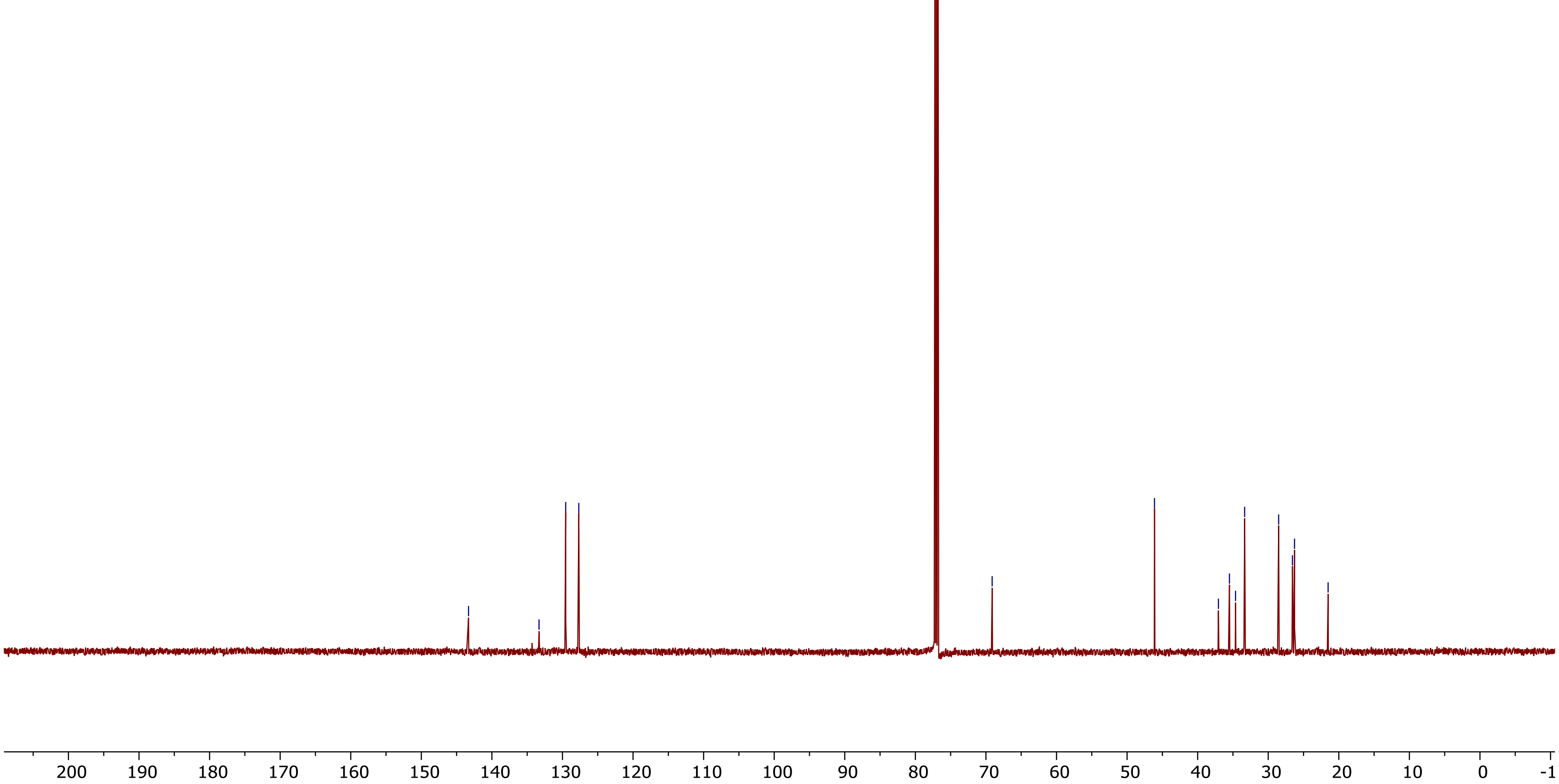

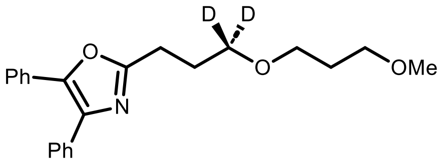

41
^{1}H NMR
(500 MHz, $CDCl_3$)

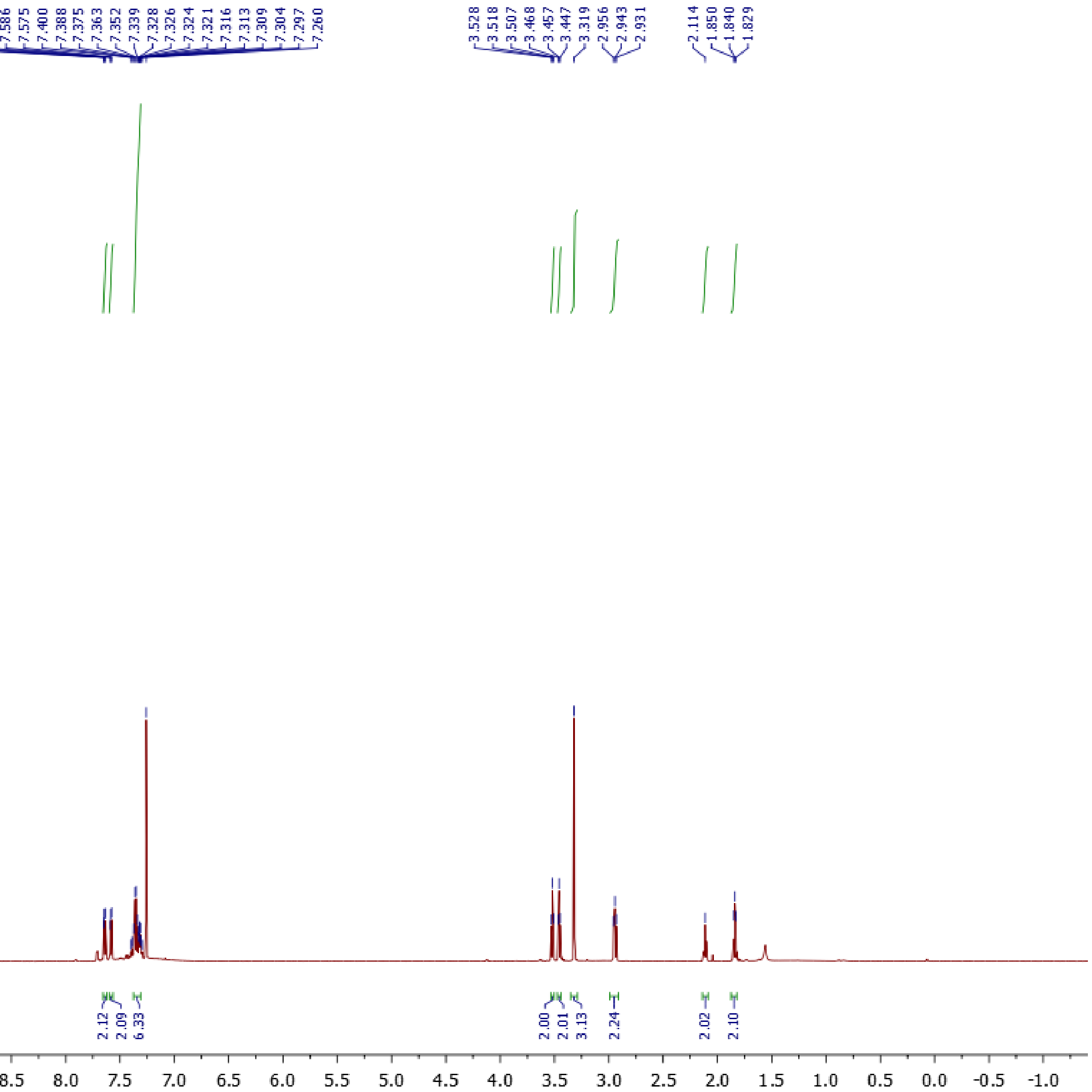

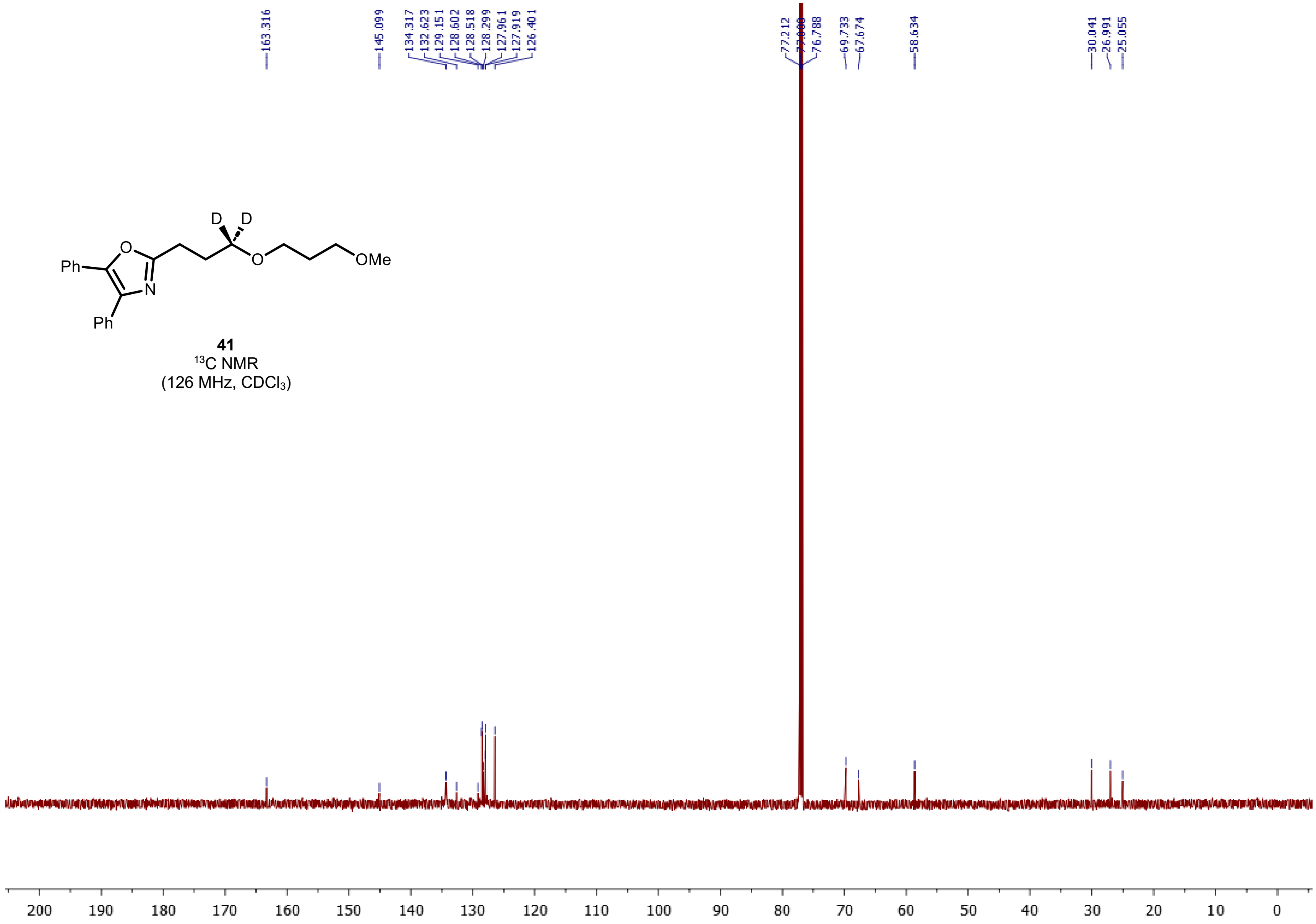

41
^{13}C NMR
(126 MHz, $CDCl_3$)

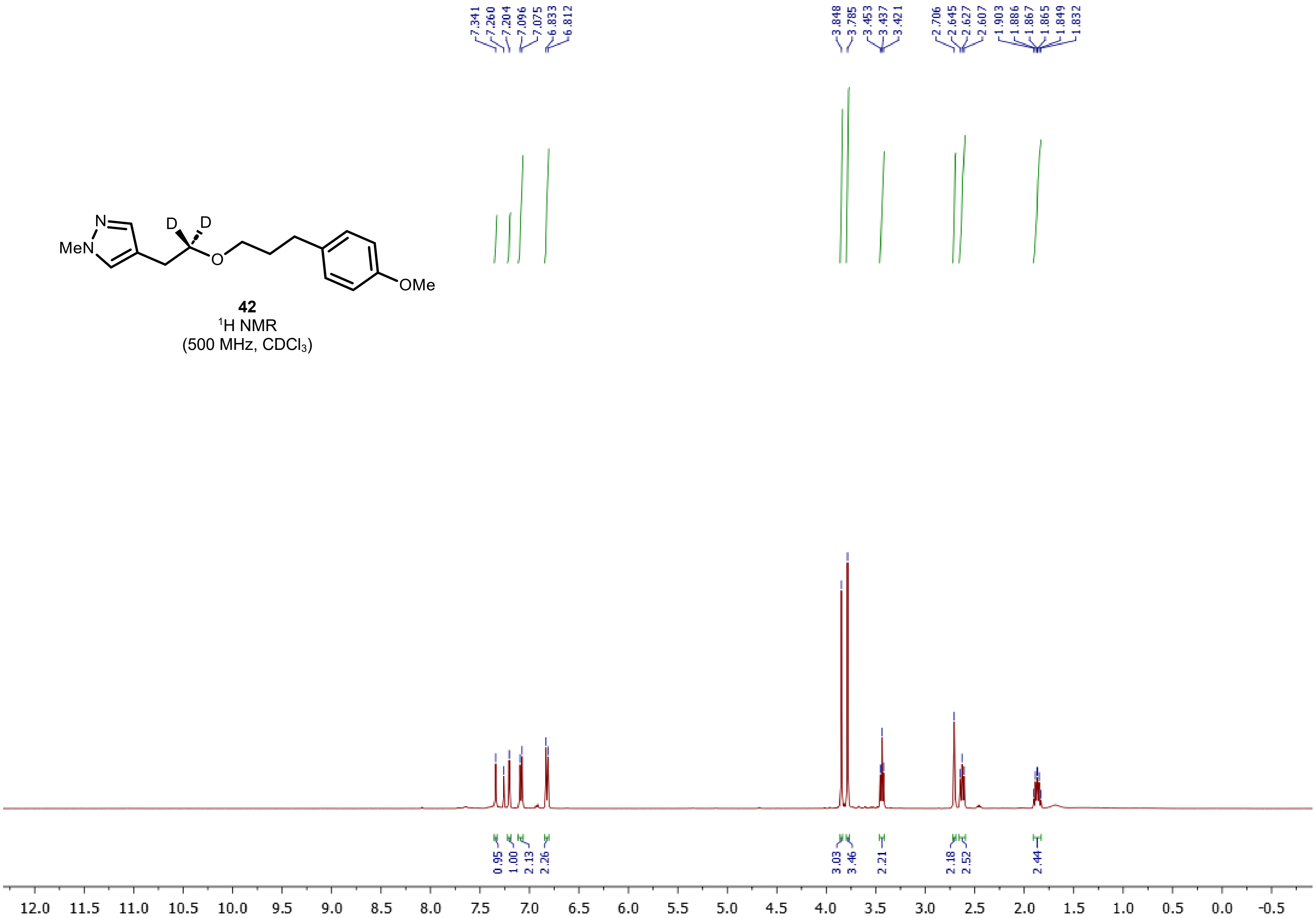

42
^{1}H NMR
(500 MHz, $CDCl_3$)

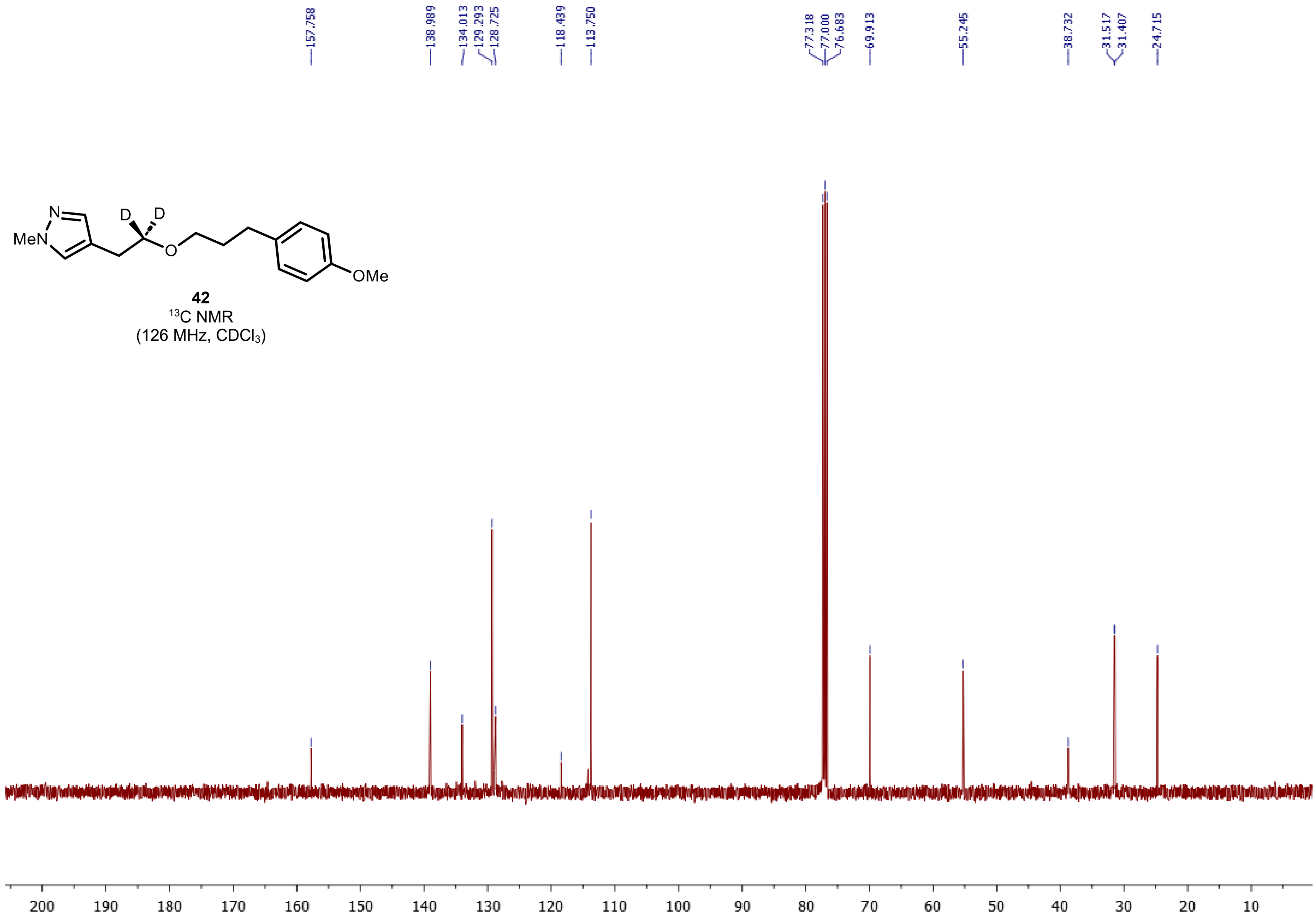

42
^{13}C NMR
(126 MHz, $CDCl_3$)

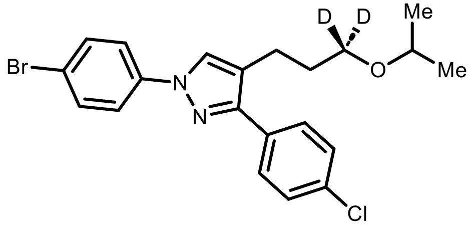

43
^{1}H NMR
(500 MHz, $CDCl_3$)

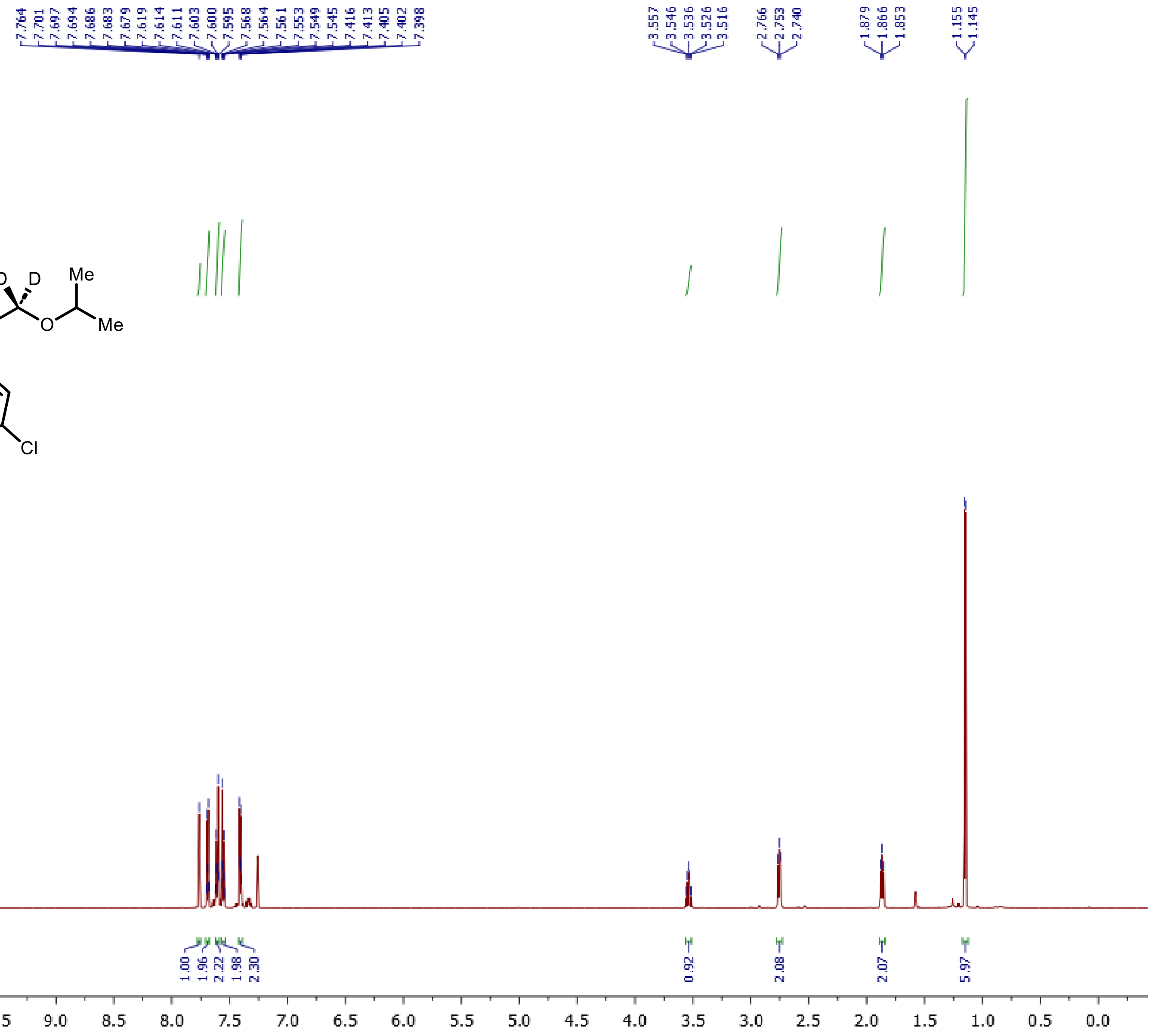

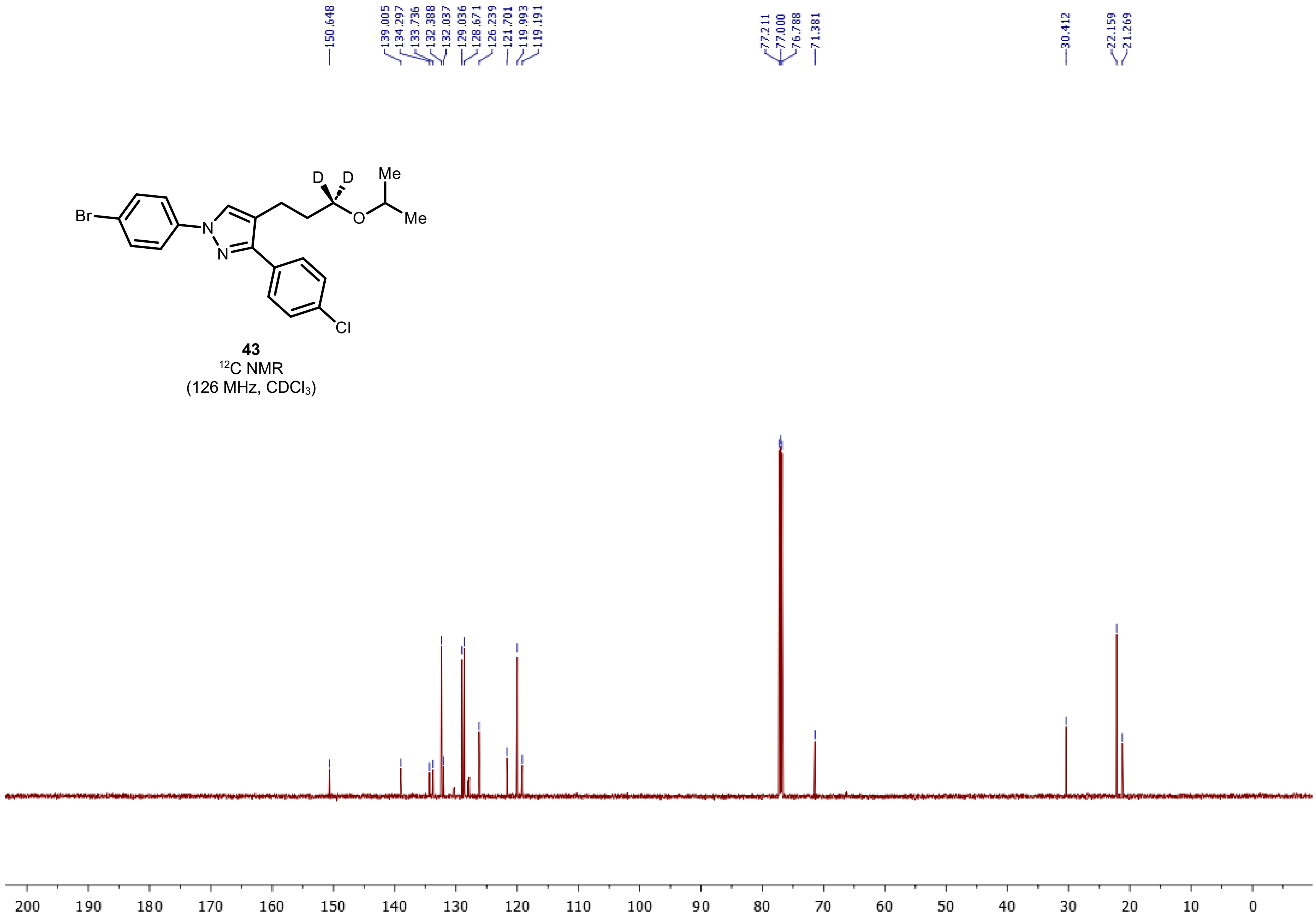

43
^{12}C NMR
(126 MHz, $CDCl_3$)

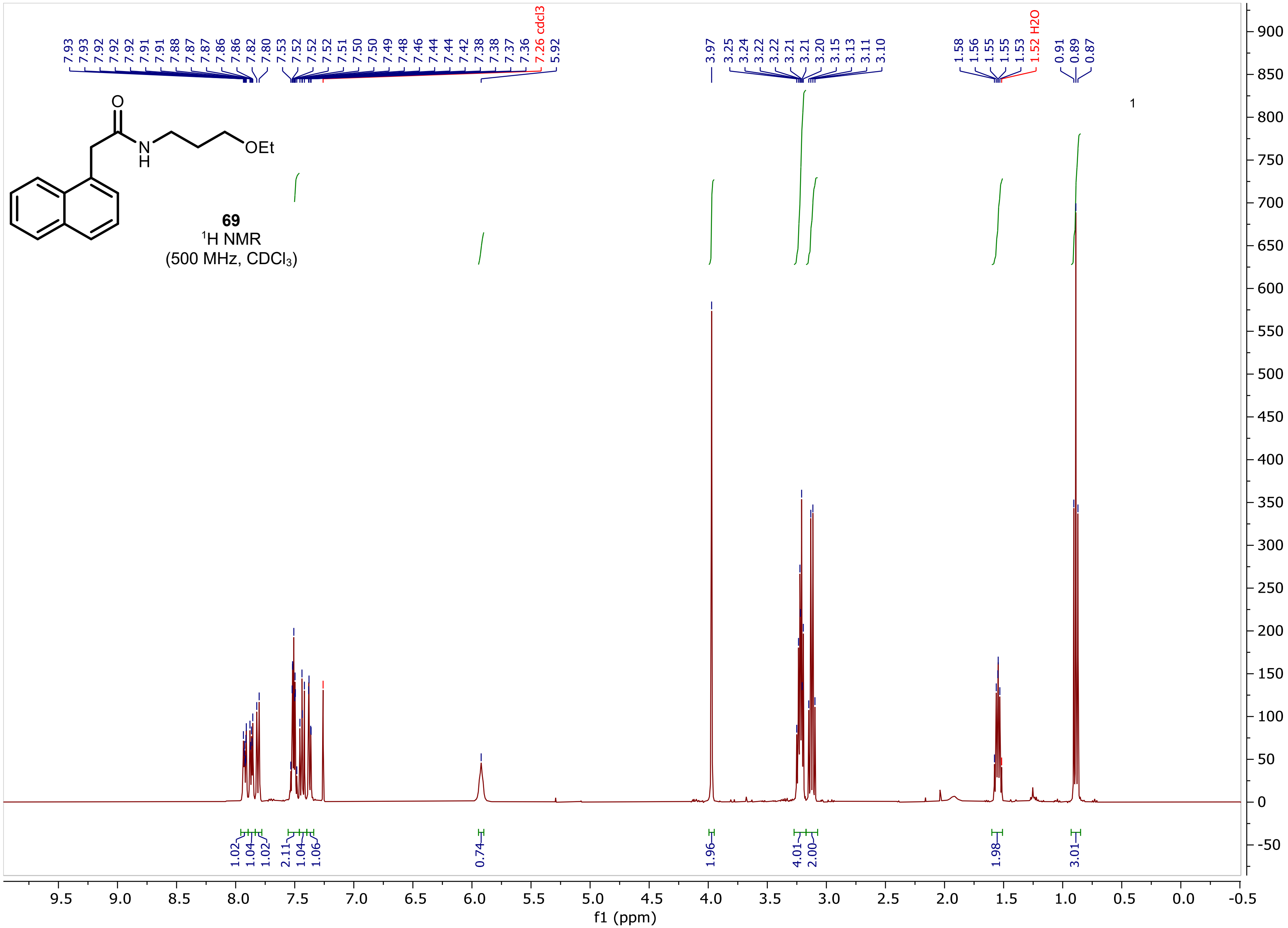
7.93 7.93 7.92 7.92 7.92 7.91 7.91 7.88 7.87 7.87 7.86 7.86 7.82 7.80 7.53 7.52 7.52 7.52 7.51 7.50 7.50 7.49 7.48 7.46 7.44 7.44 7.42 7.38 7.38 7.37 7.36 7.26 cdcl3 5.92
3.97 3.25 3.24 3.22 3.22 3.21 3.21 3.20 3.15 3.13 3.11 3.10
1.58 1.56 1.55 1.55 1.53 1.52 H2O 0.91 0.89 0.87
O
N
H
OEt
69
1H NMR
(500 MHz, CDCl3)
1.02 1.04 1.02 2.11 1.04 1.06 0.74 1.96 4.01 2.00 1.98 3.01
f1 (ppm)

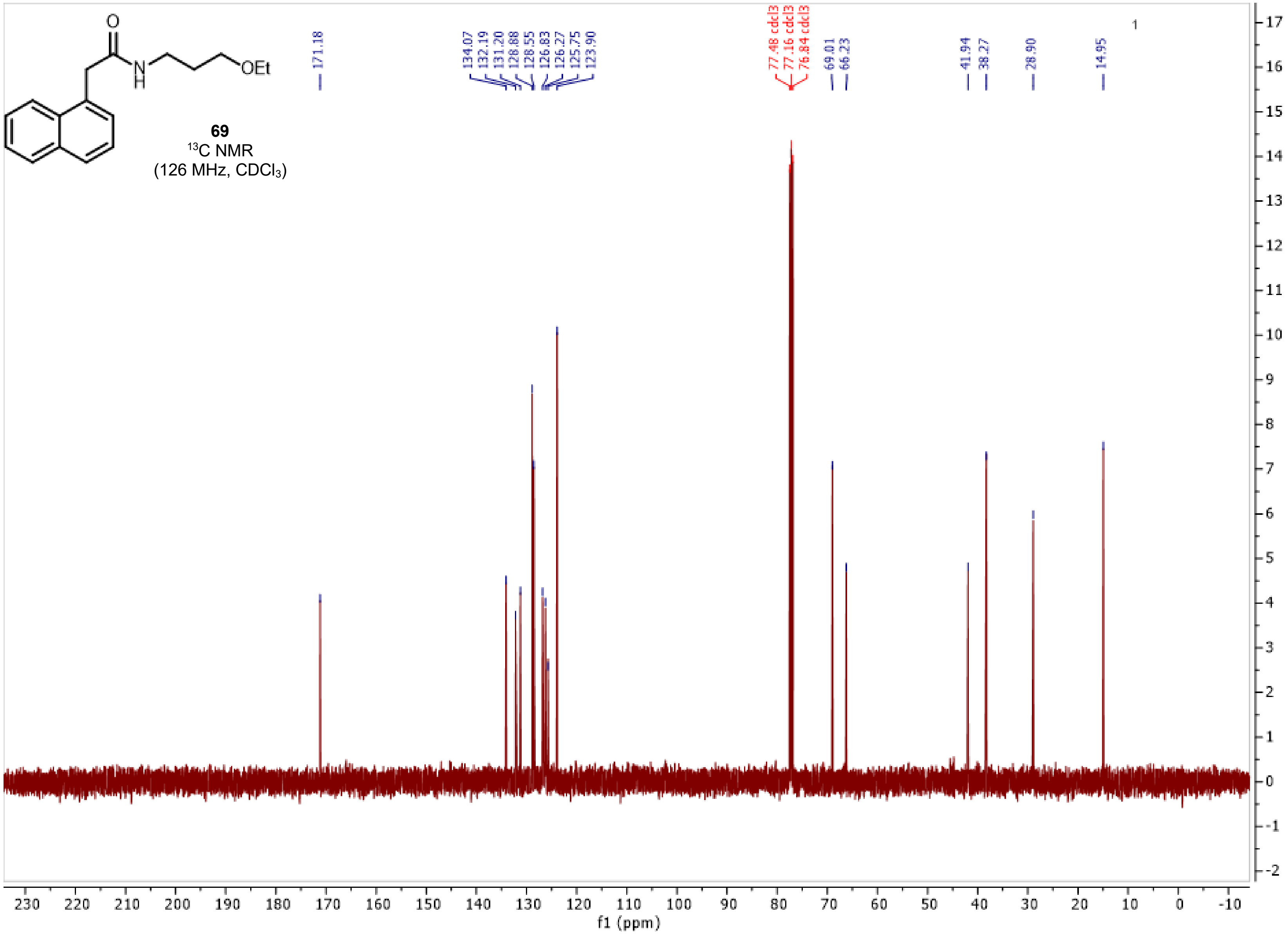
69
13C NMR
(126 MHz, CDCl3)
171.18
134.07
132.19
131.20
128.88
128.55
126.83
126.27
125.75
123.90
77.48 cdcl3
77.16 cdcl3
76.84 cdcl3
69.01
66.23
41.94
38.27
28.90
14.95
f1 (ppm)

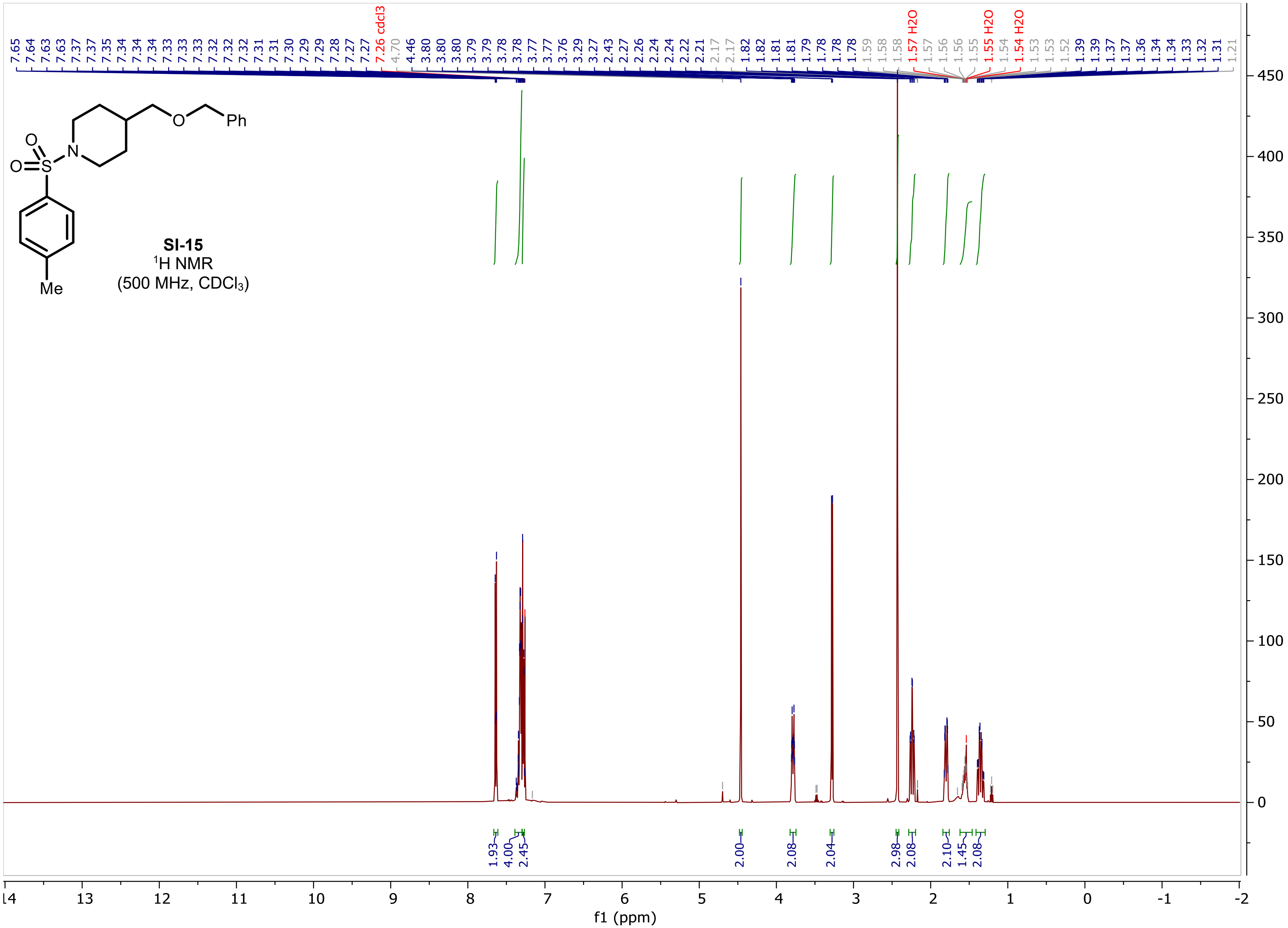
SI-15
1H NMR
(500 MHz, CDCl3)
f1 (ppm)

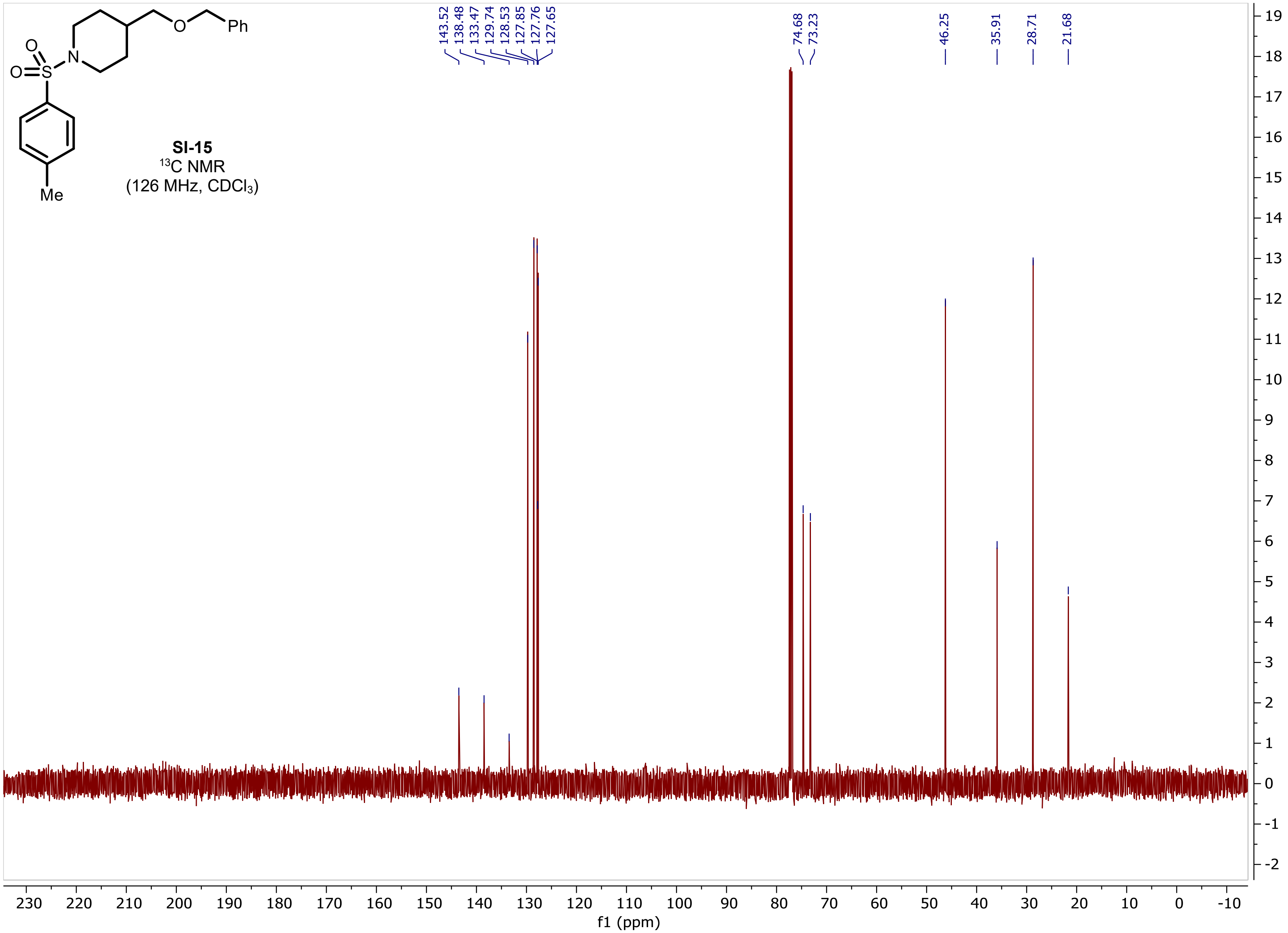
SI-15
^{13}C NMR
(126 MHz, $CDCl_3$)
143.52
138.48
133.47
129.74
128.53
127.85
127.76
127.65
74.68
73.23
46.25
35.91
28.71
21.68
f1 (ppm)

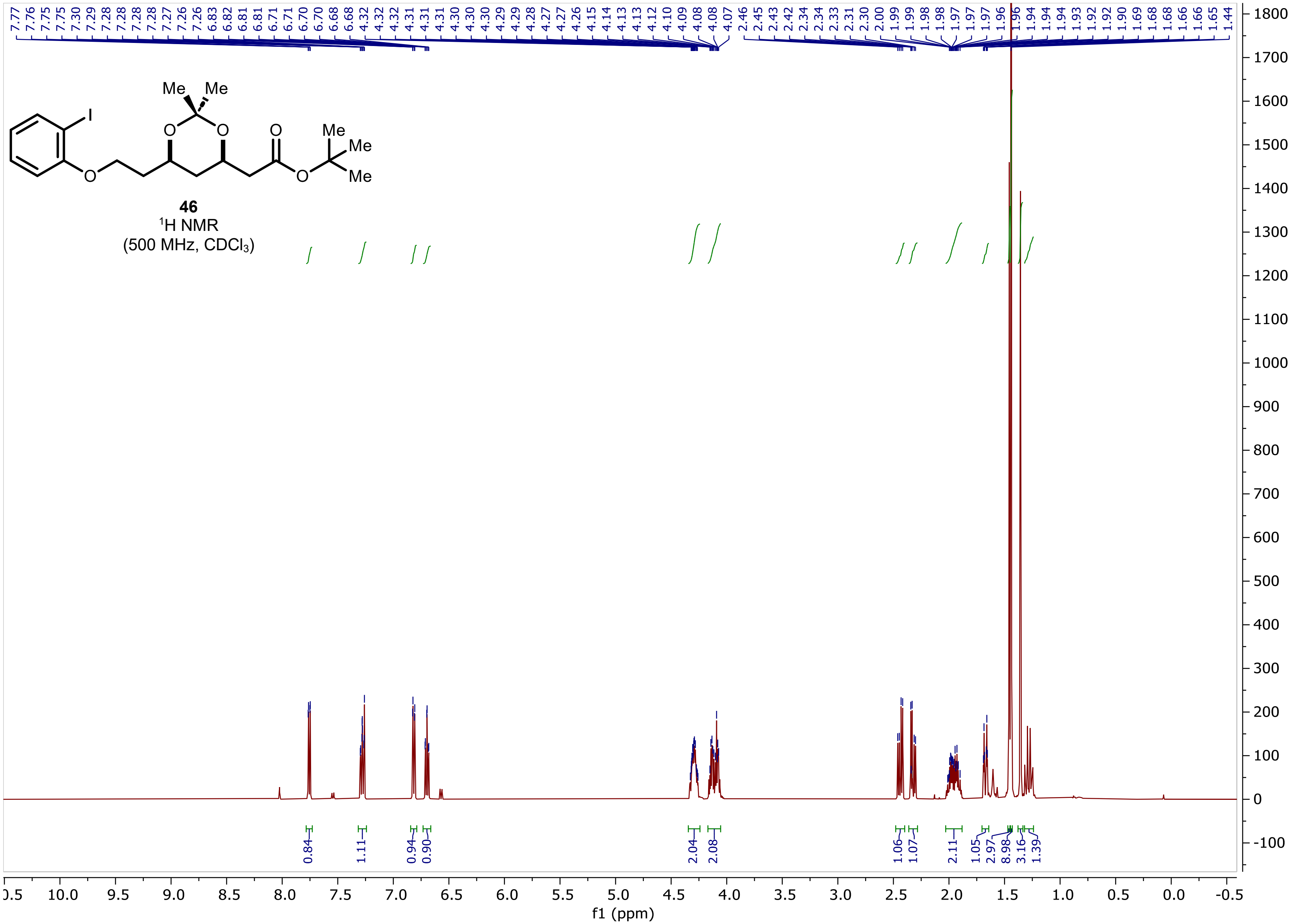
46
1H NMR
(500 MHz, CDCl3)
f1 (ppm)

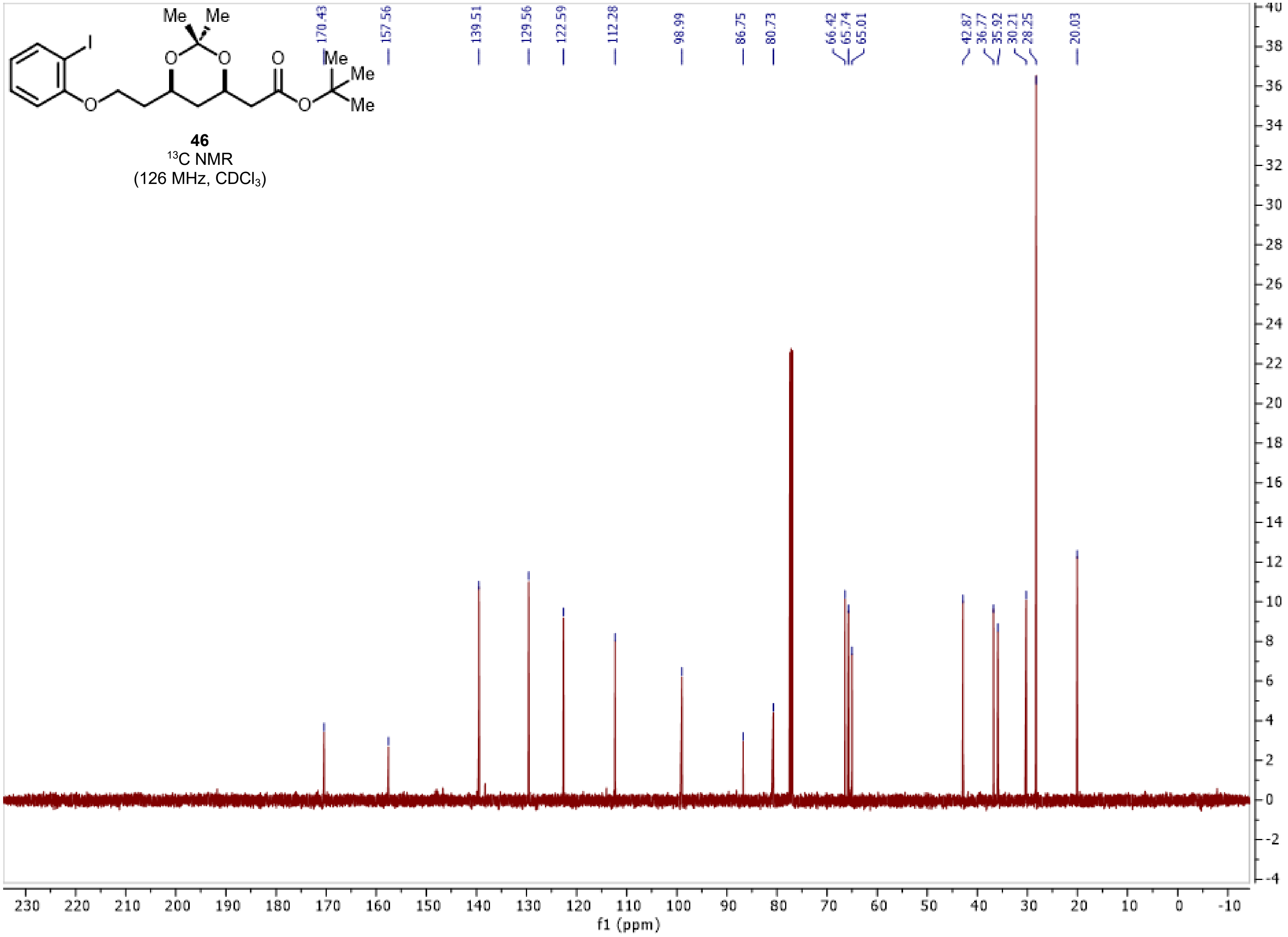
46
13C NMR
(126 MHz, CDCl3)
170.43
157.56
139.51
129.56
122.59
112.28
98.99
86.75
80.73
66.42
65.74
65.01
42.87
36.77
35.92
30.21
28.25
20.03
f1 (ppm)

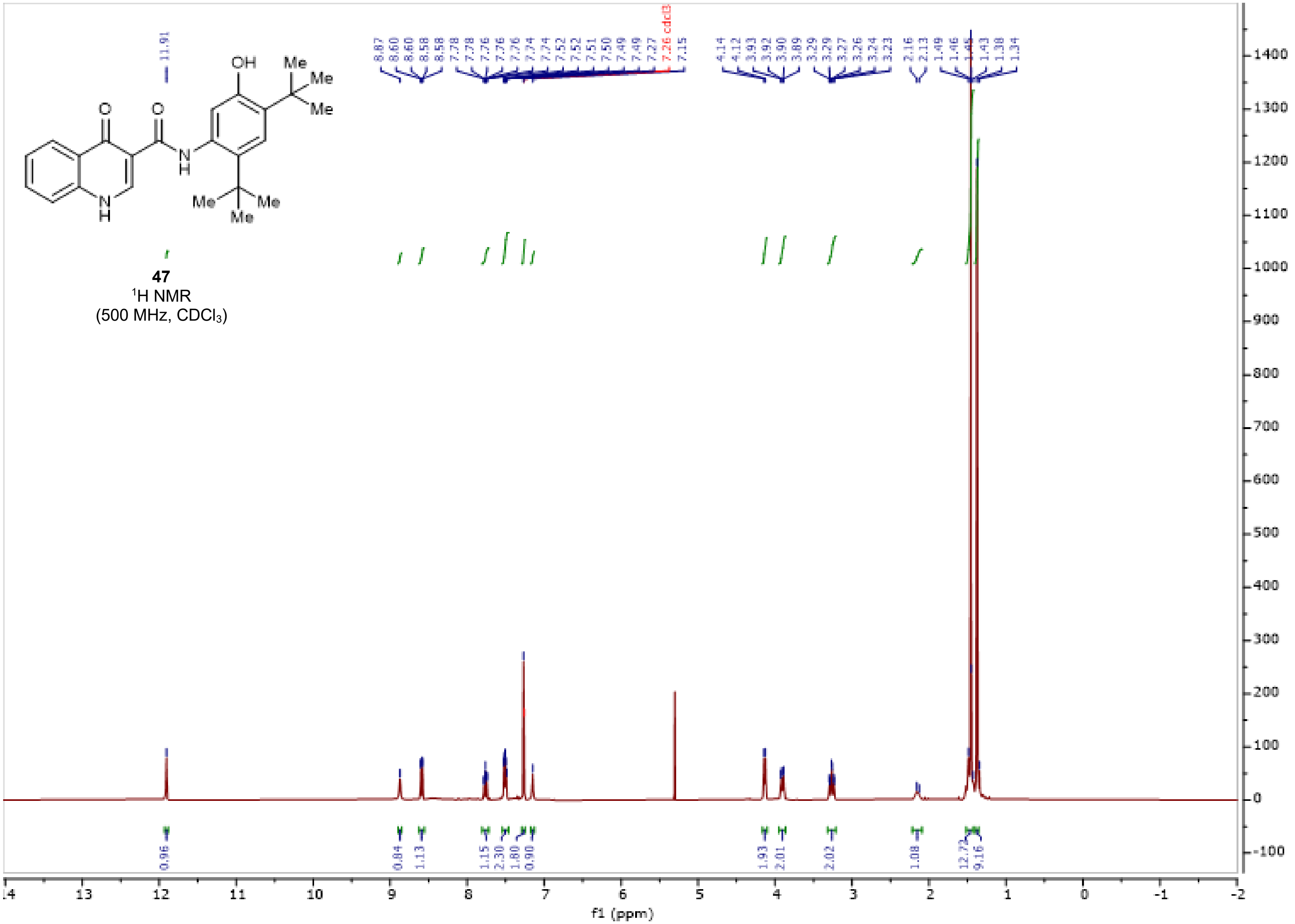

47
^{1}H NMR
(500 MHz, $CDCl_3$)

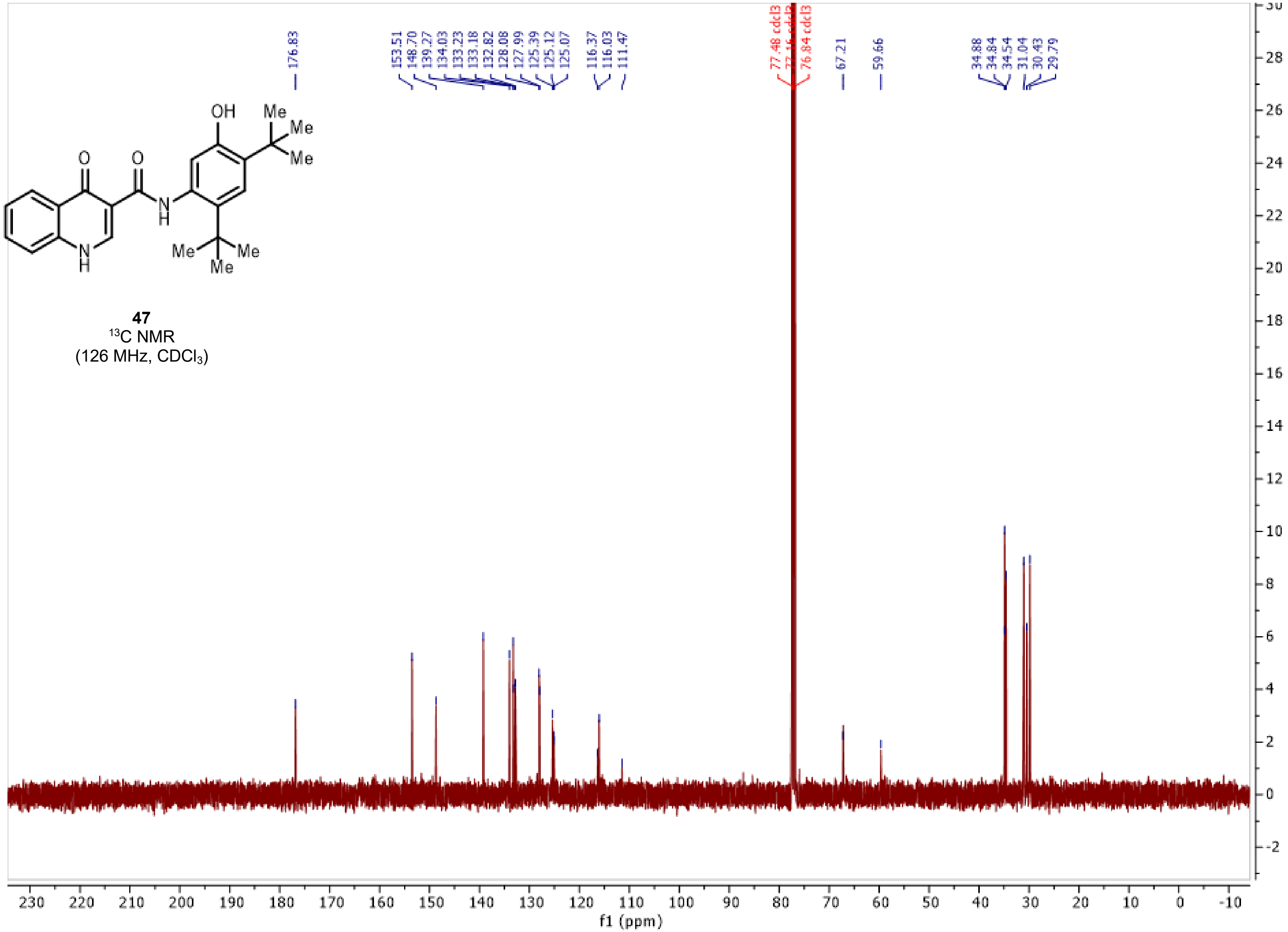

47
^{13}C NMR
(126 MHz, $CDCl_3$)

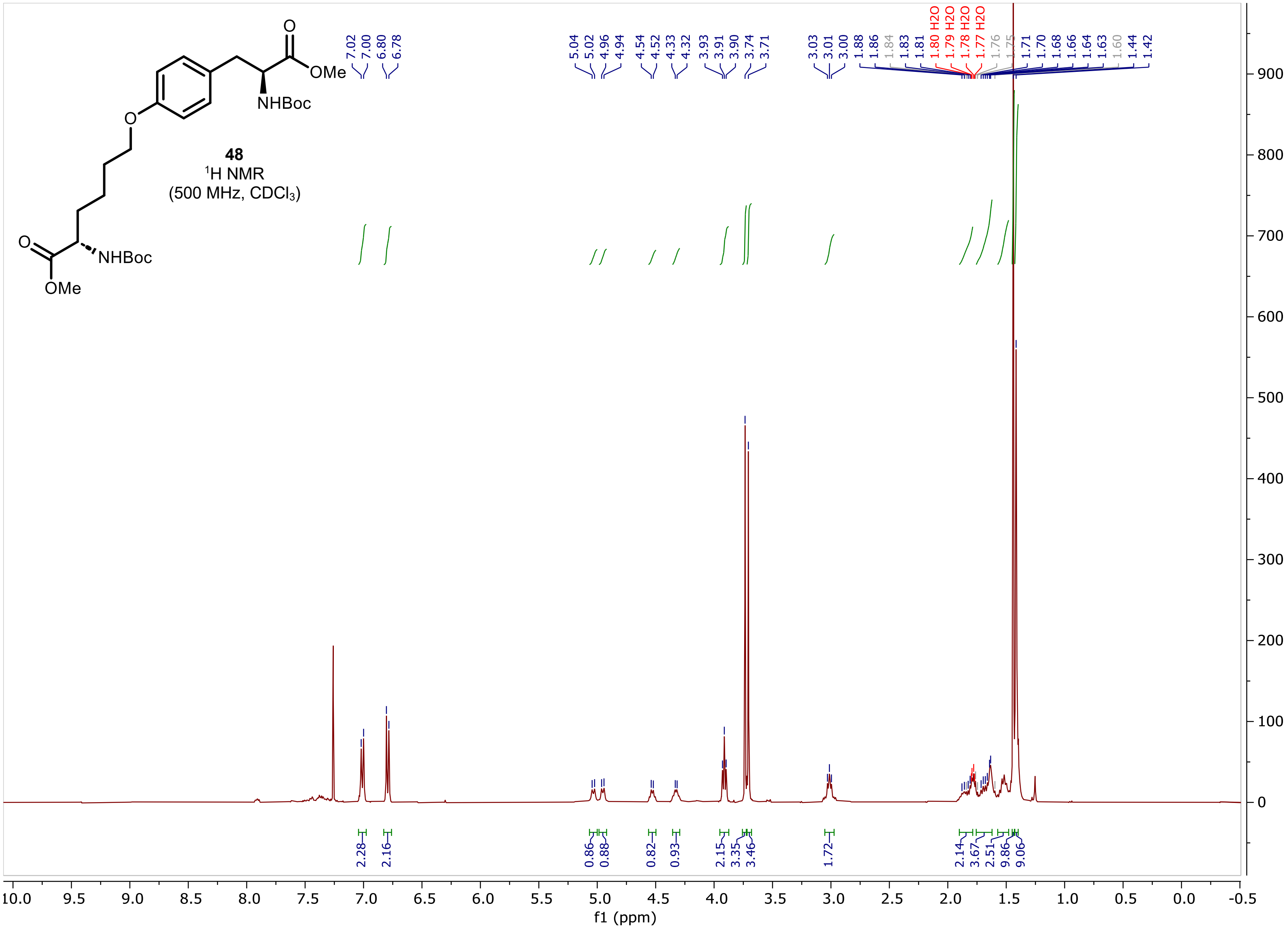
48
1H NMR
(500 MHz, CDCl3)
NHBoc
OMe
O
f1 (ppm)

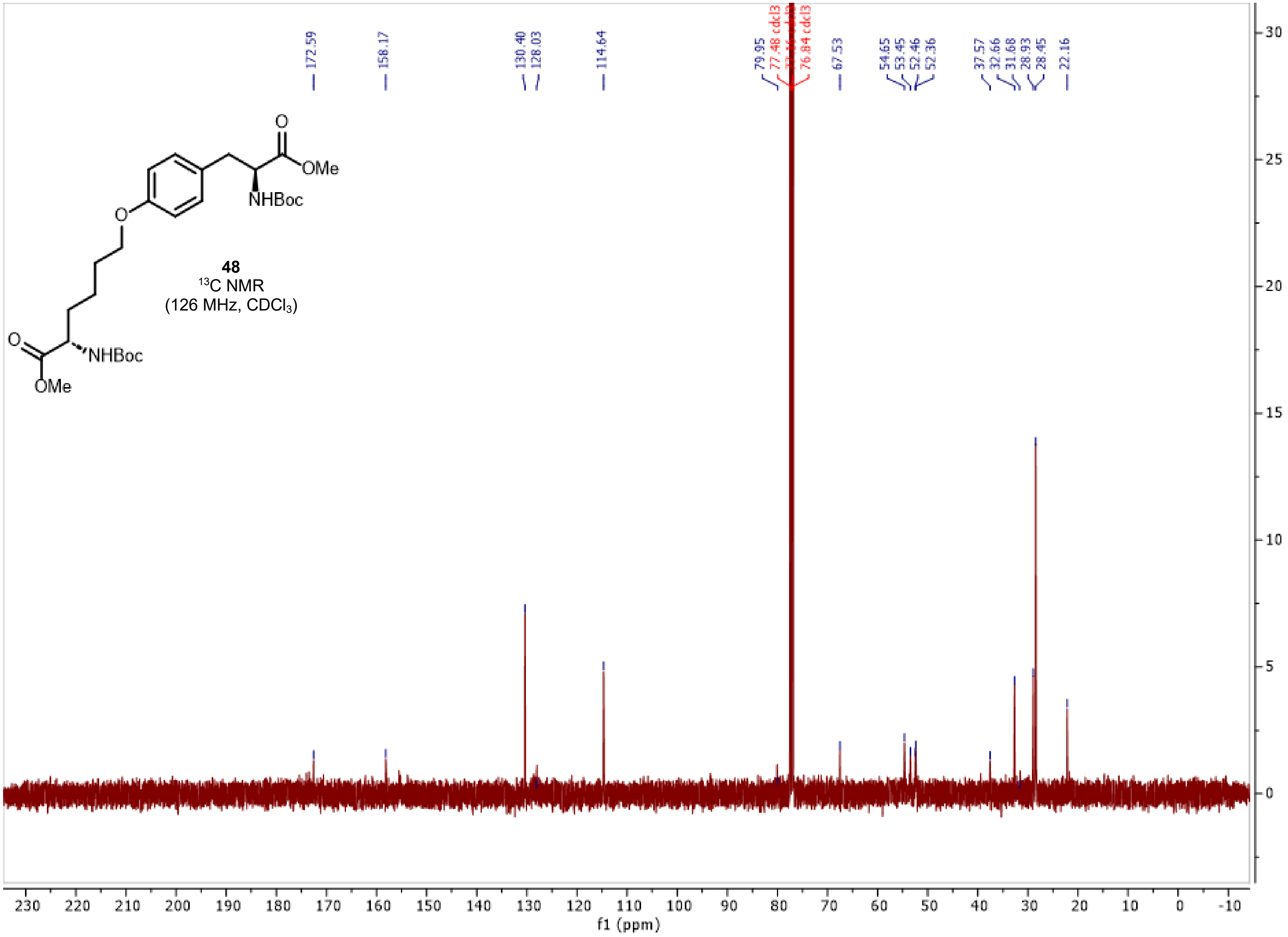
172.59
158.17
130.40
128.03
114.64
79.95
77.48 cdcl3
76.84 cdcl3
67.53
54.65
53.45
52.46
52.36
37.57
32.66
31.68
28.93
28.45
22.16
O
OMe
NHBoc
O
O
NHBoc
OMe
48
^{13}C NMR
(126 MHz, $CDCl_3$)
f1 (ppm)

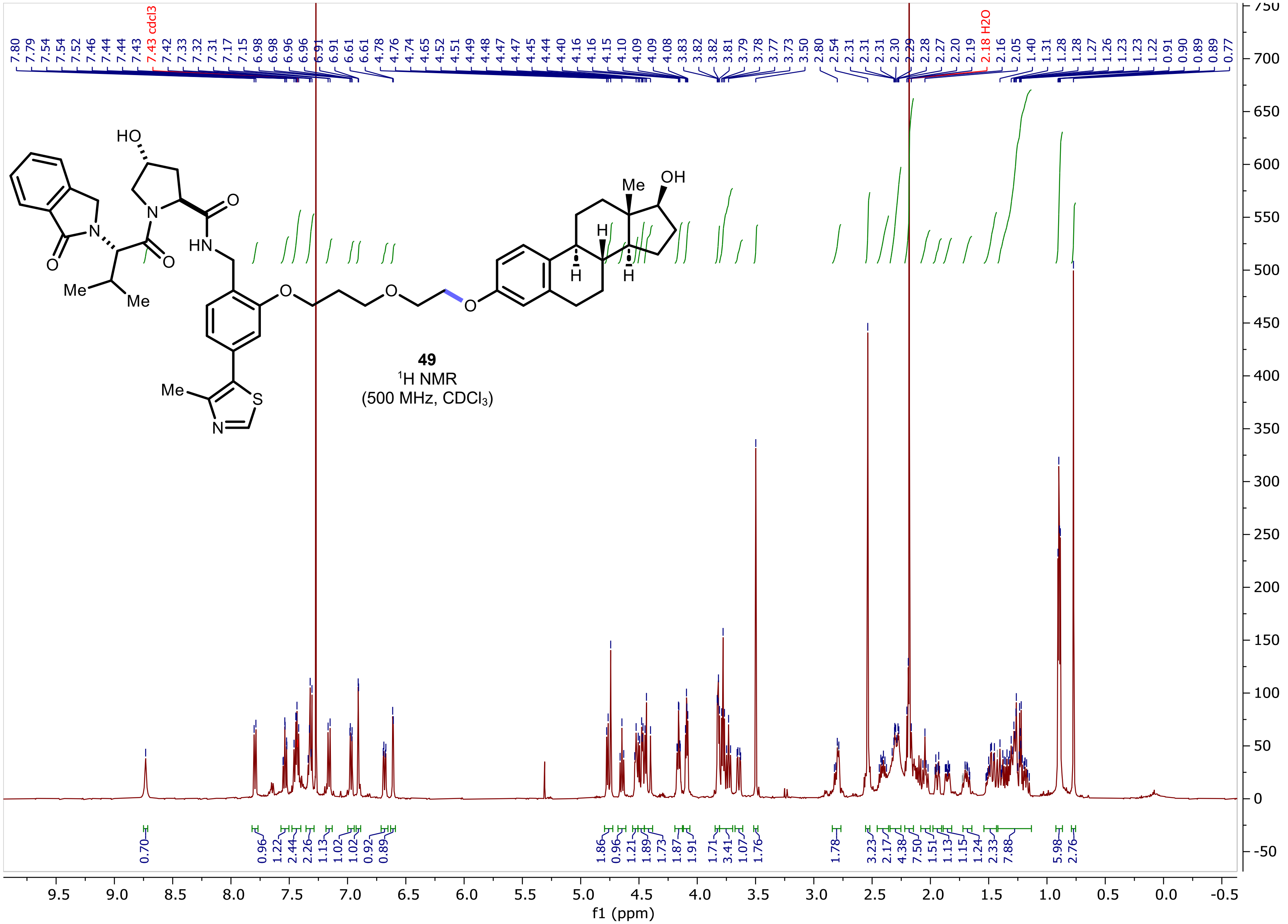
49
1H NMR
(500 MHz, CDCl3)
f1 (ppm)

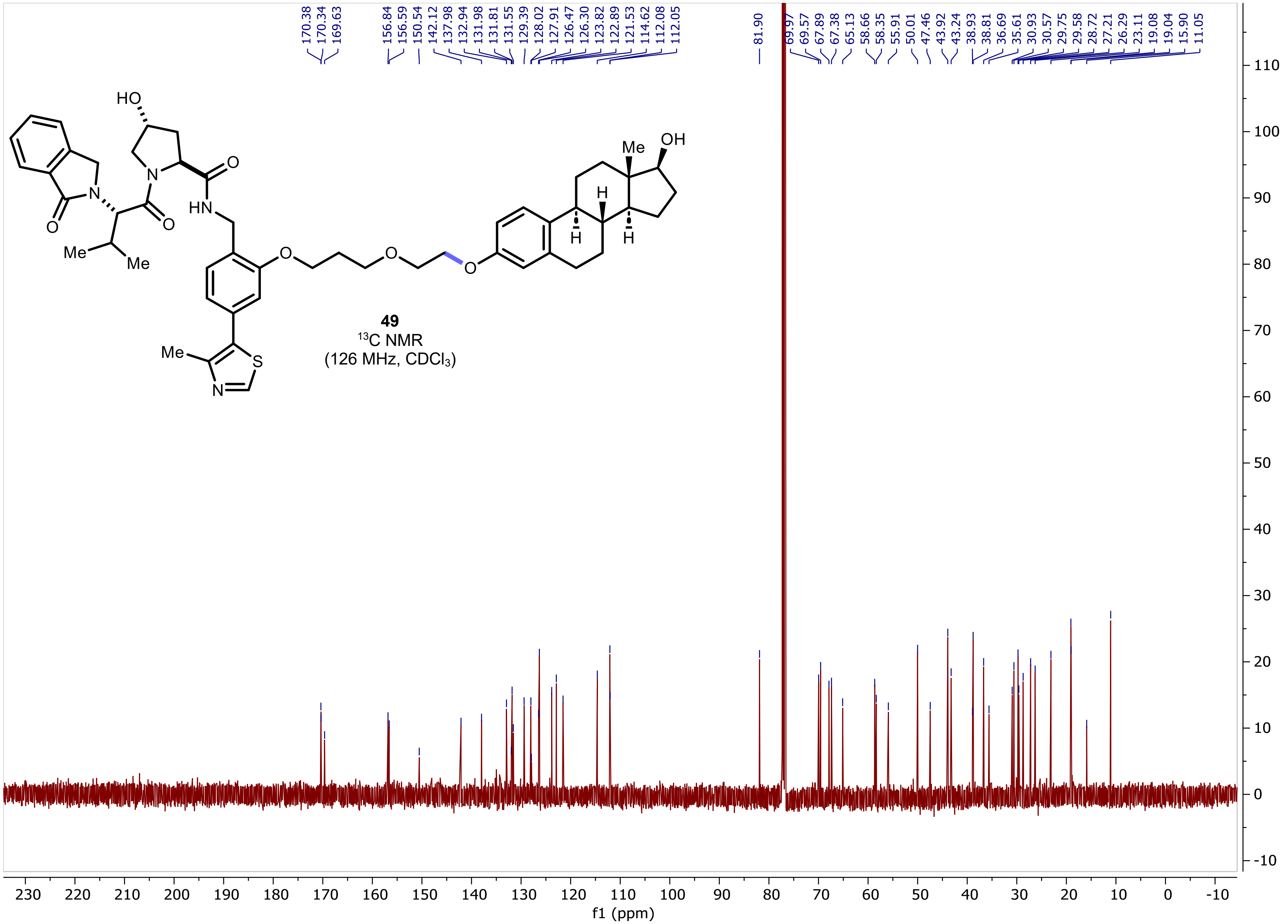
170.38
170.34
169.63
156.84
156.59
150.54
142.12
137.98
132.94
131.98
131.81
131.55
129.39
128.02
127.91
126.47
126.30
123.82
122.89
121.53
114.62
112.08
112.05
81.90
69.97
69.57
67.89
67.38
65.13
58.66
58.35
55.91
50.01
47.46
43.92
43.24
38.93
38.81
36.69
35.61
30.93
30.57
29.75
29.58
28.72
27.21
26.29
23.11
19.08
19.04
15.90
11.05
49
^{13}C NMR
(126 MHz, $CDCl_3$)
f1 (ppm)

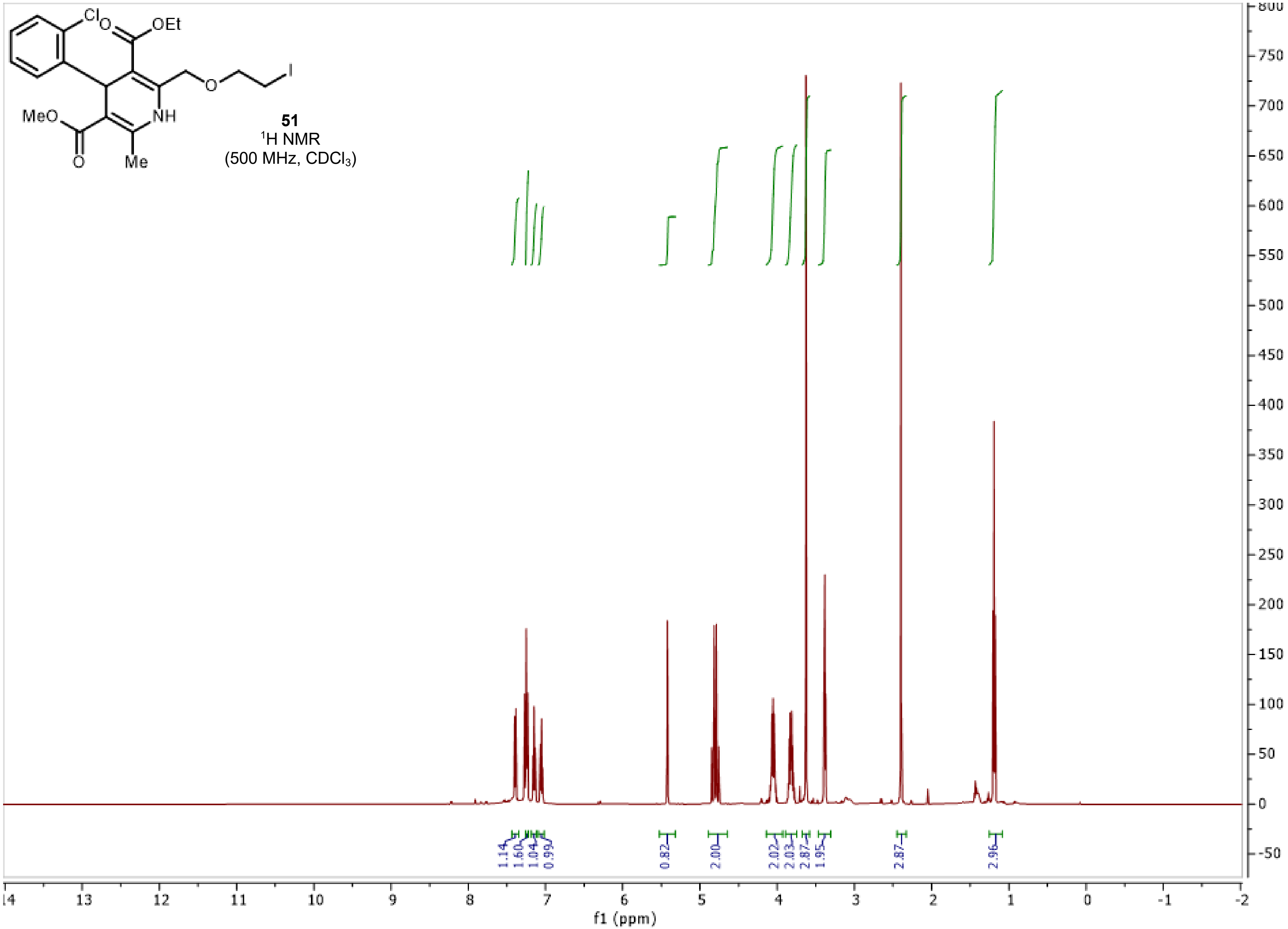

Cl
O
OEt
O
I
MeO
NH
O
Me
51
1H NMR
(500 MHz, CDCl3)
1.14
1.60
1.04
0.99
0.82
2.00
2.02
2.03
2.87
1.95
2.87
2.96
f1 (ppm)

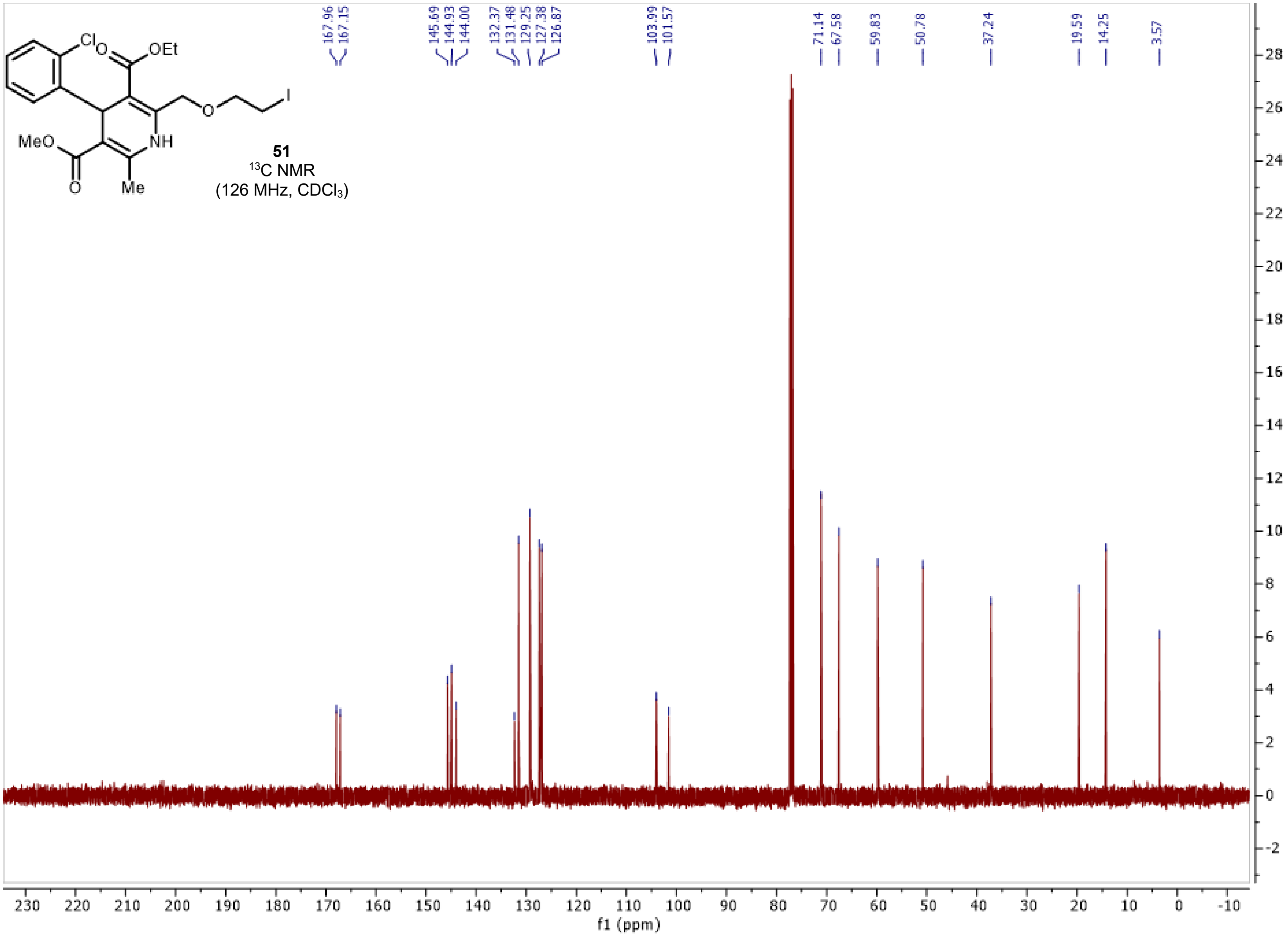

Cl
O
OEt
O
I
MeO
NH
O
Me
51
^{13}C NMR
(126 MHz, $CDCl_3$)
167.96
167.15
145.69
144.93
144.00
132.37
131.48
129.25
127.38
126.87
103.99
101.57
71.14
67.58
59.83
50.78
37.24
19.59
14.25
3.57
f1 (ppm)

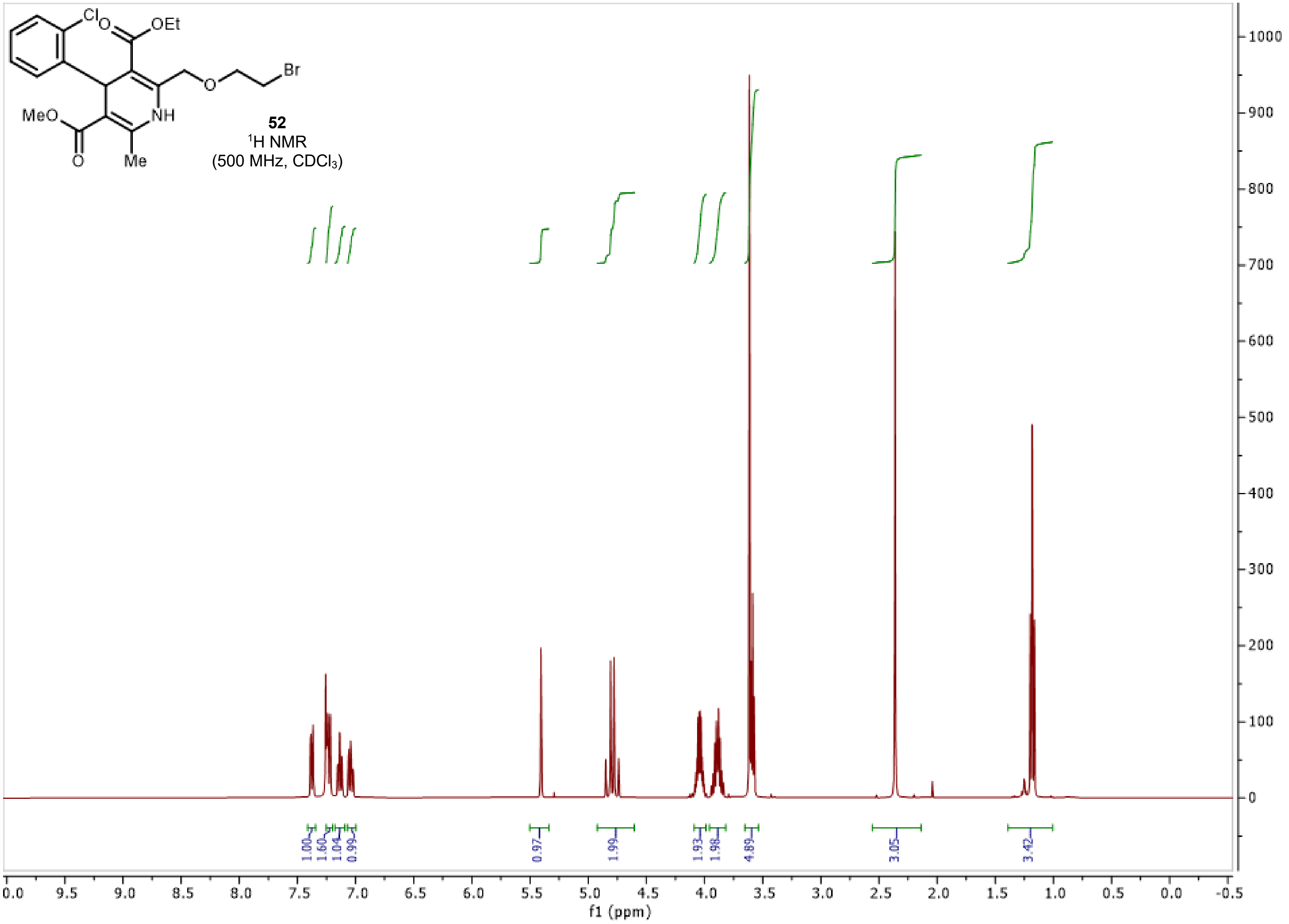

Cl
O
OEt
Br
O
NH
MeO
O
Me
52
^{1}H NMR
(500 MHz, CDCl$_3$)
1.00
1.60
1.04
0.99
0.97
1.99
1.93
1.98
4.89
3.05
3.42
f1 (ppm)

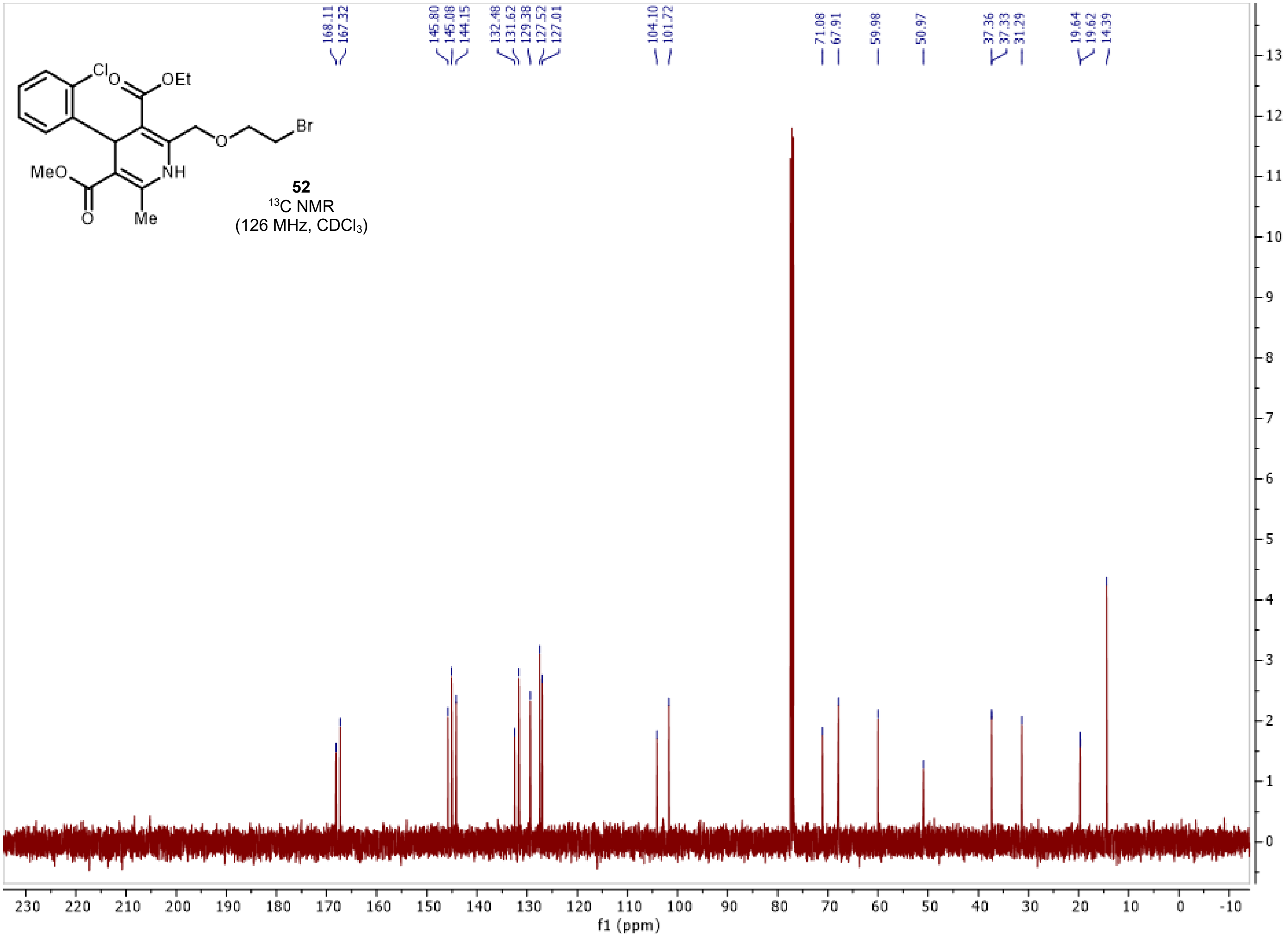

52
^{13}C NMR
(126 MHz, $CDCl_3$)
168.11
167.32
145.80
145.08
144.15
132.48
131.62
129.38
127.52
127.01
104.10
101.72
71.08
67.91
59.98
50.97
37.36
37.33
31.29
19.64
19.62
14.39
f1 (ppm)

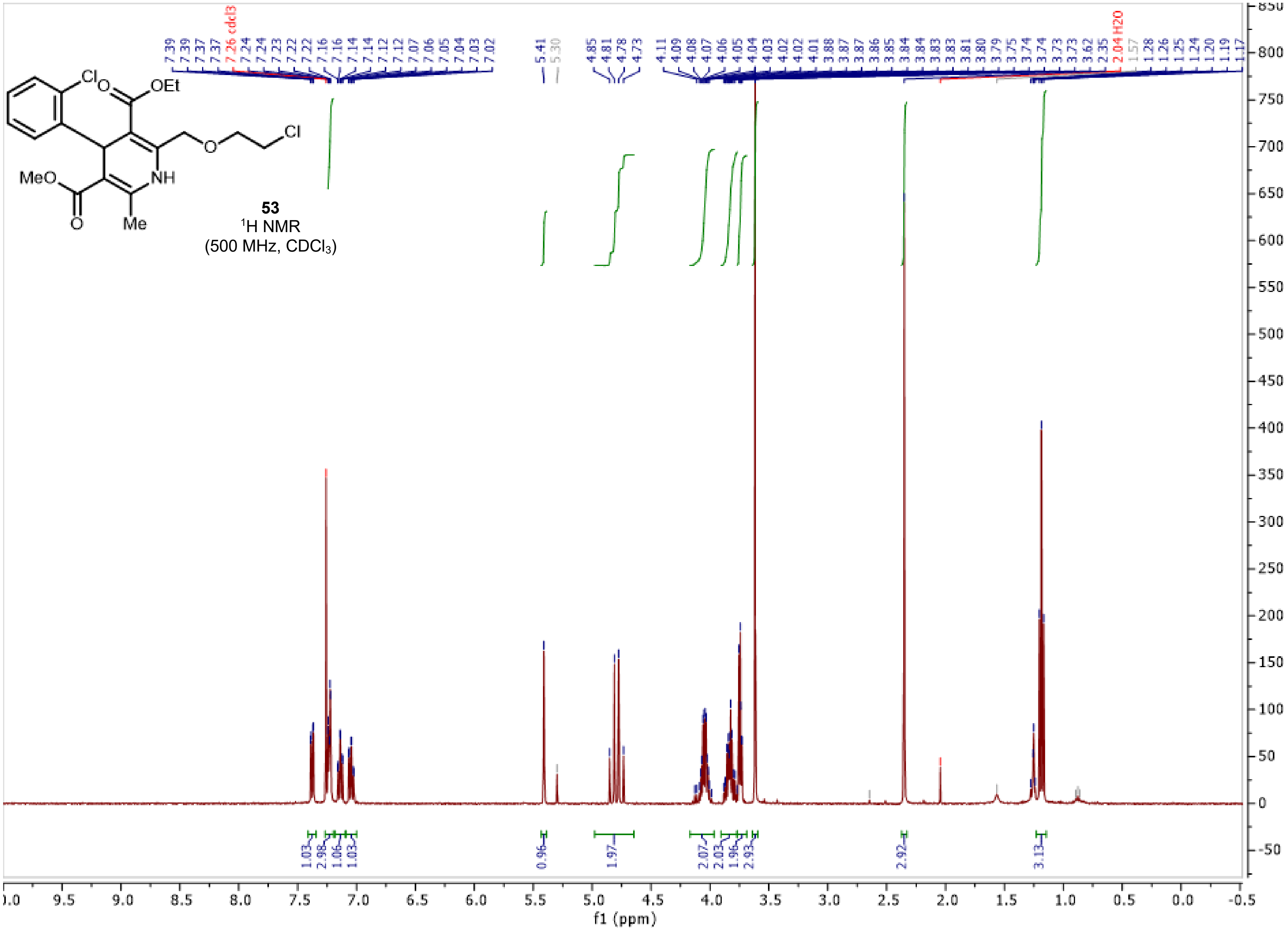

53
^{1}H NMR
(500 MHz, $CDCl_3$)

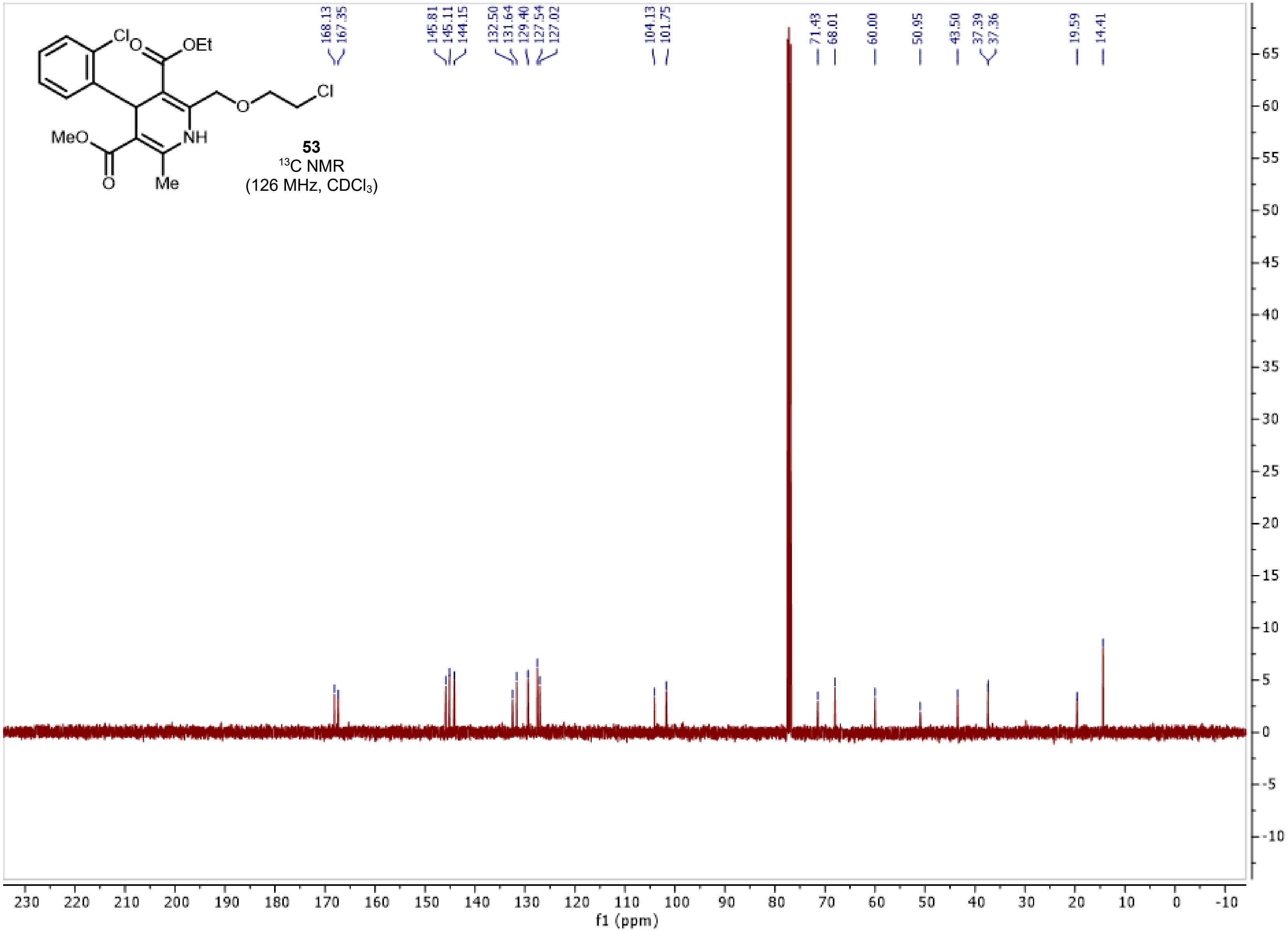
Cl
O
OEt
O
Cl
MeO
NH
O
Me
53
13C NMR
(126 MHz, CDCl3)
168.13
167.35
145.81
145.11
144.15
132.50
131.64
129.40
127.54
127.02
104.13
101.75
71.43
68.01
60.00
50.95
43.50
37.39
37.36
19.59
14.41
f1 (ppm)

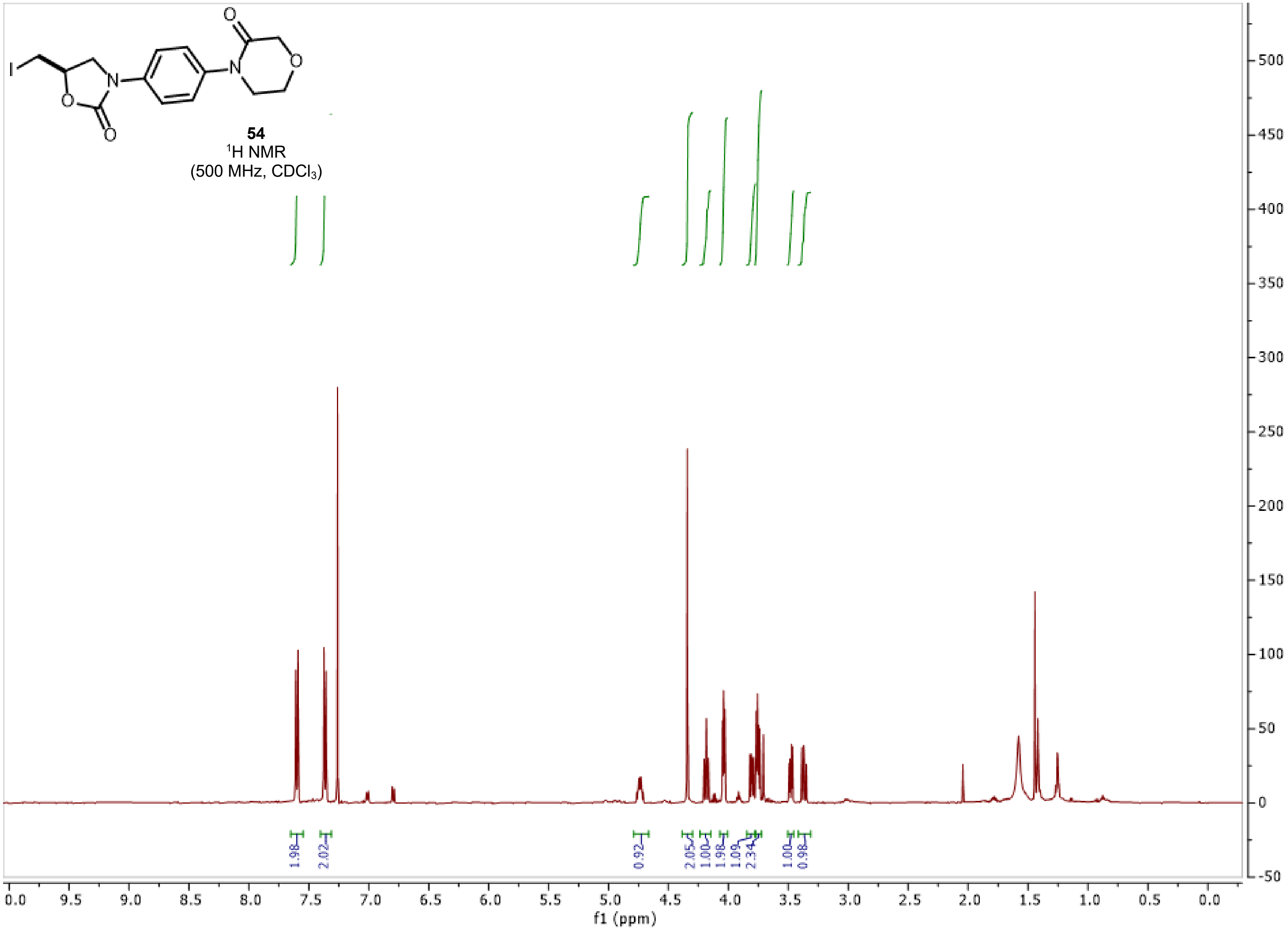
54
1H NMR
(500 MHz, CDCl3)
1.98
2.02
0.92
2.05
1.00
1.98
1.09
2.34
1.00
0.98
f1 (ppm)

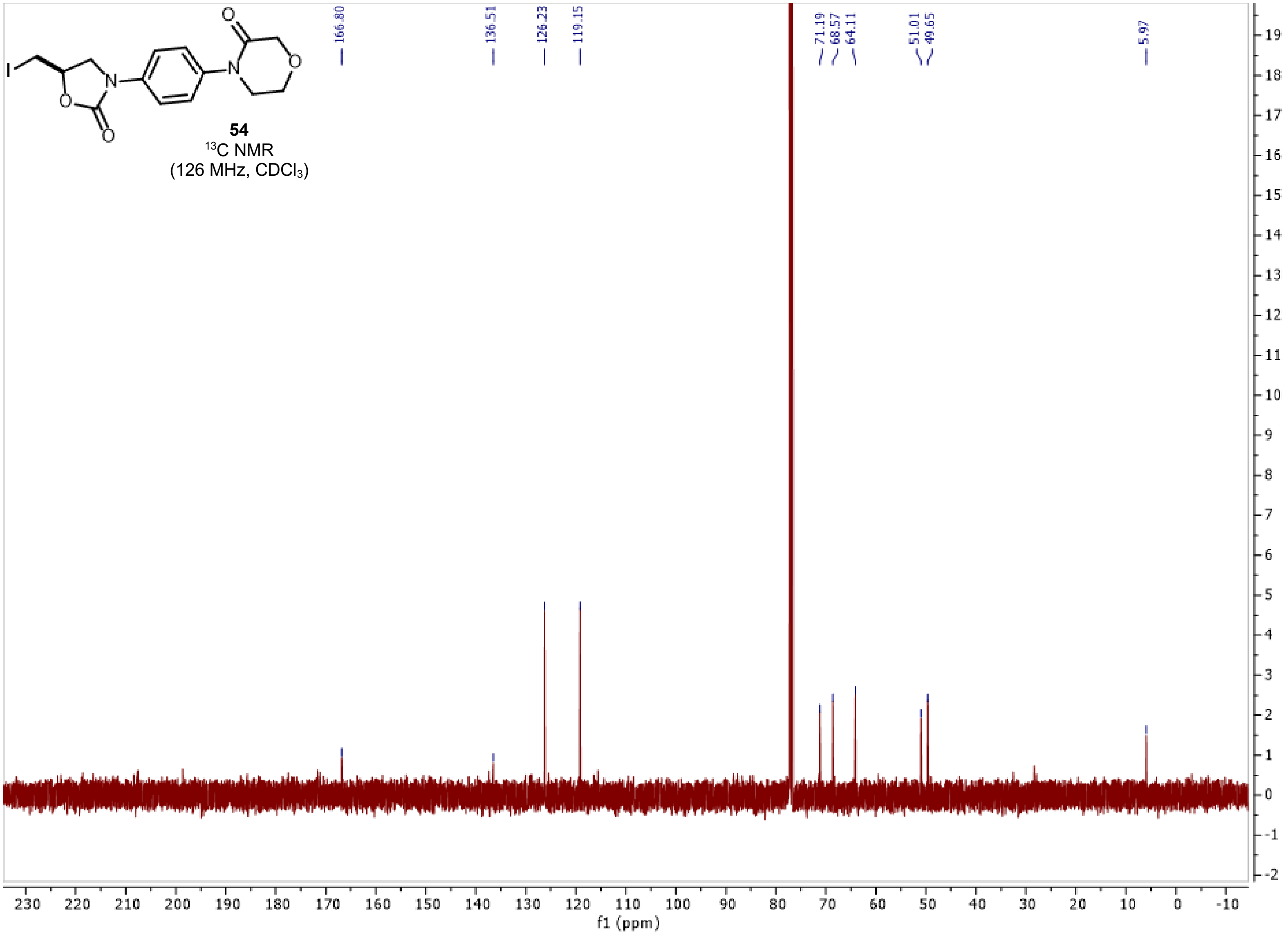
54
13C NMR
(126 MHz, CDCl3)
166.80
136.51
126.23
119.15
71.19
68.57
64.11
51.01
49.65
5.97
f1 (ppm)

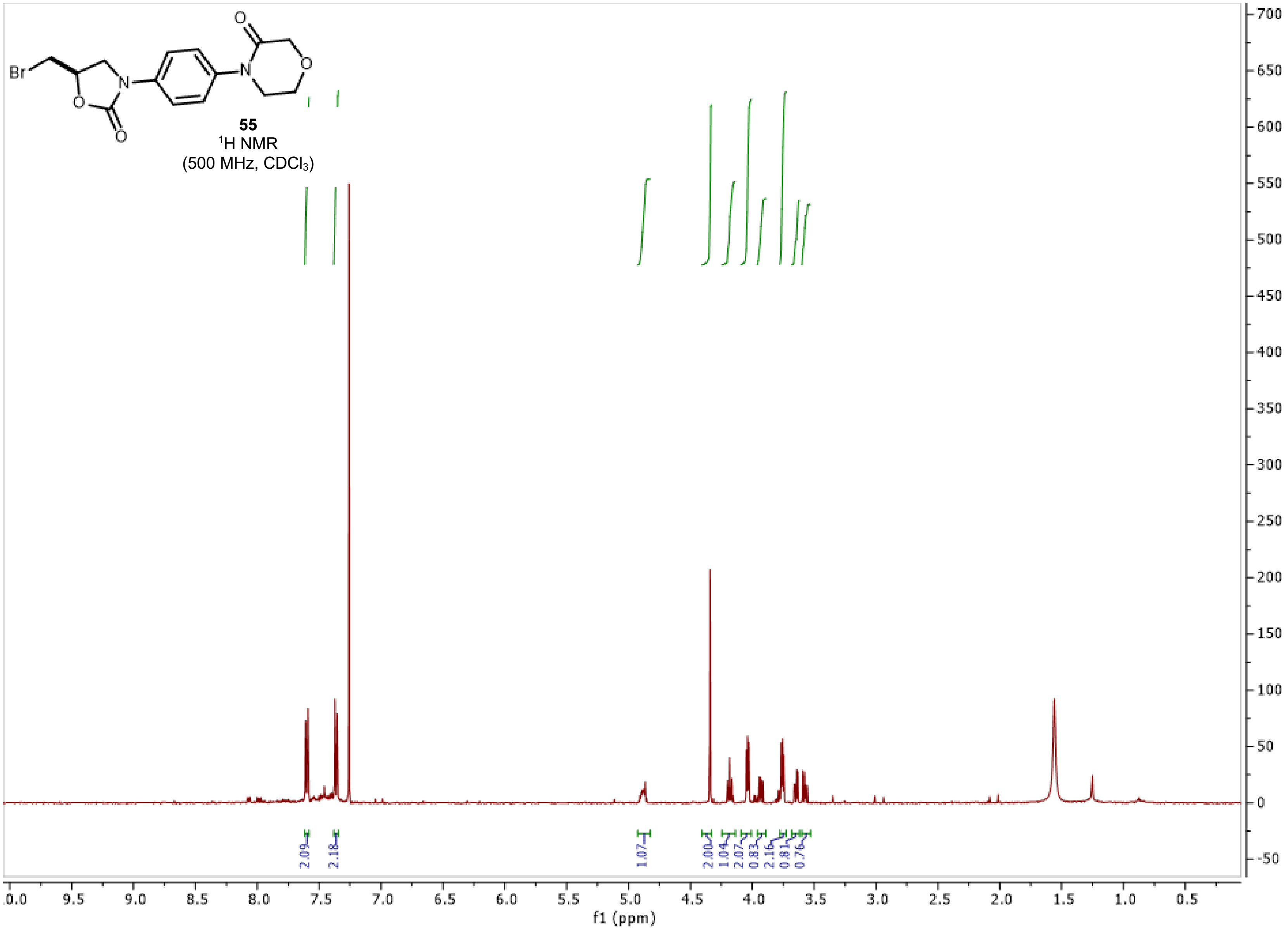

Br
N
O
O
N
O
O
55
1H NMR
(500 MHz, CDCl3)
2.09
2.18
1.07
2.00
1.04
2.07
0.83
2.16
0.81
0.76
f1 (ppm)

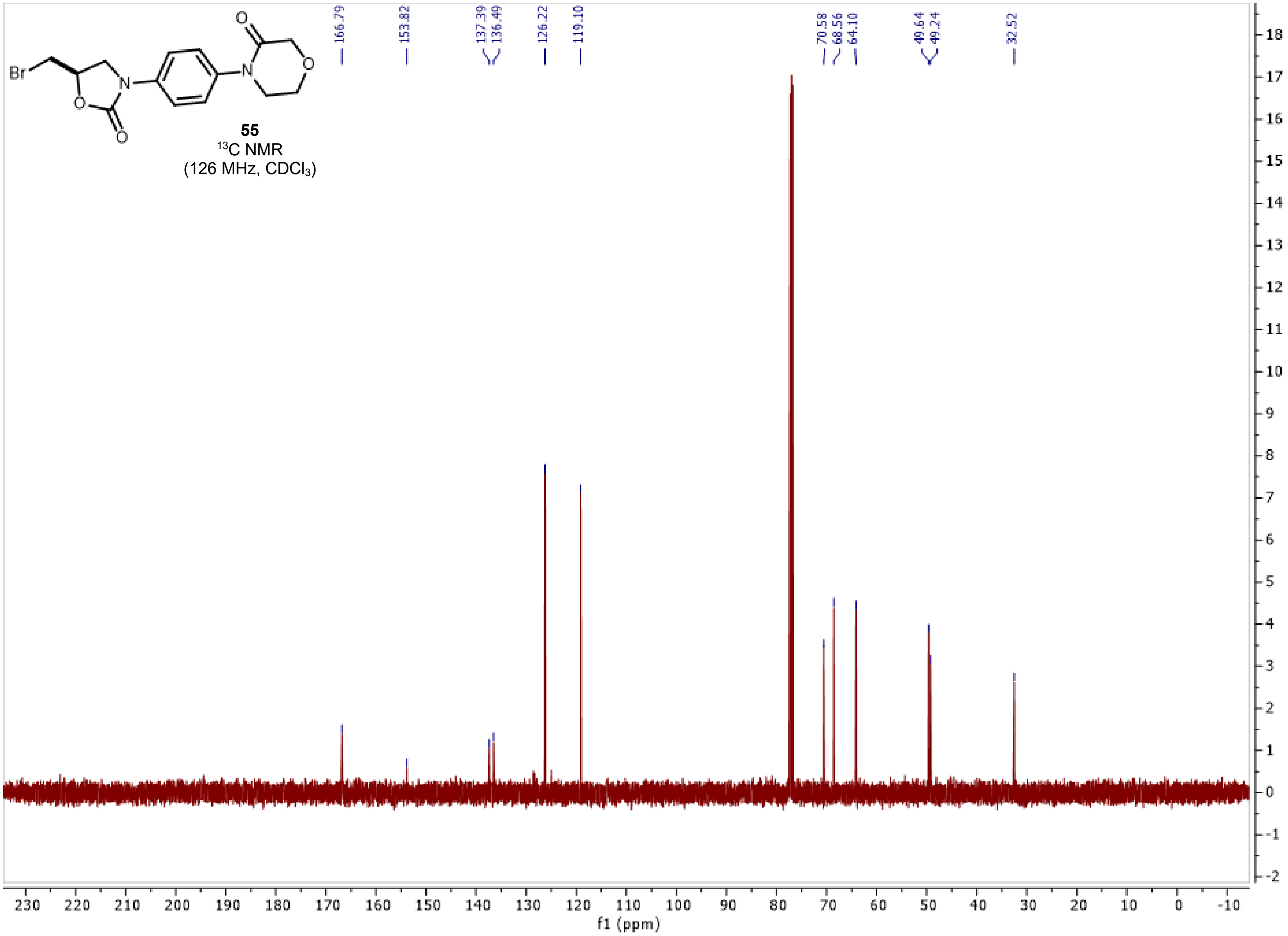

Br
N
O
O
O
N
O
55
13C NMR
(126 MHz, CDCl3)
166.79
153.82
137.39
136.49
126.22
119.10
70.58
68.56
64.10
49.64
49.24
32.52
f1 (ppm)

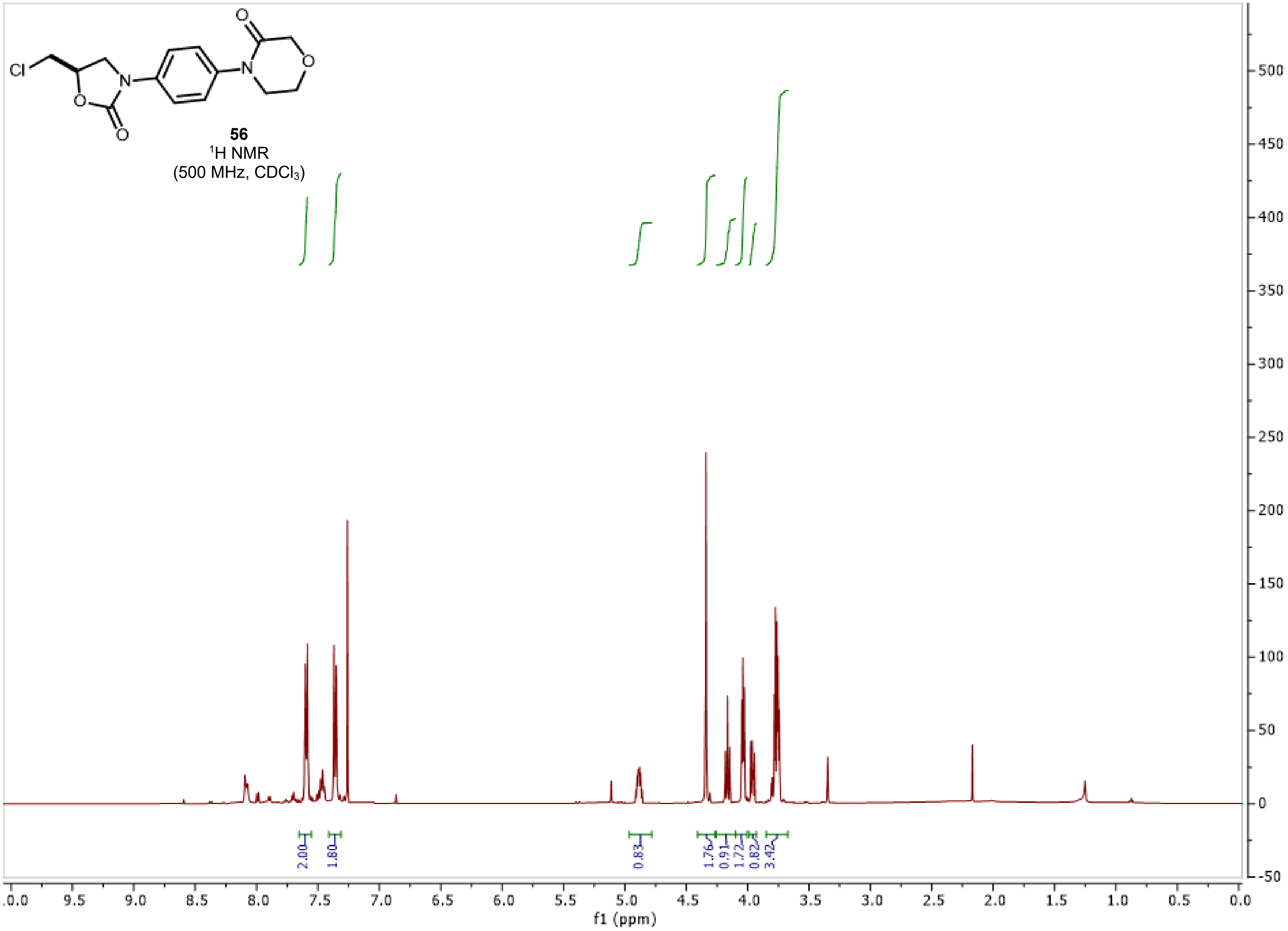

Cl
N
O
56
1H NMR
(500 MHz, CDCl3)
2.00
1.80
0.83
1.76
0.91
1.72
0.82
3.42
f1 (ppm)

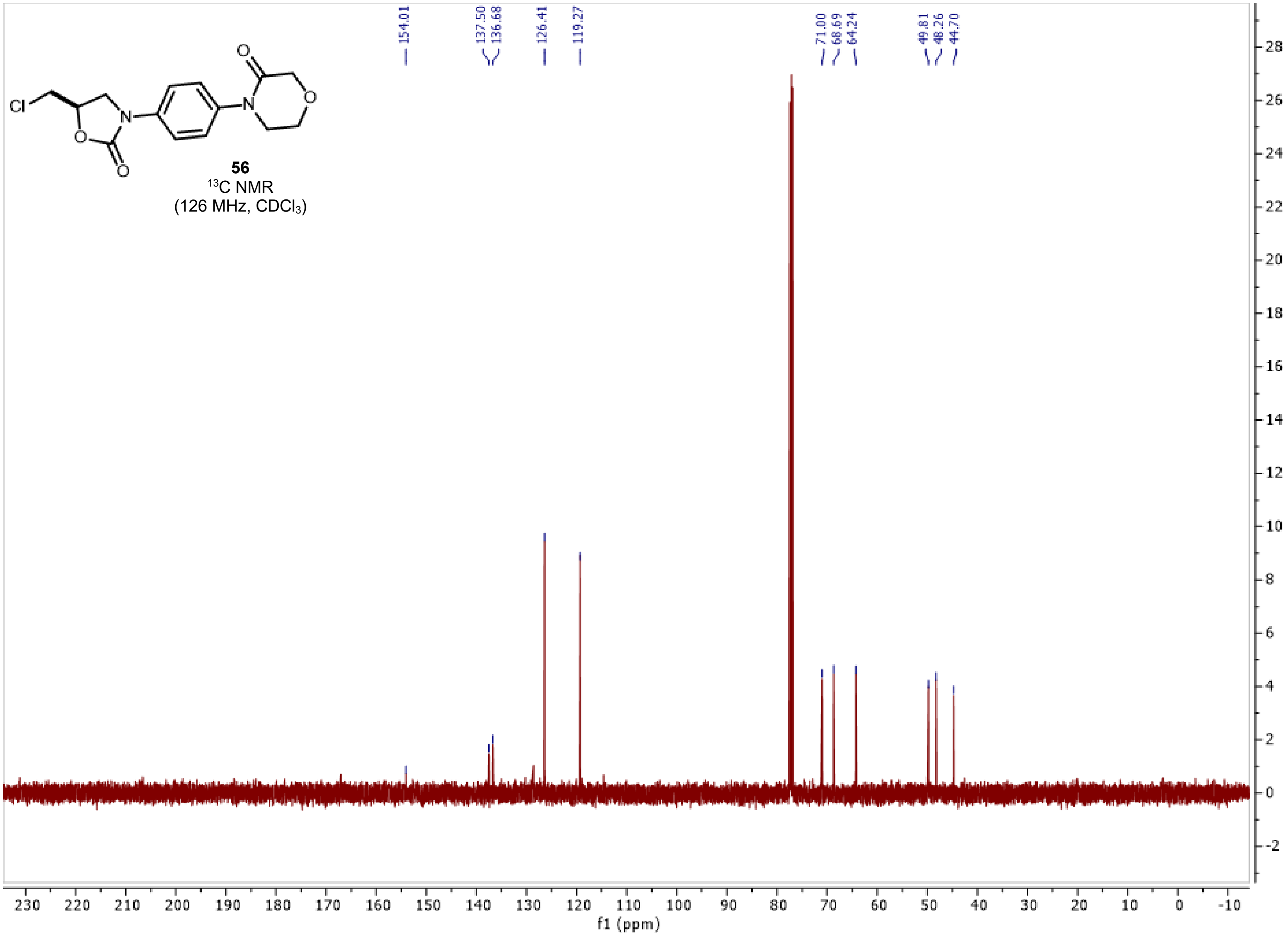
154.01
137.50
136.68
126.41
119.27
71.00
68.69
64.24
49.81
48.26
44.70
56
13C NMR
(126 MHz, CDCl3)
f1 (ppm)

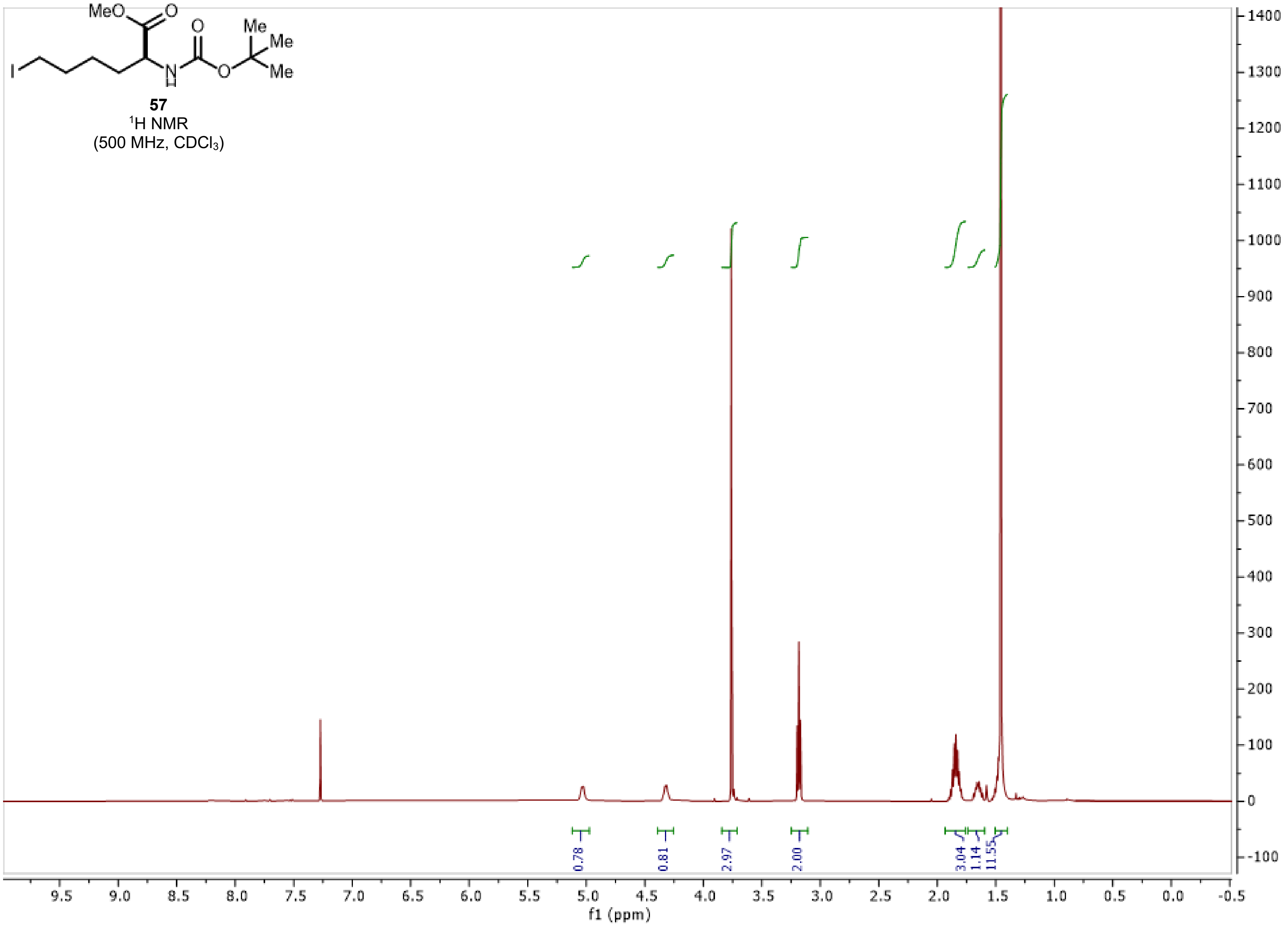
MeO
O
O
Me
Me
Me
I
N
H
O
57
^{1}H NMR
(500 MHz, $CDCl_3$)
0.78
0.81
2.97
2.00
3.04
1.14
11.55
f1 (ppm)

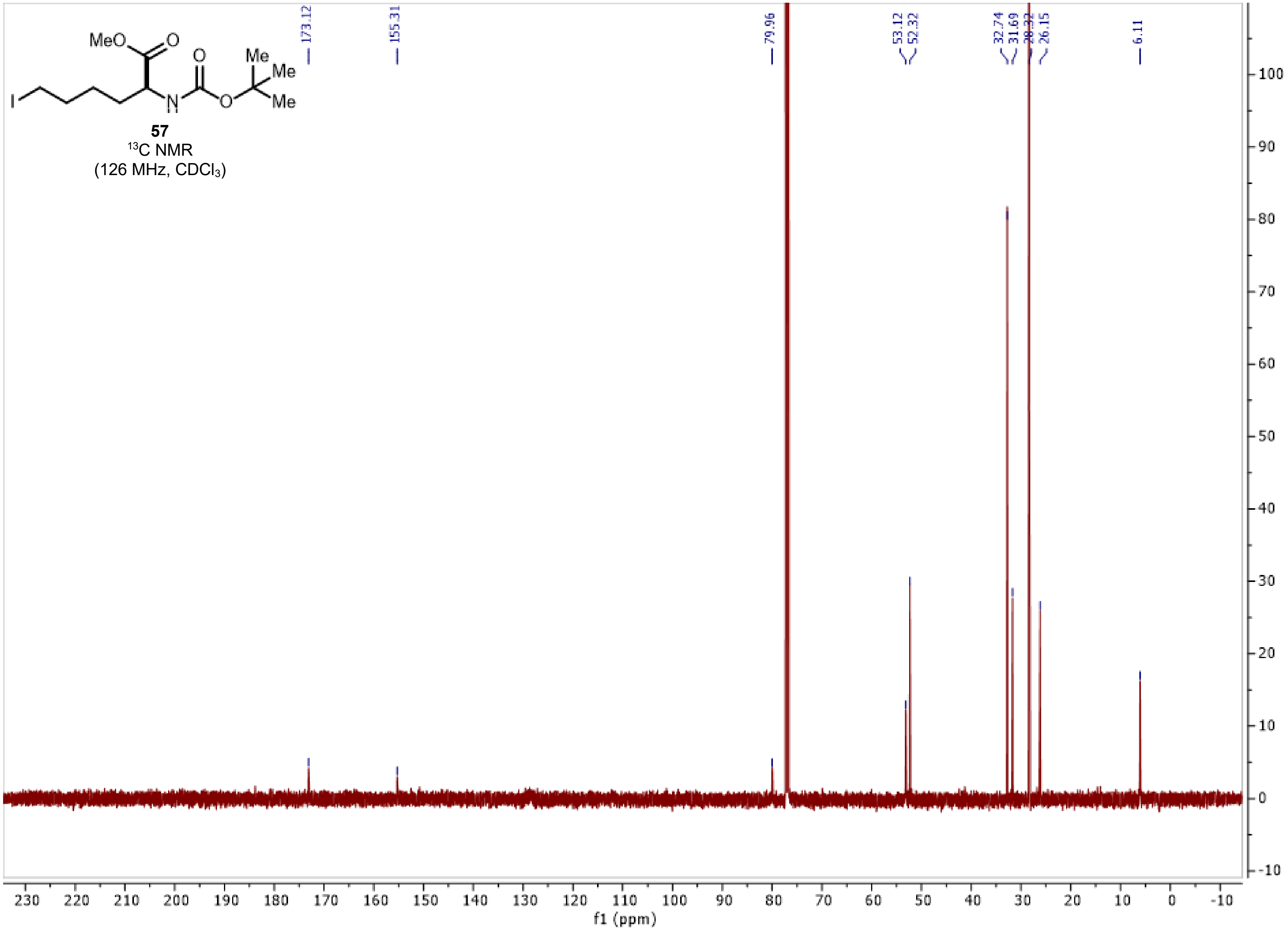

MeO
O
O
Me
Me
Me
I
N
H
O
57
^{13}C NMR
(126 MHz, $CDCl_3$)
173.12
155.31
79.96
53.12
52.32
32.74
31.69
28.32
26.15
6.11
f1 (ppm)

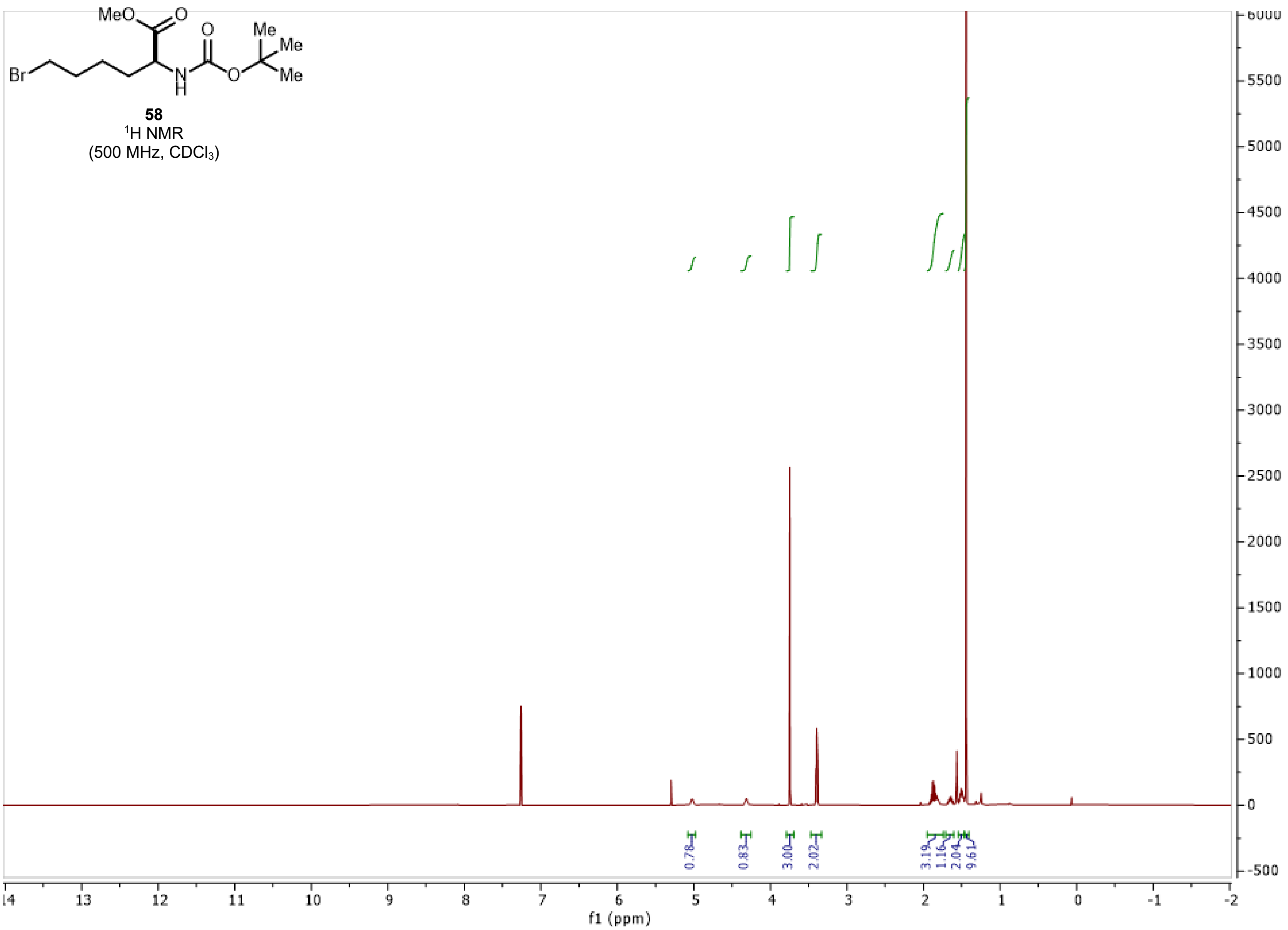
MeO
O
O
Me
Me
Br
N
H
O
Me
58
^{1}H NMR
(500 MHz, CDCl$_3$)
0.78
0.83
3.00
2.02
3.19
1.16
2.04
9.61
f1 (ppm)

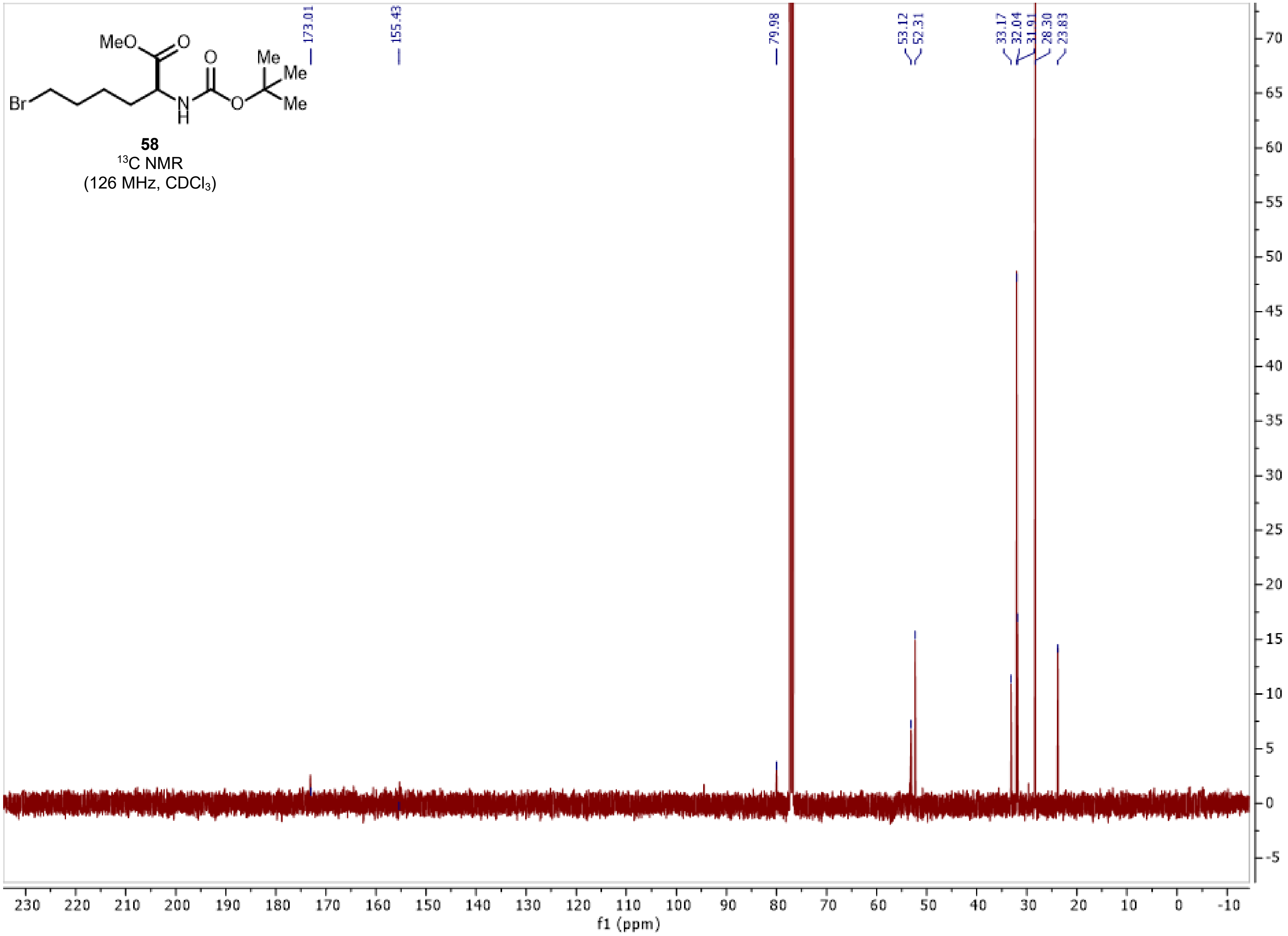
MeO
O
O
Me
Me
Me
Br
N
H
O
58
^{13}C NMR
(126 MHz, CDCl$_3$)
173.01
155.43
79.98
53.12
52.31
33.17
32.04
31.91
28.30
23.83
f1 (ppm)

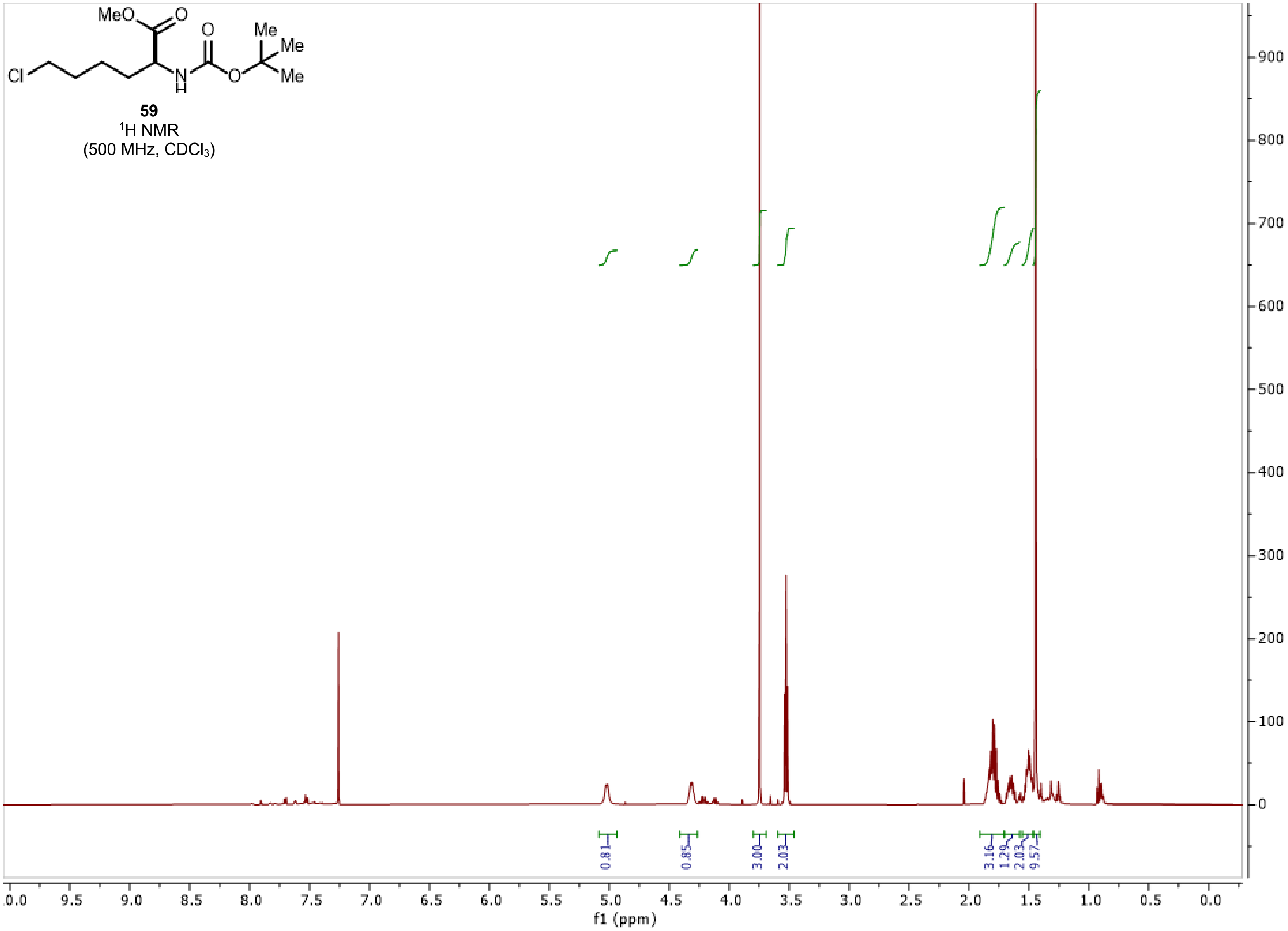
MeO
O
O
Me
Me
Me
Cl
N
H
O
59
1H NMR
(500 MHz, CDCl3)
0.81
0.85
3.00
2.03
3.16
1.29
2.03
9.57
f1 (ppm)

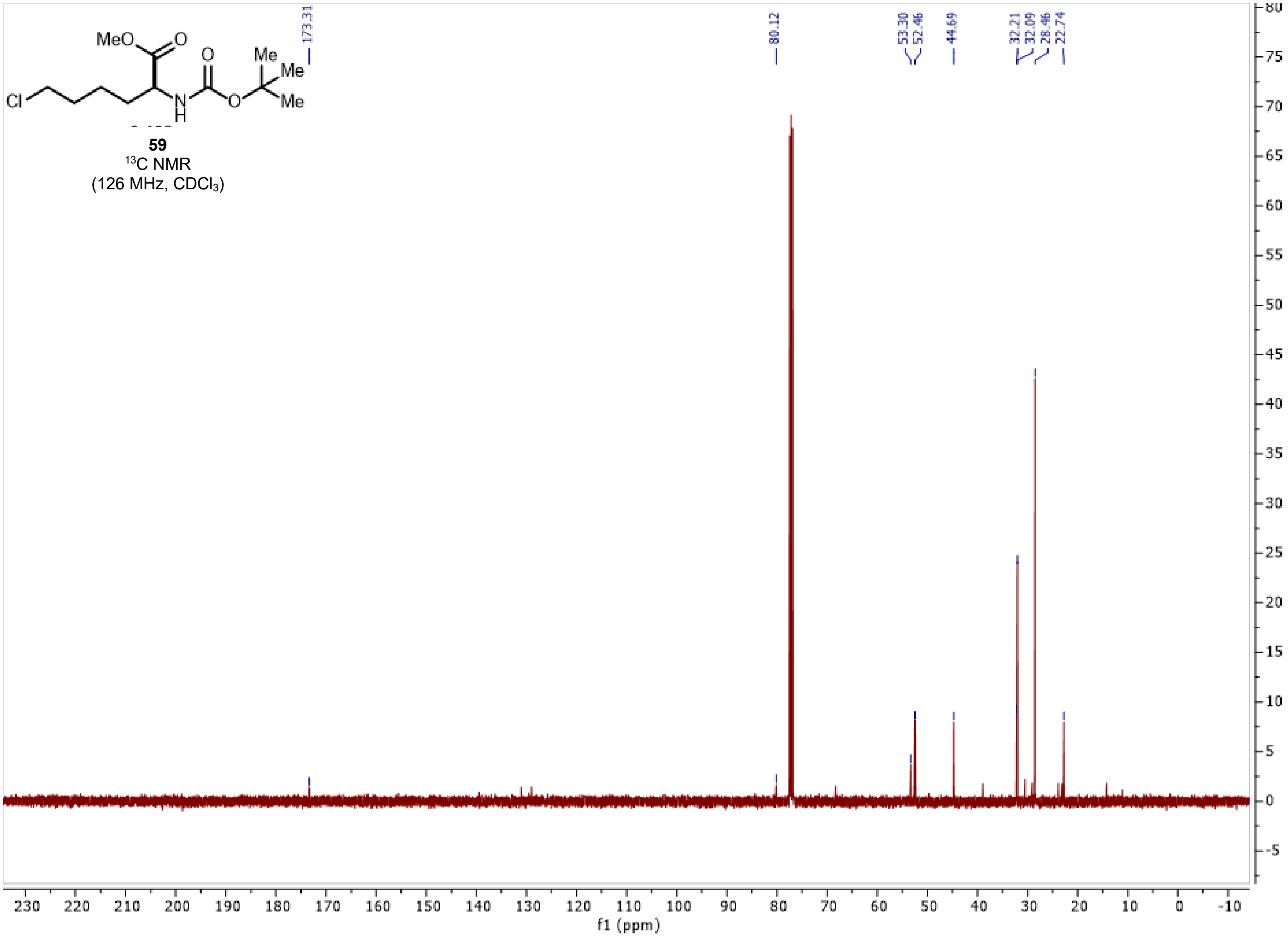
MeO
O
O
Me
Me
Cl
N
H
O
Me
59
^{13}C NMR
(126 MHz, CDCl$_3$)
173.31
80.12
53.30
52.46
44.69
32.21
32.09
28.46
22.74
f1 (ppm)

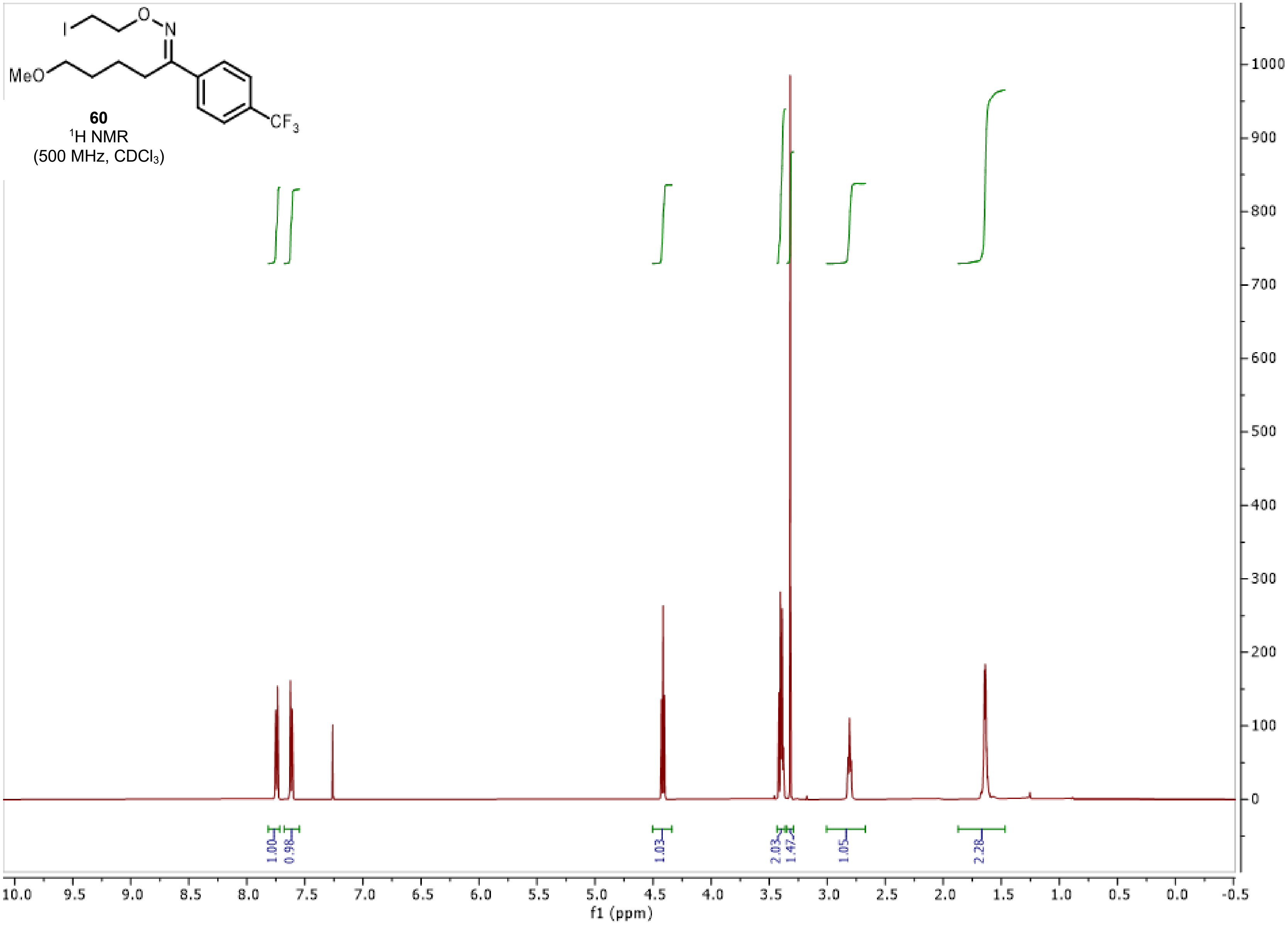

MeO
I
O
N
CF3
60
1H NMR
(500 MHz, CDCl3)
1.00
0.98
1.03
2.03
1.47
1.05
2.28
f1 (ppm)

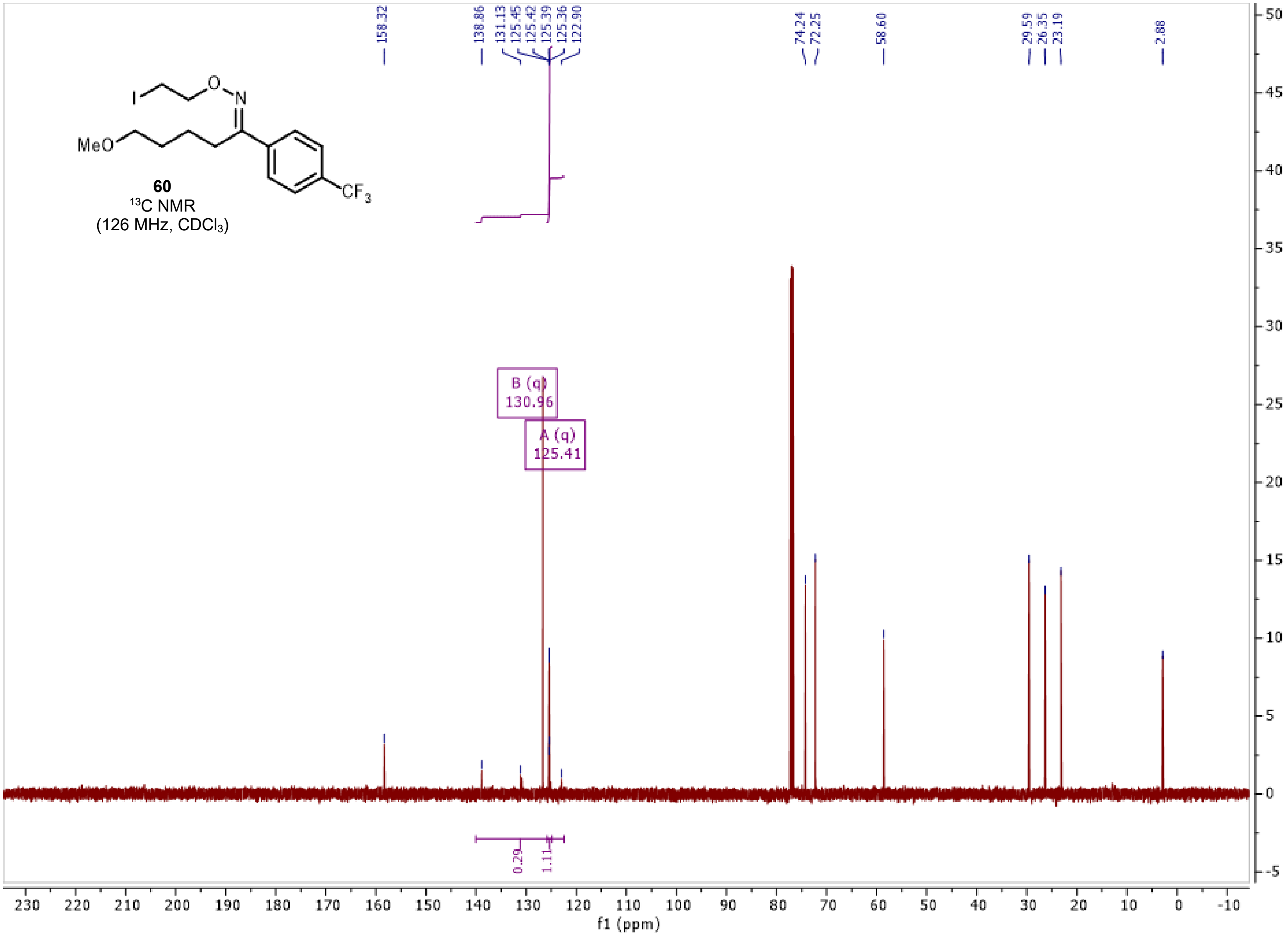

MeO
I
O
N
CF3
60
13C NMR
(126 MHz, CDCl3)
158.32
138.86
131.13
125.45
125.42
125.39
125.36
122.90
74.24
72.25
58.60
29.59
26.35
23.19
2.88
B (q)
130.96
A (q)
125.41
0.29
1.11
f1 (ppm)

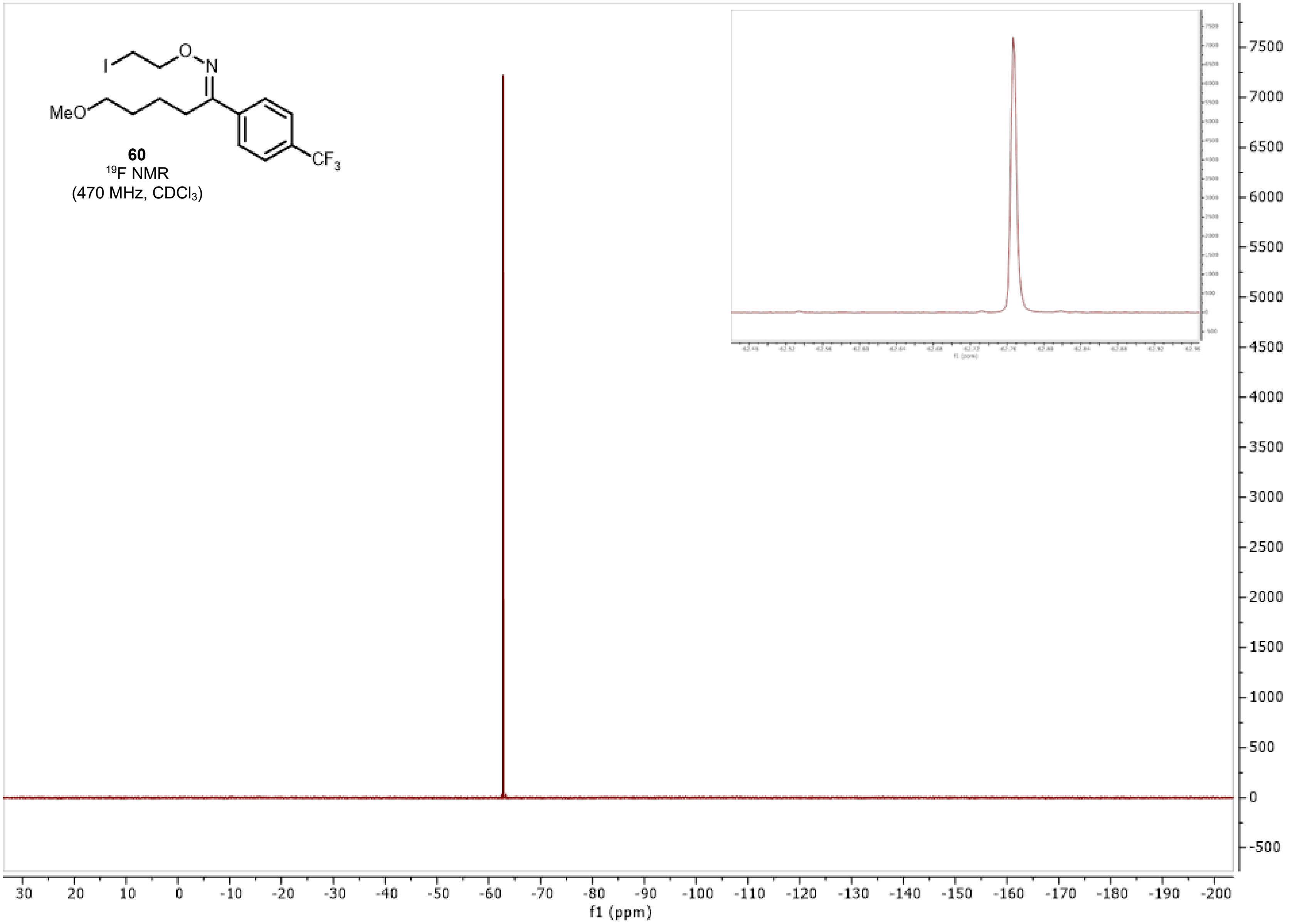
MeO
I
O
N
CF3
60
19F NMR
(470 MHz, CDCl3)
f1 (ppm)

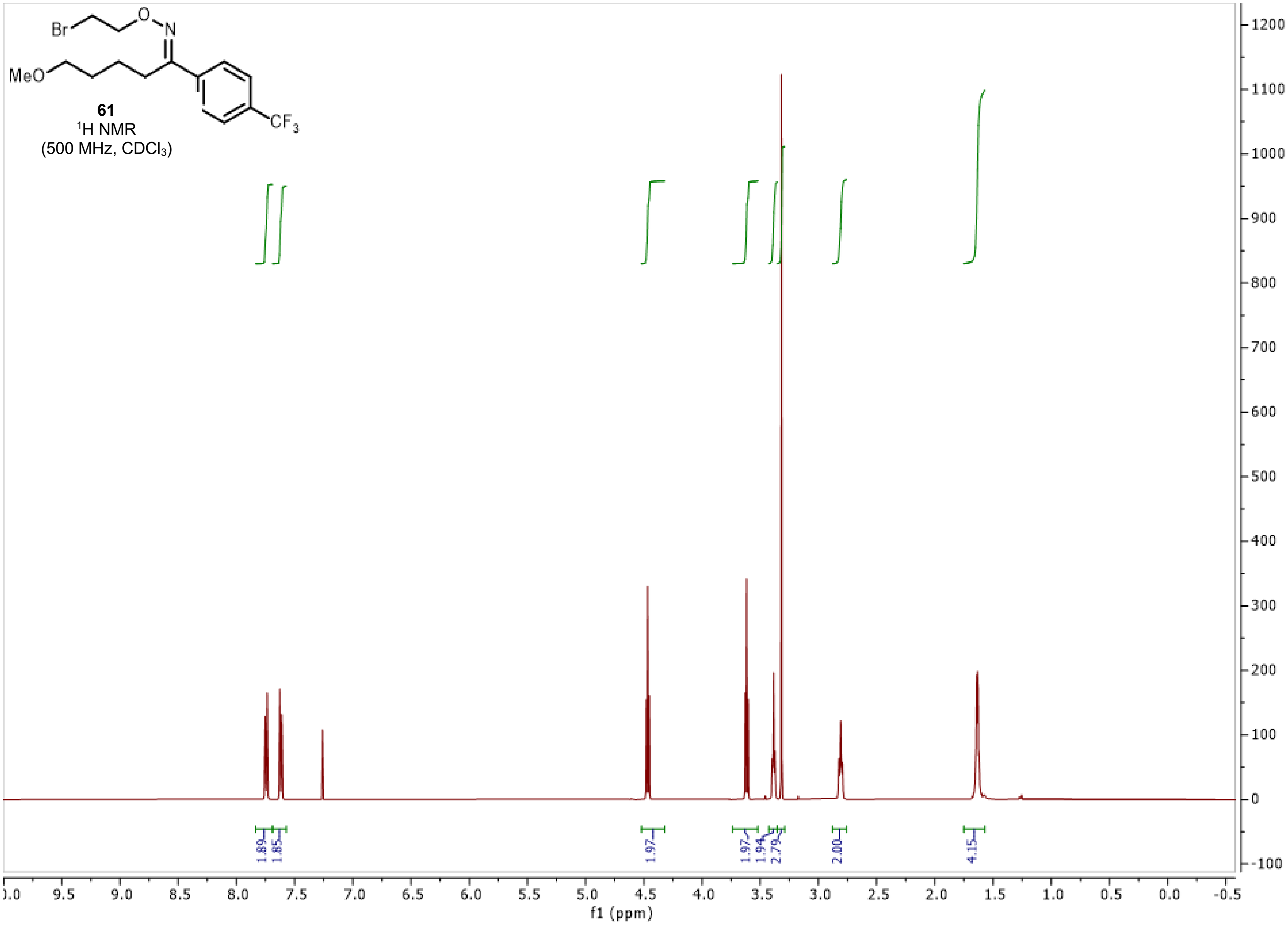

Br
O
N
MeO
CF3
61
1H NMR
(500 MHz, CDCl3)
1.89
1.85
1.97
1.97
1.94
2.79
2.00
4.15
f1 (ppm)

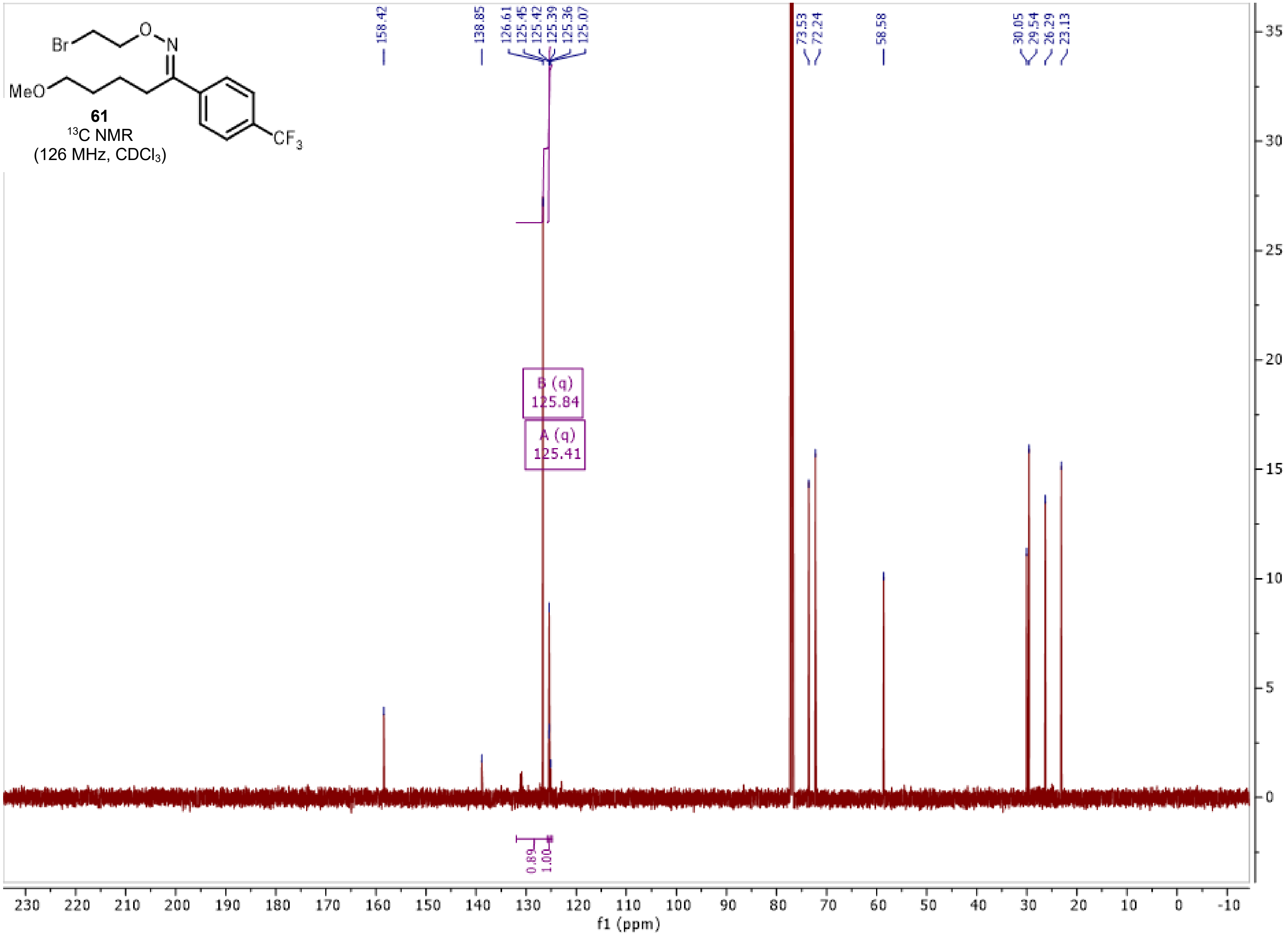

61
13C NMR
(126 MHz, CDCl3)
158.42
138.85
126.61
125.45
125.42
125.39
125.36
125.07
73.53
72.24
58.58
30.05
29.54
26.29
23.13
B (q)
125.84
A (q)
125.41
0.89
1.00
f1 (ppm)

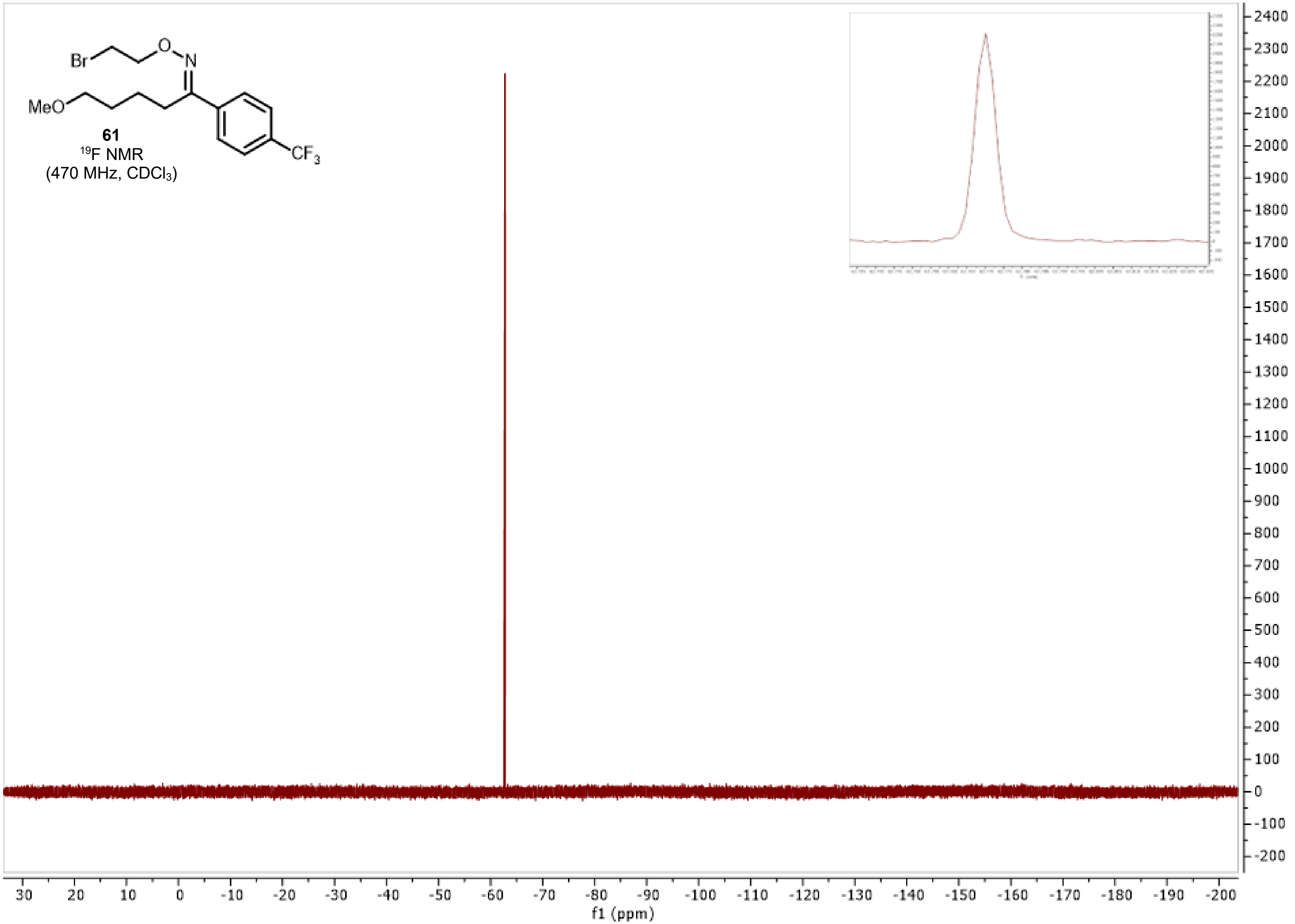

61

^{19}F NMR

(470 MHz, $CDCl_3$)

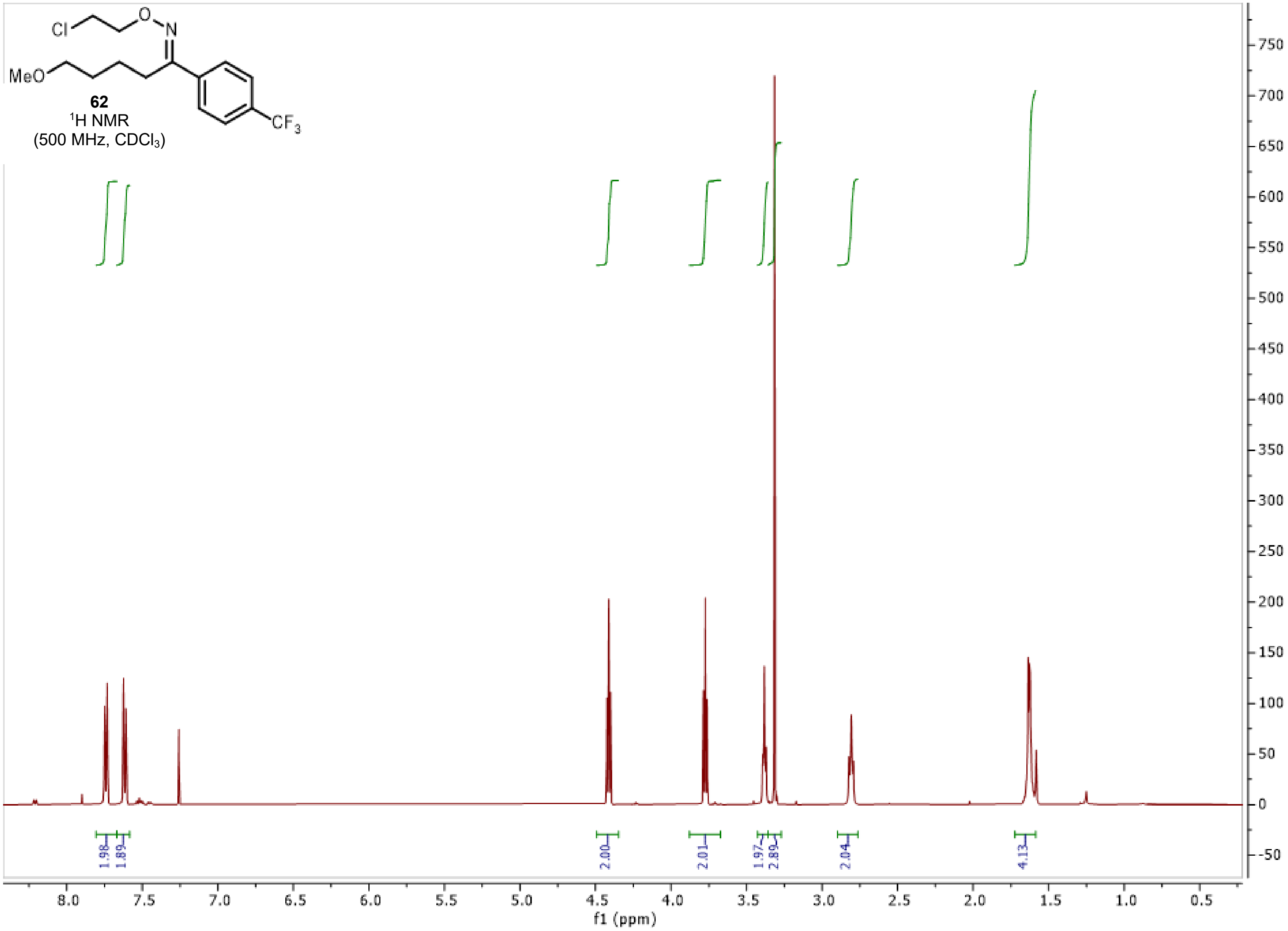
Cl
O
N
MeO
CF3
62
1H NMR
(500 MHz, CDCl3)
1.98
1.89
2.00
2.01
1.97
2.89
2.04
4.13
f1 (ppm)

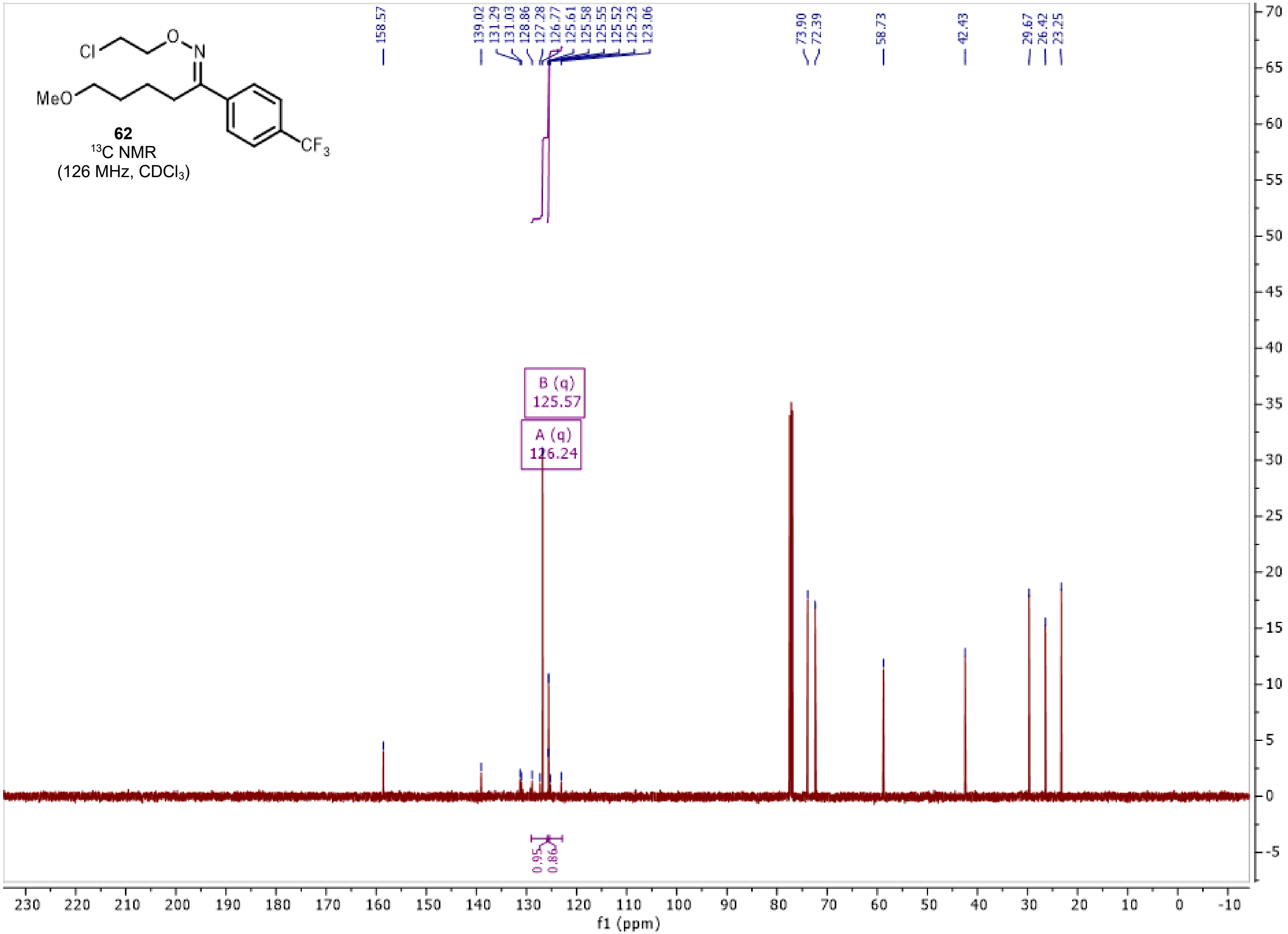
Cl
O
N
MeO
CF3
62
13C NMR
(126 MHz, CDCl3)
158.57
139.02
131.29
131.03
128.86
127.28
126.77
125.61
125.58
125.55
125.52
125.23
123.06
73.90
72.39
58.73
42.43
29.67
26.42
23.25
B (q)
125.57
A (q)
126.24
0.95
0.86
f1 (ppm)

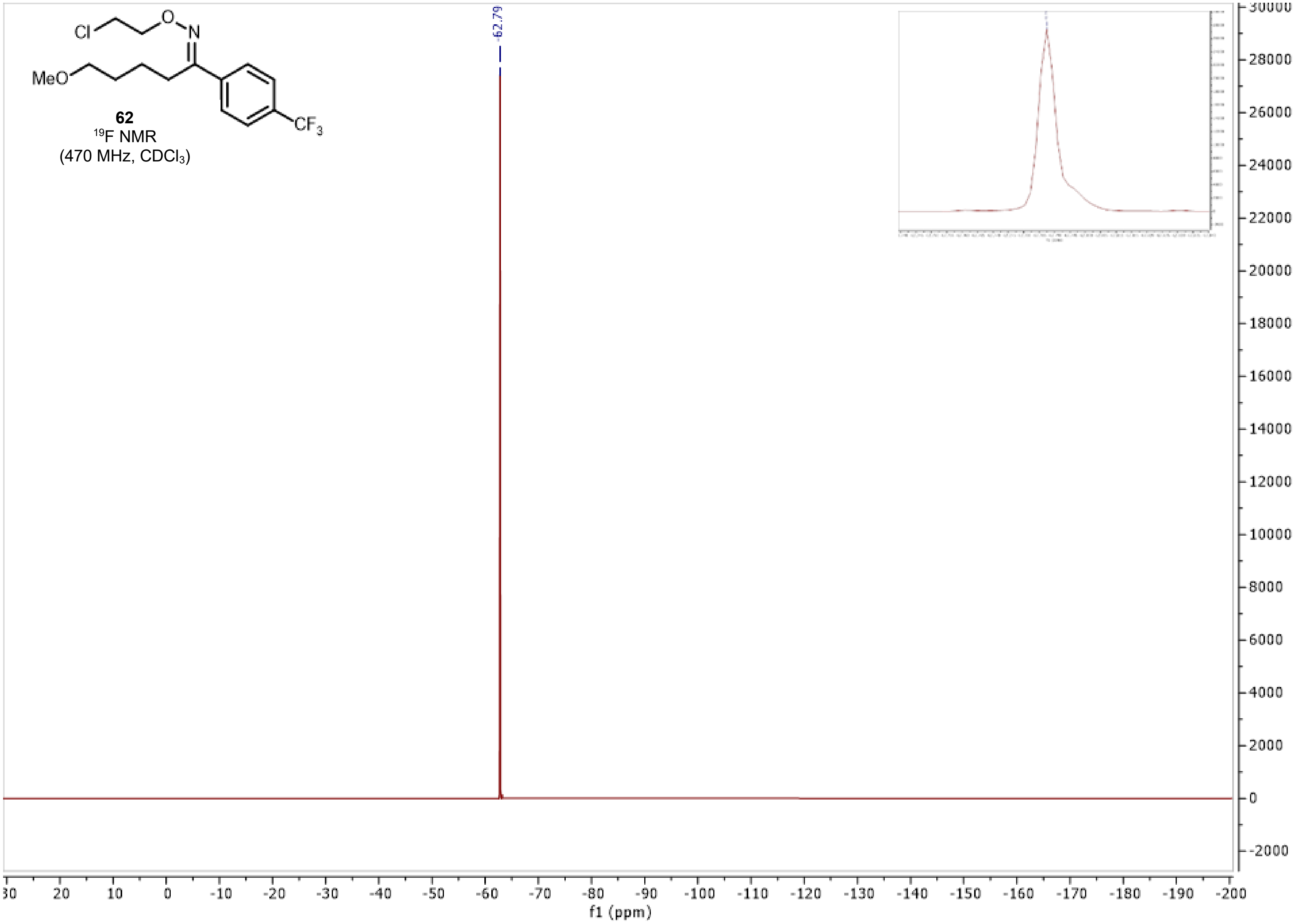

Cl
O
N
MeO
CF3
62
19F NMR
(470 MHz, CDCl3)
-62.79
f1 (ppm)

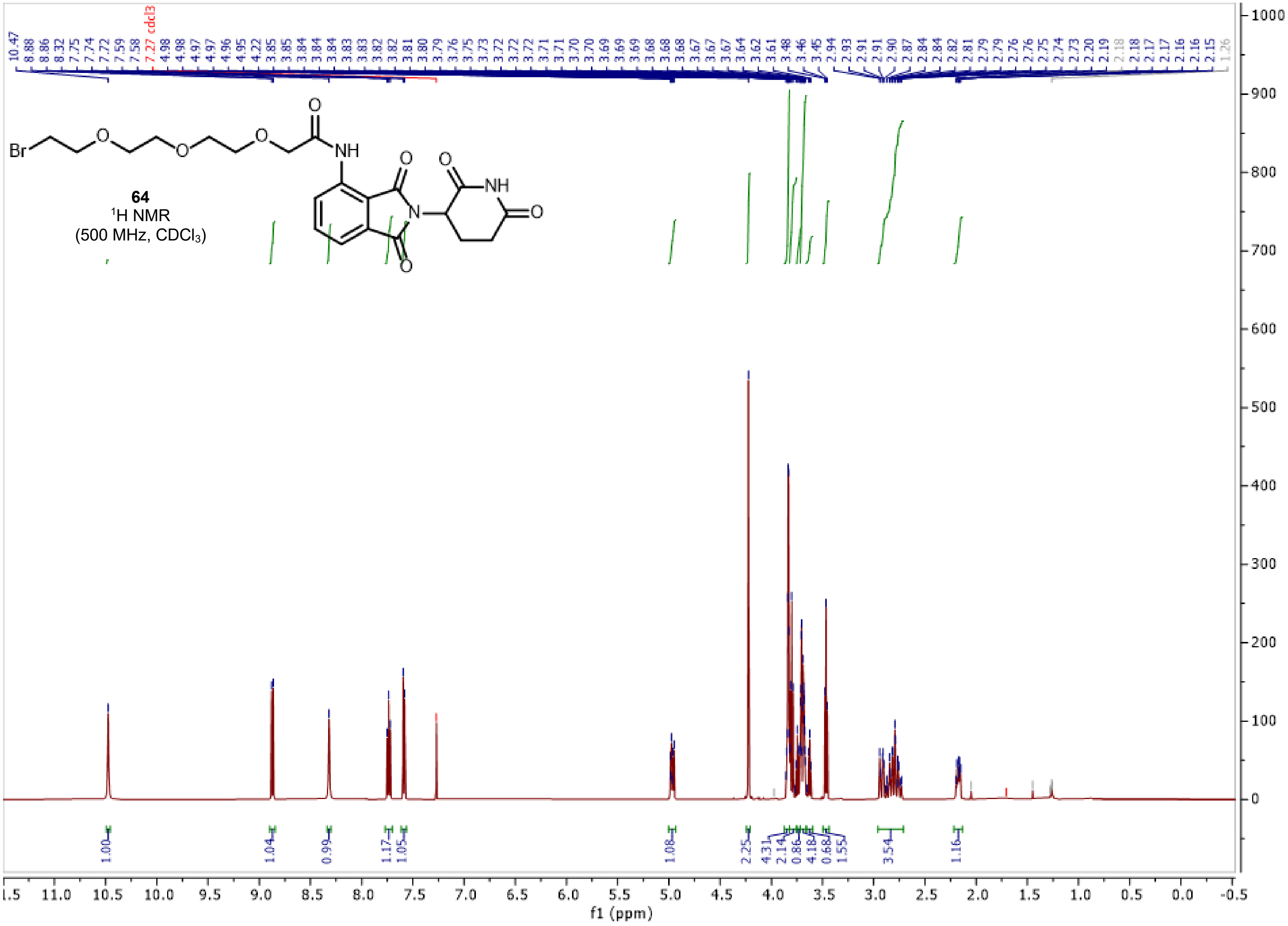
64
¹H NMR
(500 MHz, CDCl3)
f1 (ppm)

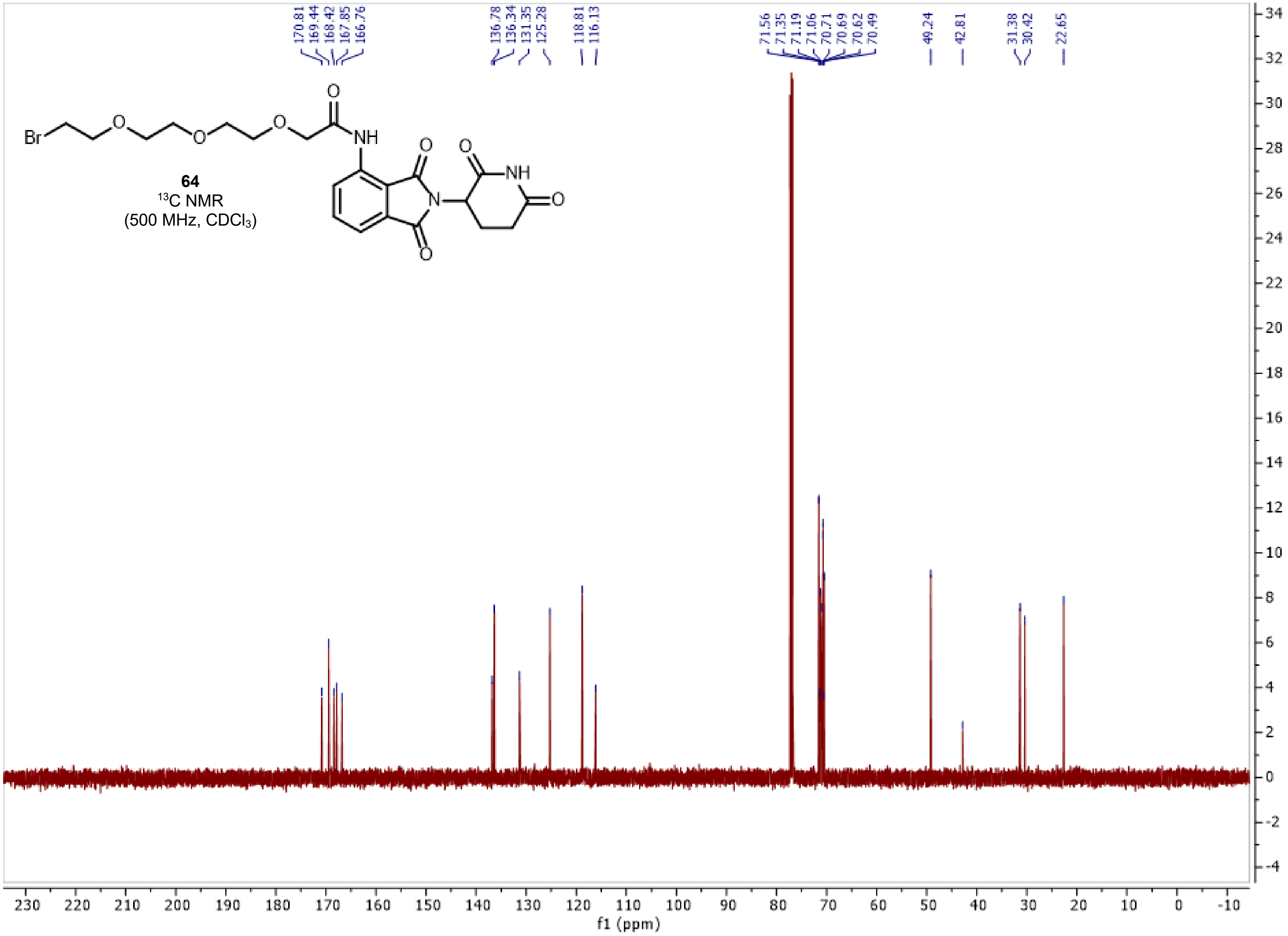
64
^{13}C NMR
(500 MHz, CDCl$_3$)
170.81
169.44
168.42
167.85
166.76
136.78
136.34
131.35
125.28
118.81
116.13
71.56
71.35
71.19
71.06
70.71
70.69
70.62
70.49
49.24
42.81
31.38
30.42
22.65
f1 (ppm)

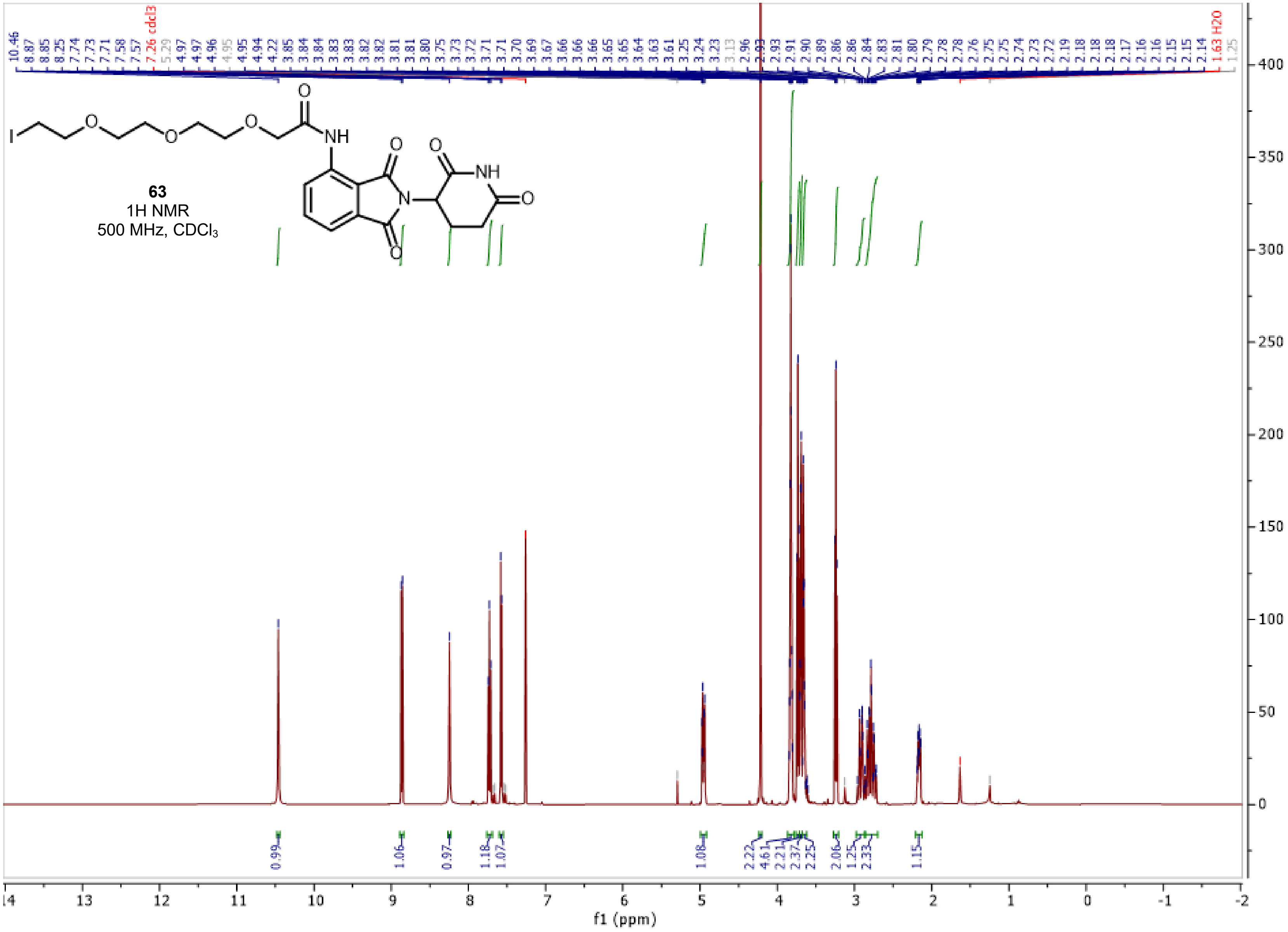
63
1H NMR
500 MHz, CDCl3
f1 (ppm)

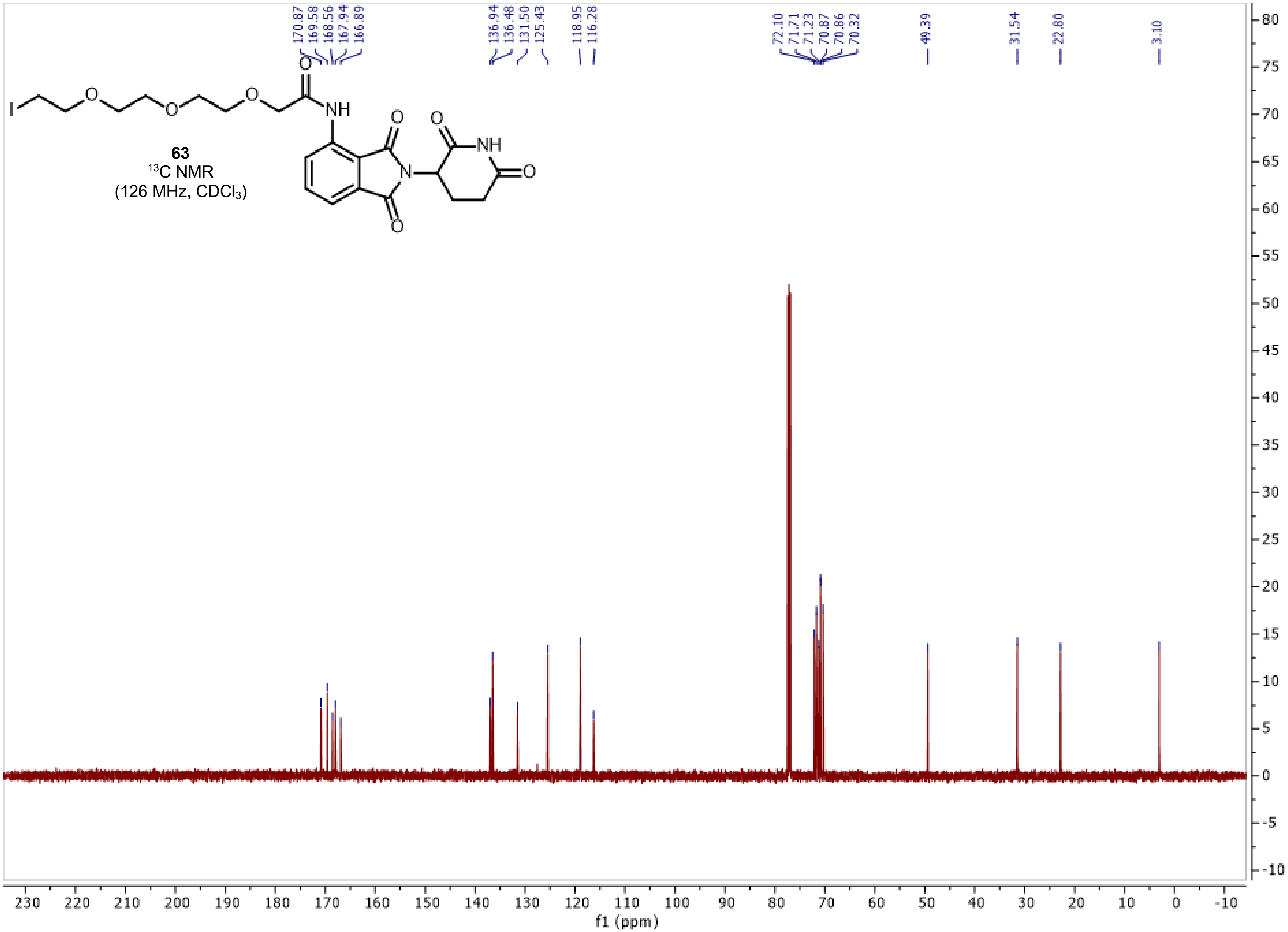
170.87
169.58
168.56
167.94
166.89
136.94
136.48
131.50
125.43
118.95
116.28
72.10
71.71
71.23
70.87
70.86
70.32
49.39
31.54
22.80
3.10
63
13C NMR
(126 MHz, CDCl3)
f1 (ppm)
